# Natural variation of bioactive gibberellin levels in maize tissues across North America

**DOI:** 10.1371/journal.pone.0340156

**Published:** 2026-10-07

**Authors:** Xia Guan, Nitin Supekar, Changjian Jiang, Junhong Guo, Edwards Allen, Charles Dietrich, Cody Postin, Shubha Shakya, Yanfei Wang

**Affiliations:** Regulatory Science, Bayer CropScience LLC., Chesterfield, Missouri, United States of America; University of Massachusetts Amherst, UNITED STATES OF AMERICA

## Abstract

Gibberellins (GA) are plant hormones that regulate growth and development throughout the plant life cycle. While gibberellins have been extensively studied under controlled conditions for their fundamental role in plant adaptation and agronomic trait development, field-based data on their natural variation of bioactive GA levels across diverse agricultural genotypes and environments remains limited. To address this knowledge gap, we analyzed bioactive gibberellin A1 (GA_1_) and gibberellin A4 (GA_4_) in multiple maize tissues from commercial conventional maize hybrids grown in diverse field environments using high-throughput liquid chromatography-tandem mass spectrometry (LC-MS/MS). This study provides quantitative measurements of GA_1_ and GA_4_ levels in maize tissues collected under standard agricultural practices and describes variation influenced by genetic background and environmental conditions. Our findings demonstrate that GA_1_ and GA_4_ concentrations from conventional maize samples varied across maize tissue types, hybrid, and field sites. Analysis of variance components indicated that the site variation was statistically significant in five of ten gibberellins by maize tissue combinations, while the genetic component was significant only for GA_4_ in grain samples, suggesting that environment factors have a larger effect than genetic factors Positive correlations between GA_1_ and GA_4_ were observed consistently across maize tissues except root. These findings offer preliminary insights into the interplay between genetics and environment, across germplasm and environments that have been previously described.

## Introduction

Gibberellins (GA) are plant hormones that play a crucial role in regulating plant growth and development throughout the plant life cycle. They were first discovered as secondary metabolites in the fungus *Fusarium fujikuroi* [1,2]. Gibberellins comprise a class of diterpenoid carboxylic acids that possess the tetracyclic ent-gibberellane carbon skeletal structure. Among various gibberellins, only a few, e.g., GA_1_, GA_3_, GA_4_, and GA_7_ exhibit significant biological activity [3]. GA_1_ and GA_4_ are the major active gibberellins that promote growth and development in plant species. They are involved in diverse developmental processes such as seed germination, stem elongation, leaf expansion, and flower and fruit development [2,4]. Additionally, gibberellins modulate plant adaptation to both biotic and abiotic stresses [5,6].

The biosynthesis and signaling pathways of gibberellins are intricate systems that exhibit complex spatiotemporal patterns across cells, tissues, and developmental stages [7,8]. These patterns result from multiple enzymatic steps and feedback mechanisms that respond dynamically to both biological and environmental signals [9,10]. Environmental conditions can influence measured gibberellin pools through effects on biosynthesis, catabolism, conjugation, transport, or signaling [11], leading to stunted growth and altered development, while optimal conditions enhance gibberellin production and promote vigorous growth [12]. The movement of gibberellins between cells and across plant organs, coupled with their variable distribution patterns, creates challenges for understanding hormone-environment interactions in agricultural settings [13–15].

Due to the vital biological activity of gibberellins in plant growth and development, several applications have been developed for commercial and biotechnological use: applications of gibberellins to promote growth, and applications or the genetic modification of biosynthesis to optimize plant phenotypes and environmental responses [10]. Research in recent years has shown that gibberellins have direct and indirect effects on many plant traits. Many plant varieties with improved agronomic traits (e.g., dwarf phenotypes or increased biomass) are shown to be related to gibberellin activity and signaling [16], thus manipulation of gibberellin levels is commonly used in agricultural practices to optimize plant growth and yield.

Maize (*Zea mays* L.; corn) is a major agricultural commodity, contributing significantly to the economy of many countries and serves as a staple food for many populations worldwide [17]. Short maize hybrids have been created through conventional breeding by researchers with the objectives to improve performance under high density and improve lodging tolerance [18]. However, breeding short traits into existing maize hybrids is time consuming, challenging, and can affect environmental responses. Genetically modified maize can be engineered through the enhancement or suppression of hormone pathways, including gibberellin biosynthesis. Scientists have developed a genetically modified short stature maize ideotype by suppressing genes that are involved in the levels of gibberellins, reducing plant stature without undermining maize yields [19].

Accurate quantification of gibberellin levels is valuable for describing variation across tissues, genotypes, and environments and for providing baseline context for future studies [20]. While previous studies have quantified bioactive gibberellins in maize tissues [8,19,21–24], these analyses were typically limited to specific varieties or locations, lacking the scope needed to characterize natural variation across diverse agricultural conditions.

Establishing baseline patterns of gibberellin abundance in agricultural settings can support subsequent research. First, understanding the natural variability of gibberellin concentrations in conventional maize tissues – a primary determinant of plant stature – and distinguishing between environmentally-induced and genetically-controlled variation provides essential context for crop improvement efforts. This baseline knowledge is particularly valuable for developing and evaluating new short-stature varieties, where gibberellin-related genetic modifications must be assessed against the background of natural gibberellin variability that occurs across diverse field environments.

Second, while gibberellin biology has been extensively studied under controlled conditions [19], quantifying the magnitude of gibberellin variation in commercial hybrids under real-world agricultural conditions helps bridge the gap between laboratory findings and field performance. Such information may provide context for future studies evaluating gibberellin-modified traits across growing environments, where environmental stresses can significantly impact gibberellin-mediated growth processes and ultimately affect yield potential. Understanding the relative contributions of genetic background and environmental factors to gibberellin concentrations in maize tissue can inform breeding strategies and help develop more resilient short-stature varieties that maintain desired height characteristics across diverse growing conditions.

Understanding the natural range and stability of gibberellin levels in conventional hybrid maize across diverse environments may help contextualize future studies of gibberellin related traits changes in new hybrids and predicting their performance across different agricultural regions. The purpose of this study is to quantify GA_1_ and GA_4_ in maize by the validated LC-MS/MS method across conventional maize hybrids, tissue types, and environments, which could provide a baseline understanding of the contribution of genetics and environment to the variation of gibberellin content.

## Materials and methods

### Maize field trials

Plant materials consisted of conventional crop varieties grown in experimental plots that were collocated with USDA permitted materials under Authorization No. AUTH0000233243. Maize samples from ten commercially available conventional maize hybrids were collected from field trials during the 2023 growing season. Six field trial locations were spread across states representing major maize-growing regions of the United States (Iowa, Illinois, Indiana, Missouri, Nebraska, and Ohio) (Table 1).

**Table 1:** Field trial locations and maize hybrid names.

Site Code	County, State	Hybrid Name
Site 1	Jefferson County, Iowa	Dekalb DKC58−32
		Lewis 12CV779
		Channel 210−79
Site 2	Shelby County, Illinois	Stone 6290
		Hubner H2390
		Dekalb DKC65–92
Site 3	Boone County, Indiana	Dekalb DKC63−58
		Hubner H2390
Site 4	Adair County, Missouri	Dekalb DKC58−32
		Lewis 12CV779
		Stone 6070
Site 5	York County, Nebraska	Stone 6290
		Hubner H2390
		Dekalb DKC64−32
Site 6	Miami County, Ohio	Dekalb DKC58−32
		Stewart 12CV209
		Channel 210−79

Each site was planted in a randomized complete block design with four replicated blocks. Hybrids listed in Table 1 were included in each of the four replicated blocks where they were planted, in addition to other hybrids not associated with this study. Within a block, each hybrid was planted in an approximately 6 m × 6 m plot. Each site received a unique randomization scheme. Blocks at each site were arranged to limit variability in environmental conditions such as soil type, slope, and previous cropping history. Normal agronomic practices were followed for each site. Trial maintenance (fertilization, pesticide use, tillage practices, irrigation, etc.) varied across sites, but all maintenance activities were applied uniformly within a site.

Maize samples were collected throughout the growing season and included the following tissues: leaf, root, stalk, pollen, grain, and forage (cut corn plant just above the soil surface and retain all aboveground plant parts (e.g., leaves, ears, and stalk) as the forage sample). The same type of material collected from each plot was pooled into a single sample. Samples were non-systematically selected with consistent field sampling criteria: plot ends were avoided, and plants were chosen to be competitive and representative of the plot as a whole. The selected tissue samples from each plot were combined to generate one pooled sample per plot, which served as the observational unit for downstream analysis. This pooling approach was applied consistently across all sites. Sampling details can be found in S1 Table. Specifically, it specifies the sampling amount used to generate one pooled sample per lot: 60 leaf sections, 8 roots, 4 stalks, 10 mL pollen, 1 plant forage, or 1 kg grain, along with the corresponding number of sampled plants and growth stages. All samples were kept on dry ice immediately after collection until storage in a −80ºC freezer prior to analysis, with the exception of grain samples which were kept at ambient temperature.

### Reagents

Gibberellin standards were used as reference standards for the analytical procedures or calibration of equipment. Stable-isotope-labelled gibberellin standards were used as internal standards (synthesized by OlChemIm s.r.o.(OlChemIm s.r.o, Czech Republic; https://olchemim.cz)). GA_1_ was synthesized by Toronto Research Chemicals (purity: 97%) and GA_4_ was synthesized by Thermo Scientific (purity: 90.30%). Formic acid (purity: 99%), ethyl acetate (HPLC grade) and methanol (purity: ≥ 99.8%) were purchased from Fisher Scientific and acetic acid (analytical grade) was obtained from Millipore, AR.

### Sample preparation

After cryomilling maize tissues, 50 ± 5 mg of fresh frozen sample was weighed into a pre-chilled 1.5 mL glass tube placed on dry ice. Next, on wet ice, 10 µL of internal standard working solutions (100 ng/mL of GA_1_-D4 and 25 ng/mL of GA_4_-D2) and 1.00 mL of pre-chilled extraction buffer (80% methanol with 0.5% formic acid) were added to each sample. The samples, arranged in a 96-well formatted rack, were manually agitated 40 times to thoroughly suspend the tissue in the extraction buffer, followed by an overnight incubation (approximately 15 hours) at 4°C with gentle mixing at 50 rpm on a Fisher brand Mixer (Fisher Scientific, 88-861-043). After incubation, the samples were centrifuged at 2000 rpm for 10 minutes at 4°C. The supernatant was transferred to a 96-well polypropylene sample collection plate. The pellets were re-extracted by adding another 1 mL of extraction buffer following the same procedure as above. The combined supernatants evaporated under nitrogen flow at 35°C using a microplate sample evaporation system (Biotage SPE Dry 96, SD-9600-DHS-NA), until about 400 µL of aqueous phase was left. Subsequently, the extracts were loaded into a Biotage 96-well supported liquid extraction (SLE) plate (Biotage, 8200400P01), which was mounted on a glass-coated 96-well collection plate. The analyte gibberellins were eluted with 2 × 0.9 mL of ethyl acetate. The eluate was then evaporated to dryness under nitrogen by using the SPE Dry 96. The dried samples were reconstituted in 100 µL of reconstitution buffer (20% Methanol with 0.1% formic acid), then vortexed at 1200 rpm at room temperature for 3 minutes. Following this, the samples were centrifuged at 2000 rpm for 3 minutes at 4°C, and 90 µL of the supernatant was transferred into a 96-well plate with glass inserts, kept on wet ice prior to LC-MS/MS analysis.

**LC-MS/MS analysis of gibberellins** Analytical instruments include mass spectrometer (Sciex triple Quad 7500), HPLC system (ExionLC 20A system) and data acquisition system (PC-based workstation with AB Sciex OS software). Reconstituted samples were separated on a 1.7 µm Kinetex 100 Å C18 LC column (100 mm × 2.1 mm) (Phenomenex, 00D4475AN) at a flow rate of 0.40 mL/min and a column oven of 40˚C. Mobile phase A is 0.5% acetic acid in water and mobile phase B is 0.5% acetic acid in methanol. The LC gradient is presented in Table 2. Mass spectrometric conditions are as follows: polarity mode, negative ionization; scan type, MRM; spray voltage, −1800 V; ion source gas 1, 40 psi; ion source gas 2, 45 psi; source temperature, 500°C; curtain gas, 40; CAD gas, 10; entrance potential (EP), −10 V. The multiple reaction monitoring (MRM) transitions for each analyte and its stable isotope-labeled internal standard (IS) can be found in Table 3 with respective optimized collision energy (CE) and collision cell exit potential (CXP). The representative MRM chromatogram of GA1 and GA4 in the LLOQ sample (standard 1) is provided as Supporting Information S1 Fig.

**Table 2:** LC gradient.

Time (min)	Flow (mL/min)	B.Conc (%)
0	0.40	26
5.30	0.40	26
5.40	0.40	55
10.20	0.40	58
10.30	0.40	80
12.70	0.40	81
12.80	0.40	98
14.00	0.40	98
14.10	0.40	26
16.00	0.40	26

**Table 3:** MRM transition parameters.

Compound ID	Retention time (minutes)	Q1 mass (Da)	Q3 mass (Da)	Dwell (ms)	CE(V)	CXP(V)
GA_1_	4.63	347.19	273.10	100	−32	−8
GA_4_	9.37	331.19	213.13	100	−40	−7
GA_1_-D4 (IS)	4.48	351.19	277.07	100	−36	−9
GA_4_-D2 (IS)	9.34	333.14	259.06	100	−33	−9

### MS data analysis

Raw data files were analyzed using Analytics in SCIEX OS software. A standard curve for each gibberellin was generated as the ratio of the gibberellin standard’s response (peak area) to the stable-isotope-labelled standard response (peak area), plotted against the concentrations of the gibberellin standard. A linear regression model was used for the quantification of the gibberellin levels with 1/x^2^ weighting. The final concentration of gibberellins in the samples was reported in pmol/g.

### Variance component analysis to compare genetic and environmental variations

To compare genetic and environmental contributions to GA1 and GA4 expression across five tissues, we first examined the distribution of the response variables. Visual inspection of the observed response and residual plots from the analysis indicated a log‑normal distribution, supporting the use of log‑transformed data in subsequent analyses. Then, a variance component analysis [25,26] was performed with the following model (1) and a log transformation for the normality assumption of the analysis.


$$\begin{array}{c} y_{ijk}=\mu +s_{i}+r_{j(i)}+m_{k}+{\left(sm\right)}_{ik}+\varepsilon_{ijk} \end{array}$$ (1)

where *y*_*ijk*_ is the observed response of each plot after transformation for the *k*^*th*^ hybrid from the *j*^*th*^ replicate (i.e., block) at the *i*^*th*^ site, *µ* is the overall mean, *s*_*i*_ is the effect of the *i*^*th*^ site, *r*_*j(i)*_ is the effect of the *j*^*th*^ replicate within the *i*^*th*^ site, *m*_*k*_ is the effect of the *k*^*th*^ hybrid, *(sm)*_*ik*_ is the interaction effect of the *k*^th^ hybrid with the *i*^*th*^ site, *ε*_*ijk*_ is the residual. The term replicate and block will be used interchangeably in the following. Before fitting the model, Levene’s tests were conducted to examine the assumption of the variance homogeneity, and non-significance was found for effects of sites, materials, and interactions on the variance. Different SAS procedures were used to estimate variance components, test significance, and examine changes in hybrid means across sites, with each procedure chosen to leverage its specific analytical strengths. To estimate variance components, Hybrid, Site, Replicate within Site, and the Hybrid-by-Site interaction were all treated as random effects, with the residual term representing error variance. Random effects were assumed to be independently and identically distributed within each classification, each associated with its own variance component. Variance was partitioned into these additive components using SAS PROC MIXED with REML (Restricted Maximum Likelihood) estimation, a well-established approach for variance component estimation in unbalanced trial designs. The significance of variance components was then evaluated using PROC GLIMMIX with the COVTEST option via likelihood ratio tests. Because the null hypothesis places a variance component on the boundary of the parameter space at zero, the resulting test statistic follows a mixture chi-square distribution. Significance was evaluated at 5%, 1%, and 0.1% levels without multiplicity correction. For variance components that were found to be significant, additional analyses were performed to examine mean changes for individual hybrids across sites, thereby illustrating the extent to which hybrid effects were connected across sites and characterizing site-specific hybrid patterns. These analyses were conducted within a linear mixed model framework using PROC MIXED. Hybrid, Site, and the Hybrid-by-Site interaction were treated as fixed effects, whereas Replicate nested within Site was modeled as a random effect. Treating Hybrid and Site as fixed effects produced local LS-means for characterizing Hybrid-by-Site patterns, while modeling nested replications as random prevented estimates from being biased by local block-level anomalies. All analyses were performed separately for each gibberellin and tissue combination, and results were presented in the following sections.

For the assumptions of the analysis, in addition to the composition of the model (1) as described above, distributional assumptions were further examined by the residual plots from PROC MIXED which showed no evidence of violation of normality (after the log transformation on the original observed response) and variance heterogeneity (as an additional visual confirmation to the above Levene’s test). Overall, the use of SAS procedures with REML estimation was appropriate for variance component estimation, significance testing, and LS-means estimation, since these procedures are well suited to handling unbalanced data within a properly specified mixed-model framework.

## Results

### Method development and validation of gibberellins by LC-MS/MS

Plant extracts contain complex mixtures; typically, gibberellins are present in trace amounts. Therefore, it is necessary to apply both a consistent sample preparation method to reliably extract gibberellins from plants and a sensitive technique to accurately quantify them.

In this study, a robust, reliable, and high-throughput method was developed for the simultaneous quantitation of gibberellins in six maize tissue types (grain, pollen, leaf, root, forage, and stalk). In brief, the gibberellins were extracted from approximately 50 mg of sample, purified by SLE, and separated and quantified by LC-MS/MS using the isotope-labeled internal standard (IS) (Fig 1). The method was validated by determining the linearity (R^2^ ≥ 0.98) over the concentration range of 20–30,000 pg/mL in six tissue types. 20 pg/mL is the LLOQ (lower limit of quantification). Precision of the method was obtained with intra-and inter-day relative standard deviations (≤17.13%) and accuracy for compounds typically ranging between 79–124%. The recoveries of our method ranged from 77.6% to 119.8% (Table 4). The bench-top stability of the sample extraction supernatant was evaluated at room temperature for each type of tissue, demonstrating stability for a duration of up to two hours for all except pollen, which exhibited stability of only one hour. Additionally, the stability of the processed samples in the autosampler was assessed at a temperature of 4 °C, with all samples maintaining stability for up to six days.

**Table 4:** Accuracy, precision, extraction efficiency and recovery.

Sample type	Analytes	Accuracy (%)		Precision (CV%)		Extraction Efficiency (%)	Recovery (%)
		Intra-assay	Inter-assay	Intra-assay	Inter-assay		
Stalk	GA_1_	83.92-114.86	95.78-100.71	0.56-11.5	1.80-14.52	94.56	96.56
	GA_4_	87.69-113.10	90.62-105.81	0.42-11.69	2.51-9.33	91.52	115.72
Grain	GA_1_	79.73-115.95	89.38-106.18	0.49-12.39	1.65-17.13	95.91	92.01
	GA_4_	85.83-110.13	86.33-108.89	0.99-12.52	1.48-11.78	93.02	77.59
Leaf	GA_1_	89.03-115.97	98.55-107.07	0.32-11.38	2.21-15.88	93.67	82.59
	GA_4_	82.38-108.36	90.10-105.68	0.53-12.52	2.16-10.61	91.39	82.33
Root	GA_1_	83.92-114.86	95.78-104.13	0.24-11.50	1.80-14.52	96.51	98.52
	GA_4_	85.23-108.36	90.62-103.43	0.42-11.69	2.51-9.43	91.08	107.03
Forage	GA_1_	83.92-123.06	92.36-110.33	0.32-9.13	2.81-13.98	94.44	93.40
	GA_4_	87.69-108.95	90.62-103.43	0.42-11.69	2.51-9.33	91.71	101.89
Pollen	GA_1_	79.79-115.95	95.18-105.44	0.32-11.38	2.21-14.28	94.78	86.95
	GA_4_	81.63-110.13	88.31-108.89	0.99-12.52	1.48-11.78	91.70	76.89

**Fig 1:**
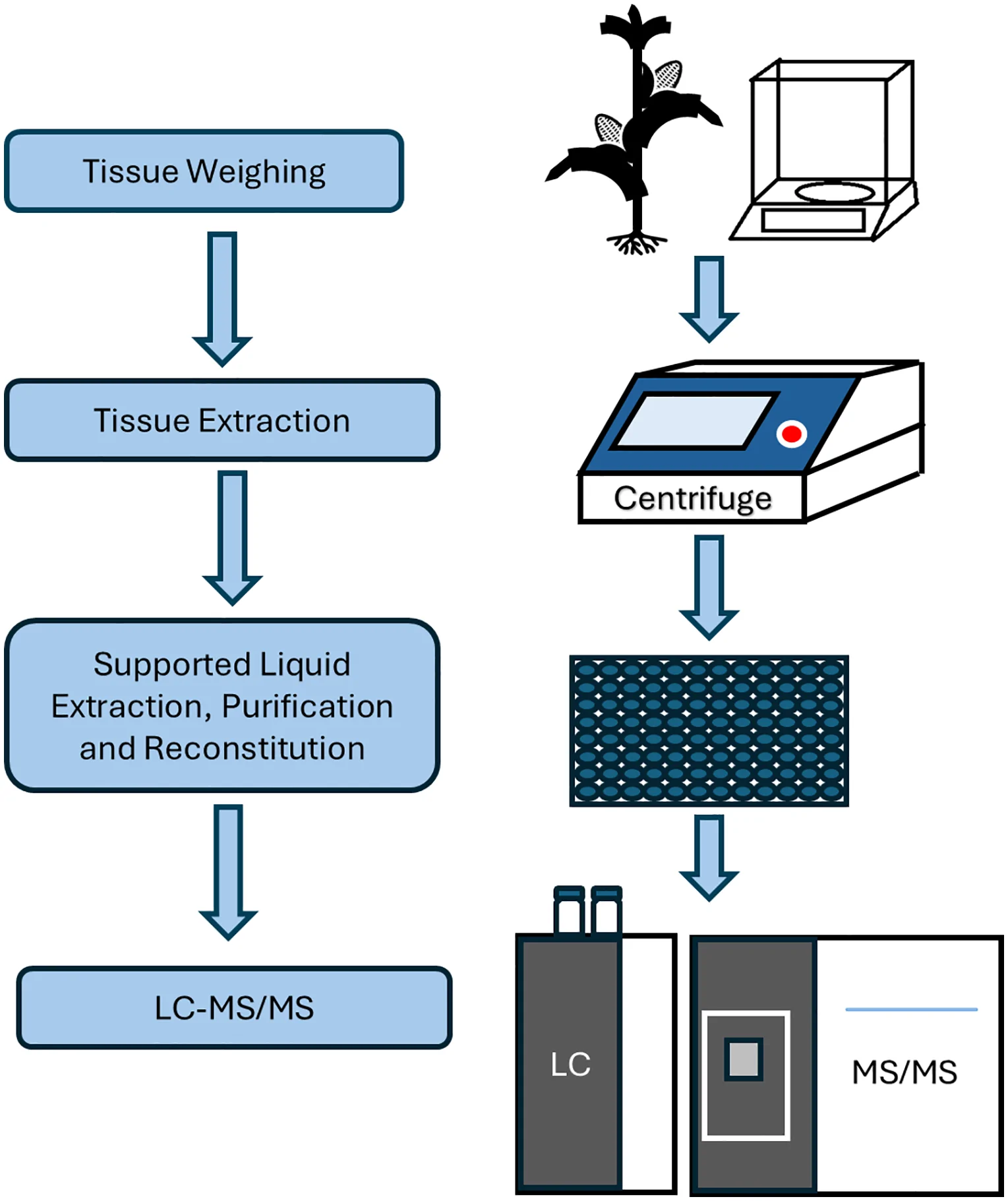
Workflow for sample preparation and analysis of gibberellins. Left Panel: The workflow consists of 4 key steps: (1) sample preparation via cryomilling and weighing, (2) extraction of gibberellins with internal standards (IS) and extraction buffer, (3) SLE analyte purification through centrifugation, supernatant collection, and reconstitution of the analyte, and (4) LC-MS/MS analysis for quantitation. Right Panel: Equipment and components used in the workflow include a balance for weighing samples, a centrifuge for supernatant separation, a SLE plate for sample purification, and an LC-MS/MS system for gibberellin quantification.

### Quantification of gibberellins in different maize tissue samples

The gibberellins in the samples were extracted, purified, separated and quantified by LC-MS/MS. The short stability window of pollen tissue limited its suitability for large‑scale sample handling; therefore, pollen data were not reported in this study. In total, 68 individual samples of each of the five other tissue types were analyzed and reported. Data with non-detection (ND) values were assigned values by replacing ND with half of the observed minimums for that tissue in order to apply the statistical analysis. The details regarding values given to ND samples are included in Table S2. Gibberellin concentrations in maize tissues were calculated based on the standard curve and the statistics are summarized in Table 5.

**Table 5:** Summary of GA_1_ and GA_4_ concentration.

Analyte	Sample	Mean (pmol/g)	Standard Error	Min (pmol/g)	Max (pmol/g)
**GA**_**1**_	Forage	7.9	2.61	0.4	131.1
**GA**_**1**_	Grain	1.4	0.43	0.1	26.0
**GA**_**1**_	Leaf	3.2	0.57	0.4	27.0
**GA**_**1**_	Root	0.5	0.04	0.1	1.3
**GA**_**1**_	Stalk	2.1	0.08	0.9	3.4
**GA**_**4**_	Forage	1.9	0.56	0.1	29.2
**GA**_**4**_	Grain	0.9	0.06	0.3	2.0
**GA**_**4**_	Leaf	0.3	0.03	0.1	1.4
**GA**_**4**_	Root	0.6	0.03	0.1	1.8
**GA**_**4**_	Stalk	0.4	0.02	0.2	0.9

### Variance components among five maize tissue samples

Variance components describe how different sources of variation contribute to observed differences in gibberellin concentrations. Estimates of variance components for five maize tissues samples are listed in Table 6. The model variance was calculated as the sum of all components except the residual.

**Table 6:** Estimated variance components in log scale and the statistical significance from each source.

Analyte	Samples	Variance (Model-log)	Variance Components (log)^1^				
			Varg	Vars	Vargxs	Varr	Vare
GA_1_	Forage	0.39	0.00	0.13	0.19	0.07	1.88
GA_1_	Grain	0.41	0.08	0.05	0.00	0.28	1.21
GA_1_	Leaf	1.29	0.09	1.09***	0.10	0.01	0.25
GA_1_	Root	0.11	0.04	0.02	0.00	0.05	0.67
GA_1_	Stalk	0.10	0.01	0.07*	0.03**	0.00	0.03
GA_4_	Forage	0.73	0.00	0.58*	0.00	0.14	1.09
GA_4_	Grain	0.19	0.06*	0.09*	0.01	0.02	0.07
GA_4_	Leaf	0.65	0.05	0.54**	0.07	0.00	0.47
GA_4_	Root	0.05	0.00	0.01	0.03	0.01	0.20
GA_4_	Stalk	0.05	0.00	0.00	0.05*	0.00	0.05

1 hybrid (Varg), site (Vars), hybrid by site interaction (Vargxs), replicate (Varr), and residual (Vare). Variance (Model) is the sum of all components except Vare.

* indicates statistical significance at the 5% level, ** for significance at the 1% level, and *** at the 0.1% level.

The relative contribution of variance components (i.e., the percentage of each source to total variance) is presented in Fig 2. As shown in Table 6 and Fig 2, the residual variation (Vare) is greater than the model-explained variation in five out of ten gibberellin–tissue combinations. Because the variance components in Table 6 and Fig 2 were estimated using an all-random-effects model, statistical significance should be interpreted as evidence that these sources of variation are likely to contribute under repeated sampling of similar sites and hybrids. While conducting 40 statistical tests increases the risk of false positives, effects supported by both substantial variance contributions and highly significant p-values (p < 0.01 and p < 0.001) provide strong evidence of meaningful structure in the data rather than being random noise. Concentrations of gibberellin A1 (GA_1_) and gibberellin A4 (GA_4_) (mean ± 1 SE) in leaf and stalk were measured and plotted for 10 maize hybrids across six field sites, which are presented in Fig 3. The standard errors are for the predicted mean of each site/hybrid combination from fitting the mixed-effect model.

**Fig 2:**
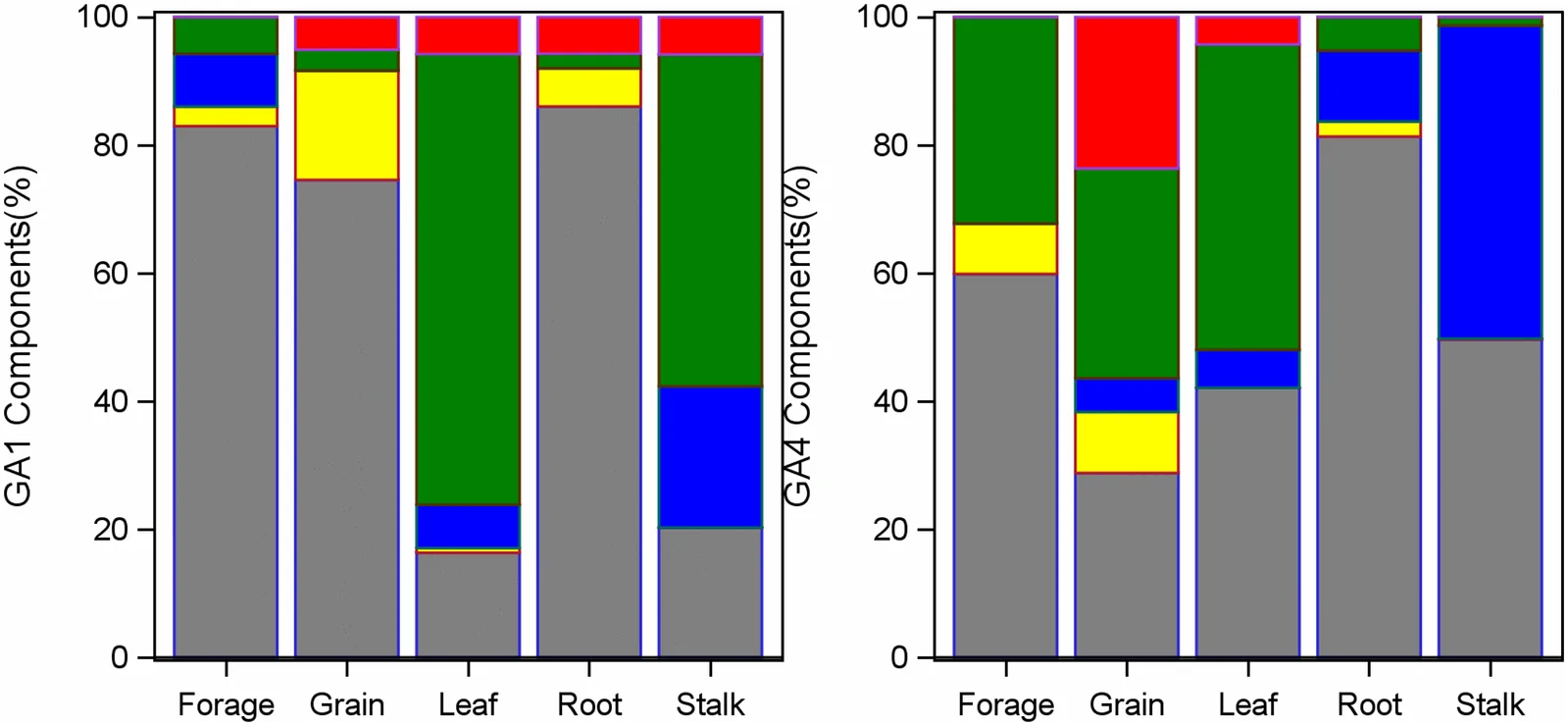
Variance component analysis of genetic and environmental contributions to GA_1_ and GA_4_ concentrations. The variance component analysis of gibberellin A1 (GA_1_) and gibberellin A4 (GA_4_) concentrations, broken down into contributing sources of variation. Bar charts show the percentage contributions of variance components: Varg (genetic variance, red), Vars (site variance, green), Vargxs (genetic-by-site interaction variance, blue), Varr (replicate variance, yellow), and Vare (residual variance, gray). (A) Variance components for GA_1_. (B) Variance components for GA_4_.

**Fig 3:**
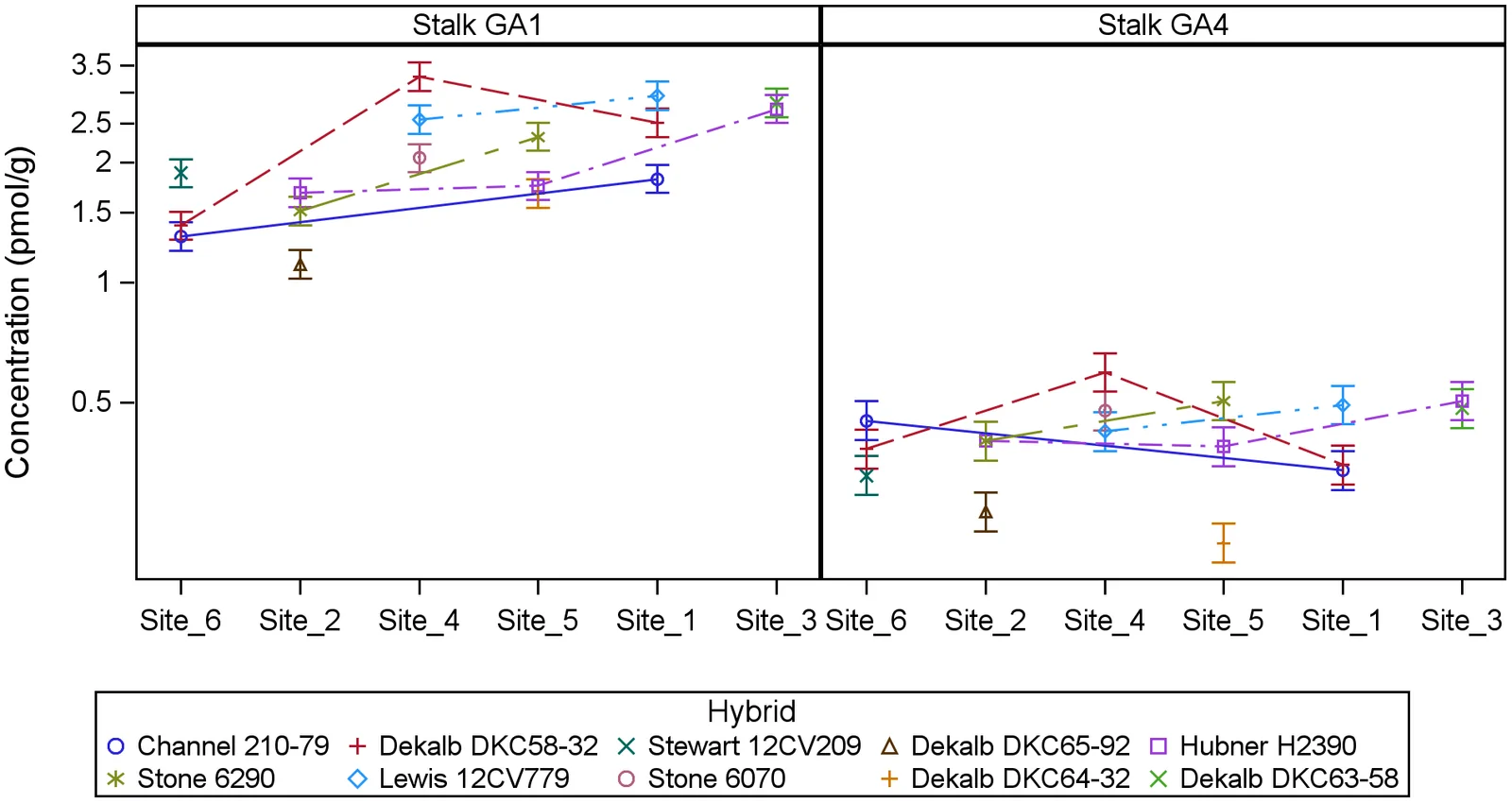
GA_1_ and GA_4_ concentrations in leaf and stalk across hybrids and sites. Concentrations of gibberellin A1 (GA_1_) and gibberellin A4 (GA_4_) (mean ± SE) in leaf and stalk were measured and plotted for 10 maize hybrids across six field sites. Data are presented in pmol/g, with hybrids represented by different markers and colors, and site-wise trends connected by lines for visualization **Panels:** Top Left: Leaf GA_1_ concentration, Top Right: Leaf GA_4_ concentration, Bottom Left: Stalk GA_1_ concentration, Bottom Right: Stalk GA_4_ concentration.

### Correlation between GA1 and GA4 across maize tissue samples

Biosynthetic pathways for GA1 and GA4 share a common upstream pathway, and the subsequent parallel branches utilize the same families of enzymes (e.g., GA20ox, GA3ox) [10]. From this perspective, the correlation between GA_1_ and GA_4_ was investigated using biplots. The plot is based on the hybrid mean at each site as shown in Fig 4. The analyses were conducted within a linear mixed model framework using PROC MIXED. Hybrid, Site, and the Hybrid-by-Site interaction were treated as fixed effects, whereas Replicate nested within Site was modeled as a random effect. In this plot, each mark represents the predicted hybrid mean over four replicates in log and then back transformed into pmol/g; five maize tissues are represented by different markers and colors. As shown in Fig 4, positive correlations were observed in four out of five tissues except root. The position and range of each tissue are represented by the differences in means (Table 5) and the variation (Table 6) among tissues. Since the hybrid mean at each site in Fig 4 consists of both the hybrid and site effects on the expressions, these positive correlations are due to similar trends in hybrid mean changes over sites between two gibberellins as shown in Fig 3. The regression coefficients of GA_4_ on GA_1_ in log were estimated as 0.01, 0.42, 0.42, 0.40, 0.49 for root, forage, leaf, grain, stalk with P values of 0.94, 0.10, 0.03, < 0.01, < 0.01, respectively, which are significant at the 5% level for leaf, grain, and stalk, and non-significance for forage. Notably, when examining residual variation after accounting for hybrid and site effects, positive correlations were observed with similar magnitude.

**Fig 4:**
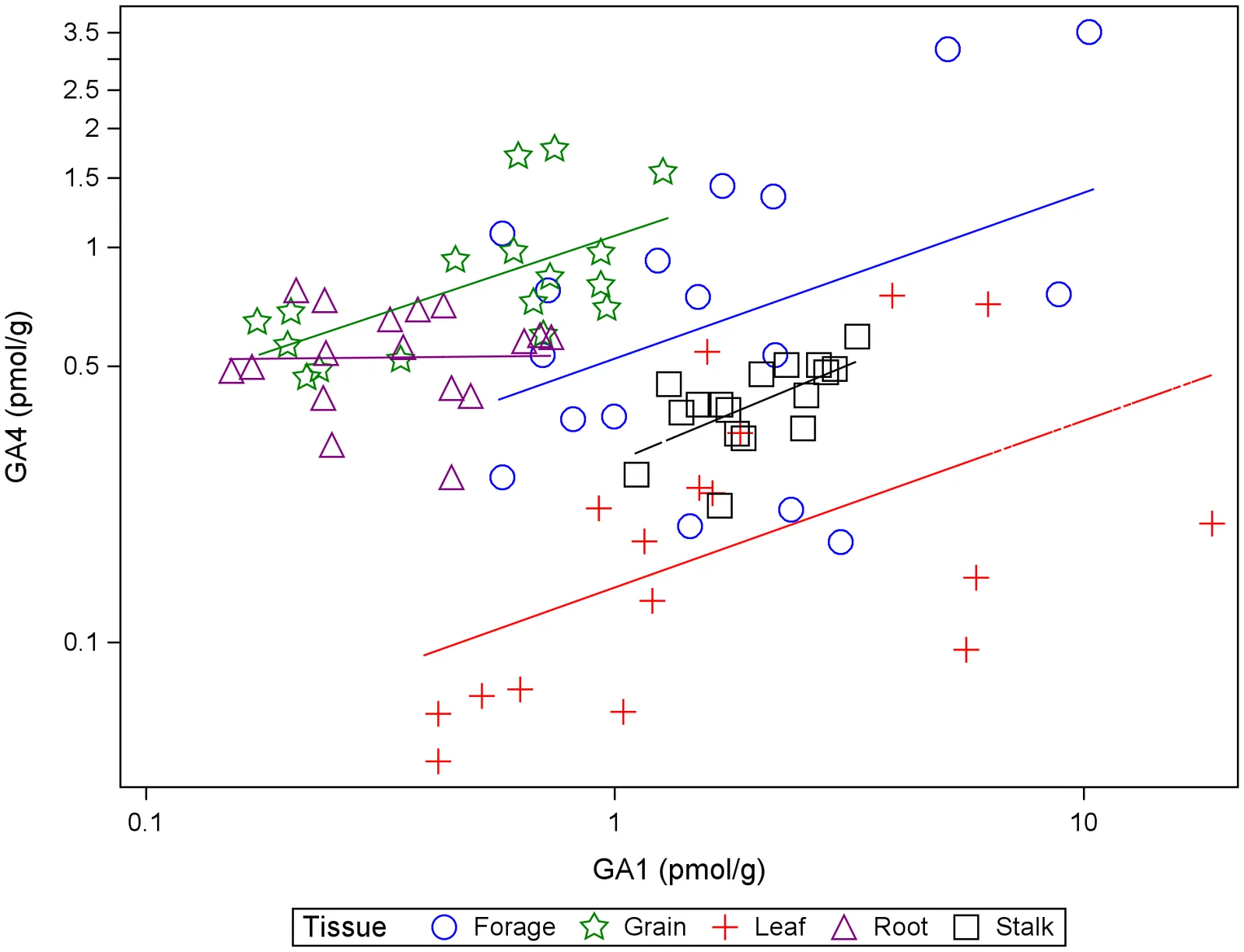
Biplot and correlation between GA_1_ and GA_4_ means across five maize tissues. The figure illustrates the relationship between gibberellin A1 (GA_1_) and gibberellin A4 (GA_4_) concentrations across five maize tissues: root, forage, leaf, grain, and stalk. Each marker in the biplot represents the predicted hybrid mean for GA_1_ and GA_4_ at a specific site, derived from four replicates, expressed in pmol/g (log-transformed and back-transformed). Different maize tissues are distinguished by unique markers and colors.

## Discussion

Quantitative analysis of gibberellins has advanced rapidly across diverse crops with the development of LC–MS/MS-based workflows [27], including methods for broad gibberellin profiling [28,29] and high-efficiency quantitation in complex plant matrices [20,30]. This study focuses on GA_1_ and GA_4_ quantification in commercial maize hybrids sampled across diverse environments, providing a baseline dataset for describing natural variation under standard agricultural practices. Among the five tissues, forage exhibited the highest active gibberellins concentrations and the broadest dynamic range, with GA_1_ ranging from 0.4 to 131.1 pmol/g and GA_4_ from 0.1 to 29.2 pmol/g. Published data for forage tissue is limited; while available data for shoot tissue can serve as a reference. GA_1_ levels in shoot ranged from 0.04 to 274.4 pmol/g [31, 32] andGA_4_ in the shoot tips ranged from 0.06 to 1.05 pmol/g [21,33].

Stalk showed a narrower and more stable range (GA_1_: 0.9–3.4 pmol/g; GA_4_: 0.2–0.9 pmol/g), aligning closely with literature values (GA_1_ ~ 1.8 pmol/g; GA_4_: 0.33–0.98 pmol/g) [19]. This consistency suggests that stalk may be a useful tissue for comparison in future studies.

Leaf in this study showed GA_1_ levels ranged from 0.4 to 27.0 pmol/g and GA_4_ from 0.1 to 1.4 pmol/g. The GA_1_ concentrations observed here are higher than literature-reported ranges of 0-1.8 pmol/g, possibly due to the limited number of samples reported previously, while GA_4_ levels fall within the broader literature range of 0–3.5 pmol/g [19,33,34]. The elevated GA_1_ levels may reflect differences in tissue sampling, developmental stage, genotype, field conditions, or study design. In contrast, the GA_4_ levels, although lower than the upper literature limit, are consistent with expected physiological ranges and suggest that GA4 levels in leaf tissue appear less variable than GA1 in this dataset.

Grain exhibited GA_1_ levels from 0.1 to 26.0 pmol/g and GA_4_ from 0.3 to 2.0 pmol/g, exceeding the ranges (GA_1_: 0.29–2.0 pmol/g; GA_4_: 0–1.2 pmol/g) reported in the literature [22,35]. This suggests that gibberellin levels in grain may span a broader range than previously reported. The tight range data in the literature may be reflecting differences in sample number, hybrid background, field site, developmental stage, or sampling design [35].

Root in this study showed GA_1_ concentrations ranging from 0.1 to 1.3 pmol/g and GA_4_ from 0.1 to 1.8 pmol/g. The relatively narrow range and lower mean values indicate that measured GA_1_ and GA_4_ levels in root were lower and less variable than in several above ground tissues in this dataset. These results support including below-ground tissues in future GA profiling studies to better describe tissue-specific GA abundance.

Overall, these comparisons highlight the broader dynamic range of bioactive GA_1_ and GA_4_ captured in this study, particularly forage, leaf, and grain tissues. The broader ranges observed in certain material types may reflect the larger number of samples, field-grown conditions, developmental differences, and the use of commercial hybrids compared with prior studies [31]. The comparison of literature data with our findings reinforces the robustness of the LC-MS/MS method and provides a reference for future studies of GA_1_ and GA_4_ abundance in maize tissues.

The contribution of different sources of variance components to measured GA_1_ and GA_4_ levels was analyzed using an all-random-effect model. Model (1) included the effects of site, hybrid, the hybrid × site interaction, replicate nested within site, and residual. The hybrid effect represents genetic variation, and the site effect represents environmental variation among locations. Replicate nested within site accounts for variation among field replicates within each location, whereas the residual captures the remaining unexplained variation, including plant sampling variation in the field and assay variation in the laboratory. The hybrid × site interaction was further examined for tissues showing evidence of interaction in the variance component analysis.

Results in Table 6 and Fig 2 indicated that while both genetic and environment are important factors, the site is a major contributor as compared with the genetic background to the gibberellin levels. In addition, in five out of ten (gibberellins by tissue) combinations, residual variations are greater than the model variation, especially for forage and root.

Results in Table 6, together with means in Table 5, demonstrate substantial differences in the distribution of GA_1_ and GA_4_ among the five maize tissues. For example, forage exhibited the highest mean concentrations as well as the largest residual variances for both GA_1_ and GA_4_, whereas stalk showed intermediate mean concentrations but the lowest residual variances. Despite this lower residual variability, stalk accounted for a higher proportion of total model-explained variation than forage, as shown in Fig 2.

Across the ten gibberellins-tissue combinations analyzed, a significant hybrid contribution was detected only for GA_4_ in grain (Varg), compared with five combinations exhibiting significant site effects (Vars). Overall, six of the ten combinations showed at least one significant variance component attributable to site, hybrid, or site × hybrid interaction (Vargxs). Notably, only root showed no significant variance components for either GA_1_ or GA_4_, while hybrid × site interactions were significant for both gibberellins in stalk.

Estimation and interpretation of variance components depend on satisfaction of the model’s normality assumptions. Following log transformation, the variance component estimates reported in Table 6 are presented on the log scale and should be interpreted in a relative sense, whereas mean concentrations and associated standard errors reported in Table 5 are expressed in absolute units (pmol/g).

The experimental design was constrained by hybrid adaptation and site capacity, resulting in different subsets of hybrids being planted at each site. Because commercial maize hybrids are bred for specific maturity zones and local growing conditions, it was not feasible to plant the same set of hybrids across the full range of environments represented in this study. This approach was necessary to achieve broad geographic and environmental coverage, despite resulting in an unbalanced hybrid–site design.

As a consequence of this design, estimation and statistical testing of variance components, especially hybrid × site interactions, are based on reduced degrees of freedom. For example, although the experiment included ten hybrids and six sites, a fully balanced design would have yielded 45 degrees of freedom for the interaction term, whereas the mixed-model analysis of the available data provided only three. This imbalance limits the precision of variance component estimates and reduces statistical power for significance testing.

Despite these limitations, the unbalanced design does not preclude qualitative interpretation of the relative contributions of genetic, environmental, and interaction effects. The variance component analysis was conducted using an all-random-effects mixed model, and significance testing was performed using likelihood-based methods, which are appropriate for unbalanced data. While quantitative estimates should therefore be interpreted cautiously, trends comparing the relative importance of site, hybrid, and hybrid × site effects remain informative.

As shown in Fig. 3, gibberellin concentration profiles for many genetic backgrounds are largely parallel across sites, suggesting consistent environmental responses. However, some hybrids exhibit clear site-dependent variation, indicating that both genetic and environmental factors contribute meaningfully to gibberellin accumulation. For example, the hybrid Dekalb DKC58−32 showed the highest gibberellin concentrations at site 4 but relatively lower concentrations at other sites, illustrating environment-dependent expression patterns. Although the specific environmental drivers underlying these differences cannot be resolved in the present study, factors such as temperature, humidity, and rainfall are likely contributors, and further targeted experimentation will be required to assess their relative importance.

In our field dataset, GA_1_ and GA_4_ were positively correlated in forage, grain, leaf, and stalk, but not in root. Because these data were collected under standard agronomic conditions, the observed correlations could also reflect a shared response to overall plant physiological status (e.g., more vigorous growth) and to environmentally driven differences in growing conditions among sites, consistent with the strong site effects detected in the variance component analysis. Notably, the GA_1_–GA_4_ association also persisted when examining residual variation after accounting for hybrid, site, and hybrid × site effects.

## Conclusions

In this study, we applied a validated high-throughput LC-MS/MS method to simultaneously quantify the bioactive gibberellins GA_1_ and GA_4_ across multiple maize tissues of commercial maize hybrids grown in North American field environments. This work provides a field-based baseline dataset describing GA_1_ and GA_4_ variation under standard agricultural practices. We found that gibberellin concentrations varied significantly across the sampled material tissues, with forage exhibiting the broadest dynamic range and highest levels, while stalk tissue showed narrower concentration ranges, which may make it useful for comparison in future studies.

Variance component analysis indicated that site-associated variation contributed to measured GA_1_ and GA_4_ levels in several tissue-by-analyte combinations. We identified a significant genetic (hybrid) contribution to GA_4_ variance specifically in grain, which suggests that this tissue-analyte combination may warrant further study in larger, targeted experiments.

While this study provides a foundational dataset, it is important to note that it is based on a single growing season and limited to bioactive GA_1_ and GA_4_. Future multi-year studies and additional GA metabolites will be necessary to confirm these trends and further explore the impact of annual weather variations. We hope that the data and analyses presented here provide useful insights into the complex interplay between genetics and environment, in maize.

## Supporting information

S1 Table Summary of sample type and collection information. (DOCX)

S2 Table Value assigned to non-detection (ND) data points for statistical analysis. (DOCX)

S1 Fig The representative MRM chromatogram for GA1 and GA4 in the LLOQ (standard 1). (PDF)

S1 Appendix Raw dataset. (XLSX)

S2 Appendix Validation data. (XLSX)

## References

[1] Kurosawa E. Experimental studies on the nature of the substance secreted by the “bakanae” fungus. Nat Hist Soc Formosa. 1926;16:213–27.

[2] Castro-Camba R, Sánchez C, Vidal N, Vielba JM. Plant development and crop yield: the role of gibberellins. Plants (Basel). 2022;11(19):2650. doi: 10.3390/plants11192650 36235516 PMC9571322

[3] Yamaguchi S. Gibberellin metabolism and its regulation. Annu Rev Plant Biol. 2008;59:225–51. doi: 10.1146/annurev.arplant.59.032607.092804 18173378

[4] Ubeda-Tomás S, Federici F, Casimiro I, Beemster GTS, Bhalerao R, Swarup R, et al. Gibberellin signaling in the endodermis controls Arabidopsis root meristem size. Curr Biol. 2009;19(14):1194–9. doi: 10.1016/j.cub.2009.06.023 19576770

[5] Verma V, Ravindran P, Kumar PP. Plant hormone-mediated regulation of stress responses. BMC Plant Biol. 2016;16:86. doi: 10.1186/s12870-016-0771-y 27079791 PMC4831116

[6] Illouz-Eliaz N, Ramon U, Shohat H, Blum S, Livne S, Mendelson D, et al. Multiple Gibberellin Receptors Contribute to Phenotypic Stability under Changing Environments. Plant Cell. 2019;31(7):1506–19. doi: 10.1105/tpc.19.00235 31076539 PMC6635849

[7] Rizza A, Jones AM. The makings of a gradient: spatiotemporal distribution of gibberellins in plant development. Curr Opin Plant Biol. 2019;47:9–15. doi: 10.1016/j.pbi.2018.08.001 30173065 PMC6414749

[8] Nelissen H, Rymen B, Jikumaru Y, Demuynck K, Van Lijsebettens M, Kamiya Y, et al. A local maximum in gibberellin levels regulates maize leaf growth by spatial control of cell division. Curr Biol. 2012;22(13):1183–7. doi: 10.1016/j.cub.2012.04.065 22683264

[9] Hedden P. The current status of research on gibberellin biosynthesis. Plant Cell Physiol. 2020;61(11):1832–49. doi: 10.1093/pcp/pcaa092 32652020 PMC7758035

[10] Salazar-Cerezo S, Martínez-Montiel N, García-Sánchez J, Pérez-Y-Terrón R, Martínez-Contreras RD. Gibberellin biosynthesis and metabolism: A convergent route for plants, fungi and bacteria. Microbiol Res. 2018;208:85–98. doi: 10.1016/j.micres.2018.01.010 29551215

[11] Ilyas M, Nisar M, Khan N, Hazrat A, Khan AH, Hayat K, et al. Drought tolerance strategies in plants: A mechanistic approach. J Plant Growth Regul. 2020;40(3):926–44. doi: 10.1007/s00344-020-10174-5

[12] Emongor VE. Gibberellic Acid (GA3) Influence on Vegetative Growth, Nodulation and Yield of Cowpea (Vigna unguiculata (L.) Walp. J Agronomy. 2007;6(4):509–17. doi: 10.3923/ja.2007.509.517

[13] Shani E, Hedden P, Sun T-P. Highlights in gibberellin research: A tale of the dwarf and the slender. Plant Physiol. 2024;195(1):111–34. doi: 10.1093/plphys/kiae044 38290048 PMC11060689

[14] Rizza A, Walia A, Lanquar V, Frommer WB, Jones AM. In vivo gibberellin gradients visualized in rapidly elongating tissues. Nat Plants. 2017;3(10):803–13. doi: 10.1038/s41477-017-0021-9 28970478

[15] Binenbaum J, Weinstain R, Shani E. Gibberellin localization and transport in plants. Trends in Plant Sci. 2018;23(5):410–21. doi: 10.1016/j.tplants.2018.02.005 29530380

[16] Wu K, Xu H, Gao X, Fu X. New insights into gibberellin signaling in regulating plant growth-metabolic coordination. Curr Opin Plant Biol. 2021;63:102074. doi: 10.1016/j.pbi.2021.102074 34217918

[17] Erenstein O, Jaleta M, Sonder K, Mottaleb K, Prasanna BM. Global maize production, consumption and trade: Trends and R&D implications. Food Security. 2022;14(5):1295–319. doi: 10.1007/s12571-022-01288-7

[18] Stokstad E. High hopes for short corn. Science. 2023;382(6669):364–7. doi: 10.1126/science.adl5302 37883569

[19] Paciorek T, Chiapelli BJ, Wang JY, Paciorek M, Yang H, Sant A, et al. Targeted suppression of gibberellin biosynthetic genes ZmGA20ox3 and ZmGA20ox5 produces a short stature maize ideotype. Plant Biotechnol J. 2022;20(6):1140–53. doi: 10.1111/pbi.13797 35244326 PMC9129074

[20] Su X, Zheng J, Hao Y, Xia R, Xu J. A high-efficiency method for simultaneous quantitation of bioactive gibberellins in Litchi chinensis using UHPLC-QQQ-MS/MS. Scientia Horticulturae. 2024.

[21] Zhang J, Zhang X, Chen R, Yang L, Fan K, Liu Y, et al. Generation of transgene-free semidwarf maize plants by gene Editing of Gibberellin-Oxidase20-3 Using CRISPR/Cas9. Front Plant Sci. 2020;11:1048. doi: 10.3389/fpls.2020.01048 32742269 PMC7365143

[22] Murofushi N, Honda I, Hirasawa R, Yamaguchi I, Takahashi N, Phinney BO. Gibberellins from the Seed, Tassel, Cob and Silk of Maize. Agricultural and Biological Chemistry. 1991;55(2):435–9. doi: 10.1080/00021369.1991.10870589

[23] Yamaguchi I, Nakazawa HK, Nakagawa R, Suzuki Y, Kurogochi S, Murofushi N. Identification and semi-quantification of gibberellins from the pollen and anthers of Zea mays by immunoassay and GC/MS. Plant and Cell Physiology. 1990;31:1063–9.

[24] Murofushi N, Honda I, Hirasawa R, Yamaguchi I, Takahashi N, Phinney BO. Gibberellins from the tassel, cob, ‘seed,’ silk, and pollen of maize. New York, NY: Springer New York. 1991.

[25] Searle SRC, McCulloch CE. Variance Components. John Wiley & Sons, Inc. 2006.

[26] Sahai HAM. The Analysis of Variance Birkhäuser. Boston, MA; 2012.

[27] Pan C, Tan SN, Yong JWH, Ge L. Progress and development of analytical methods for gibberellins. J Sep Sci. 2017;40(1):346–60. doi: 10.1002/jssc.201600857 27717135

[28] Urbanová T, Tarkowská D, Novák O, Hedden P, Strnad M. Analysis of gibberellins as free acids by ultra performance liquid chromatography-tandem mass spectrometry. Talanta. 2013;112:85–94. doi: 10.1016/j.talanta.2013.03.068 23708542

[29] López-Cristoffanini C, Serrat X, Jáuregui O, Nogués S, López-Carbonell M. Phytohormone profiling method for rice: Effects of GA20ox mutation on the gibberellin content of Japonica rice varieties. Frontiers in Plant Science. 2019. doi: 10.3389/fpls.2019.00733 PMC6565999 31231411

[30] Fernández H, Doumas P, Bonnet-Masimbert M. Quantification of GA1, GA3, GA4, GA7, GA8, GA9, GA19 and GA20; and GA20 metabolism in dormant and non-dormant beechnuts. Plant Growth Regulation. 1997;22(1):29–35. doi: 10.1023/a:1005814926549

[31] Rood SB, Buzzell RI, Mander LN, Pearce D, Pharis RP. Gibberellins: a phytohormonal basis for heterosis in maize. Science. 1988;241(4870):1216–8. doi: 10.1126/science.241.4870.1216 17740785

[32] Fujioka S, Yamane H, Spray CR, Katsumi M, Phinney BO, Gaskin P, et al. The dominant non-gibberellin-responding dwarf mutant (D8) of maize accumulates native gibberellins. Proc Natl Acad Sci U S A. 1988;85(23):9031–5. doi: 10.1073/pnas.85.23.9031 16594001 PMC282656

[33] Liu Y, Chen Z, Zhang C, Guo J, Liu Q, Yin Y, et al. Gene editing of ZmGA20ox3 improves plant architecture and drought tolerance in maize. Plant Cell Rep. 2023;43(1):18. doi: 10.1007/s00299-023-03090-x 38148416

[34] Chen Z, Liu Y, Yin Y, Liu Q, Li N, Liu X, et al. Development of dwarfish and yield-effective GM maize through passivation of bioactive gibberellin. Transgenic Res. 2019;28(5–6):589–99. doi: 10.1007/s11248-019-00172-z 31595387

[35] White CN, Proebsting WM, Hedden P, Rivin CJ. Gibberellins and seed development in maize. I. Evidence that gibberellin/abscisic acid balance governs germination versus maturation pathways. Plant Physiol. 2000;122(4):1081–8. doi: 10.1104/pp.122.4.1081 10759503 PMC58942

