# Supplementary material for: Natural variation of bioactive gibberellin levels in maize tissues across North America
Source: PLoS One. 2026 Oct 7;21(10):e0340156. doi: 10.1371/journal.pone.0340156 (PMC13645078; doi:10.1371/journal.pone.0340156)

S1 Fig. The representative chromatogram of LLOQ (standard 1)

Top left: TIC, Top right: XIC of GA1, Bottom left: XIC of GA1-D4 and GA4-D2, Bottom right: XIC of GA4.

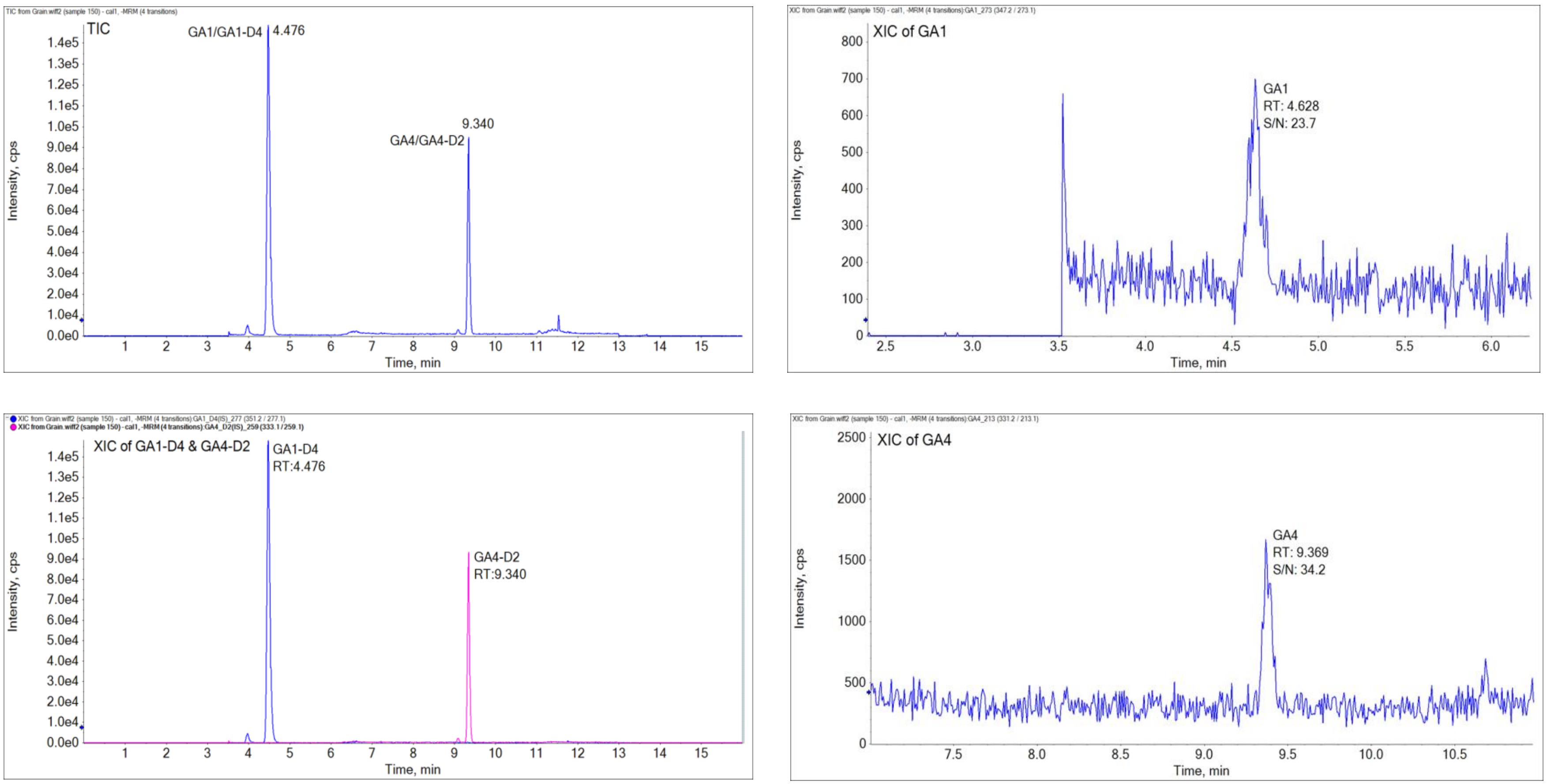

Supplement: Supplementary file 1 [file pone.0340156.s003.pdf]
