# Supplementary material for: Natural variation of bioactive gibberellin levels in maize tissues across North America
Source: PLoS One. 2026 Oct 7;21(10):e0340156. doi: 10.1371/journal.pone.0340156 (PMC13645078; doi:10.1371/journal.pone.0340156)
Supplement: Supplementary file 2 [file pone.0340156.s008.pdf]

PLOS One

Natural variation of bioactive gibberellin levels in maize tissues across North America

--Manuscript Draft--

**Manuscript Number:**	PONE-D-25-66749R2
**Article Type:**	Research Article
**Full Title:**	Natural variation of bioactive gibberellin levels in maize tissues across North America
**Short Title:**	Natural variation of gibberellin levels in maize tissues
**Corresponding Author:**	Yanfei Wang<br>Bayer Crop Science United States: Bayer CropScience LP<br>Chesterfield, UNITED STATES OF AMERICA
**Keywords:**	Gibberellin A1 (GA1); Gibberellin A4 (GA4); Liquid chromatography-tandem mass spectrometry (LC-MS/MS); natural variation; conventional maize tissues; stalk; leaf
**Abstract:**	Gibberellins (GA) are endogenous phytohormones that regulate growth and development throughout the plant lifecycle. While gibberellins have been extensively studied under controlled conditions for their fundamental role in plant adaptation and agronomic trait development, field-based data on their natural variation of bioactive GA levels across diverse agricultural genotypes and environments remains limited. To address this knowledge gap, we analyzed bioactive gibberellin A1 (GA1) and gibberellin A4 (GA4) in multiple tissues from commercial conventional maize hybrids grown in diverse field environments using high-throughput liquid chromatography-tandem mass spectrometry (LC-MS/MS). This study provides quantitative measurements of endogenous GA1 and GA4 levels in maize tissues collected under standard agricultural practices and describes variation influenced by genetic background and environmental conditions. Our findings demonstrate that GA1 and GA4 concentrations from conventional maize samples varied across tissue types, hybrid, and field sites. Analysis of variance components indicated that the site variation was statistically significant in five of ten gibberellins by tissue combinations, while the genetic component was significant only for GA4 in grain samples, suggesting that environment factors have a larger effect than genetic factors Positive correlations between GA1 and GA4 were observed consistently across tissues except root. These findings offer preliminary insights into the interplay between genetics and environment across germplasm and environments that have been previously described.
**Order of Authors:**	Yanfei Wang
	Xia Guan
	Nitin Supekar
	Changjian Jiang
	Junhong Guo
	Edwards Allen
	Charles Dietrich
	Cody Postin
	Shubha Shakya
**Opposed Reviewers:**	
**Response to Reviewers:**	Response to Reviewers<br>PONE-D-25-66749R2<br>Natural variation of bioactive gibberellin levels in maize tissues across North America<br>PLOS One<br><br>Dear Dr. Wang,<br>Thank you for submitting your manuscript to PLOS ONE. After careful consideration, we feel that it has merit but does not fully meet PLOS ONE's publication criteria as it currently stands. Therefore, we invite you to submit a revised version of the manuscript

that addresses the points raised during the review process.

Response
Dear Editor,
Thank you very much for your letter and for the careful evaluation of our manuscript. We sincerely appreciate your comments and the reviewers' constructive suggestions, which have been very helpful in improving the quality and clarity of our work.
We have carefully addressed the comments from both the editor and the reviewers on a point-by-point basis. In response to these comments, we have revised the manuscript accordingly and made the necessary changes throughout the text.
In our revised submission, we have included the following files as requested:
1. Response to Reviewers – a separate letter responding to each point raised by the academic editor and reviewers.
2. Revised Manuscript with Track Changes – a marked-up version highlighting all changes made to the manuscript PONE-D-25-66749R1.
3. Manuscript – a clean, unmarked version of the revised paper without tracked changes.
Thank you again for your time and consideration. We are grateful for the opportunity to revise our manuscript for further consideration by PLOS ONE.

You have improved the paper notably. Nevertheless problems remain. Reviewer 1, a statistician, points out fairly serious inadequacies remain in your presentation. Insofar as statistical analysis constitutes a big chunk of your paper, I believe that you need to get this right. Hopefully, the problems identified by the reviewer are matters of language (what you write about the methods). But in any case, I hope you can respond satisfactorily.

Response:
Thank you for highlighting this important concern. We appreciate the editor's emphasis on the need for a clear and rigorous presentation of the statistical analysis, particularly given its central role in the manuscript. In response, we carefully re-examined the statistical methods and substantially revised the "Variance component analysis to compare genetic and environmental variations" section to improve clarity, precision, transparency, and consistency.
We thoroughly revised the methodology section to clarify our statistical approaches, with emphasis on model assumptions, structure, and limitations. We explicitly defined our variance-partitioning framework, detailing how hybrid, site, replicate within site, and the hybrid-by-site interaction were treated across different stages of the analysis, specifically for estimating variance components, testing their significance, and examining changes in hybrid means across sites. To address the reviewer's concerns, we clarified that our use of REML (Restricted Maximum Likelihood) estimation is a well-established approach for variance component estimation in unbalanced trial designs. We also explained our rationale for not applying multiple-testing adjustments during significance testing of these components, and why confidence intervals for variance components were not provided. In addition, we removed references to SAS PROC GLM, which had been used only for intermediate data exploration, so that the manuscript now reflects only the analyses underlying the reported results. Overall, these revisions present the statistical methodology with greater transparency, stronger theoretical justification, and more appropriate caution regarding the scope and unbalanced nature of the available data.

Reviewer three (the original reviewer 2 was not available) points out several small but useful things. Their main point tho is about interpretation. Writing for PLoS One differs from many other journals because "importance" does not matter. For most journals, we write to convince the editor, the reviewers, and indeed readers, that our work is important. But importance is subjective. PLoS One removes that criteron, explicitly. Instead, what matters is validity. Writing on that head relieves us of the burden of hype. We present what we found, a straightforward account. We can of course state why we think the work matters but in doing so we should be brief and circumspect. Reviewer 3 points out where you need to town down your claims. I would add that you can probably shorten the discussion; compared to the results, it seems quite long.

Response:
Thank you for the great comments. The revised Discussion now focuses more directly

on the validity of the findings, the limitations of the study design and measurements, and a more circumspect interpretation of the implications.
We revised the Discussion to align more closely with the data presented in the Results. Specifically, we removed or rephrased language that could be interpreted as overly broad or mechanistic, including statements related to “biosynthesis” and “plasticity of the gibberellin pathway,”. We also shortened the Discussion by removing repetitive statements and reducing broader contextual language.

Small points: When you cite references in the text, please put a space between the word and the bracket. Like this [14]. Not like this[14]. Also on line 74, the word 'corn' should not be capitalized.

Response:
Thank you for pointing these out. We have made these corrections throughout the manuscript, including adding a space before citation brackets where needed and changing “Corn” to “corn” on previous line 74.

Please submit your revised manuscript by Aug 05 2026 11:59PM. If you will need more time than this to complete your revisions, please reply to this message or contact the journal office at plosone@plos.org. When you're ready to submit your revision, log on to https://www.editorialmanager.com/pone/ and select the 'Submissions Needing Revision' folder to locate your manuscript file.

Please include the following items when submitting your revised manuscript:
•A letter that responds to each point raised by the academic editor and reviewer(s). You should upload this letter as a separate file labeled 'Response to Reviewers'.
•A marked-up copy of your manuscript that highlights changes made to the original version. You should upload this as a separate file labeled 'Revised Manuscript with Track Changes'.
•An unmarked version of your revised paper without tracked changes. You should upload this as a separate file labeled 'Manuscript'.
If you would like to make changes to your financial disclosure, please include your updated statement in your cover letter. Guidelines for resubmitting your figure files are available below the reviewer comments at the end of this letter.

If applicable, we recommend that you deposit your laboratory protocols in protocols.io to enhance the reproducibility of your results. Protocols.io assigns your protocol its own identifier (DOI) so that it can be cited independently in the future. For instructions see: https://journals.plos.org/plosone/s/submission-guidelines#loc-laboratory-protocols. Additionally, PLOS ONE offers an option for publishing peer-reviewed Lab Protocol articles, which describe protocols hosted on protocols.io. Read more information on sharing protocols at https://plos.org/protocols?utm_medium=editorial-email&utm_source=authorletters&utm_campaign=protocols.
As the corresponding author, your ORCID iD is verified in the submission system and will appear in the published article. PLOS supports the use of ORCID, and we encourage all coauthors to register for an ORCID iD and use it as well. Please encourage your coauthors to verify their ORCID iD within the submission system before final acceptance, as unverified ORCID iDs will not appear in the published article. Only the individual author can complete the verification step; PLOS staff cannot verify ORCID iDs on behalf of authors.

We look forward to receiving your revised manuscript.
Kind regards,
Tobias Isaac Baskin
Academic Editor
PLOS One

Journal Requirements:
If the reviewer comments include a recommendation to cite specific previously published works, please review and evaluate these publications to determine whether they are relevant and should be cited. There is no requirement to cite these works unless the editor has indicated otherwise.

[Note: HTML markup is below. Please do not edit.]

Reviewers' comments:
Reviewer's Responses to Questions
Comments to the Author
1. If the authors have adequately addressed your comments raised in a previous round of review and you feel that this manuscript is now acceptable for publication, you may indicate that here to bypass the "Comments to the Author" section, enter your conflict of interest statement in the "Confidential to Editor" section, and submit your "Accept" recommendation.
Reviewer #1: (No Response)
Reviewer #3: (No Response)

---

2. Is the manuscript technically sound, and do the data support the conclusions?

The manuscript must describe a technically sound piece of scientific research with data that supports the conclusions. Experiments must have been conducted rigorously, with appropriate controls, replication, and sample sizes. The conclusions must be drawn appropriately based on the data presented.
Reviewer #1: Partly
Reviewer #3: Partly

---

3. Has the statistical analysis been performed appropriately and rigorously?
Reviewer #1: No
Reviewer #3: Yes

---

4. Have the authors made all data underlying the findings in their manuscript fully available?

The PLOS Data policy requires authors to make all data underlying the findings described in their manuscript fully available without restriction, with rare exception (please refer to the Data Availability Statement in the manuscript PDF file). The data should be provided as part of the manuscript or its supporting information, or deposited to a public repository. For example, in addition to summary statistics, the data points behind means, medians and variance measures should be available. If there are restrictions on publicly sharing data—e.g. participant privacy or use of data from a third party—those must be specified.
Reviewer #1: Yes
Reviewer #3: Yes

---

5. Is the manuscript presented in an intelligible fashion and written in standard English?

PLOS ONE does not copyedit accepted manuscripts, so the language in submitted articles must be clear, correct, and unambiguous. Any typographical or grammatical errors should be corrected at revision, so please note any specific errors here.
Reviewer #1: Yes
Reviewer #3: Yes

---

6. Review Comments to the Author

Please use the space provided to explain your answers to the questions above. You may also include additional comments for the author, including concerns about dual publication, research ethics, or publication ethics. (Please upload your review as an attachment if it exceeds 20,000 characters)
Reviewer #1: Experiments (blocking structure, randomisation, etc) are now much more clearly explained in the Methods. Though l130-132 "Maize samples were collected throughout the growing season and included the following tissues: leaf, root, stalk, pollen, forage, and grain. The same tissues collected from each plot were pooled into a single sample." remains somewhat ambiguous. How much of each tissue (and was this by weight, or volume, or something else)? From how many plants, and how were they selected (random, non-edge, convenience)? How pooled? Was pooling consistent across all sites?

Response:

Thank you for the comments. We have revised the Methods section to clarify sufficient details regarding the amount of tissue collected, the number of plants sampled, the selection criteria, and the pooling procedure. For each plot, sampled plants were selected non-systematically but with consistent field-sampling criteria: plot ends were avoided, and plants were chosen to be competitive and representative of the plot as a whole. The selected tissue samples from each plot were combined to generate one pooled sample per plot, which served as the observational unit for downstream analysis. This pooling approach was applied consistently across all sites.
We have added detailed sampling information in S1 Table, including the pooled sample amount per plot, the number of plants sampled per plot, tissue-specific sampling details, and the collection growth stage. Specifically, the revised table now specifies the tissue-specific sampling amount used to generate one pooled sample per lot: 60 leaf sections, 8 roots, 4 stalks, 10 mL pollen, 1 forage plant, or 1 kg grain, depending on tissue type, along with the corresponding number of sampled plants and growth stages.
S1 Table. Summary of sample type and collection information
Sample TypePooled Sample Amount per PlotNumber of Plants Sampled per PlotSampling DetailsCollection Growth Stage Growth Stage Description
Leaf   60 leaves60Approximately 15 cm of most recently collared leafV2-V4 A plant with 2 to 4 leaf collars
Root 8 roots8All below-ground root tissue, thoroughly washedV2-V4 A plant with 2 to 4 leaf collars
Stalk   4 stalks4All above-ground stalk tissue with leaf blade, leaf sheath, ear, and tassel removedV10-V12 A plant with 10 to 12 leaf collars
Pollen   10 mLHighly variable based on environmental conditions, though typically >10Fresh pollen with all anthers removedPollen shed A plant that is actively shedding pollen
Forage   1 plant1All above-ground plant tissueR5, early dent Most kernels are dented and contain approximately 55% moisture
Grain   1 kgMinimum of 4Shelled grain dried to ≤15.5% moisture prior to shipping and storageR6 Physiological maturity; black layer visible at base of kernels

Whilst the Methods section explains the experiment better, there's been no attempt made to connect it to the subsequent model, eqn (1). Does "block" (in intro) = "replicate" (in the model)? Does "plot" (in the intro) = the observational unit (in the model)? Does "pooling" (in intro) mean there is one observation per plot? The disconnect is a bit jarring, but could easily be fixed.
Response: Thank you for the suggestion. In the revised manuscript, we clarified that “block” in the experimental design corresponds to “replicate” in the statistical model, and that “plot” is the observational unit used in the model. We also clarified that the tissue samples collected within each plot were pooled to generate one composite sample per plot; therefore, there was one analytical observation per plot for each tissue and GA measurement. These clarifications have been added to the revised Methods section to better connect the field design, sample pooling, and statistical model.

The issue of unbalancedness is still not well acknowledged or addressed. I appreciate that this is (now) historical data and it is out of scope to remedy the imbalance by collecting more data. Nevertheless, it is important still that the statistical inference is accurate and doesn't overstate what the data imply. l202-230 unfortunately still feel muddled with respect to the assumed statistical model. l204-206 refer to "fitting the model", but does so before fully specifying the model: which coefficients are random, and with what variance structure? The sentence "Differences may present under an unbalanced design as in this investigation, and PROC GLIMMIX is the best for significant testing due to the likelihood-based method (which is the ultimate standard for all statistical analyses), PROC MIXED supposes to be unbiased for the estimate of variance component because of the restricted maximum likelihood and the application of mixed model equations for random effects, and PROC GLM provided mean predictions of random effects by the method of moment (i.e. the expected mean square or EMS)" is very unclear to me. It does not describe the statistical assumptions or methods directly, but rather refers to SAS procedures and seems to expect the reader to reverse-engineer the implied model from that. “PROC GLM provided mean predictions of random effects by the method of moment” is especially confusing: my understanding is that PROC GLM applies to fixed effects rather than random effects, so what is meant by this statement is at best unclear. This whole section remains

muddled, suggests over-reliance/over-confidence in software, and still underplays the problems caused by the unbalanced design. Likelihood-based methods are not automatically reliable in small or highly unbalanced settings, and similarly REML "unbiasedness" is not guaranteed in that same setting. It should not be for the reader or reviewer to have to unpick this.

Response:
The manuscript was revised to clarify the methodological approaches. Specifically, to estimate variance components, hybrid, site, replicate within site, and the hybrid-by-site interaction were all treated as random effects, with the residual term representing error variance. Random effects were assumed to be independently and identically distributed within each classification, each associated with its own variance component. Variance was partitioned into these additive components using SAS PROC MIXED with REML (Restricted Maximum Likelihood) estimation, a well-established approach for variance component estimation in unbalanced trial designs. The significance of variance components was then evaluated using PROC GLIMMIX with the COVTEST option via likelihood ratio tests. Because the null hypothesis places a variance component on the boundary of the parameter space at zero, the resulting test statistic follows a mixture chi-square distribution. Significance was evaluated at the 5%, 1%, and 0.1% levels without multiplicity correction.
For variance components that were found to be significant, additional analyses were performed to examine mean changes for individual hybrids across sites, thereby illustrating the extent to which hybrid effects were connected across sites and characterizing site-specific hybrid patterns.
These analyses were conducted within a linear mixed model framework using PROC MIXED. hybrid, site, and the hybrid-by-site interaction were treated as fixed effects, whereas Replicate nested within Site was modeled as a random effect. Treating hybrid and site as fixed effects produced local LS-means for characterizing hybrid-by-site patterns, while modeling nested replications as random prevented estimates from being biased by local block-level anomalies.
In addition, we removed references to SAS PROC GLM, which had been used only for intermediate data exploration, so that the manuscript now reflects only the analyses underlying the reported results.
Consequently, the approach provides valid and reliable inference for unbalanced data, though statistical power may be reduced relative to a fully balanced design.

On multiple testing, I also found the authors' response confusing ("The multiple testing was not considered because the relative comparison (e.g. hybrid versus site)…"), and it would help to be more explicit. In Table 5, are you not reporting significance tests for variance components (at least hybrid, site, and hybrid × site, I think) separately for 10 GA–tissue combinations, i.e. at least 30 formal tests? Then later there are five separate GA1–GA4 regression/correlation tests, one for each tissue. At present these nominal p-values appear to be interpreted directly, and merely noting significance at multiple levels (5%, 1%, and 0.1%) does not really address the multiplicity issue. I am not suggesting that a formal multiple-testing correction is needed in what is partly an exploratory study, but the manuscript should at least acknowledge the number of related tests being carried out and temper the interpretation of marginal findings accordingly.

Response:
The revisions were made in the manuscript to address this concern. The primary objective of this analysis was to describe how variation in GA levels was partitioned among model components, rather than to perform confirmatory hypothesis testing. For each GA-by-tissue combination, variance components associated with hybrid, site, replicate nested within site, and the hybrid-by-site interaction were evaluated using individual likelihood ratio tests, resulting in 40 variance-component tests in total. No multiplicity adjustment was applied because these tests were used for an exploratory assessment of variance structure. Accordingly, the resulting p-values should be interpreted descriptively, together with the estimated variance components and their relative contributions to total variation. Although conducting 40 tests increases the chance of false positives, effects supported by both substantial estimated variance contributions and very small p-values provide stronger evidence of meaningful structure in the data than marginally significant effects.

On confidence intervals, I do not understand why the authors regard the log scale as an obstacle (since variance components are naturally on that scale anyway). Confidence intervals are precisely what convey uncertainty in weakly identified settings, such as stemming from unbalanced design.

Response:
Variance components were estimated by fitting an all-random model that included hybrid, site, replicate nested within site, the hybrid-by-site interaction, and residual error. This model was specified to decompose total response variation and quantify the relative contributions of environmental, design-related, and interaction sources across sites. Because the primary objective was to identify the main drivers of variability, results are presented as variance-component point estimates and their relative percentage contributions. These measures provide the most direct and practical basis for evaluating the importance of each source of variation.

Reviewer #3: This manuscript reports a validated high-throughput LC-MS/MS workflow and uses it to quantify the bioactive gibberellins GA1 and GA4 in five maize tissues from ten commercial hybrids grown at six U.S. field sites, partitioning the variation into genetic (hybrid), environmental (site), and interaction components. The analytical method appears sound and the geographic breadth is a genuine strength, so the descriptive dataset has value as a baseline resource.

I have read the previous review round. The authors have made constructive changes — the Results and Discussion are now separated, the experimental design and sampling are described in more detail (Table S1), the GA1–GA4 correlation discussion has been appropriately tempered to acknowledge its correlational nature, and the closing claims have been softened to "preliminary insights." I appreciate these revisions.

That said, my main remaining concern is that the conclusions still reach beyond what the measured data can support, and this stems in large part from what was measured. Only the two bioactive end-products (GA1, GA4) were quantified. A steady-state pool reflects the balance of biosynthesis, 2β-hydroxylation-mediated inactivation, and conjugation, so the present data cannot attribute any observed change to a specific step. This is particularly problematic for the central claim that site (i.e., environment) is the dominant factor: the manuscript cannot say whether a site-associated change in GA1/GA4 arises from altered biosynthetic flux or from altered inactivation.

Maize is an early-13-hydroxylation (GA1-type) species, in which GA1 is inactivated to GA8 and GA4 to GA34 by GA2ox. At minimum, the authors must (i) state this limitation explicitly and (ii) remove or rephrase "biosynthesis" and "plasticity of the gibberellin pathway" language in favour of "endogenous level/abundance" wherever flux was not measured. To support any mechanistic interpretation of the site effect, quantification of the principal inactivated GAs (GA8, GA34) and key precursors (e.g., GA20, GA9), together with active-to-inactive ratios (GA1/GA8, GA4/GA34), would help indicate whether a given change reflects enhanced synthesis or reduced catabolism — although, as these are themselves steady-state pools, such ratios remain indirect proxies for the underlying flux.

Response:
Thanks for the comments. We revised the Abstract, Introduction, Discussion, and Conclusion by deleting the language such as “biosynthesis,” “pathway plasticity,” and “regulation”. Biosynthetic pathway was not discussed in the paper.

Methods (L167–181). Present the LC gradient as a table including the initial hold, and confirm the steep short segments (e.g., B 26%→55% over 0.1 min) are not transcription errors. Critically, retention times for GA1, GA4, and the internal standards are not reported; please add them (and a representative MRM chromatogram near the LLOQ), as they are needed to judge whether this unusual gradient performs as intended.

Response:
Thank you for this helpful suggestion. We have revised the Methods section to present the LC gradient as Table 2, including the initial hold. We confirm that the steep, short gradient segments, such as the change from 26% to 55% B over 0.1 min, are

intentional and are not transcription errors.
We have also added the retention times for GA1, GA4, and the corresponding internal standards in Table 3. In addition, we provided a representative MRM chromatogram at the LLOQ (standard 1), as Supporting Information S1 Fig. This added information should allow readers to better assess the chromatographic performance of the method and confirm that the gradient performs as intended.
S1 Fig. The representative chromatogram of LLOQ (standard 1)
Top left: TIC, Top right: XIC of GA1, Bottom left: XIC of GA1-D4 and GA4-D2, Bottom right: XIC of GA4.

Table 3 (pollen). The text states that pollen data were not reported because of its short stability window (L262–263), and pollen is correctly absent from Tables 4–5 and Figs 2–4. However, Table 3 still lists method-validation data for pollen.

Response:
Thank you for raising this point. The pollen results included in Table 3 refer only to the method-validation experiment. We validated the method for pollen along with the other maize tissues, including assessments of accuracy, precision, extraction efficiency, recovery, and benchtop stability; therefore, the validated pollen data are appropriately included in Table 3.
However, pollen had a much shorter benchtop stability window than the other tissues. Although this was manageable during method validation because only a small number of samples were processed, it was not feasible for the large field-sample set. The field study included more samples, and pollen sample preparation could not be completed within the validated stability window. For this reason, we did not consider the field pollen measurements sufficiently reliable for downstream biological interpretation and therefore excluded pollen from Tables 4–5 and Figures 2–4 for field data.

Minor comments
Introduction (L49). "Gibberella fujikuroi" → the accepted name is Fusarium fujikuroi (teleomorph Gibberella fujikuroi).
Response: “Gibberella fujikuroi" was changed to “Fusarium fujikuroi”.

Methods (L157). "until about 400 mL of aqueous phase was left" — should be 400 µL.
Response: 400 “mL” was corrected to 400 “µL”.

Methods (L152). "Nutating Mixter" → Mixer; and a missing space after "88-861-043)" before "After incubation".
Response: "Nutating Mixter" was changed to “Mixer”, and a space was added before "After incubation".

Methods (L168). "AB Sxiex OS" → "AB Sciex OS"
Response: "AB Sxiex OS" was corrected to "AB Sciex OS".

7. PLOS authors have the option to publish the peer review history of their article (what does this mean?). If published, this will include your full peer review and any attached files.

If you choose “no”, your identity will remain anonymous but your review may still be made public.

Do you want your identity to be public for this peer review? For information about this choice, including consent withdrawal, please see our Privacy Policy.
Reviewer #1: No
Reviewer #3: No

[NOTE: If reviewer comments were submitted as an attachment file, they will be attached to this email and accessible via the submission site. Please log into your account, locate the manuscript record, and check for the action link "View Attachments". If this link does not appear, there are no attachment files.]

	To ensure your figures meet our technical requirements, please review our figure guidelines: https://journals.plos.org/plosone/s/figures<br>You may also use PLOS's free figure tool, NAAS, to help you prepare publication quality figures: https://journals.plos.org/plosone/s/figures#loc-tools-for-figure-preparation.<br>NAAS will assess whether your figures meet our technical requirements by comparing each figure against our figure specifications.
**Additional Information:**	
**Question**	**Response**
**Competing Interests**<br><br>Use the instructions below to enter a competing interest statement for this submission. On behalf of all authors, disclose any competing interests that could be perceived to bias this work—acknowledging all financial support and any other relevant financial or non-financial competing interests.<br><br>This statement is required for submission and **will appear in the published article** if the submission is accepted. Please make sure it is accurate and that any funding sources listed in your Funding Information later in the submission form are also declared in your Financial Disclosure statement.<br><br>View published research articles from *PLOS ONE* for specific examples.<br><br>**NO authors have competing interests**<br><br>Enter: *The authors have declared that no competing interests exist.*<br><br>**Authors with competing interests**<br><br>Enter competing interest details beginning with this statement:<br><br>*I have read the journal's policy and the authors of this manuscript have the following competing interests: [insert competing interests here]*	The authors have declared that no competing interests exist.

Powered by Editorial Manager® and ProduXion Manager® from Aries Systems Corporation

* typeset	
**Data Availability**<br><br>Authors are required to make all data underlying the findings described fully available, without restriction, and from the time of publication. PLOS allows rare exceptions to address legal and ethical concerns. See the PLOS Data Policy and FAQ for detailed information.<br><br>A Data Availability Statement describing where the data can be found is required at submission. Your answers to this question constitute the Data Availability Statement and **will be published in the article**, if accepted.<br><br>**Important:** Stating 'data available on request from the author' is not sufficient. If your data are only available upon request, select 'No' for the first question and explain your exceptional situation in the text box.<br><br>Do the authors confirm that all data underlying the findings described in their manuscript are fully available without restriction?	Yes - all data are fully available without restriction
Additional data availability information:	
**Financial Disclosure**<br><br>Enter a financial disclosure statement that describes the sources of funding for the work included in this submission. Review the submission guidelines for detailed requirements. View published research articles from *PLOS ONE* for specific examples.<br><br>This statement is required for submission and **will appear in the published article** if the submission is accepted. Please make sure it is accurate.	Yes

**Funded studies**<br>Enter a statement with the following details:<br>• Initials of the authors who received each award<br>• Grant numbers awarded to each author<br>• The full name of each funder<br>• URL of each funder website<br>• Did the sponsors or funders play any role in the study design, data collection and analysis, decision to publish, or preparation of the manuscript?<br><br>Did you receive funding for this work?	
**Ethics Statement**<br><br>Enter an ethics statement for this submission. This statement is required if the study involved:<br><br>• Human participants<br>• Human specimens or tissue<br>• Vertebrate animals or cephalopods<br>• Vertebrate embryos or tissues<br>• Field research<br><br>Write "N/A" if the submission does not require an ethics statement.<br><br>General guidance is provided below. Consult the submission guidelines for detailed instructions. **Make sure that all information entered here is included in the Methods section of the manuscript.**	Plant materials consisted of conventional crop varieties grown in experimental plots that were collocated with USDA-permitted materials under Authorization No. AUTH-0000233243

Powered by Editorial Manager® and ProduXion Manager® from Aries Systems Corporation

**Format for specific study types**<br><br>**Human Subject Research (involving human participants and/or tissue)**<br>• Give the name of the institutional review board or ethics committee that approved the study<br>• Include the approval number and/or a statement indicating approval of this research<br>• Indicate the form of consent obtained (written/oral) or the reason that consent was not obtained (e.g. the data were analyzed anonymously)<br><br>**Animal Research (involving vertebrate animals, embryos or tissues)**<br>• Provide the name of the Institutional Animal Care and Use Committee (IACUC) or other relevant ethics board that reviewed the study protocol, and indicate whether they approved this research or granted a formal waiver of ethical approval<br>• Include an approval number if one was obtained<br>• If the study involved *non-human primates*, add *additional details* about animal welfare and steps taken to ameliorate suffering<br>• If anesthesia, euthanasia, or any kind of animal sacrifice is part of the study, include briefly which substances and/or methods were applied<br><br>**Field Research**<br><br>Include the following details if this study involves the collection of plant, animal, or other materials from a natural setting:<br>• Field permit number<br>• Name of the institution or relevant body that granted permission	
**Data Availability**<br><br>Before publication, Authors are required to make fully available and without restriction all data underlying their findings. Sharing the data underlying your findings increases the visibility, credibility, and impact of your work, while supporting transparency and future scientific discovery. Please see our PLOS Data	All relevant data are within the manuscript and its Supporting Information files.

Powered by Editorial Manager® and ProduXion Manager® from Aries Systems Corporation

Policy page for detailed information on this policy.<br><br>A **Data Availability Statement**, detailing where the data can be accessed, is required at first submission. Insert your Data Availability Statement in the box below.<br><br>Please see the data reporting section of our submission guidelines for instructions on what you need to include in your Data Availability Statement. Examples of acceptable Data Availability Statements are included here for reference:<br>1. The minimal data set [and accompanying code, where generated] is available at [repository name] via [DOI hyperlink]<br>2. No data was generated by this study. The following existing data sources were used: [dataset name] from [dataset location] available via [web address or DOI hyperlink].<br>3. For Study Protocols: No datasets were generated or analysed during the current study. All relevant data from this study will be made available upon study completion.<br><br>*PLOS allows rare exemptions to address legal and ethical concerns. If you have legal or ethical restrictions, please use the box below to detail these in full sentences for the Journal team to consider.*<br><br>This statement will be typeset if the manuscript is accepted for publication.	
Please add funding details.<br>as follow-up to "**Financial Disclosure**<br><br>Enter a financial disclosure statement that describes the sources of funding for the work included in this submission. Review the submission guidelines for detailed requirements. View published research	Funding for this research was provided by Bayer CropScience LLC.<br>The funder provided support in the form of salaries for authors XG, NS, CJ, JG, EA, CD, CP, SS, and YW, but had no additional role in study design, data collection and analysis, decision to publish, or preparation of the manuscript. The specific roles of these authors are articulated in the “Author Contributions” section.

articles from *PLOS ONE* for specific examples.<br><br>This statement is required for submission and **will appear in the published article** if the submission is accepted. Please make sure it is accurate.<br><br>**Funded studies**<br>Enter a statement with the following details:<br>• Initials of the authors who received each award<br>• Grant numbers awarded to each author<br>• The full name of each funder<br>• URL of each funder website<br>• Did the sponsors or funders play any role in the study design, data collection and analysis, decision to publish, or preparation of the manuscript?<br><br>Did you receive funding for this work?"	
Please select the country of your main research funder (please select carefully as in some cases this is used in fee calculation).<br>as follow-up to "**Financial Disclosure**<br><br>Enter a financial disclosure statement that describes the sources of funding for the work included in this submission. Review the submission guidelines for detailed requirements. View published research articles from *PLOS ONE* for specific examples.<br><br>This statement is required for submission and **will appear in the published article** if the submission is accepted. Please make sure it is accurate.	UNITED STATES - US

**Funded studies**<br>Enter a statement with the following details:<br>• Initials of the authors who received each award<br>• Grant numbers awarded to each author<br>• The full name of each funder<br>• URL of each funder website<br>• Did the sponsors or funders play any role in the study design, data collection and analysis, decision to publish, or preparation of the manuscript?<br><br>Did you receive funding for this work?"	

Aug 2nd, 2026

Dear Academic Editor,

Thank you for the opportunity to revise our work and resubmit it for your consideration. We are resubmitting our research article entitled “Natural variation of bioactive gibberellin levels in maize tissues across North America” for consideration for publication in *PLOS ONE*.

The following items have been addressed in the revised submission:

- A point-by-point response to the Academic Editor and reviewers has been uploaded as a separate file labeled “Response to Reviewers.”
- A revised manuscript with tracked changes has been uploaded (“Revised Manuscript with Track Changes”).
- A clean version of the revised manuscript without tracked changes has been uploaded (“Manuscript”).
- The Funding Statement and Competing Interests Statement updated last time (included below).
- Updated S1 Table and added S1 Fig.

Author change
In previous submission, we request authorship change with 1. Added one author Shubha Shakya 2. Change the author’s order. We also have sent an email regarding the change. Please let us know if there are any questions.

Updated financial disclosure statement
Funding for this research was provided by Bayer CropScience LLC.
The funder provided support in the form of salaries for authors XG, NS, CJ, JG, EA, CD, CP, SS, and YW, but had no additional role in study design, data collection and analysis, decision to publish, or preparation of the manuscript. The specific roles of these authors are articulated in the “Author Contributions” section.
Updated competing interests statement
A patent application has been filed for this work. This does not alter our adherence to *PLOS ONE* policies on sharing data and materials.

Once again, thank you for your consideration. We look forward to hearing from you.

Best regards,

Yanfei Wang, Ph.D.
Bayer CropScience
700 Chesterfield Parkway West
Chesterfield, MO 63017
(314)624-2832
Yanfei.wang1@bayer.com

RESTRICTED

Natural variation of bioactive gibberellin levels in maize tissues across North America

Xia Guan, Nitin Supekar, Changjian Jiang, Junhong Guo, Edwards Allen, Charles Dietrich, Cody Postin, Shubha Shakya, Yanfei Wang*

Bayer CropScience LLC., Chesterfield, Missouri, United States of America

*Corresponding Author

E-mail: yanfei.wang1@bayer.com

Keywords: Gibberellin A1 (GA_1), Gibberellin A4 (GA_4), Liquid chromatography-tandem mass spectrometry (LC-MS/MS), natural variation, conventional maize tissues, stalk, leaf

Abstract

Gibberellins (GA) are endogenous phytohormones that regulate growth and development throughout the plant lifecycle. While gibberellins have been extensively studied under controlled conditions for their fundamental role in plant adaptation and agronomic trait development, field-based data on their natural variation of bioactive GA levels across diverse agricultural genotypes and environments remains limited. To address this knowledge gap, we analyzed bioactive gibberellin A1 (GA_1) and gibberellin A4 (GA_4) in multiple tissues from commercial conventional maize hybrids grown in diverse field environments using high-throughput liquid chromatography-tandem mass spectrometry (LC-MS/MS). This study provides quantitative measurements of endogenous GA_1 and GA_4 levels in maize tissues collected under standard agricultural practices and describes variation influenced by genetic background and environmental conditions. Our findings demonstrate that GA_1 and GA_4 concentrations from conventional maize samples varied across tissue types, hybrid, and field sites. Analysis of variance components indicated that the site variation was statistically significant in five of ten gibberellins by tissue combinations, while the genetic component was significant only for GA_4 in grain samples, suggesting that environment factors have a larger effect than genetic factors Positive correlations between GA_1 and GA_4 were observed consistently across tissues except root. These findings offer preliminary insights into the interplay between genetics and environment across germplasm and environments that have been previously described.

Introduction

Gibberellins (GA) are endogenous phytohormones that play a crucial role in regulating plant growth and development throughout the plant lifecycle. They were first discovered as secondary metabolites in the fungus *Fusarium fujikuroi*. [1, 2] Gibberellins comprise a class of diterpenoid carboxylic acids that possess the tetracyclic ent-gibberellane carbon skeletal structure. Among various gibberellins, only a few, e.g., GA_1, GA_3, GA_4 and GA_7 exhibit significant biological activity [3]. GA_1 and GA_4 are the major active gibberellins that promote growth and development in plant species. They regulate and control diverse developmental processes such as seed germination, stem elongation, leaf expansion, and flower and fruit development [2, 4]. Additionally, gibberellins regulate plant adaptation to both biotic and abiotic stresses [5, 6].

The biosynthesis and signaling pathways of gibberellins are intricate systems that exhibit complex spatiotemporal patterns across cells, tissues, and developmental stages [7, 8]. These patterns result from multiple enzymatic steps and feedback mechanisms that respond dynamically to both endogenous and environmental signals [9, 10]. Environmental conditions can influence measured gibberellin pools through effects on biosynthesis, catabolism, conjugation, transport, or signaling [11], leading to stunted growth and altered development, while optimal conditions enhance gibberellin production and promote vigorous growth [12]. The movement of gibberellins between cells and across plant organs, coupled with their variable distribution patterns, creates challenges for understanding hormone-environment interactions in agricultural settings [13-15].

Due to the vital biological activity of gibberellins in plant growth and development, several applications have been developed with commercial and biotechnological utilization; exogenous applications of gibberellins to promote growth, and exogenous applications of its concentration or the genetic modification of biosynthesis to optimize plant phenotypes and environmental responses

[10]. Research in recent years has shown that gibberellins have direct and indirect effects on the regulation of many plant traits. Many plant varieties with improved agronomic traits (e.g., dwarf phenotypes or increased biomass) are shown to be related to gibberellin activity and signaling [16], thus manipulation of gibberellin levels is commonly used in agricultural practices to optimize plant growth and yield.

Maize (*Zea mays* L.; corn) is a major agricultural commodity, contributing significantly to the economy of many countries and serves as a staple food for many populations worldwide [17]. Short maize hybrids have been created through conventional breeding by researchers with the objectives to improve performance under high density and improve lodging tolerance [18]. However, breeding short traits into existing maize hybrids is time consuming, challenging, and can affect environmental responses. Genetically modified maize can be engineered through the enhancement or suppression of hormone pathways, including gibberellin biosynthesis. Scientists have developed a genetically modified short stature maize ideotype by suppressing maize genes that regulate the levels of gibberellins, reducing plant stature without undermining maize yields [19].

Accurate quantification of endogenous GA levels is valuable for describing variation across tissues, genotypes, and environments and for providing baseline context for future studies [20]. While previous studies have quantified bioactive gibberellins in maize tissues [8, 19, 21-24], these analyses were typically limited to specific varieties or locations, lacking the scope needed to characterize natural variation across diverse agricultural conditions. Establishing baseline patterns of endogenous gibberellin abundance in agricultural settings can support subsequent research. First, understanding the natural variability of gibberellin concentrations in conventional maize tissues - a primary determinant of plant stature – and

distinguishing between environmentally-induced and genetically-controlled variation provides essential context for crop improvement efforts. This baseline knowledge is particularly valuable for developing and evaluating new short-stature varieties, where gibberellin-related genetic modifications must be assessed against the background of natural gibberellin variability that occurs across diverse field environments.

Second, while gibberellin biology has been extensively studied under controlled conditions [19], quantifying the magnitude of gibberellin variation in commercial hybrids under real-world agricultural conditions helps bridge the gap between laboratory findings and field performance. Such information may provide context for future studies evaluating gibberellin-modified traits across growing environments, where environmental stresses can significantly impact gibberellin-mediated growth processes and ultimately affect yield potential. Understanding the relative contributions of genetic background and environmental factors to gibberellin concentrations in maize tissue can inform breeding strategies and help develop more resilient short-stature varieties that maintain desired height characteristics across diverse growing conditions.

Understanding the natural range and stability of endogenous gibberellin levels in conventional hybrid maize across diverse environments may help contextualize future studies of gibberellin related traits changes in new hybrids and predicting their performance across different agricultural regions. The purpose of this study is to quantify ~~major bioactive endogenous~~ GA_1 and GA_4 in maize ~~tissues~~ by the validated LC-MS/MS method across conventional maize hybrids, tissue types, and environments, which could provide a baseline understanding of the contribution of genetics and environment to the variation of gibberellin content.

Materials and methods

Maize field trials

Maize samples from ten commercially available conventional maize hybrids were collected from field trials during the 2023 growing season. Six field trial locations were spread across states representing major maize-growing regions of the United States (Iowa, Illinois, Indiana, Missouri, Nebraska, and Ohio) (Table 1). Plant materials consisted of conventional crop varieties grown in experimental plots that were collocated with USDA permitted materials under Authorization No. AUTH0000233243.

Each site was planted in a randomized complete block design with four replicated blocks. Hybrids listed in Table 1 were included in each of the four replicated blocks where they were planted, in addition to other hybrids not associated with this study. Within a block, each hybrid was planted in an approximately 6 m × 6 m plot. Each site received a unique randomization scheme. Blocks at each site were arranged to limit variability in environmental conditions such as soil type, slope, and previous cropping history. Normal agronomic practices were followed for each site. Trial maintenance (fertilization, pesticide use, tillage practices, irrigation, etc.) varied across sites, but all maintenance activities were applied uniformly within a site. Maize samples were collected throughout the growing season and included the following tissues: leaf, root, stalk, pollen, forage, and grain. The same tissues collected from each plot were pooled into a single sample. Tissue samples were non-systematically selected with consistent field sampling criteria: plot ends were avoided, and plants were chosen to be competitive and representative of the plot as a whole. The selected tissue samples from each plot were combined to generate one pooled sample per plot, which served as the observational unit for downstream analysis. This pooling approach was applied consistently across all sites. Sampling details can be found in Table S1. Specifically, it

specifies the tissue-specific sampling amount used to generate one pooled sample per lot: 60 leaf sections, 8 roots, 4 stalks, 10 mL pollen, 1 forage plant, or 1 kg grain, depending on tissue type, along with the corresponding number of sampled plants and growth stages. All samples were kept on dry ice immediately after collection until storage in a -80ºC freezer prior to analysis, with the exception of grain samples which were kept at ambient temperature.

Table 1. Field trial locations and maize hybrid names

Site Code	County, State	Hybrid Name
Site 1	Jefferson County, Iowa	Dekalb DKC58-32
		Lewis 12CV779
		Channel 210-79
Site 2	Shelby County, Illinois	Stone 6290
		Hubner H2390
		Dekalb DKC65-92
Site 3	Boone County, Indiana	Dekalb DKC63-58
		Hubner H2390
Site 4	Adair County, Missouri	Dekalb DKC58-32
		Lewis 12CV779
		Stone 6070
Site 5	York County, Nebraska	Stone 6290
		Hubner H2390
		Dekalb DKC64-32
Site 6	Miami County, Ohio	Dekalb DKC58-32
		Stewart 12CV209
		Channel 210-79

Reagents

Gibberellin standards were used as reference standards for the analytical procedures or calibration of equipment. Stable-isotope-labelled gibberellin standards were used as internal standards (synthesized by OlChemlm). GA_1 was synthesized by Toronto Research Chemicals (purity: 97%) and GA_4 was synthesized by Thermo Scientific (purity: 90.30%). Formic acid (purity: 99%), ethyl

acetate (HPLC grade) and methanol (purity: ≥99.8%) were purchased from Fisher Scientific and acetic acid (analytical grade) was obtained from Millipore, AR.

Sample preparation

After cryomilling maize tissues, 50 ± 5 mg of fresh frozen -sample was weighed into a pre-chilled 1.5 mL glass tube placed on dry ice. Next, on wet ice, 10 µL of internal standard working solutions (100 ng/mL of GA_1-D4 and 25 ng/mL of GA_4-D2) and 1.00 mL of pre-chilled extraction buffer (80% methanol with 0.5% formic acid) were added to each sample. The samples, arranged in a 96-well formatted rack, were manually agitated 40 times to thoroughly suspend the tissue in the extraction buffer, followed by an overnight incubation (approximately 15 hours) at 4°C with gentle mixing at 50 rpm on a Fisher brand Mixer (Fisher Scientific, 88-861-043). After incubation, the samples were centrifuged at 2000 rpm for 10 minutes at 4°C. The supernatant was transferred to a 96-well polypropylene sample collection plate. The pellets were re-extracted by adding another 1 mL of extraction buffer following the same procedure as above. The combined supernatants evaporated under nitrogen flow at 35°C using a microplate sample evaporation system (Biotage SPE Dry 96, SD-9600-DHS-NA), until about 400 μL of aqueous phase was left. Subsequently, the extracts were loaded into a Biotage 96-well supported liquid extraction (SLE) plate (Biotage, 8200400P01), which was mounted on a glass-coated 96-well collection plate. The analyte gibberellins were eluted with 2 × 0.9 mL of ethyl acetate. The eluate was then evaporated to dryness under nitrogen by using the SPE Dry 96. The dried samples were reconstituted in 100 µL of reconstitution buffer (20% Methanol with 0.1% formic acid), then vortexed at 1200 rpm at room temperature for 3 minutes. Following this, the samples were centrifuged at 2000 rpm for 3 minutes at 4°C, and 90 µL of the supernatant was transferred into a 96-well plate with glass inserts, kept on wet ice prior to LC-MS/MS analysis.

LC-MS/MS analysis of gibberellins in maize tissues

Analytical instruments include mass spectrometer (Sciex triple Quad 7500), HPLC system (ExionLC 20A system) and data acquisition system (PC-based workstation with AB Sciex OS software). Reconstituted samples were separated on a 1.7 µm Kinetex 100 Å C18 LC column (100 mm × 2.1 mm) (Phenomenex, 00D4475AN) at a flow rate of 0.40 mL/min and a column oven of 40˚C. Mobile phase A is 0.5% acetic acid in water and mobile phase B is 0.5% acetic acid in methanol. The LC gradient is presented in Table 2. Mass spectrometric conditions are as follows: polarity mode, negative ionization; scan type, MRM; spray voltage, -1800 V; ion source gas 1, 40 psi; ion source gas 2, 45 psi; source temperature, 500°C; curtain gas, 40; CAD gas, 10; entrance potential (EP), -10 V. The multiple reaction monitoring (MRM) transitions for each analyte and its stable isotope-labeled internal standard (IS) can be found in Table 3 with respective optimized collision energy (CE) and collision cell exit potential (CXP). The representative MRM chromatogram of GA_1 and GA_4 in the LLOQ sample (standard 1) was provided as Supporting Information S1 Fig.

Table 2. LC gradient

Time (min)	Flow (mL/min)	B.Conc (%)
0	0.40	26
5.30	0.40	26
5.40	0.40	55
10.20	0.40	58
10.30	0.40	80
12.70	0.40	81
12.80	0.40	98
14.00	0.40	98
14.10	0.40	26
16.00	0.40	26

Table 3. MRM transition parameters

Compound ID	Retention time (minutes)	Q1 mass (Da)	Q3 mass (Da)	Dwell (ms)	CE(V)	CXP(V)
GA_1	4.63	347.19	273.10	100	-32	-8
GA_4	9.37	331.19	213.13	100	-40	-7
GA_1-D4 (IS)	4.48	351.19	277.07	100	-36	-9
GA_4-D2 (IS)	9.34	333.14	259.06	100	-33	-9

MS data analysis

Raw data files were analyzed using Analytics in SCIEX OS software. A standard curve for each endogenous gibberellin was generated as the ratio of the gibberellin standard's response (peak area) to the stable-isotope-labelled standard response (peak area), plotted against the concentrations of the gibberellin standard. A linear regression model was used for the quantification of the endogenous gibberellin levels with $1/x^2$ weighting. The final concentration of gibberellins in the tissues was reported in pmol/g.

Variance component analysis to compare genetic and environmental variations

To compare genetic and environmental contributions to GA1 and GA4 expression across five tissues, we first examined the distribution of the response variables. Visual inspection of the observed response and residual plots from the analysis indicated a log-normal distribution, supporting the use of log-transformed data in subsequent analyses. Then, a variance component analysis [25] was performed with the following model (1) and a log transformation $y = \log(x)$ for the normality assumption of the analysis.

$$y_{ijk} = \mu + s_i + r_{j(i)} + m_k + (sm)_{ik} + \varepsilon_{ijk} \quad (1)$$

where y_{ijk} is the observed response of each plot after transformation for the kth hybrid from the jth replicate (i.e. block) at the ith site, μ is the overall mean, s_i is the effect of the ith site, $r_{j(i)}$ is the effect of the jth replicate within the ith site, m_k is the effect of the kth hybrid, $(sm)_{ik}$ is the interaction effect of the kth hybrid with the ith site, ε_{ijk} is the residual. The term replicate and block will be used interchangeably in the following. Before fitting the model, Levene's tests were conducted to examine the assumption of the variance homogeneity, and non-significance was found for effects of sites, materials, and interactions on the variance. Different SAS procedures were used to estimate variance components, test significance, and examine changes in hybrid means across sites, with each procedure chosen to leverage its specific analytical strengths. To estimate variance components, Hybrid, Site, Replicate within Site, and the Hybrid-by-Site interaction were all treated as random effects, with the residual term representing error variance. Random effects were assumed to be independently and identically distributed within each classification, each associated with its own variance component. Variance was partitioned into these additive components using SAS PROC MIXED with REML (Restricted Maximum Likelihood) estimation, a well-established approach for variance component estimation in unbalanced trial designs. The significance of variance components was then evaluated using PROC GLIMMIX with the COVTEST option via likelihood ratio tests. Because the null hypothesis places a variance component on the boundary of the parameter space at zero, the resulting test statistic follows a mixture chi-square distribution. Significance was evaluated at 5%, 1%, and 0.1% levels without multiplicity correction. For variance components that were found to be significant, additional analyses were performed to examine mean changes for individual hybrids across sites, thereby illustrating the extent to which hybrid effects were connected across sites and characterizing site-specific hybrid patterns. These

analyses were conducted within a linear mixed model framework using PROC MIXED. Hybrid, Site, and the Hybrid-by-Site interaction were treated as fixed effects, whereas Replicate nested within Site was modeled as a random effect. Treating Hybrid and Site as fixed effects produced local LS-means for characterizing Hybrid-by-Site patterns, while modeling nested replications as random prevented estimates from being biased by local block-level anomalies. All analyses were performed separately for each gibberellin and tissue combination, and results were presented in the following sections.

For the assumptions of the analysis, in addition to the composition of the model (1) as described above, distributional assumptions were further examined by the residual plots from PROC MIXED which showed no evidence of violation of normality (after the log transformation on the original observed response) and variance heterogeneity (as an additional visual confirmation to the above Levene’s test). Overall, the use of SAS procedures with REML estimation was appropriate for variance component estimation, significance testing, and LS-means estimation, since these procedures are well suited to handling unbalanced data within a properly specified mixed-model framework.

Results

Method development and validation of gibberellins by LC-MS/MS

Plant ~~tissue~~ extracts contain complex mixtures~~, and~~ typically gibberellins are present in trace amounts. Therefore, it is necessary to apply both a consistent sample preparation method to reliably extract gibberellins from plant ~~tissues~~ and a sensitive technique to accurately quantify them.

In this study, a robust, reliable, and high-throughput method was developed for the simultaneous quantitation of gibberellins in six ~~maize tissue types~~ (grain, pollen, leaf, root, forage, and stalk). In

brief, the gibberellins were extracted from approximately 50 mg of sample, purified by SLE, and separated and quantified by LC-MS/MS using the isotope-labeled internal standard (IS) as shown in Fig 1. The method was validated by determining the linearity ($R^2 \geq 0.98$) over the concentration range of 20-30,000 pg/mL in six tissue types. 20 pg/mL is the LLOQ (lower limit of quantification). Precision of the method was obtained with intra-and inter-day relative standard deviations (≤17.13%) and accuracy for compounds typically ranging between 79-124%. The recoveries of our method ranged from 77.6% to 119.8% in six maize tissues (Table 4). The bench-top stability of the tissue extraction supernatant was evaluated at room temperature for each type of tissue, demonstrating stability for a duration of up to two hours for all tissues except pollen, which exhibited stability of only one hour. Additionally, the stability of the processed samples in the autosampler was assessed at a temperature of 4 °C, with all tissue samples maintaining stability for up to six days.

Fig 1. Workflow for Sample Preparation and Analysis of Gibberellins in Maize Tissues Left Panel: The workflow consists of 4 key steps: (1) sample preparation via cryomilling and weighing, (2) extraction of gibberellins with internal standards (IS) and extraction buffer, (3) SLE analyte purification through centrifugation-, supernatant collection, and reconstitution of the analyte, and (4) LC-MS/MS analysis for quantitation.

Right Panel: Equipment and components used in the workflow include a balance for weighing samples, a centrifuge for supernatant separation, a SLE plate for sample purification, and an LC-MS/MS system for gibberellin quantification.

Table 4. Accuracy, precision, extraction efficiency and recovery

Tissue type	Analytes	Accuracy (%)		Precision (CV%)		Extraction Efficiency (%)	Recovery (%)
		Intra-assay	Inter-assay	Intra-assay	Inter-assay		
**Stalk**	GA_1	83.92-114.86	95.78-100.71	0.56-11.5	1.80-14.52	94.56	96.56
	GA_4	87.69-113.10	90.62-105.81	0.42-11.69	2.51-9.33	91.52	115.72
**Grain**	GA_1	79.73-115.95	89.38-106.18	0.49-12.39	1.65-17.13	95.91	92.01
	GA_4	85.83-110.13	86.33-108.89	0.99-12.52	1.48-11.78	93.02	77.59
**Leaf**	GA_1	89.03-115.97	98.55-107.07	0.32-11.38	2.21-15.88	93.67	82.59
	GA_4	82.38-108.36	90.10-105.68	0.53-12.52	2.16-10.61	91.39	82.33
**Root**	GA_1	83.92-114.86	95.78-104.13	0.24-11.50	1.80-14.52	96.51	98.52
	GA_4	85.23-108.36	90.62-103.43	0.42-11.69	2.51-9.43	91.08	107.03
**Forage**	GA_1	83.92-123.06	92.36-110.33	0.32-9.13	2.81-13.98	94.44	93.40
	GA_4	87.69-108.95	90.62-103.43	0.42-11.69	2.51-9.33	91.71	101.89
**Pollen**	GA_1	79.79-115.95	95.18-105.44	0.32-11.38	2.21-14.28	94.78	86.95
	GA_4	81.63-110.13	88.31-108.89	0.99-12.52	1.48-11.78	91.70	76.89

Quantification of gibberellins in different maize tissue samples

The gibberellins in maize samples were extracted, purified, separated and quantified by LC-MS/MS. The short stability window of pollen tissue limited its suitability for large-scale sample handling; therefore, pollen data were not reported in this study. In total, 68 individual samples of each of the five other tissue types were analyzed and reported. Data with non-detection (ND) values were assigned values by replacing ND with half of the observed minimums for that tissue in order to apply the statistical analysis. The details regarding values given to ND samples are included in Table S2. Gibberellin concentrations in maize tissues were calculated based on the standard curve and the statistics were summarized in Table 5.

Table 5. Summary of GA_1 and GA_4 concentration in different maize tissues

Analyte	~~Tissue~~	Mean (pmol/g)	Standard Error	Min (pmol/g)	Max (pmol/g)
**GA_1**	Forage	7.9	2.61	0.4	131.1
**GA_1**	Grain	1.4	0.43	0.1	26.0
**GA_1**	Leaf	3.2	0.57	0.4	27.0
**GA_1**	Root	0.5	0.04	0.1	1.3
**GA_1**	Stalk	2.1	0.08	0.9	3.4
**GA_4**	Forage	1.9	0.56	0.1	29.2
**GA_4**	Grain	0.9	0.06	0.3	2.0
**GA_4**	Leaf	0.3	0.03	0.1	1.4
**GA_4**	Root	0.6	0.03	0.1	1.8
**GA_4**	Stalk	0.4	0.02	0.2	0.9

Variance components ~~in~~ five ~~tissues~~

Variance components describe how different sources of variation contribute to observed differences in gibberellin concentrations. Estimates of variance components for five tissues are listed in Table 6. The model variance was calculated as the sum of all components except the residual.

Table 6. Estimated variance components in log scale and the statistical significance from each source.

			Variance Components (log)[1]				
Analyte	Tissue	Variance (Model-log)	Varg	Vars	Vargxs	Varr	Vare
GA_1	Forage	0.39	0.00	0.13	0.19	0.07	1.88
GA_1	Grain	0.41	0.08	0.05	0.00	0.28	1.21
GA_1	Leaf	1.29	0.09	1.09***	0.10	0.01	0.25

GA_1	Root	0.11	0.04	0.02	0.00	0.05	0.67
GA_1	Stalk	0.10	0.01	0.07*	0.03**	0.00	0.03
GA_4	Forage	0.73	0.00	0.58*	0.00	0.14	1.09
GA_4	Grain	0.19	0.06*	0.09*	0.01	0.02	0.07
GA_4	Leaf	0.65	0.05	0.54**	0.07	0.00	0.47
GA_4	Root	0.05	0.00	0.01	0.03	0.01	0.20
GA_4	Stalk	0.05	0.00	0.00	0.05*	0.00	0.05

[1] hybrid (Varg), site (Vars), hybrid by site interaction (Vargxs), replicate (Varr), and residual (Vare). Variance (Model) is the sum of all components except Vare.

* indicates statistical significance at the 5% level, ** for significance at the 1% level, and *** at the 0.1% level.

The relative contribution of variance components (i.e., the percentage of each source to total variance) is presented in Fig 2. As shown in Table 6 and Fig 2, the residual variation (Vare) is greater than the model-explained variation in five out of ten gibberellin–tissue combinations. Because the variance components in Table 6 and Fig 2 were estimated using an all-random-effects model, statistical significance should be interpreted as evidence that these sources of variation are likely to contribute under repeated sampling of similar sites and hybrids. While conducting 40 statistical tests increases the risk of false positives, effects supported by both substantial variance contributions and highly significant p-values ($p<0.01$ and $p<0.001$) provide strong evidence of meaningful structure in the data rather than being random noise. Concentrations of gibberellin A1 (GA_1) and gibberellin A4 (GA_4) (mean ± 1 SE) in leaf and stalk were measured and plotted for 10 maize hybrids across six field sites, which are presented in Fig 3.

Fig 2. Variance Component Analysis of Genetic and Environmental Contributions to GA_1 and GA_4 Concentrations. The ~~figure presents the~~ variance component analysis of gibberellin A1

(GA_1) and gibberellin A4 (GA_4) concentrations across different tissues in maize, broken down into contributing sources of variation. Bar charts show the percentage contributions of variance components: Varg (genetic variance, red), Vars (site variance, green), Vargxs (genetic-by-site interaction variance, blue), Varr (replicate variance, yellow), and Vare (residual variance, gray). (A) Variance components for GA_1. (B) Variance components for GA_4.

Fig 3. GA_1 and GA_4 Concentrations in Leaf and Stalk Across Hybrids and Sites

Concentrations of gibberellin A1 (GA_1) and gibberellin A4 (GA_4) (mean ± SE) in leaf and stalk were measured and plotted for 10 maize hybrids across six field sites. Data are presented in pmol/g, with hybrids represented by different markers and colors, and site-wise trends connected by lines for visualization **Panels:** Top Left: Leaf GA_1 concentration, Top Right: Leaf GA_4 concentration, Bottom Left: Stalk GA_1 concentration, Bottom Right: Stalk GA_4 concentration

Correlation between GA1 and GA4 across tissues

Biosynthetic pathways for GA1 and GA4 share a common upstream pathway, and the subsequent parallel branches utilize the same families of enzymes (e.g., GA20ox, GA3ox) [10]. From this perspective, the correlation between GA_1 and GA_4 was investigated using biplots. The plot is based on the hybrid mean at each site as shown in Fig 4. In this plot, each mark represents the predicted hybrid mean over four replicates in log and then back transformed into pmol/g; five tissues are represented by different markers and colors. As shown in Fig 4, positive correlations were observed in four out of five tissues except root. The position and range of each tissue are represented by the differences in means (see Table 5) and the variation (see Table 6) among tissues. Since the hybrid mean at each site in Fig 4 consists of both the hybrid and site effects on the expressions, these positive correlations are due to similar trends in hybrid mean changes over sites between two gibberellins as shown in Fig 3. The regression coefficients of GA_4

on GA_1 in log were estimated as 0.01, 0.42, 0.42, 0.40, 0.49 for root, forage, leaf, grain, stalk with P values of 0.94, 0.10, 0.03, <0.01, <0.01, respectively, which are significant at the 5% level for leaf, grain, and stalk, and non-significance for forage. Notably, when examining residual variation after accounting for hybrid and site effects, positive correlations were observed with similar magnitude.

Fig 4. Biplot and Correlation Between GA_1 and GA_4 Means ~~Across Five Maize Tissues~~

The figure illustrates the relationship between gibberellin A1 (GA_1) and gibberellin A4 (GA_4) concentrations across ~~five maize tissues~~: root, forage, leaf, grain, and stalk. Each marker in the biplot represents the predicted hybrid mean for GA_1 and GA_4 at a specific site, derived from four replicates, expressed in pmol/g (log-transformed and back-transformed). ~~Different tissues~~ are distinguished by unique markers and colors.

Discussion

Quantitative analysis of gibberellins has advanced rapidly across diverse crops with the development of LC–MS/MS-based workflows [27], including methods for broad ~~GA~~ profiling [28, 29] and high-efficiency quantitation in complex plant matrices [20, 30]. This study focuses on ~~endogenous~~ GA_1 and GA_4 quantification ~~across five tissues of field-grown~~ commercial maize hybrids sampled across diverse environments, providing a baseline dataset for describing natural variation under standard agricultural practices. Among the five ~~tissues,~~ forage exhibited the highest bioactive gibberellins concentrations and the broadest dynamic range, with GA_1 ranging from 0.4 to 131.1 pmol/g and GA_4 from 0.1 to 29.2 pmol/g. Published data for forage tissue is limited; while available data for shoot tissue can serve as a reference. GA_1 levels in shoot ranged

from 0.04 to 274.4 pmol/g[31, 32] andGA_4 in the shoot tips ranged from 0.06 to 1.05 pmol/g [21, 33]

Stalk showed a narrower and more stable range (GA_1: 0.9–3.4 pmol/g; GA_4: 0.2–0.9 pmol/g), aligning closely with literature values (GA_1 ~1.8 pmol/g; GA_4: 0.33–0.98 pmol/g) [19]. This consistency suggests that stalk may be a useful tissue for comparison in future studies.

Leaf in this study showed GA_1 levels ranged from 0.4 to 27.0 pmol/g and GA_4 from 0.1 to 1.4 pmol/g. The GA_1 concentrations observed here are higher than literature-reported ranges of 0–1.8 pmol/g, possibly due to the limited number of samples reported previously, while GA_4 levels fall within the broader literature range of 0–3.5 pmol/g [19, 33, 34]. The elevated GA_1 levels may reflect differences in tissue sampling, developmental stage, genotype, field conditions, or study design. In contrast, the GA_4 levels, although lower than the upper literature limit, are consistent with expected physiological ranges and suggest that GA4 levels in leaf tissue appeared less variable than GA1 in this dataset.

Grain exhibited GA_1 levels from 0.1 to 26.0 pmol/g and GA_4 from 0.3 to 2.0 pmol/g, exceeding the ranges (GA_1: 0.29–2.0 pmol/g; GA_4: 0–1.2 pmol/g) reported from literature [22, 35]. This suggests that gibberellin levels in grain may span a broader range than previously reported. The tight range data from literature may be possibly reflecting differences in sample number, hybrid background, field site, developmental stage, or sampling design [35].

Root in this study showed GA_1 concentrations ranging from 0.1 to 1.3 pmol/g and GA_4 from 0.1 to 1.8 pmol/g. The relatively narrow range and lower mean values indicate that measured GA_1 and GA_4 levels in root were lower and less variable than in several above ground tissues in this dataset. These results support including below-ground tissues in future GA profiling studies to better describe tissue-specific GA abundance.

Overall, these comparisons highlight the broader dynamic range of bioactive GA_1 and GA_4 captured in this study, particularly ~~for~~ forage, leaf, and grain tissues. The broader ranges observed in ~~several tissues~~ may reflect the larger number of samples, field-grown conditions, developmental differences, and the use of commercial hybrids compared with prior studies [31]. The comparison of literature data with our findings reinforces the robustness of the LC-MS/MS method and provides a reference for future studies of endogenous GA_1 and GA_4 abundance in maize tissues.

The contribution of different sources of variance components to measured GA_1 and GA_4 levels was analyzed using an all-random-effect model. Model (1) included the effects of site, hybrid, the hybrid × site interaction, replicate nested within site, and residual. The hybrid effect represents genetic variation, and the site effect represents environmental variation among locations. Replicate nested within site accounts for variation among field replicates within each location, whereas the residual captures the remaining unexplained variation, including plant sampling variation in the field and assay variation in the laboratory. The hybrid × site interaction was further examined for tissues showing evidence of interaction in the variance component analysis.

Results in Table 6 and Fig 2 indicated that while both genetic and environment are important factors, the site is a major contributor as compared with the genetic background to the gibberellin levels. In addition, in five out of ten (gibberellins by tissue) combinations, residual variations are greater than the model variation, especially for forage and root.

Results in Table 6, together with means in Table 5, demonstrate substantial differences in the distribution of GA_1 and GA_4 among the five tissues. For example, forage exhibited the highest mean concentrations as well as the largest residual variances for both GA_1 and GA_4, whereas stalk showed intermediate mean concentrations but the lowest residual variances. Despite this lower

residual variability, stalk accounted for a higher proportion of total model-explained variation than forage, as shown in Fig 2.

Across the ten gibberellins-tissue combinations analyzed, a significant hybrid contribution was detected only for GA_4 in grain (Varg), compared with five combinations exhibiting significant site effects (Vars). Overall, six of the ten combinations showed at least one significant variance component attributable to site, hybrid, or site × hybrid interaction (Vargxs). Notably, only root showed no significant variance components for either GA_1 or GA_4, while hybrid × site interactions were significant for both gibberellins in stalk.

Estimation and interpretation of variance components depend on satisfaction of the model's normality assumptions. Following log transformation, the variance component estimates reported in Table 6 are presented on the log scale and should be interpreted in a relative sense, whereas mean concentrations and associated standard errors reported in Table 5 are expressed in absolute units (pmol/g).

The experimental design was constrained by hybrid adaptation and site capacity, resulting in different subsets of hybrids being planted at each site. Because commercial maize hybrids are bred for specific maturity zones and local growing conditions, it was not feasible to plant the same set of hybrids across the full range of environments represented in this study. This approach was necessary to achieve broad geographic and environmental coverage, despite resulting in an unbalanced hybrid–site design.

As a consequence of this design, estimation and statistical testing of variance components, especially hybrid × site interactions, are based on reduced degrees of freedom. For example, although the experiment included ten hybrids and six sites, a fully balanced design would have yielded 45 degrees of freedom for the interaction term, whereas the mixed-model analysis of the

available data provided only three. This imbalance limits the precision of variance component estimates and reduces statistical power for significance testing.

Despite these limitations, the unbalanced design does not preclude qualitative interpretation of the relative contributions of genetic, environmental, and interaction effects. The variance component analysis was conducted using an all-random-effects mixed model, and significance testing was performed using likelihood-based methods, which are appropriate for unbalanced data. While quantitative estimates should therefore be interpreted cautiously, trends comparing the relative importance of site, hybrid, and hybrid × site effects remain informative.

As shown in Fig. 3, gibberellin concentration profiles for many genetic backgrounds are largely parallel across sites, suggesting consistent environmental responses. However, some hybrids exhibit clear site-dependent variation, indicating that both genetic and environmental factors contribute meaningfully to gibberellin accumulation. For example, the hybrid Dekalb DKC58-32 showed the highest gibberellin concentrations at site 4 but relatively lower concentrations at other sites, illustrating environment-dependent expression patterns. Although the specific environmental drivers underlying these differences cannot be resolved in the present study, factors such as temperature, humidity, and rainfall are likely contributors, and further targeted experimentation will be required to assess their relative importance.

In our field dataset, GA_1 and GA_4 were positively correlated in forage, grain, leaf, and stalk, but not in root. Because these data were collected under standard agronomic conditions, the observed correlations could also reflect a shared response to overall plant physiological status (e.g., more vigorous growth) and to environmentally driven differences in growing conditions among sites, consistent with the strong site effects detected in the variance component analysis. Notably, the

GA_1–GA_4 association also persisted when examining residual variation after accounting for hybrid, site, and hybrid × site effects.

Conclusions

In this study, we applied a validated high-throughput LC-MS/MS method to simultaneously quantify the bioactive gibberellins GA_1 and GA_4 across multiple tissues of commercial maize hybrids grown in North American field environments. This work provides a field-based baseline dataset describing endogenous GA_1 and GA_4 variation under standard agricultural practices. We found that gibberellin concentrations varied significantly across tissues, with forage exhibiting the broadest dynamic range and highest levels, while stalk tissue showed narrower concentration ranges, which may make it useful for comparison in future studies.

Variance component analysis indicated that site-associated variation contributed to measured GA_1 and GA_4 levels in several tissue-by-analyte combinations. We identified a significant genetic (hybrid) contribution to GA_4 variance specifically in grain, which suggests that this tissue-analyte combination may warrant further study in larger, targeted experiments.

While this study provides a foundational dataset, it is important to note that it is based on a single growing season and limited to bioactive GA_1 and GA_4. Future multi-year studies and additional GA metabolites will be necessary to confirm these trends and further explore the impact of annual weather variations. We hope that the data and analyses presented here provide useful insights into the complex interplay between genetics and environment, in maize.

Acknowledgments

The authors thank Leah Riter, Vinod Jakkula, Edward Cargill, Mohamed Bedair, David Caldwell, Prema Karunanithi and Paul Tietz for providing valuable feedback on the manuscript. The authors also extend special thanks to Xin Li for his valuable input on the statistical analysis presented in the manuscript

References

1. Kurosawa E. Experimental studies on the nature of the substance secreted by the" bakanae" fungus. Nat Hist Soc Formosa. 1926;16:213-27.

2. Castro-Camba R, Sánchez C, Vidal N, Vielba JM. Plant Development and Crop Yield: The Role of Gibberellins. Plants (Basel). 2022;11(19). Epub 20221009. doi: 10.3390/plants11192650. PubMed PMID: 36235516; PubMed Central PMCID: PMCPMC9571322.

3. Yamaguchi S. Gibberellin metabolism and its regulation. Annu Rev Plant Biol. 2008;59:225-51. doi: 10.1146/annurev.arplant.59.032607.092804. PubMed PMID: 18173378.

4. Ubeda-Tomás S, Federici F, Casimiro I, Beemster GTS, Bhalerao R, Swarup R, et al. Gibberellin Signaling in the Endodermis Controls Arabidopsis Root Meristem Size. Current Biology. 2009;19(14):1194-9. doi: 10.1016/j.cub.2009.06.023.

5. Verma V, Ravindran P, Kumar PP. Plant hormone-mediated regulation of stress responses. BMC Plant Biol. 2016;16:86. Epub 20160414. doi: 10.1186/s12870-016-0771-y. PubMed PMID: 27079791; PubMed Central PMCID: PMCPMC4831116.

6. Illouz-Eliaz N, Ramon U, Shohat H, Blum S, Livne S, Mendelson D, et al. Multiple Gibberellin Receptors Contribute to Phenotypic Stability under Changing Environments. Plant Cell. 2019;31(7):1506-19. Epub 20190510. doi: 10.1105/tpc.19.00235. PubMed PMID: 31076539; PubMed Central PMCID: PMCPMC6635849.

7. Rizza A, Jones AM. The makings of a gradient: spatiotemporal distribution of gibberellins in plant development. Curr Opin Plant Biol. 2019;47:9-15. Epub 20180830. doi: 10.1016/j.pbi.2018.08.001. PubMed PMID: 30173065; PubMed Central PMCID: PMCPMC6414749.

8. Nelissen H, Rymen B, Jikumaru Y, Demuynck K, Van Lijsebettens M, Kamiya Y, et al. A local maximum in gibberellin levels regulates maize leaf growth by spatial control of cell division. Curr Biol. 2012;22(13):1183-7. Epub 20120607. doi: 10.1016/j.cub.2012.04.065. PubMed PMID: 22683264.

9. Hedden P. The Current Status of Research on Gibberellin Biosynthesis. Plant Cell Physiol. 2020;61(11):1832-49. doi: 10.1093/pcp/pcaa092. PubMed PMID: 32652020; PubMed Central PMCID: PMCPMC7758035.

10. Salazar-Cerezo S, Martínez-Montiel N, García-Sánchez J, Pérez-y-Terrón R, Martínez-Contreras RD. Gibberellin biosynthesis and metabolism: A convergent route for plants, fungi and bacteria. Microbiological research. 2018;208:85-98.

11. Ilyas M, Nisar M, Khan N, Hazrat A, Khan AH, Hayat K, et al. Drought Tolerance Strategies in Plants: A Mechanistic Approach. Journal of Plant Growth Regulation. 2021;40(3):926-44. doi: 10.1007/s00344-020-10174-5.

12. Emongor V. Gibberellic Acid (GA3) Influence on Vegetative Growth, Nodulation and Yield of Cowpea (Vigna unguiculata (L.) Walp. Journal of Agronomy. 2007;6:509-17.

13. Shani E, Hedden P, Sun T-p. Highlights in gibberellin research: A tale of the dwarf and the slender. Plant Physiology. 2024;195(1):111-34. doi: 10.1093/plphys/kiae044.

14. Rizza A, Walia A, Lanquar V, Frommer WB, Jones AM. In vivo gibberellin gradients visualized in rapidly elongating tissues. Nature Plants. 2017;3(10):803-13. doi: 10.1038/s41477-017-0021-9.

15. Binenbaum J, Weinstain R, Shani E. Gibberellin Localization and Transport in Plants. Trends in Plant Science. 2018;23(5):410-21. doi: 10.1016/j.tplants.2018.02.005.

16. Wu K, Xu H, Gao X, Fu X. New insights into gibberellin signaling in regulating plant growth-metabolic coordination. Curr Opin Plant Biol. 2021;63:102074. Epub 20210701. doi: 10.1016/j.pbi.2021.102074. PubMed PMID: 34217918.

17. Erenstein O, Jaleta M, Sonder K, Mottaleb K, Prasanna BM. Global maize production, consumption and trade: trends and R&D implications. Food Security. 2022;14(5):1295-319. doi: 10.1007/s12571-022-01288-7.

18. Stokstad E. High hopes for short corn. Science. 2023;382(6669):364-7. Epub 20231026. doi: 10.1126/science.adl5302. PubMed PMID: 37883569.

19. Paciorek T, Chiapelli BJ, Wang JY, Paciorek M, Yang H, Sant A, et al. Targeted suppression of gibberellin biosynthetic genes ZmGA20ox3 and ZmGA20ox5 produces a short stature maize ideotype. Plant Biotechnol J. 2022;20(6):1140-53. Epub 20220309. doi: 10.1111/pbi.13797. PubMed PMID: 35244326; PubMed Central PMCID: PMCPMC9129074.

20. Su X, Zheng J, Hao Y, Xia R, Xu J. A high-efficiency method for simultaneous quantitation of bioactive gibberellins in Litchi chinensis using UHPLC-QQQ-MS/MS. Scientia Horticulturae. 2024.

21. Zhang J, Zhang X, Chen R, Yang L, Fan K, Liu Y, et al. Generation of Transgene-Free Semidwarf Maize Plants by Gene Editing of Gibberellin-Oxidase20-3 Using CRISPR/Cas9. Front Plant Sci. 2020;11:1048. Epub 20200709. doi: 10.3389/fpls.2020.01048. PubMed PMID: 32742269; PubMed Central PMCID: PMCPMC7365143.

22. Murofushi N, Honda I, Hirasawa R, Yamaguchi I, Takahashi N, Phinney BO. Gibberellins from the Seed, Tassel, Cob and Silk of Maize. Agricultural and Biological Chemistry. 1991;55(2):435-9. doi: 10.1080/00021369.1991.10870589.

23. Yamaguchi I, Nakazawa HK, Nakagawa R, Suzuki Y, Kurogochi S, Murofushi N, et al. Identification and Semi-Quantification of Gibberellins from the Pollen and Anthers of Zea mays by Immunoassay and GC/MS. Plant and Cell Physiology. 1990;31:1063-9.

24. Murofushi N, Honda I, Hirasawa R, Yamaguchi I, Takahashi N, Phinney BO, editors. Gibberellins from the Tassel, Cob, 'Seed,' Silk, and Pollen of Maize1991; New York, NY: Springer New York.

25. Searle SRC, G; McCulloch, C.E. . Variance Components John Wiley & Sons, Inc; 2006.

26. Sahai HA, M. The Analysis of Variance Birkhäuser Boston, MA; 2012.

27. Pan C, Tan, S.N., Yong, J.W.H., Ge, L. Progress and development of analytical methods for gibberellins. Journal of Separation Science. 2017;40:346-60. doi: https://doi.org/10.1002/jssc.201600857.

28. Urbanová T, Tarkowská D, Novák O, Hedden P, Strnad M. Analysis of gibberellins as free acids by ultra performance liquid chromatography–tandem mass spectrometry. Talanta. 2013;112:85-94. doi: https://doi.org/10.1016/j.talanta.2013.03.068.

29. López-Cristoffanini C, Serrat X, Jáuregui O, Nogués S, López-Carbonell M. Phytohormone Profiling Method for Rice: Effects of GA20ox Mutation on the Gibberellin Content of Japonica Rice Varieties. Frontiers in Plant Science. 2019;10. doi: 10.3389/fpls.2019.00733.

30. Fernández H, Doumas P, Bonnet-Masimbert M. Quantification of GA1, GA3, GA4, GA7, GA8, GA9, GA19 and GA20; and GA20 metabolism in dormant and non-dormant beechnuts. Plant Growth Regulation. 1997;22(1):29-35. doi: 10.1023/A:1005814926549.

31. Rood SB, Buzzell RI, Mander LN, Pearce D, Pharis RP. Gibberellins: A Phytohormonal Basis for Heterosis in Maize. Science. 1988;241(4870):1216-8. doi: doi:10.1126/science.241.4870.1216.

32. Fujioka S, Yamane H, Spray CR, Katsumi M, Phinney BO, Gaskin P, et al. The dominant non-gibberellin-responding dwarf mutant (D8) of maize accumulates native gibberellins. Proc Natl Acad Sci U S A. 1988;85(23):9031-5. doi: 10.1073/pnas.85.23.9031. PubMed PMID: 16594001; PubMed Central PMCID: PMCPMC282656.

33. Liu Y, Chen Z, Zhang C, Guo J, Liu Q, Yin Y, et al. Gene editing of ZmGA20ox3 improves plant architecture and drought tolerance in maize. Plant Cell Rep. 2023;43(1):18. Epub 20231226. doi: 10.1007/s00299-023-03090-x. PubMed PMID: 38148416.

34. Chen Z, Liu Y, Yin Y, Liu Q, Li N, Liu X, et al. Development of dwarfish and yield-effective GM maize through passivation of bioactive gibberellin. Transgenic Res. 2019;28(5-6):589-99. Epub 20191008. doi: 10.1007/s11248-019-00172-z. PubMed PMID: 31595387.

35. Constance NW, Proebsting WM, Hedden P, Rivin CJ. Gibberellins and Seed Development in Maize. I. Evidence That Gibberellin/Abscisic Acid Balance Governs Germination versus Maturation Pathways. Plant Physiology. 2000;122(4):1081-8.

Supporting information

S1 Table. Summary of sample type and collection information.

S2 Table. Value assigned to non-detection (ND) data points for statistical analysis

S1 Fig. The representative MRM chromatogram for GA1 and GA4 in the LLOQ (standard 1)

Contributorship

Credit: Yanfei Wang: Conceptualization, Project administration, Resources, Supervision, Writing – review & editing; Xia Guan: Formal analysis, Investigation, Methodology, Writing – original draft, Writing – review & editing; Nitin Supekar: Formal analysis, Investigation, Writing – original draft, Writing – review & editing; Changjian Jiang: Statistical analysis, Data curation, Formal analysis, Methodology, Writing – original draft, Writing – review & editing; Junhong Guo: Methodology, Formal analysis, Investigation, Writing – original draft, Writing – review & editing; Ed Allen: Project administration, Writing – review & editing; Charles Dietrich: Project administration, Writing – review & editing; Cody Postin: Formal analysis, Investigation, Methodology, Writing – original draft, Writing – review & editing; Shubha Shakya: Figure 1 udpate, Writing – review & editing;

Fig 2A

Click here to access/download;Figure;Fig 2A.tif

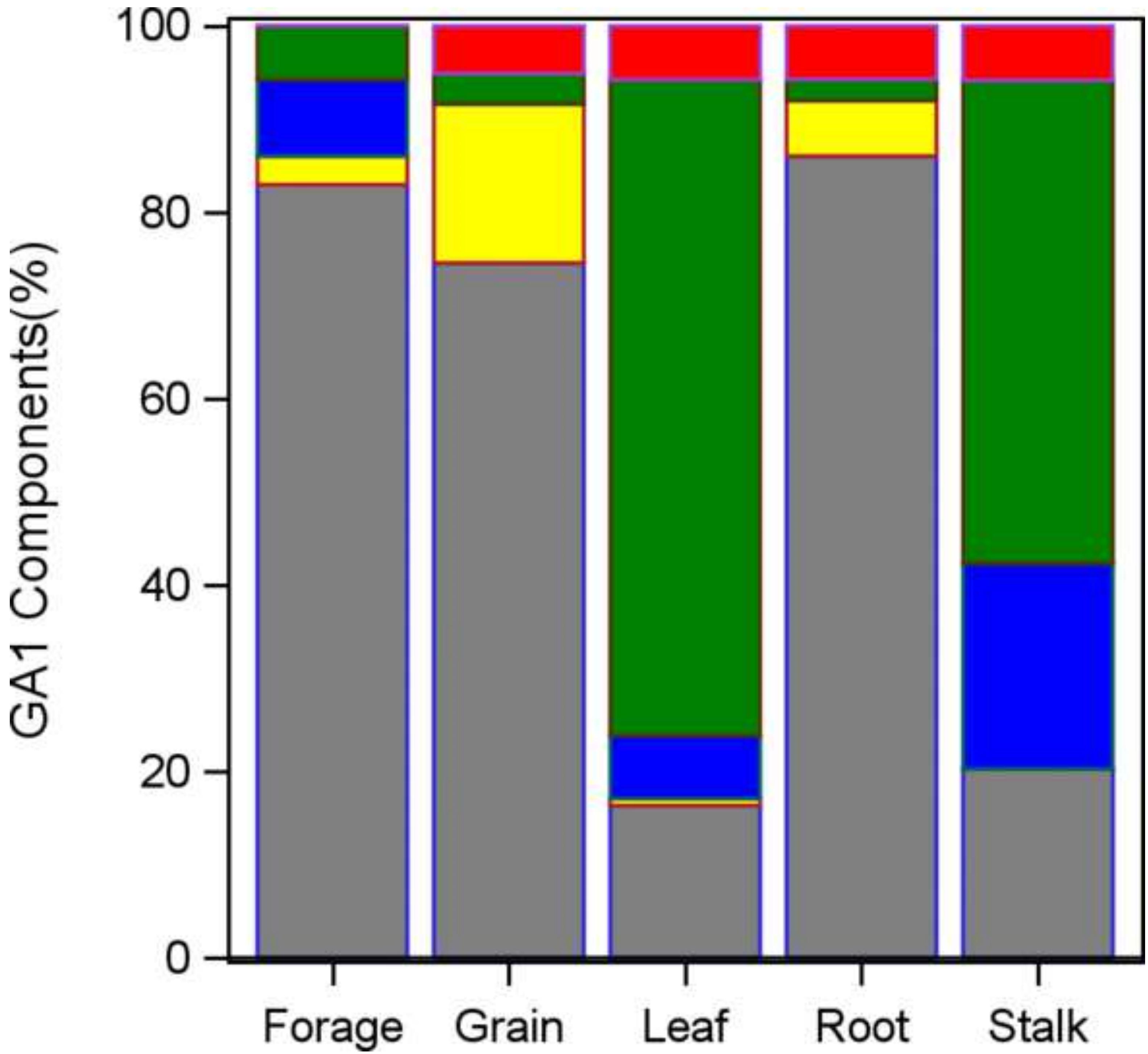

Fig 2B

Click here to access/download;Figure;Fig 2B.tif

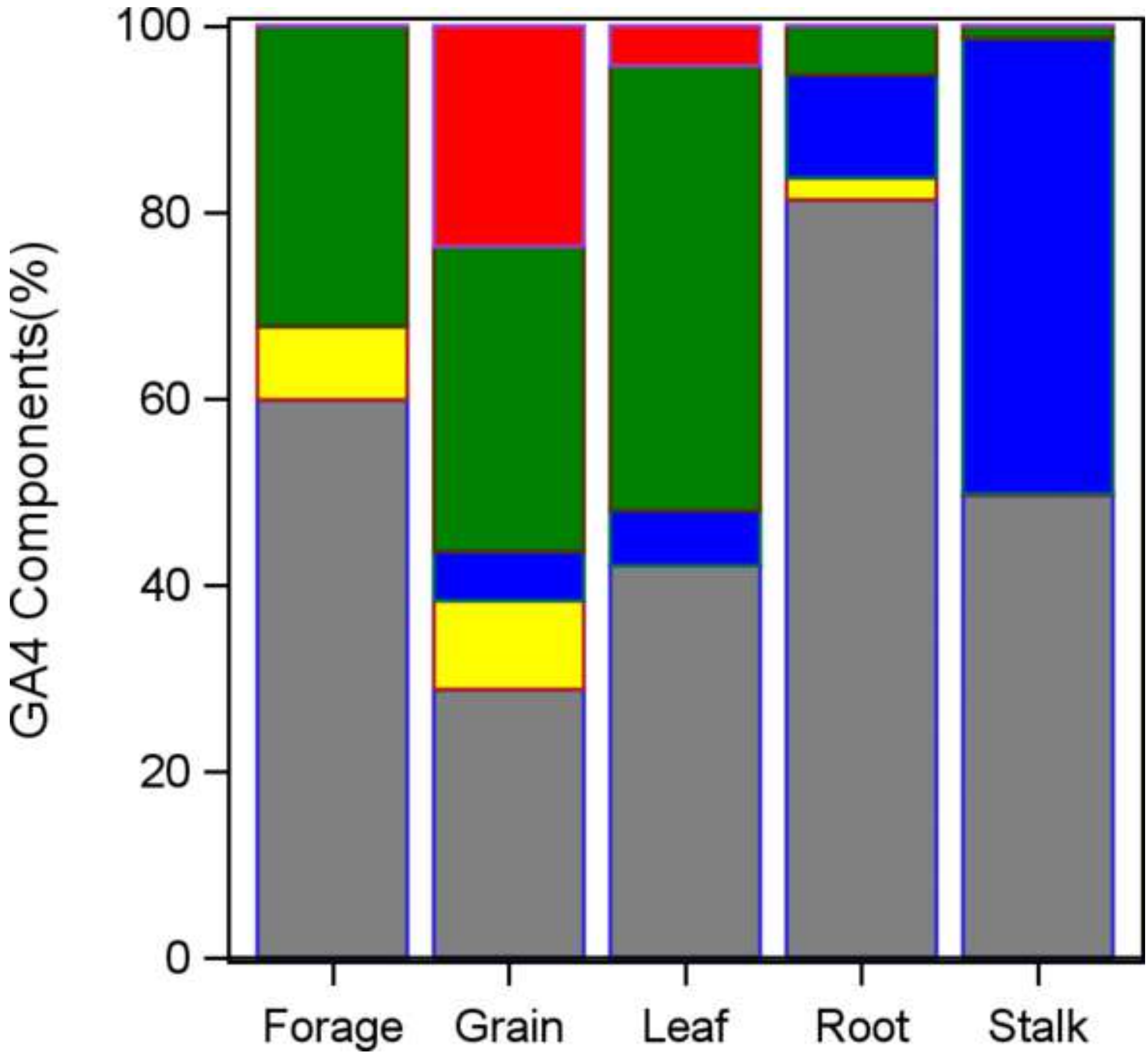

Fig 3

Click here to access/download;Figure;Fig 3.tif

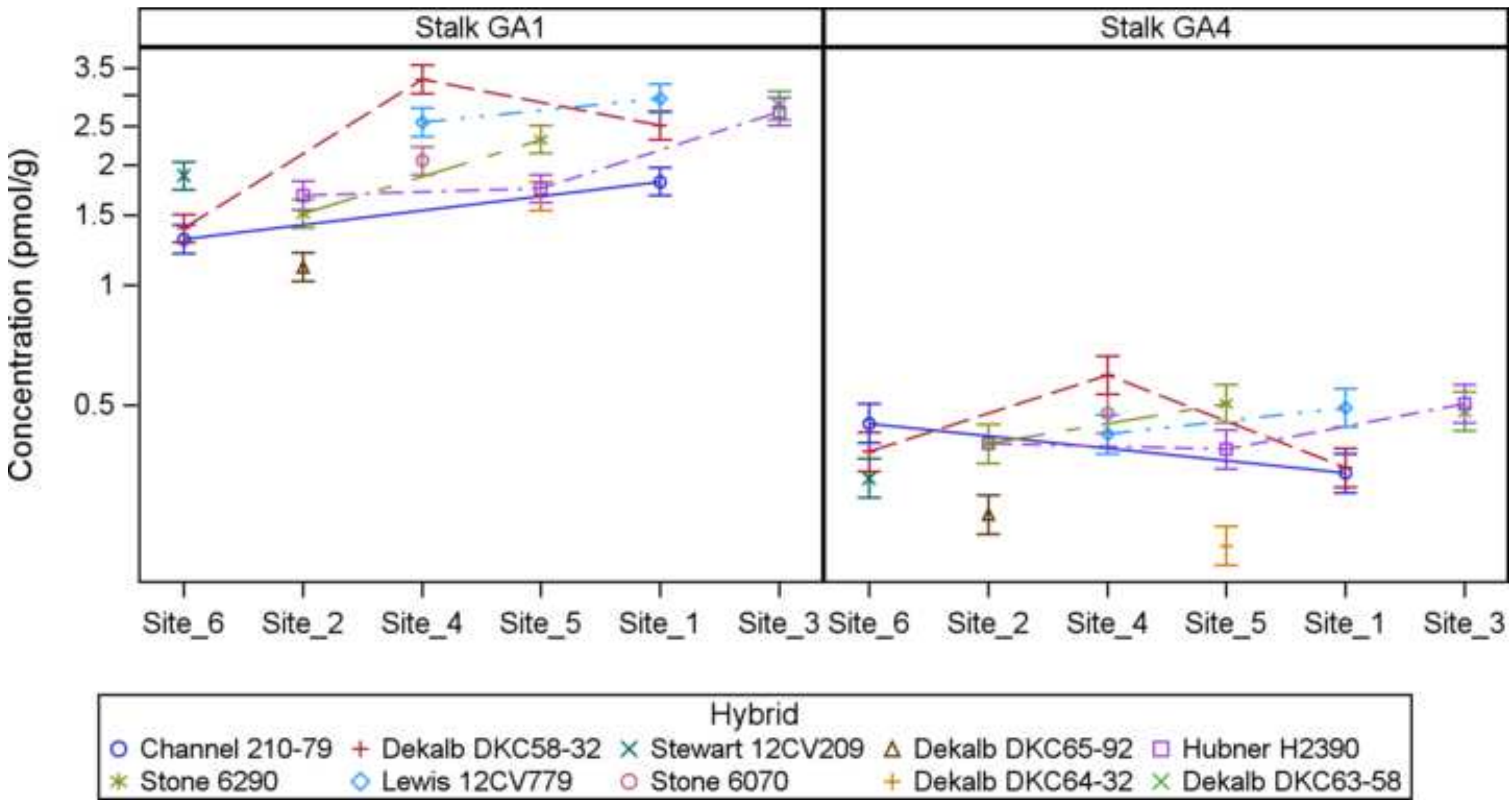

Fig 4

Click here to access/download;Figure;Fig 4.tif

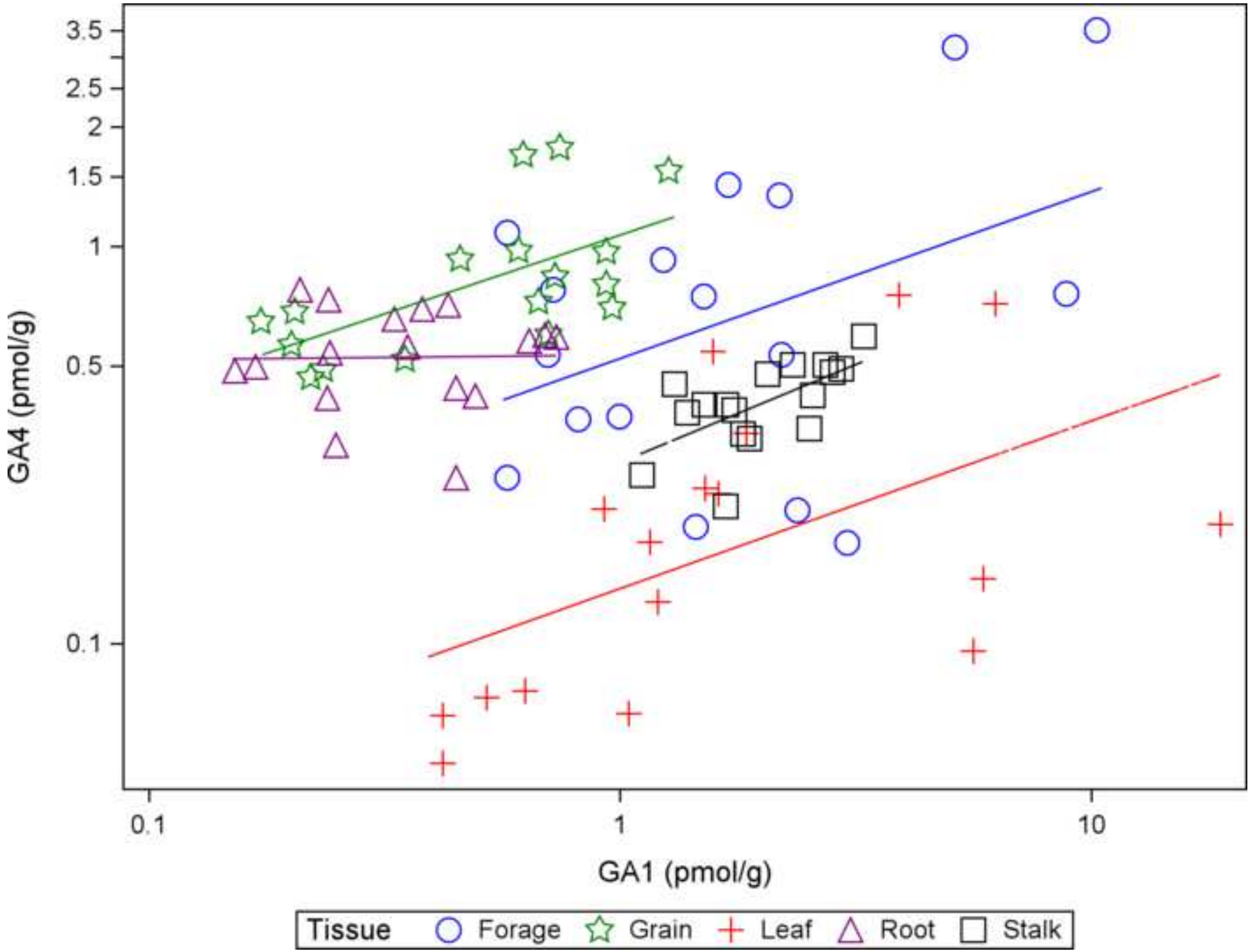

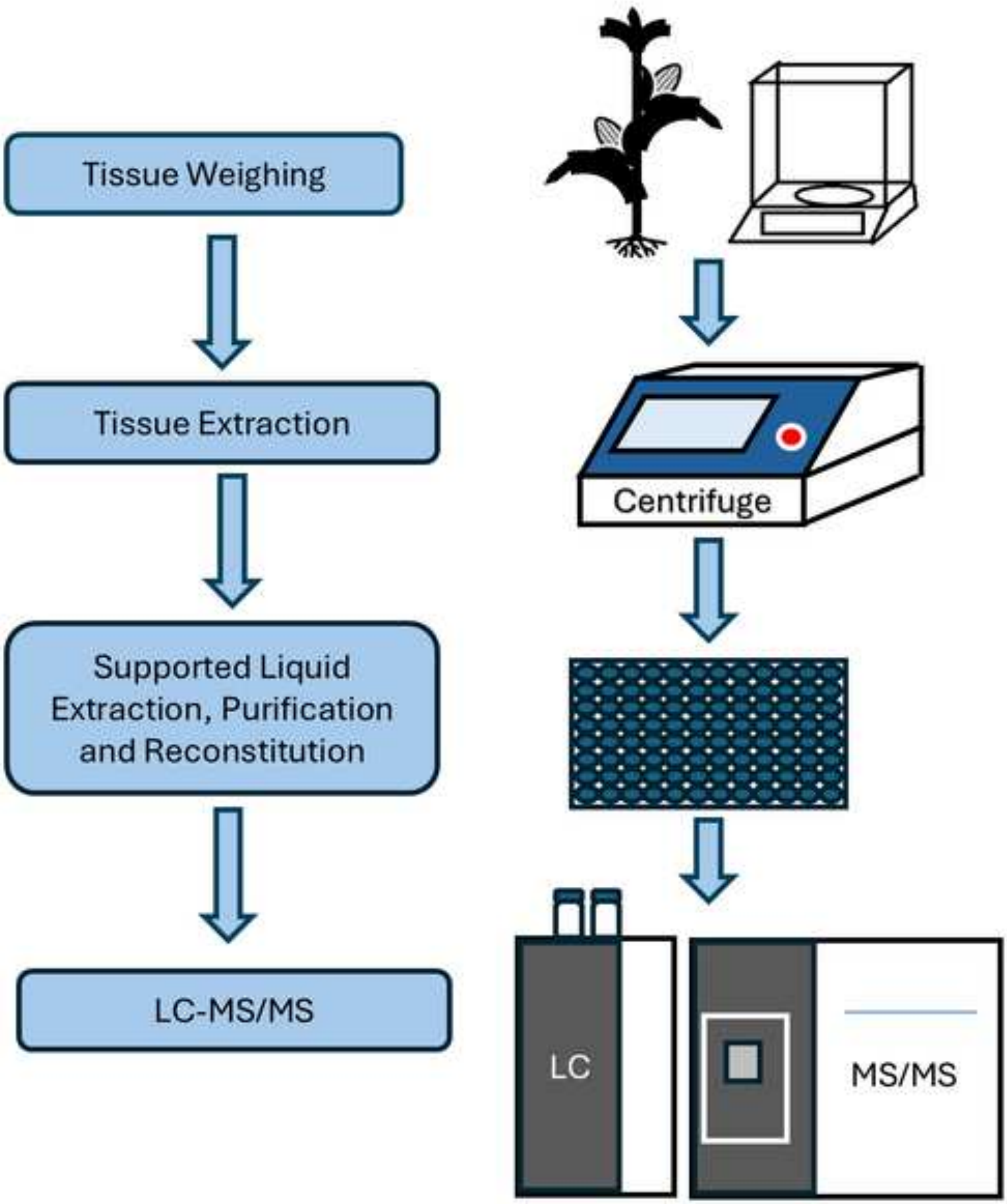
Tissue Weighing
Tissue Extraction
Supported Liquid Extraction, Purification and Reconstitution
LC-MS/MS
Centrifuge
LC
MS/MS

Click here to access/download

Supporting Information

Raw dataset_field sample.xlsx

Click here to access/download
Supporting Information
Validation data.xlsx

S2 Table

Click here to access/download
Supporting Information
S2 Table.docx

Click here to access/download
Supporting Information
S1_Fig.pdf

Click here to access/download
Supporting Information
S1 Table.docx

Click here to access/download
Other
PLOS_Authorship_Changes_Form_(2026)_Signed.pdf

Click here to access/download

Other

PLOS Authorship Changes Form (2026).pdf

Natural variation of bioactive gibberellin levels in maize tissues across North America

Xia Guan, Nitin Supekar, Changjian Jiang, Junhong Guo, Edwards Allen, Charles Dietrich, Cody Postin, Shubha Shakya, Yanfei Wang*

Bayer CropScience LLC., Chesterfield, Missouri, United States of America

*Corresponding Author

E-mail: yanfei.wang1@bayer.com

Keywords: Gibberellin A1 (GA_1), Gibberellin A4 (GA_4), Liquid chromatography-tandem mass spectrometry (LC-MS/MS), natural variation, conventional maize tissues, stalk, leaf

Abstract

Gibberellins (GA) are endogenous phytohormones that regulate growth and development throughout the plant lifecycle. While gibberellins have been extensively studied under controlled conditions for their fundamental role in plant adaptation and agronomic trait development, field-based data on their natural variation of bioactive GA levels across diverse agricultural genotypes and environments remains limited. To address this knowledge gap, we analyzed bioactive gibberellin A1 (GA_1) and gibberellin A4 (GA_4) in multiple tissues from ~~across~~ commercial conventional ~~multiple~~ maize hybrids grown in diverse field environments using high-throughput liquid chromatography-tandem mass spectrometry (LC-MS/MS). This study provides ~~comprehensive~~ quantitative~~ion~~ measurements of endogenous GA_1 and GA_4 levels in maize tissues ~~from diverse commercial hybrids grown~~collected under standard agricultural practices and describes~~, revealing hormonal~~ variation ~~associated with~~ influenced by genetic background and environmental conditions. Our findings demonstrate that GA_1 and GA_4 concentrations from conventional maize samples varied across tissue types, hybrid, and field sites. Analysis of variance components indicated that the site variation was statistically significant in five ~~out~~ of ten gibberellin by tissue combinations, while the genetic component was significant only for GA_4 in grain samples, suggesting ~~indicating~~ that environment factors have a larger effect than genetic factors. Positive correlations between GA_1 and GA_4 were observed consistently across tissues except root. ~~These findings highlight the common metabolic pathways involved in regulating gibberellin levels in maize and provide the first comprehensive dataset of GA_1 and GA_4 variation under real-world agricultural conditions~~. These findings offer preliminary insights into the interplay between genetics and~~,~~ environment~~, and hormonal regulation in maize,~~ across germplasm

and environments that have been previously described.~~, that need to be considered when breeding for traits that impact gibberellin content in maize~~

Introduction

Gibberellins (GA) are endogenous phytohormones that play a crucial role in regulating plant growth and development throughout the plant lifecycle. They were first discovered as secondary metabolites in the fungus ~~*Gibberella fujikuroi*~~ *Fusarium fujikuroi*. [1, 2] Gibberellins comprise a class of diterpenoid carboxylic acids that possess the tetracyclic ent-gibberellane carbon skeletal structure. Among various gibberellins, only a few, e.g., GA_1, GA_3, GA_4 and GA_7 exhibit significant biological activity [3]. GA_1 and GA_4 are the major active gibberellins that promote growth and development in plant species. They regulate and control diverse developmental processes such as seed germination, stem elongation, leaf expansion, and flower and fruit development [2, 4]. Additionally, gibberellins regulate plant adaptation to both biotic and abiotic stresses [5, 6].

The biosynthesis and signaling pathways of gibberellins are intricate systems that exhibit complex spatiotemporal patterns across cells, tissues, and developmental stages [7, 8]. These patterns result from multiple enzymatic steps and feedback mechanisms that respond dynamically to both endogenous and environmental signals [9, 10]. Environmental conditions can influence measured gibberellin pools through effects on biosynthesis, catabolism, conjugation, transport, or signaling ~~Environmental factors such as drought stress can significantly reduce gibberellin biosynthesis across plant species~~ [11], leading to stunted growth and altered development, while optimal conditions enhance gibberellin production and promote vigorous growth [12]. The movement of gibberellins between cells and across plant organs, coupled with their variable distribution patterns, creates challenges for understanding hormone-environment interactions in agricultural settings [13-15].

Formatted: Font: (Default) Times New Roman, Italic

Due to the vital biological activity of gibberellins in plant growth and development, several applications have been developed with commercial and biotechnological utilization: exogenous applications of gibberellins to promote growth, and exogenous applications of its concentration or the genetic modification of biosynthesis to optimize plant phenotypes and environmental responses [10]. Research in recent years has shown that gibberellins have direct and indirect effects on the regulation of many plant traits. Many plant varieties with improved agronomic traits (e.g., dwarf phenotypes or increased biomass) are shown to be related to gibberellin activity and signaling [16], thus manipulation of gibberellin levels is commonly used in agricultural practices to optimize plant growth and yield.

Maize (*Zea mays* L.; ~~C~~corn) is a major agricultural commodity, contributing significantly to the economy of many countries and serves as a staple food for many populations worldwide [17]. Short maize hybrids have been created through conventional breeding by researchers with the objectives to improve performance under high density and improve lodging tolerance [18]. However, breeding short traits into existing maize hybrids is time consuming, challenging, and can affect environmental responses. Genetically modified maize can be engineered through the enhancement or suppression of hormone pathways, including gibberellin biosynthesis. Scientists have developed a genetically modified short stature maize ideotype by suppressing maize genes that regulate the levels of gibberellins, reducing plant stature without undermining maize yields [19].

Accurate quantification of endogenous GA levels is ~~useful~~ valuable for describing variation across tissues, genotypes, and environments and for providing baseline context for future studies ~~Accurate quantification of gibberellins in plant tissues has been instrumental in understanding gibberellin-mediated mechanisms and effectively improving their practical applications~~ [20].

While previous studies have quantified bioactive gibberellins in maize tissues [8, 19, 21-24], these analyses were typically limited to specific varieties or locations, lacking the scope needed to characterize natural variation across diverse agricultural conditions. However, Eestablishing baseline patterns of endogenous gibberellin variation abundance in agricultural settings can there support subsequent research is crucial for several reasons. First, understanding the natural variability of gibberellin concentrations in conventional maize tissues - a primary determinant of plant stature – and distinguishing between environmentally-induced and genetically-controlled variation provides essential context for crop improvement efforts. This baseline knowledge is particularly valuable for developing and evaluating new short-stature varieties, where gibberellin-related genetic modifications must be assessed against the background of natural gibberellin fluctuations variablity that occurs across diverse field environments.

Second, while gibberellin biology has been extensively studied under controlled conditions [19], quantifying the magnitude of gibberellin variation in commercial hybrids under real-world agricultural conditions helps bridge the gap between laboratory findings and field performance. Such information may provide context for future studies evaluating This knowledge is critical for predicting the stability and effectiveness of gibberellin-modified traits across different growing environments, where environmental stresses can significantly impact gibberellin-mediated growth processes and ultimately affect yield potential. Understanding the relative contributions of genetic background and environmental factors to gibberellin concentrations in maize tissue can inform breeding strategies and help develop more resilient short-stature varieties that maintain desired height characteristics across diverse growing conditions.

Understanding the natural range and stability of endogenous gibberellin levels in conventional hybrid maize across diverse environments may becomes essential forhelp contextualizeing future

studies of ~~the~~ gibberellin related traits changes in new hybrids and predicting their performance across different agricultural regions. ~~Such comprehensive baseline data can inform both the development and deployment of gibberellin modified hybrids while providing insights into the inherent plasticity of gibberellin regulation in field conditions.~~ The purpose of this study is to quantify major bioactive endogenous GA_1 and GA_4 in maize tissues by the validated LC-MS/MS method across conventional maize hybrids, tissue types, and environments, which could provide a baseline understanding of the contribution of genetics and environment to the variation of gibberellin content. ~~to suggest potential targets for breeding programs aimed at improving agronomic traits such as yield.~~

Materials and methods

Maize field trials

Maize samples from ten commercially available conventional maize hybrids were collected from field trials during the 2023 growing season. Six field trial locations were spread across states representing major maize-growing regions of the United States (Iowa, Illinois, Indiana, Missouri, Nebraska, and Ohio) (Table 1). Plant materials consisted of conventional crop varieties grown in experimental plots that were collocated with USDA permitted materials under Authorization No. AUTH0000233243.

Each site was planted in a randomized complete block design with four replicated blocks. Hybrids listed in Table 1 were included in each of the four replicated blocks where they were planted, in addition to other hybrids not associated with this study. Within a block, each hybrid was planted in an approximately 6 m × 6 m plot. Each site received a unique randomization scheme. Blocks at

each site were arranged to limit variability in environmental conditions such as soil type, slope, and previous cropping history. Normal agronomic practices were followed for each site. Trial maintenance (fertilization, pesticide use, tillage practices, irrigation, etc.) varied across sites, but all maintenance activities were applied uniformly within a site. Maize samples were collected throughout the growing season and included the following tissues: leaf, root, stalk, pollen, forage, and grain. The same tissues collected from each plot were pooled into a single sample. -Tissue samples were non-systematically selected with consistent field sampling criteria: ~~, aside from avoiding~~ plot ends were avoided, and ~~ensuring sampled~~ plants were chosen to be~~are~~ competitive and representative of the plot as a whole. The selected tissue samples from each plot were combined to generate one pooled sample per plot, which served as the observational unit for downstream analysis. This pooling approach was applied consistently across all sites. Sampling details, ~~including sample amounts and growth stage timing,~~ can be found in Table S1. Specifically, it specifies the tissue-specific sampling amount used to generate one pooled sample per lot: 60 leaf sections, 8 roots, 4 stalks, 10 mL pollen, 1 forage plant, or 1 kg grain, depending on tissue type, along with the corresponding number of sampled plants and growth stages. All samples were kept on dry ice immediately after collection until storage in a -80ºC freezer prior to analysis, with the exception of grain samples which were kept at ambient temperature.

Table 1. Field trial locations and maize hybrid names

Site Code	County, State	Hybrid Name
Site 1	Jefferson County, Iowa	Dekalb DKC58-32
		Lewis 12CV779
		Channel 210-79
Site 2	Shelby County, Illinois	Stone 6290
		Hubner H2390
		Dekalb DKC65-92
Site 3	Boone County, Indiana	Dekalb DKC63-58
		Hubner H2390
Site 4	Adair County, Missouri	Dekalb DKC58-32

		Lewis 12CV779
		Stone 6070
Site 5	York County, Nebraska	Stone 6290
		Hubner H2390
		Dekalb DKC64-32
Site 6	Miami County, Ohio	Dekalb DKC58-32
		Stewart 12CV209
		Channel 210-79

Reagents

Gibberellin standards were used as reference standards for the analytical procedures or calibration of equipment. Stable-isotope-labelled gibberellin standards were used as internal standards (synthesized by OlChemlm). GA_1 was synthesized by Toronto Research Chemicals (purity: 97%) and GA_4 was synthesized by Thermo Scientific (purity: 90.30%). Formic acid (purity: 99%), ethyl acetate (HPLC grade) and methanol (purity: ≥99.8%) were purchased from Fisher Scientific and acetic acid (analytical grade) was obtained from Millipore, AR.

Sample preparation

After cryomilling maize tissues, 50 ± 5 mg of fresh frozen sample was weighed into a pre-chilled 1.5 mL glass tube placed on dry ice. Next, on wet ice, 10 µL of internal standard working solutions (100 ng/mL of GA_1-D4 and 25 ng/mL of GA_4-D2) and 1.00 mL of pre-chilled extraction buffer (80% methanol with 0.5% formic acid) were added to each sample. The samples, arranged in a 96-well formatted rack, were manually agitated 40 times to thoroughly suspend the tissue in the extraction buffer, followed by an overnight incubation (approximately 15 hours) at 4°C with gentle mixing at 50 rpm on a Fisher brand Nutating Mixter (Fisher Scientific, 88-861-043). After incubation, the samples were centrifuged at 2000 rpm for 10 minutes at 4°C. The supernatant was transferred to a 96-well polypropylene sample collection plate. The pellets were re-extracted by

adding another 1 mL of extraction buffer following the same procedure as above. The combined supernatants evaporated under nitrogen flow at 35°C using a microplate sample evaporation system (Biotage SPE Dry 96, SD-9600-DHS-NA), until about 400 ~~mL~~ µL of aqueous phase was left. Subsequently, the extracts were loaded into a Biotage 96-well supported liquid extraction (SLE) plate (Biotage, 8200400P01), which was mounted on a glass-coated 96-well collection plate. The analyte gibberellins were eluted with 2 × 0.9 mL of ethyl acetate. The eluate was then evaporated to dryness under nitrogen by using the SPE Dry 96. The dried samples were reconstituted in 100 µL of reconstitution buffer (20% Methanol with 0.1% formic acid), then vortexed at 1200 rpm at room temperature for 3 minutes. Following this, the samples were centrifuged at 2000 rpm for 3 minutes at 4°C, and 90 µL of the supernatant was transferred into a 96-well plate with glass inserts, kept on wet ice prior to LC-MS/MS analysis.

LC-MS/MS analysis of gibberellins in maize tissues

Analytical instruments include mass spectrometer (Sciex triple Quad 7500), HPLC system (ExionLC 20A system) and data acquisition system (PC-based workstation with AB ~~Sxiex~~ Sciex OS software). Reconstituted samples were separated on a 1.7 µm Kinetex 100 Å C18 LC column (100 mm × 2.1 mm) (Phenomenex, 00D4475AN) at a flow rate of 0.40 mL/min and a column oven of 40˚C. Mobile phase A is 0.5% acetic acid in water and mobile phase B is 0.5% acetic acid in methanol. The LC gradient is ~~as below :~~presented in Table 2. ~~mobile phase B (%) changes from 26 to 55 from 5.30 to 5.40 min, 55 to 58 from 5.40 to 10.20 min, 58 to 80 from 10.20 to 10.30 min, 80 to 81 from 10.30 to 12.70, 81 to 98 from 12.70 to 12.80, B (%) stays at 98 to 14.00 min, then drops from 98 to 26 from 14.00 to 14.10; finally, the column was equilibrated from 14.10 to 16 min to its initial conditions of 26% of B.~~ Mass spectrometric conditions are as follows: polarity mode, negative ionization; scan type, MRM; spray voltage, -1800 V; ion source gas 1, 40 psi; ion

source gas 2, 45 psi; source temperature, 500°C; curtain gas, 40; CAD gas, 10; entrance potential (EP), -10 V. The multiple reaction monitoring (MRM) transitions for each analyte and its stable isotope-labeled internal standard (IS) can be found in Table ~~2~~3 with respective optimized collision energy (CE) and collision cell exit potential (CXP). The representative MRM chromatogram of GA_1 and GA_4 in the LLOQ sample (standard 1) was provided as Supporting Information S1 Fig.

Table 2. LC gradient

Time (min)	Flow (mL/min)	B.Conc (%)
0	0.40	26
5.30	0.40	26
5.40	0.40	55
10.20	0.40	58
10.30	0.40	80
12.70	0.40	81
12.80	0.40	98
14.00	0.40	98
14.10	0.40	26
16.00	0.40	26

Table ~~2~~3. MRM transition parameters

Compound ID	Q1 mass (Da)	Q3 mass (Da)	Dwell (ms)	CE(V)	CXP(V)
GA_1	347.19	273.101	100	-32	-8
GA_4	331.19	213.131	100	-40	-7
GA_1-D4 (IS)	351.19	277.069	100	-36	-9
GA_4-D2 (IS)	333.14	259.064	100	-33	-9

Formatted: Font: (Default) +Body (Aptos), No underline, Font color: Auto

Formatted: Font: (Default) +Body (Aptos), No underline, Font color: Auto, Subscript

Formatted: Font: (Default) +Body (Aptos), No underline, Font color: Auto

Formatted: Font: (Default) +Body (Aptos), No underline, Font color: Auto, Subscript

Formatted: Font: (Default) +Body (Aptos), No underline, Font color: Auto

Formatted: Font: (Default) +Body (Aptos)

Formatted: Font: (Default) +Body (Aptos)

Formatted: Space After: 0 pt

Formatted: Space After: 0 pt

Formatted: Space After: 0 pt

Formatted: Space After: 0 pt

Formatted: Space After: 0 pt

Formatted: Space After: 0 pt

Formatted: Space After: 0 pt

Formatted: Space After: 0 pt

Formatted: Space After: 0 pt

Formatted: Space After: 0 pt

Compound ID	Retention time (minutes)	Q1 mass (Da)	Q3 mass (Da)	Dwell (ms)	CE(V)	CXP(V)
GA_1	4.63	347.19	273.10	100	-32	-8
GA_4	9.37	331.19	213.13	100	-40	-7
GA_1-D4 (IS)	4.48	351.19	277.07	100	-36	-9
GA_4-D2 (IS)	9.34	333.14	259.06	100	-33	-9

Formatted: Justified, Line spacing: Double
Formatted Table
Formatted: Justified, Line spacing: Double
Formatted: Justified, Line spacing: Double
Formatted: Justified, Line spacing: Double
Formatted: Justified, Line spacing: Double

MS data analysis

Raw data files were analyzed using Analytics in SCIEX OS software. A standard curve for each endogenous gibberellin was generated as the ratio of the gibberellin standard's response (peak area) to the stable-isotope-labelled standard response (peak area), plotted against the concentrations of the gibberellin standard. A linear regression model was used for the quantification of the endogenous gibberellin levels with $1/x^2$ weighting. The final concentration of gibberellins in the tissues was reported in pmol/g.

Variance component analysis to compare genetic and environmental variations

To compare genetic and environmental contributions to GA1 and GA4 expression across five tissues, we first examined the distribution of the response variables. Visual inspection of the observed response and residual plots from the analysis indicated a log-normal distribution, supporting the use of log-transformed data in subsequent analyses. Then, a variance component analysis [25] was performed with the following model (1) and a log transformation $y = \log(x)$ for the normality assumption of the analysis, where is the observed response. .

$$y_{ijk} = \mu + s_i + r_{j(i)} + m_k + (sm)_{ik} + \varepsilon_{ijk} \qquad (1)$$

where y_{ijk} is the observed response of each plot after transformation for the kth hybrid from the jth replicate (i.e. block) at the ith site, μ is the overall mean, s_i is the effect of the ith site, $r_{j(i)}$ is the effect of the jth ~~block~~ replicate within the ith site, m_k is the effect of the kth ~~material~~hybrid, $(sm)_{ik}$ is the interaction effect of the kth hybrid with the ith site, ε_{ijk} is the residual. The term replicate and block will be used interchangeably in the following. Before fitting the model, Levene's tests were conducted to examine the assumption of the variance homogeneity, and non-significance was found for effects of sites, materials, and interactions on the variance. ~~After fitting the model, the residual plots were examined for the normality assumption of the distribution. Since the purpose of the analysis was to estimate focus on the variance components, all effects were assumed to be random in all three following analyses (estimating variance components, significance testing, and examining hybrid mean changes over sites) each performed by a different SAS procedure (each procedure applysing an algorithm for better serving the specific purpose).~~ Different SAS procedures were used to estimate variance components, test significance, and examine changes in hybrid means across sites, with each procedure chosen to leverage its specific analytical strengths. ~~and SAS procedure PROC MIXED was applied for unbiased estimates of the variance components (with the restriction of nonnegative estimate). For statistical significance of each component, PROC GLIMMIX with option COVTEST was applied for significant testing using a likelihood-based test due to the zero boundary of variance components (which compares the likelihood of the model with and without the effect of the hypothesis using a mixed chi-square distribution for the variance on zero boundaryrequires a mixed chi-square distribution to determine the critical value of the test) at the 5%, 1%, and 0.1% significance levels without correcting the multiple tests. When the significance was confirmed, further investigation was made for the mean changes of individual~~

Formatted: Line spacing: Double

Formatted: Font: Not Italic

hybrid across sites to demonstrate how the hybrid effects are connected across sites for justifying the separation of all effects in the model (1) especially the interaction (as explained further in discussion) despite of the unbalanced design of the experiment. In this case, the least square means were predicted by using SAS procedure PROC GLM, with the same model (1). Notice that three Three SAS procedures are expected to provide similar results under a balanced design and all components greater than zero. Differences may be present under an unbalanced design as in this investigation., For example, and PROC GLIMMIX is the best for significant testing due to the likelihood-based method (which is the ultimate standard for all statistical analyses), PROC MIXED supposes to be unbiased for the estimate of variance component because of used the restricted maximum likelihood and nonnegative estimates of the variance components are guaranteed. While PROC GLM a negative estimate of the variance is possible, PROC GLM can provided mean predictions of random effects by the method of moment (i.e. the expected mean square or EMS). To estimate variance components, Hybrid, Site, Replicate within Site, and the Hybrid-by-Site interaction were all treated as random effects, with the residual term representing error variance. Random effects were assumed to be independently and identically distributed within each classification, each associated with its own variance component. Variance was partitioned into these additive components using SAS PROC MIXED with REML (Restricted Maximum Likelihood) estimation, a well-established approach for variance component estimation in unbalanced trial designs. The significance of variance components was then evaluated using PROC GLIMMIX with the COVTEST option via likelihood ratio tests. Because the null hypothesis places a variance component on the boundary of the parameter space at zero, the resulting test statistic follows a mixture chi-square distribution. Significance was evaluated at the 5%, 1%, and 0.1% levels without multiplicity correction. For variance components that were

found to be significant, additional analyses were performed to examine mean changes for individual hybrids across sites, thereby illustrating the extent to which hybrid effects were connected across sites and ~~supporting the separation of effects in the model.~~characterizing site-specific hybrid patterns. These analyses were conducted within a linear mixed model framework using PROC MIXED. Hybrid, Site, and the Hybrid-by-Site interaction were treated as fixed effects, whereas Replicate nested within Site was modeled as a random effect. Treating Hybrid and Site as fixed effects produced local LS-means for ~~accurately~~ characterizing ~~unique~~ ~~h~~Hybrid-by-S~~s~~ite patterns, while modeling nested replications as random prevented estimates from being biased by local block-level anomalies. All analyses were performed separately for each gibberellin and ~~each~~ tissue combinations, and results were presented in the following sections.

For t~~T~~he assumptions of the analysis, in addition to the composition of ~~statistical analyses fit~~the model (1) as described above, ~~separately for GA_1 and GA_4.~~distributional assumptions were further examined by the ~~R~~residual plots from PROC MIXED which showed no evidence of violation of normality (after the log transformation on the original observed response) and variance heterogeneity (as an additional visual confirmation to the above Levene's test). The unbiasedness of the estimated variance components and the reliability of the significance were supported by the corresponding algorithm of each SAS procedure despite of the unbalanced nature of the data. ~~Since the purpose of the analysis was to estimate the variation, all effects in model (1) were assumed to be random. The significance of each component was tested by the chi-square test with PROC GLIMMIX option COVTEST at the 5% significance level which accounts for the unbalanced design of the hybrid across sites (as explained further in discussion).~~. Overall, the use of SAS procedures with REML estimation was appropriate for variance component estimation,

significance testing, and LS-means estimation, since these procedures are well suited to handling unbalanced data within a properly specified mixed-model framework.

Results

Method development and validation of gibberellins by LC-MS/MS

Plant tissue extracts contain complex mixtures, and typically gibberellins are present in trace amounts. Therefore, it is necessary to apply both a consistent sample preparation method to reliably extract gibberellins from plant tissues and a sensitive technique to accurately quantify them.

In this study, a robust, reliable, and high-throughput method was developed for the simultaneous quantitation of gibberellins in six maize tissue types (grain, pollen, leaf, root, forage, and stalk). In brief, the gibberellins were extracted from approximately 50 mg of sample, purified by SLE, and separated and quantified by LC-MS/MS using the isotope-labeled internal standard (IS) as shown in Fig 1. The method was validated by determining the linearity ($R^2 \geq 0.98$) over the concentration range of 20-30,000 pg/mL in six tissue types. 20 pg/mL is the LLOQ (lower limit of quantification). Precision of the method was obtained with intra-and inter-day relative standards deviations (≤17.13%) and accuracy for compounds typically ranging between 79-124%. The recoveries of our method ranged from 77.6% to 119.8% in six maize tissues (Table 34). The bench-top stability of the tissue extraction supernatant was evaluated at room temperature for each type of tissue, demonstrating stability for a duration of up to two hours for all tissues except pollen, which exhibited stability of only one hour. Additionally, the stability of the processed samples in the autosampler was assessed at a temperature of 4 °C, with all tissue samples maintaining stability for up to six days.

Fig 1. Workflow for Sample Preparation and Analysis of Gibberellins in Maize Tissues Left Panel: The workflow consists of 4 key steps: (1) sample preparation via cryomilling and weighing, (2) extraction of gibberellins with internal standards (IS) and extraction buffer, (3) SLE analyte purification through centrifugation~~-~~, supernatant collection, and reconstitution of the analyte, and (4) LC-MS/MS analysis for quantitation.

Right Panel: Equipment and components used in the workflow include a balance for weighing samples, a centrifuge for supernatant separation, a SLE plate for sample purification, and an LC-MS/MS system for gibberellin quantification.

Table ~~3~~4. Accuracy, precision, extraction efficiency and recovery ~~**for six maize tissues**~~

Tissue type	Analytes	Accuracy (%)		Precision (CV%)		Extraction Efficiency (%)	Recovery (%)
		Intra-assay	Inter-assay	Intra-assay	Inter-assay		
**Stalk**	GA_1	83.92-114.86	95.78-100.71	0.56-11.5	1.80-14.52	94.56	96.56
	GA_4	87.69-113.10	90.62-105.81	0.42-11.69	2.51-9.33	91.52	115.72
**Grain**	GA_1	79.73-115.95	89.38-106.18	0.49-12.39	1.65-17.13	95.91	92.01
	GA_4	85.83-110.13	86.33-108.89	0.99-12.52	1.48-11.78	93.02	77.59
**Leaf**	GA_1	89.03-115.97	98.55-107.07	0.32-11.38	2.21-15.88	93.67	82.59
	GA_4	82.38-108.36	90.10-105.68	0.53-12.52	2.16-10.61	91.39	82.33
**Root**	GA_1	83.92-114.86	95.78-104.13	0.24-11.50	1.80-14.52	96.51	98.52
	GA_4	85.23-108.36	90.62-103.43	0.42-11.69	2.51-9.43	91.08	107.03
**Forage**	GA_1	83.92-123.06	92.36-110.33	0.32-9.13	2.81-13.98	94.44	93.40
	GA_4	87.69-108.95	90.62-103.43	0.42-11.69	2.51-9.33	91.71	101.89
**Pollen**	GA_1	79.79-115.95	95.18-105.44	0.32-11.38	2.21-14.28	94.78	86.95
	GA_4	81.63-110.13	88.31-108.89	0.99-12.52	1.48-11.78	91.70	76.89

Formatted Table

Quantification of gibberellins in different maize tissue samples

The gibberellins in maize samples were extracted, purified, separated and quantified by LC-MS/MS. The short stability window of pollen tissue limited its suitability for large-scale sample handling; therefore, pollen data were not reported in this study. In total, 68 individual samples of each of the five other tissue types were analyzed and reported. Data with non-detection (ND) values were assigned values by replacing ND with half of the observed minimums for that tissue in order to apply the statistical analysis. The details regarding values given to ND samples are included in Table S2. Gibberellin concentrations in maize tissues were calculated based on the standard curve and the statistics were summarized in Table 45.

Table 45. Summary of GA_1 and GA_4 concentration in different maize tissues

**Analyte**	**Tissue**	**Mean (pmol/g)**	**Standard Error**	**Min (pmol/g)**	**Max (pmol/g)**
**GA_1**	Forage	7.9	2.61	0.4	131.1
**GA_1**	Grain	1.4	0.43	0.1	26.0
**GA_1**	Leaf	3.2	0.57	0.4	27.0
**GA_1**	Root	0.5	0.04	0.1	1.3
**GA_1**	Stalk	2.1	0.08	0.9	3.4
**GA_4**	Forage	1.9	0.56	0.1	29.2
**GA_4**	Grain	0.9	0.06	0.3	2.0
**GA_4**	Leaf	0.3	0.03	0.1	1.4
**GA_4**	Root	0.6	0.03	0.1	1.8
**GA_4**	Stalk	0.4	0.02	0.2	0.9

Variance components in five tissues

Variance components describe how different sources of variation contribute to observed differences in gibberellin concentrations. Estimates of variance components for five tissues are

listed in Table ~~5~~6. The model variance was calculated as the sum of all components except the residual.

Table ~~5~~6. Estimated variance components in log scale and the statistical significance from each source.

		Variance	Variance Components (log)[1]				
Analyte	Tissue	(Model-log)	Varg	Vars	Vargxs	Varr	Vare
GA_1	Forage	0.39	0.00	0.13	0.19	0.07	1.88
GA_1	Grain	0.41	0.08	0.05	0.00	0.28	1.21
GA_1	Leaf	1.29	0.09	1.09***	0.10	0.01	0.25
GA_1	Root	0.11	0.04	0.02	0.00	0.05	0.67
GA_1	Stalk	0.10	0.01	0.07*	0.03**	0.00	0.03
GA_4	Forage	0.73	0.00	0.58*	0.00	0.14	1.09
GA_4	Grain	0.19	0.06*	0.09*	0.01	0.02	0.07
GA_4	Leaf	0.65	0.05	0.54**	0.07	0.00	0.47
GA_4	Root	0.05	0.00	0.01	0.03	0.01	0.20
GA_4	Stalk	0.05	0.00	0.00	0.05*	0.00	0.05

[1] hybrid (Varg), site (Vars), hybrid by site interaction (Vargxs), replicate (Varr), and residual (Vare). Variance (Model) is the sum of all components except Vare.

* indicates statistical significance at the 5% level, ** for significance at the 1% level, and *** at the 0.1% level.

The relative contribution of variance components (i.e., the percentage of each source to total variance) is presented in Fig 2. As shown in Table ~~5~~6 and Fig 2, the residual variation (Vare) is greater than the model-explained variation in five out of ten gibberellin–tissue combinations. Because the variance components in Table ~~5~~6 and Fig 2 were estimated using an all-random-effects model, statistical significance should be interpreted as evidence that these sources of variation are

likely to contribute under repeated sampling of similar sites and hybrids. ~~Despite the multiple testing (i.e. 40 in total including site, hybrid, interaction, and replicate) and possible false positive, the relative contributions of different sources to the overall variation likely persist especially for those significant at 1% and 0.1% levels.~~ While conducting 40 statistical tests increases the risk of false positives, effects supported by both substantial variance contributions and highly significant p-values ($p<0.01$ and $p<0.001$) provide strong evidence of meaningful structure in the data rather than being random noise. Concentrations of gibberellin A1 (GA_1) and gibberellin A4 (GA_4) (mean ± 1 SE) in leaf and stalk were measured and plotted for 10 maize hybrids across six field sites, which are presented in Fig 3.

Fig 2. Variance Component Analysis of Genetic and Environmental Contributions to GA_1 and GA_4 Concentrations. The figure presents the variance component analysis of gibberellin A1 (GA_1) and gibberellin A4 (GA_4) concentrations across different tissues in maize, broken down into contributing sources of variation. Bar charts show the percentage contributions of variance components: Varg (genetic variance, red), Vars (site variance, green), Vargxs (genetic-by-site interaction variance, blue), Varr (replicate variance, yellow), and Vare (residual variance, gray). (A) Variance components for GA_1. (B) Variance components for GA_4.

Formatted: Font color: Text 1

Fig 3. GA_1 and GA_4 Concentrations in Leaf and Stalk Across Hybrids and Sites Concentrations of gibberellin A1 (GA_1) and gibberellin A4 (GA_4) (mean ± SE) in leaf and stalk were measured and plotted for 10 maize hybrids across six field sites. Data are presented in pmol/g, with hybrids represented by different markers and colors, and site-wise trends connected by lines for visualization **Panels:** Top Left: Leaf GA_1 concentration, Top Right: Leaf GA_4 concentration, Bottom Left: Stalk GA_1 concentration, Bottom Right: Stalk GA_4 concentration

Correlation between GA1 and GA4 across tissues

Biosynthetic pathways for GA1 and GA4 share a common upstream pathway, and the subsequent parallel branches utilize the same families of enzymes (e.g., GA20ox, GA3ox) [10]. From this perspective, the correlation between GA_1 and GA_4 was investigated using ~~the~~ biplots. The plot is based on the hybrid mean at each site as shown in Fig 4. In this plot, each mark represents the predicted hybrid mean over four replicates in log and then back transformed into pmol/g; five tissues are represented by different markers and colors. As shown in Fig 4, positive correlations were observed in four out of five tissues except root. The position and range of each tissue are represented by the differences in means (see Table ~~4~~5) and the variation (see Table ~~5~~6) among tissues. Since the hybrid mean at each site in Fig 4 consists of both the hybrid and site effects on the expressions, these positive correlations are due to similar trends in hybrid mean changes over sites between two gibberellins as shown in Fig 3. The regression coefficients of GA_4 on GA_1 in log were estimated as 0.01, 0.42, 0.42, 0.40, 0.49 for root, forage, leaf, grain, stalk with P values of 0.94, 0.10, 0.03, <0.01, <0.01, respectively, which are significant at the 5% level for leaf, grain, and stalk, and non-significance for forage. Notably, when examining residual variation after accounting for hybrid and site effects, positive correlations were observed with similar magnitude.

Fig 4. Biplot and Correlation Between GA_1 and GA_4 Means Across Five Maize Tissues

Formatted: No underline

The figure illustrates the relationship between gibberellin A1 (GA_1) and gibberellin A4 (GA_4) concentrations across five maize tissues: root, forage, leaf, grain, and stalk. Each marker in the biplot represents the predicted hybrid mean for GA_1 and GA_4 at a specific site, derived from four replicates, expressed in pmol/g (log-transformed and back-transformed). Different tissues are distinguished by unique markers and colors.

Discussion

Quantitative analysis of gibberellins has advanced rapidly across diverse crops with the development of LC–MS/MS-based workflows [27], including methods for broad GA profiling [28, 29] and high-efficiency quantitation in complex plant matrices [20, 30]. Thise ~~eCurrent~~ study focus~~s~~es on endogenous GA$_1$ and GA$_4$ quantification across five tissues ~~to~~of field-grown commercial maize hybrids sampled across diverse environments, providing a baseline dataset for describing ~~interpreting~~ natural variation under ~~real-world~~standard agricultural practices ~~conditions~~.

~~The current study provides quantification of endogenous GA$_1$ and GA$_4$ across five maize tissues collected from diverse field environments across the United States.~~ Among the~~se~~ five tissues, forage exhibited the highest bioactive gibberellins concentrations and the broadest dynamic range, with GA$_1$ ranging from 0.4 to 131.1 pmol/g and GA$_4$ from 0.1 to 29.2 pmol/g. Published data for forage tissue are limited; while available data for shoot tissue ~~are available and~~ can serve as a reference. GA$_1$ levels in shoot ~~(used as a proxy for forage)~~ ranged from 0.04 to 274.4 pmol/g ~~ADDIN EN.CITE~~ [31, 32] and~~.~~ GA$_4$ ~~levels~~ in the shoot tips ranged from 0.06 to 1.05 pmol/g ~~ADDIN EN.CITE~~ [21, 33]~~, which are notably lower than the maximum forage GA$_4$ values observed in this study. This might due to different tissues studied or it could indicate that field-grown forage may accumulate higher GA$_4$ levels, potentially due to environmental stimulation or hybrid-specific biosynthetic activity.~~

Stalk showed a narrower and more stable range (GA$_1$: 0.9–3.4 pmol/g; GA$_4$: 0.2–0.9 pmol/g), aligning closely with literature values (GA$_1$ ~1.8 pmol/g; GA$_4$: 0.33–0.98 pmol/g) [19]. This consistency ~~supports~~ suggests that~~the use of~~ stalk may be ~~as a stable reference~~ a useful tissue for

Formatted: Subscript

Formatted: Subscript

Formatted: Left

comparison in future studies. for gibberellins quantification, likely due to its structural maturity and reduced metabolic flexibility.

Leaf in this study showed GA_1 levels rangeding from 0.4 to 27.0 pmol/g and GA_4 from 0.1 to 1.4 pmol/g. The GA_1 concentrations observed here are higher than literature-reported ranges of 0–1.8 pmol/g, possibly due to the limited number of samples reported previously, while GA_4 levels fall within the broader literature range of 0–3.5 pmol/g [19, 33, 34]. The elevated GA_1 levels may reflect enhanced biosynthesis in field-grown hybrids or differences in tissue sampleing, developmental stage, at sampling genotype, field conditions, or study design. In contrast, the GA_4 levels, although lower than the upper literature limit, are consistent with expected physiological ranges and suggest that GA_4 biosynthesis in leaf tissue may be more tightly regulated or less variable than GA_1. GA4 levels in leaf tissue appeared less variable than GA1 in this dataset.

Grain exhibited GA_1 levels from 0.1 to 26.0 pmol/g and GA_4 from 0.3 to 2.0 pmol/g, exceeding literaturethe ranges (GA_1: 0.29–2.0 pmol/g; GA_4: 0–1.2 pmol/g) reported from literature [22, 35]. This suggests that grain gibberellins levels in grain may be more variablespan a broader range than previously reported. The tight range data from in previous literature may be possibly reflecting differences in sample number, hybrid background, field site, developmental stage, or sampling designdue to the limited number of the samples analyzed or due to hybrid-specific traits or environmental modulation during seed development [35].

Root in this study showed GA_1 concentrations ranging from 0.1 to 1.3 pmol/g and GA_4 from 0.1 to 1.8 pmol/g. While literature data for gibberellins levels in maize roots are limited or not reported, these findings provide a valuable baseline for future studies. The relatively narrow range and lower mean values -indicate that measured GA_1 and GA_4 levels in root were lower and less variable than in several aboveground tissues in this datasetsuggest that gibberellins biosynthesis in root tissues

Formatted: Subscript

Formatted: Subscript

may be tightly regulated or less responsive to environmental variation compared to aboveground tissues. These results also highlightsupport the importance of including belowincluding below-ground tissues in future GA hormonal profiling studies to better understand whole plant hormone dynamics.describe tissue-specific GA abundance.

Overall, these comparisons highlight the broader dynamic range of bioactive GA_1 and GA_4 captured in this study, particularly for forage, leaf, and grain tissues. The broader ranges observed in several tissues may reflect the larger number of samples, field-grown conditions, developmental differences, and the use of commercial hybrids compared with prior studiesThe elevated gibberellins levels observed in several tissues may be linked to heterosis, as previous studies have shown that F1 hybrids often exhibit enhanced gibberellins biosynthesis [31]. The comparison of literature data with our findings reinforces the robustness of the LC-MS/MS method and provides a valuable reference for future studies of endogenous on GA_1 and GA_4 abundance gibberellins-mediated growth regulation in maize tissues.

The present study focused on bioactive gibberellin quantification at the tissue level, potential research could be considered about the spatial distribution of gibberellins within growing organs which would be valuable for elucidating how localized gibberellin gradients contribute to organ growth and developmental patterning.

In this investigation, tThe contribution of different sources of variance components to measured the gibberellin expression GA_1 and GA_4 levels was analyzed for relative comparisons by using an all-random-effect model. The model (1) includes effects of site, hybrid, hybrid by site interaction, replicate, and residual. The hybrid effect represents the genetic effect, site effect may include various environmental captures difference among field sites and may include environmental, agronomic, and site-specific sampling factors. factors., and the residual includes the plant sample

Formatted: Subscript

Formatted: Subscript

Formatted: Subscript

Formatted: Subscript

Formatted: Subscript

Formatted: Subscript

Formatted: Font: (Default) +Body (Aptos)

~~variation in the field and the assay variation in the lab.~~ Model (1) included the effects of site, hybrid, the hybrid × site interaction, replicate nested within site, and residual. The hybrid effect represents genetic variation, and the site effect represents environmental variation among locations. Replicate nested within site accounts for variation among field replicates within each location, whereas the residual captures the remaining unexplained variation, including plant sampling variation in the field and assay variation in the laboratory. The hybrid × site interaction was further examined for tissues showing evidence of interaction in the variance component analysis.

Results in Table ~~5~~6 and Fig 2 indicated that while both genetic and environment are important factors, the site is a major contributor as compared with the genetic background to the gibberellin levels~~expression~~. In addition, in five out of ten (gibberellins by tissue) combinations, residual variations are greater than the model variation, especially for forage and root.

~~Site represents the main environmental factor that affects gibberellin concentrations in maize tissues, while hybrid represents the genetic factor. The interaction between environmental and genetic factors (hybrid × site) was further examined for tissues showing evidence of interaction in the variance component analysis.~~

Results ~~summarized in~~ in Table ~~5~~6, together with means in Table ~~4~~5, demonstrate substantial differences in the distribution of GA_1 and GA_4 among the five tissues. For example, forage exhibited the highest mean concentrations as well as the largest residual variances for both GA_1 and GA_4, whereas stalk showed intermediate mean concentrations but the lowest residual variances. Despite this lower residual variability, stalk accounted for a higher proportion of total model-explained variation than forage, as shown in Fig 2.

Across the ten gibberellins-tissue combinations analyzed, a significant hybrid contribution was detected only for GA_4 in grain (Varg), compared with five combinations exhibiting significant site effects (Vars). Overall, six of the ten combinations showed at least one significant variance component attributable to site, hybrid, or site × hybrid interaction (Vargxs). Notably, only root showed no significant variance components for either GA_1 ~~and~~or GA_4, while hybrid × site interactions were significant for both gibberellins in stalk.

Estimation and interpretation of variance components depend on satisfaction of the model's normality assumptions. Following log transformation, the variance component estimates reported in Table ~~5~~6 are presented on the log scale and should be interpreted in a relative sense, whereas mean concentrations and associated standard errors reported in Table ~~4~~5 are expressed in absolute units (pmol/g).

The experimental design was constrained by hybrid adaptation and site capacity, resulting in different subsets of hybrids being planted at each site. Because commercial maize hybrids are bred for specific maturity zones and local growing conditions, it was not feasible to plant the same set of hybrids across the full range of environments represented in this study. This approach was necessary to achieve broad geographic and environmental coverage, despite resulting in an unbalanced hybrid–site design.

~~As a consequence of this design, estimation and statistical testing of variance components — particularly hybrid × site interactions — are based on reduced degrees of freedom.~~ As a consequence of this design, estimation and statistical testing of variance components, especially hybrid × site interactions, are based on reduced degrees of freedom. For example, although the experiment included ten hybrids and six sites, a fully balanced design would have yielded 45 degrees of freedom for the interaction term, whereas the mixed-model analysis of the available

data provided only three. This imbalance limits the precision of variance component estimates and reduces statistical power for significance testing.

Despite these limitations, the unbalanced design does not preclude qualitative interpretation of the relative contributions of genetic, environmental, and interaction effects. The variance component analysis was conducted using an all-random-effects mixed model, and significance testing was performed using likelihood-based methods, which are appropriate for unbalanced data. While quantitative estimates should therefore be interpreted cautiously, trends comparing the relative importance of site, hybrid, and hybrid × site effects remain informative.

As shown in Fig. 3, gibberellin concentration profiles for many genetic backgrounds are largely parallel across sites, suggesting consistent environmental responses. However, some hybrids exhibit clear site-dependent variation, indicating that both genetic and environmental factors contribute meaningfully to gibberellin accumulation. For example, the hybrid Dekalb DKC58-32 showed the highest gibberellin concentrations at site 4 but relatively lower concentrations at other sites, illustrating environment-dependent expression patterns. Although the specific environmental drivers underlying these differences cannot be resolved in the present study, factors such as temperature, humidity, and rainfall are likely contributors, and further targeted experimentation will be required to assess their relative importance.

~~Biosynthetic pathways for GA_1 and GA_4 share a common upstream route and downstream branches that use the same enzyme families (e.g., GA20ox and GA3ox) [10]. This biochemical architecture makes coordinated variation between GA_1 and GA_4 plausible.~~ In our field dataset, GA_1 and GA_4 were positively correlated in forage, grain, leaf, and stalk, but not in root. Because these data were collected under ~~real world~~standard agronomic conditions, the observed correlations could also reflect a shared response to overall plant physiological status (e.g., more

vigorous growth) and to environmentally driven differences in growing conditions among sites, consistent with the strong site effects detected in the variance component analysis. Notably, the GA_1–GA_4 association also persisted when examining residual variation after accounting for hybrid, site, and hybrid × site effects~~. We recorded some growth and environmental information during the trials; however, these measures were not sufficiently detailed to support a robust covariate adjustment aimed at separating direct co-regulation from confounding by overall vigor or weather. Therefore, while co-regulation through shared upstream control points or transcriptional programs is possible, our results are correlational and do not distinguish among these alternative explanations.~~

Conclusions

In this study, we applied a validated high-throughput LC-MS/MS method to simultaneously quantify the bioactive gibberellins GA_1 and GA_4 across multiple tissues of commercial maize hybrids grown in North American field environments. ~~Our~~ This work provides a field ~~establishes the first comprehensive~~ based baseline dataset ~~of natural~~describing endogenous GA_1 and GA_4 variation under ~~real world~~standard ~~-~~agricultural practices~~conditions~~. We found that gibberellin concentrations varied significantly across tissues, with forage exhibiting the broadest dynamic range and highest levels, while stalk tissue showed narrower ~~more stable and narrow~~ concentration ranges, which may make it useful~~suggesting its potential as a reference tissue~~ for comparison in ~~for~~ future studies.

~~A key finding of this research is the substantial influence of the environment on gibberellin regulation.~~ Variance component analysis ~~revealed~~ indicated that site-associated variation contributed to ~~the field site was the most significant factor driving the variability in~~ measured GA_1

and GA_4 levels forin several most tissue-by-analyte combinationss. This underscores the remarkable plasticity of the gibberellin expression level pathway in response to local environmental conditions and highlights the importance of considering environmental context in studies of hormonal regulation. We identified a significant genetic (hybrid) contribution to GA_4 variance specifically in grain, indicating that GA_4 levels in grain may be subject to genetic control. This presents a potential target for hybrid selection and breeding programs aimed at optimizing agronomic traits, including yield. The detection of hybrid-associated variance for GA4 in grainwhich suggests that this tissue-analyte combination may warrant further study in larger, targeted experiments

While this study provides a foundational dataset, it is important to note that it is based on a single growing season and limited to bioactive GA_1 and GA_4. Future multi-year studies and additional GA metabolites will be necessary to confirm the stability of these trends and further explore the impact of annual weather variations. We hope that the data and analyses presented here provide useful insights into the complex interplay between genetics and, environment, and hormonal regulation in maize. This research may serve as an essential framework for future investigations into the specific environmental drivers of gibberellins expression and provides a compelling rationale for exploring the genetic basis of GA_4 accumulation in grain as a potential avenue for crop improvement.

Formatted: Subscript

Formatted: Subscript

Acknowledgments

The authors thank Leah Riter, Vinod Jakkula, Edward Cargill, Mohamed Bedair, David Caldwell, Prema Karunanithi and Paul Tietz for providing valuable feedback on the manuscript. The authors also extend special thanks to Xin Li for his valuable input on the statistical analysis presented in the manuscript.

References

1. Kurosawa E. Experimental studies on the nature of the substance secreted by the" bakanae" fungus. Nat Hist Soc Formosa. 1926;16:213-27.

2. Castro-Camba R, Sánchez C, Vidal N, Vielba JM. Plant Development and Crop Yield: The Role of Gibberellins. Plants (Basel). 2022;11(19). Epub 20221009. doi: 10.3390/plants11192650. PubMed PMID: 36235516; PubMed Central PMCID: PMCPMC9571322.

3. Yamaguchi S. Gibberellin metabolism and its regulation. Annu Rev Plant Biol. 2008;59:225-51. doi: 10.1146/annurev.arplant.59.032607.092804. PubMed PMID: 18173378.

4. Ubeda-Tomás S, Federici F, Casimiro I, Beemster GTS, Bhalerao R, Swarup R, et al. Gibberellin Signaling in the Endodermis Controls Arabidopsis Root Meristem Size. Current Biology. 2009;19(14):1194-9. doi: 10.1016/j.cub.2009.06.023.

5. Verma V, Ravindran P, Kumar PP. Plant hormone-mediated regulation of stress responses. BMC Plant Biol. 2016;16:86. Epub 20160414. doi: 10.1186/s12870-016-0771-y. PubMed PMID: 27079791; PubMed Central PMCID: PMCPMC4831116.

6. Illouz-Eliaz N, Ramon U, Shohat H, Blum S, Livne S, Mendelson D, et al. Multiple Gibberellin Receptors Contribute to Phenotypic Stability under Changing Environments. Plant Cell. 2019;31(7):1506-19. Epub 20190510. doi: 10.1105/tpc.19.00235. PubMed PMID: 31076539; PubMed Central PMCID: PMCPMC6635849.

7. Rizza A, Jones AM. The makings of a gradient: spatiotemporal distribution of gibberellins in plant development. Curr Opin Plant Biol. 2019;47:9-15. Epub 20180830. doi: 10.1016/j.pbi.2018.08.001. PubMed PMID: 30173065; PubMed Central PMCID: PMCPMC6414749.

8. Nelissen H, Rymen B, Jikumaru Y, Demuynck K, Van Lijsebettens M, Kamiya Y, et al. A local maximum in gibberellin levels regulates maize leaf growth by spatial control of cell

division. Curr Biol. 2012;22(13):1183-7. Epub 20120607. doi: 10.1016/j.cub.2012.04.065. PubMed PMID: 22683264.

9. Hedden P. The Current Status of Research on Gibberellin Biosynthesis. Plant Cell Physiol. 2020;61(11):1832-49. doi: 10.1093/pcp/pcaa092. PubMed PMID: 32652020; PubMed Central PMCID: PMCPMC7758035.

10. Salazar-Cerezo S, Martínez-Montiel N, García-Sánchez J, Pérez-y-Terrón R, Martínez-Contreras RD. Gibberellin biosynthesis and metabolism: A convergent route for plants, fungi and bacteria. Microbiological research. 2018;208:85-98.

11. Ilyas M, Nisar M, Khan N, Hazrat A, Khan AH, Hayat K, et al. Drought Tolerance Strategies in Plants: A Mechanistic Approach. Journal of Plant Growth Regulation. 2021;40(3):926-44. doi: 10.1007/s00344-020-10174-5.

12. Emongor V. Gibberellic Acid (GA3) Influence on Vegetative Growth, Nodulation and Yield of Cowpea (Vigna unguiculata (L.) Walp. Journal of Agronomy. 2007;6:509-17.

13. Shani E, Hedden P, Sun T-p. Highlights in gibberellin research: A tale of the dwarf and the slender. Plant Physiology. 2024;195(1):111-34. doi: 10.1093/plphys/kiae044.

14. Rizza A, Walia A, Lanquar V, Frommer WB, Jones AM. In vivo gibberellin gradients visualized in rapidly elongating tissues. Nature Plants. 2017;3(10):803-13. doi: 10.1038/s41477-017-0021-9.

15. Binenbaum J, Weinstain R, Shani E. Gibberellin Localization and Transport in Plants. Trends in Plant Science. 2018;23(5):410-21. doi: 10.1016/j.tplants.2018.02.005.

16. Wu K, Xu H, Gao X, Fu X. New insights into gibberellin signaling in regulating plant growth-metabolic coordination. Curr Opin Plant Biol. 2021;63:102074. Epub 20210701. doi: 10.1016/j.pbi.2021.102074. PubMed PMID: 34217918.

17. Erenstein O, Jaleta M, Sonder K, Mottaleb K, Prasanna BM. Global maize production, consumption and trade: trends and R&D implications. Food Security. 2022;14(5):1295-319. doi: 10.1007/s12571-022-01288-7.

18. Stokstad E. High hopes for short corn. Science. 2023;382(6669):364-7. Epub 20231026. doi: 10.1126/science.adl5302. PubMed PMID: 37883569.

19. Paciorek T, Chiapelli BJ, Wang JY, Paciorek M, Yang H, Sant A, et al. Targeted suppression of gibberellin biosynthetic genes ZmGA20ox3 and ZmGA20ox5 produces a short stature maize ideotype. Plant Biotechnol J. 2022;20(6):1140-53. Epub 20220309. doi: 10.1111/pbi.13797. PubMed PMID: 35244326; PubMed Central PMCID: PMCPMC9129074.

20. Su X, Zheng J, Hao Y, Xia R, Xu J. A high-efficiency method for simultaneous quantitation of bioactive gibberellins in Litchi chinensis using UHPLC-QQQ-MS/MS. Scientia Horticulturae. 2024.

21. Zhang J, Zhang X, Chen R, Yang L, Fan K, Liu Y, et al. Generation of Transgene-Free Semidwarf Maize Plants by Gene Editing of Gibberellin-Oxidase20-3 Using CRISPR/Cas9. Front Plant Sci. 2020;11:1048. Epub 20200709. doi: 10.3389/fpls.2020.01048. PubMed PMID: 32742269; PubMed Central PMCID: PMCPMC7365143.

22. Murofushi N, Honda I, Hirasawa R, Yamaguchi I, Takahashi N, Phinney BO. Gibberellins from the Seed, Tassel, Cob and Silk of Maize. Agricultural and Biological Chemistry. 1991;55(2):435-9. doi: 10.1080/00021369.1991.10870589.

23. Yamaguchi I, Nakazawa HK, Nakagawa R, Suzuki Y, Kurogochi S, Murofushi N, et al. Identification and Semi-Quantification of Gibberellins from the Pollen and Anthers of Zea mays by Immunoassay and GC/MS. Plant and Cell Physiology. 1990;31:1063-9.

24. Murofushi N, Honda I, Hirasawa R, Yamaguchi I, Takahashi N, Phinney BO, editors. Gibberellins from the Tassel, Cob, 'Seed,' Silk, and Pollen of Maize1991; New York, NY: Springer New York.

25. Searle SRC, G; McCulloch, C.E. . Variance Components John Wiley & Sons, Inc; 2006.

26. Sahai HA, M. The Analysis of Variance Birkhäuser Boston, MA; 2012.

27. Pan C, Tan, S.N., Yong, J.W.H., Ge, L. Progress and development of analytical methods for gibberellins. Journal of Separation Science. 2017;40:346-60. doi: https://doi.org/10.1002/jssc.201600857.

28. Urbanová T, Tarkowská D, Novák O, Hedden P, Strnad M. Analysis of gibberellins as free acids by ultra performance liquid chromatography–tandem mass spectrometry. Talanta. 2013;112:85-94. doi: https://doi.org/10.1016/j.talanta.2013.03.068.

29. López-Cristoffanini C, Serrat X, Jáuregui O, Nogués S, López-Carbonell M. Phytohormone Profiling Method for Rice: Effects of GA20ox Mutation on the Gibberellin Content of Japonica Rice Varieties. Frontiers in Plant Science. 2019;10. doi: 10.3389/fpls.2019.00733.

30. Fernández H, Doumas P, Bonnet-Masimbert M. Quantification of GA1, GA3, GA4, GA7, GA8, GA9, GA19 and GA20; and GA20 metabolism in dormant and non-dormant beechnuts. Plant Growth Regulation. 1997;22(1):29-35. doi: 10.1023/A:1005814926549.

31. Rood SB, Buzzell RI, Mander LN, Pearce D, Pharis RP. Gibberellins: A Phytohormonal Basis for Heterosis in Maize. Science. 1988;241(4870):1216-8. doi: doi:10.1126/science.241.4870.1216.

32. Fujioka S, Yamane H, Spray CR, Katsumi M, Phinney BO, Gaskin P, et al. The dominant non-gibberellin-responding dwarf mutant (D8) of maize accumulates native gibberellins. Proc Natl Acad Sci U S A. 1988;85(23):9031-5. doi: 10.1073/pnas.85.23.9031. PubMed PMID: 16594001; PubMed Central PMCID: PMCPMC282656.

33. Liu Y, Chen Z, Zhang C, Guo J, Liu Q, Yin Y, et al. Gene editing of ZmGA20ox3 improves plant architecture and drought tolerance in maize. Plant Cell Rep. 2023;43(1):18. Epub 20231226. doi: 10.1007/s00299-023-03090-x. PubMed PMID: 38148416.

34. Chen Z, Liu Y, Yin Y, Liu Q, Li N, Liu X, et al. Development of dwarfish and yield-effective GM maize through passivation of bioactive gibberellin. Transgenic Res. 2019;28(5-6):589-99. Epub 20191008. doi: 10.1007/s11248-019-00172-z. PubMed PMID: 31595387.

35. Constance NW, Proebsting WM, Hedden P, Rivin CJ. Gibberellins and Seed Development in Maize. I. Evidence That Gibberellin/Abscisic Acid Balance Governs Germination versus Maturation Pathways. Plant Physiology. 2000;122(4):1081-8.

Supporting information

S1 Table. Summary of sample type and collection information.

S2 Table. Value assigned to non-detection (ND) data points for statistical analysis

S1 Fig. The representative MRM chromatogram for GA1 and GA4 in the LLOQ (standard 1)

Contributorship

Credit: Yanfei Wang: Conceptualization, Project administration, Resources, Supervision, Writing – review & editing; Xia Guan: Formal analysis, Investigation, Methodology, Writing – original draft, Writing – review & editing; Nitin Supekar: Formal analysis, Investigation, Writing – original draft, Writing – review & editing; Changjian Jiang: Statistical analysis, Data curation, Formal analysis, Methodology, Writing – original draft, Writing – review & editing; Junhong Guo: Methodology, Formal analysis, Investigation, Writing – original draft, Writing – review & editing; Ed Allen: Project administration, Writing – review & editing; Charles Dietrich: Project administration, Writing – review & editing; Cody Postin: Formal analysis, Investigation, Methodology, Writing – original draft, Writing – review & editing; Shubha Shakya: Figure 1 udpate, Writing – review & editing;

Response to Reviewers

PONE-D-25-66749R2

Natural variation of bioactive gibberellin levels in maize tissues across North America

PLOS One

Dear Dr. Wang,

Thank you for submitting your manuscript to PLOS ONE. After careful consideration, we feel that it has merit but does not fully meet PLOS ONE's publication criteria as it currently stands. Therefore, we invite you to submit a revised version of the manuscript that addresses the points raised during the review process.

Response
Dear Editor,
Thank you very much for your letter and for the careful evaluation of our manuscript. We sincerely appreciate your comments and the reviewers' constructive suggestions, which have been very helpful in improving the quality and clarity of our work.
We have carefully addressed the comments from both the editor and the reviewers on a point-by-point basis. In response to these comments, we have revised the manuscript accordingly and made the necessary changes throughout the text.
In our revised submission, we have included the following files as requested:
1. Response to Reviewers – a separate letter responding to each point raised by the academic editor and reviewers.
2. Revised Manuscript with Track Changes – a marked-up version highlighting all changes made to the manuscript PONE-D-25-66749R1.
3. Manuscript – a clean, unmarked version of the revised paper without tracked changes.
Thank you again for your time and consideration. We are grateful for the opportunity to revise our manuscript for further consideration by PLOS ONE.

You have improved the paper notably. Nevertheless problems remain. Reviewer 1, a statistician, points out fairly serious inadequacies remain in your presentation. Insofar as statistical analysis constitutes a big chunk of your paper, I believe that you need to get this right. Hopefully, the problems identified by the reviewer are matters of language (what you write about the methods). But in any case, I hope you can respond satisfactorily.

Response: Thank you for highlighting this important concern. We appreciate the editor's emphasis on the need for a clear and rigorous presentation of the statistical analysis, particularly given its central role in the manuscript. In response, we carefully re-examined the statistical methods and substantially revised the "Variance component analysis to compare genetic and environmental variations" section to improve clarity, precision, transparency, and consistency.
We thoroughly revised the methodology section to clarify our statistical approaches, with emphasis on model assumptions, structure, and limitations. We explicitly defined our variance-partitioning framework, detailing how hybrid, site, replicate within site, and the hybrid-by-site interaction were treated across different stages of the analysis, specifically for estimating variance components, testing their significance, and examining changes in hybrid means across sites. To address the reviewer's concerns, we clarified that our use of REML (Restricted Maximum Likelihood) estimation is a well-established approach for variance component estimation in unbalanced trial designs. We also explained our rationale for not applying multiple-testing adjustments during significance testing of these components, and why confidence intervals for variance components were not provided. In addition, we removed references to SAS PROC GLM, which had been used only for intermediate data exploration, so that the manuscript now reflects only the analyses underlying the reported results. Overall, these revisions present the statistical methodology with greater transparency, stronger theoretical justification, and more appropriate caution regarding the scope and unbalanced nature of the available data.

Reviewer three (the original reviewer 2 was not available) points out several small but useful things. Their main point tho is about interpretation. Writing for PLoS One differs from many other journals because "importance" does not matter. For most journals, we write to convince the editor, the reviewers, and indeed readers, that our work is important. But importance is subjective. PLoS One removes that criteron, explicitly. Instead, what matters is validity. Writing on that head relieves us of the burden of hype. We present what we found, a straightforward account. We can of course state why we think the work matters but in doing so we should be brief and circumspect. Reviewer 3 points out where you need to town down your claims. I would add that you can probably shorten the discussion; compared to the results, it seems quite long.

Response: Thank you for the great comments. The revised Discussion now focuses more directly on the validity of the findings, the limitations of the study design and measurements, and a more circumspect interpretation of the implications.
We revised the Discussion to align more closely with the data presented in the Results. Specifically, we removed or rephrased language that could be interpreted as overly broad or mechanistic, including statements related to "biosynthesis" and

“plasticity of the gibberellin pathway,”. We also shortened the Discussion by removing repetitive statements and reducing broader contextual language.

Small points: When you cite references in the text, please put a space between the word and the bracket. Like this [14]. Not like this[14]. Also on line 74, the word 'corn' should not be capitalized.

Response: Thank you for pointing these out. We have made these corrections throughout the manuscript, including adding a space before citation brackets where needed and changing “Corn” to “corn” on previous line 74.

Please submit your revised manuscript by Aug 05 2026 11:59PM. If you will need more time than this to complete your revisions, please reply to this message or contact the journal office at plosone@plos.org. When you're ready to submit your revision, log on to https://www.editorialmanager.com/pone/ and select the 'Submissions Needing Revision' folder to locate your manuscript file.

Please include the following items when submitting your revised manuscript:

- A letter that responds to each point raised by the academic editor and reviewer(s). You should upload this letter as a separate file labeled 'Response to Reviewers'.
- A marked-up copy of your manuscript that highlights changes made to the original version. You should upload this as a separate file labeled 'Revised Manuscript with Track Changes'.
- An unmarked version of your revised paper without tracked changes. You should upload this as a separate file labeled 'Manuscript'.

If you would like to make changes to your financial disclosure, please include your updated statement in your cover letter. Guidelines for resubmitting your figure files are available below the reviewer comments at the end of this letter.

If applicable, we recommend that you deposit your laboratory protocols in protocols.io to enhance the reproducibility of your results. Protocols.io assigns your protocol its own identifier (DOI) so that it can be cited independently in the future. For instructions see: https://journals.plos.org/plosone/s/submission-guidelines#loc-laboratory-protocols. Additionally, PLOS ONE offers an option for publishing peer-reviewed Lab Protocol articles, which describe protocols hosted on protocols.io. Read more information on sharing protocols at https://plos.org/protocols?utm_medium=editorial-email&utm_source=authorletters&utm_campaign=protocols.
As the corresponding author, your ORCID iD is verified in the submission system and will appear in the published article. PLOS supports the use of ORCID, and we encourage all coauthors to register for an ORCID iD and use it as well. Please encourage your coauthors to verify their ORCID iD within the submission system before final acceptance, as unverified ORCID iDs will not appear in the published article. *Only* the individual author can complete the verification step; PLOS staff *cannot* verify ORCID iDs on behalf of authors.

We look forward to receiving your revised manuscript.
Kind regards,
Tobias Isaac Baskin
Academic Editor
PLOS One

Journal Requirements:
If the reviewer comments include a recommendation to cite specific previously published works, please review and evaluate these publications to determine whether they are relevant and should be cited. There is no requirement to cite these works unless the editor has indicated otherwise.

[Note: HTML markup is below. Please do not edit.]

Reviewers' comments:
Reviewer's Responses to Questions
Comments to the Author
1. If the authors have adequately addressed your comments raised in a previous round of review and you feel that this manuscript is now acceptable for publication, you may indicate that here to bypass the “Comments to the Author” section, enter your conflict of interest statement in the “Confidential to Editor” section, and submit your "Accept" recommendation.
Reviewer #1: (No Response)
Reviewer #3: (No Response)

---

2. Is the manuscript technically sound, and do the data support the conclusions?

The manuscript must describe a technically sound piece of scientific research with data that supports the conclusions. Experiments must have been conducted rigorously, with appropriate controls, replication, and sample sizes. The conclusions must be drawn appropriately based on the data presented.
Reviewer #1: Partly
Reviewer #3: Partly

---

3. Has the statistical analysis been performed appropriately and rigorously?
Reviewer #1: No
Reviewer #3: Yes

---

4. Have the authors made all data underlying the findings in their manuscript fully available?

The PLOS Data policy requires authors to make all data underlying the findings described in their manuscript fully available without restriction, with rare exception (please refer to the Data Availability Statement in the manuscript PDF file). The data should be provided as part of the manuscript or its supporting information, or deposited

to a public repository. For example, in addition to summary statistics, the data points behind means, medians and variance measures should be available. If there are restrictions on publicly sharing data—e.g. participant privacy or use of data from a third party—those must be specified.
Reviewer #1: Yes
Reviewer #3: Yes

---

5. Is the manuscript presented in an intelligible fashion and written in standard English?

PLOS ONE does not copyedit accepted manuscripts, so the language in submitted articles must be clear, correct, and unambiguous. Any typographical or grammatical errors should be corrected at revision, so please note any specific errors here.
Reviewer #1: Yes
Reviewer #3: Yes

---

6. Review Comments to the Author

Please use the space provided to explain your answers to the questions above. You may also include additional comments for the author, including concerns about dual publication, research ethics, or publication ethics. (Please upload your review as an attachment if it exceeds 20,000 characters)
Reviewer #1: Experiments (blocking structure, randomisation, etc) are now much more clearly explained in the Methods. Though l130-132 "Maize samples were collected throughout the growing season and included the following tissues: leaf, root, stalk, pollen, forage, and grain. The same tissues collected from each plot were pooled into a single sample." remains somewhat ambiguous. How much of each tissue (and was this by weight, or volume, or something else)? From how many plants, and how were they selected (random, non-edge, convenience)? How pooled? Was pooling consistent across all sites?

Response: Thank you for the comments. We have revised the Methods section to clarify sufficient details regarding the amount of tissue collected, the number of plants sampled, the selection criteria, and the pooling procedure. For each plot, sampled plants were selected non-systematically but with consistent field-sampling criteria: plot ends were avoided, and plants were chosen to be competitive and representative of the plot as a whole. The selected tissue samples from each plot were combined to generate one pooled sample per plot, which served as the observational unit for downstream analysis. This pooling approach was applied consistently across all sites.
We have added detailed sampling information in S1 Table, including the pooled sample amount per plot, the number of plants sampled per plot, tissue-specific sampling details, and the collection growth stage. Specifically, the revised table now specifies the tissue-specific sampling amount used to generate one pooled sample per lot: 60 leaf sections, 8 roots, 4 stalks, 10 mL pollen, 1 forage plant, or 1 kg grain, depending on tissue type, along with the corresponding number of sampled plants and growth stages.

S1 Table. Summary of sample type and collection information

Sample Type	*Pooled Sample Amount per Plot*	*Number of Plants Sampled per Plot*	*Sampling Details*	*Collection Growth Stage*	*Growth Stage Description*
Leaf	*60 leaves*	*60*	*Approximately 15 cm of most recently collared leaf*	*V2-V4*	*A plant with 2 to 4 leaf collars*
Root	*8 roots*	*8*	*All below-ground root tissue, thoroughly washed*	*V2-V4*	*A plant with 2 to 4 leaf collars*
Stalk	*4 stalks*	*4*	*All above-ground stalk tissue with leaf blade, leaf sheath, ear, and tassel removed*	*V10-V12*	*A plant with 10 to 12 leaf collars*
Pollen	*10 mL*	*Highly variable based on environmental conditions, though typically >10*	*Fresh pollen with all anthers removed*	*Pollen shed*	*A plant that is actively shedding pollen*
Forage	*1 plant*	*1*	*All above-ground plant tissue*	*R5, early dent*	*Most kernels are dented and contain approximately 55% moisture*
Grain	*1 kg*	*Minimum of 4*	*Shelled grain dried to ≤15.5% moisture prior to shipping and storage*	*R6*	*Physiological maturity; black layer visible at base of kernels*

Whilst the Methods section explains the experiment better, there's been no attempt made to connect it to the subsequent model, eqn (1). Does "block" (in intro) = "replicate" (in the model)? Does "plot" (in the intro) = the observational unit (in the model)? Does "pooling" (in intro) mean there is one observation per plot? The disconnect is a bit jarring, but could easily be fixed.

Response: Thank you for the suggestion. In the revised manuscript, we clarified that "block" in the experimental design corresponds to "replicate" in the statistical

model, and that "plot" is the observational unit used in the model. We also clarified that the tissue samples collected within each plot were pooled to generate one composite sample per plot; therefore, there was one analytical observation per plot for each tissue and GA measurement. These clarifications have been added to the revised Methods section to better connect the field design, sample pooling, and statistical model.

The issue of unbalancedness is still not well acknowledged or addressed. I appreciate that this is (now) historical data and it is out of scope to remedy the imbalance by collecting more data. Nevertheless, it is important still that the statistical inference is accurate and doesn't overstate what the data imply. l202-230 unfortunately still feel muddled with respect to the assumed statistical model. l204-206 refer to "fitting the model", but does so before fully specifying the model: which coefficients are random, and with what variance structure? The sentence "Differences may present under an unbalanced design as in this investigation, and PROC GLIMMIX is the best for significant testing due to the likelihood-based method (which is the ultimate standard for all statistical analyses), PROC MIXED supposes to be unbiased for the estimate of variance component because of the restricted maximum likelihood and the application of mixed model equations for random effects, and PROC GLM provided mean predictions of random effects by the method of moment (i.e. the expected mean square or EMS)" is very unclear to me. It does not describe the statistical assumptions or methods directly, but rather refers to SAS procedures and seems to expect the reader to reverse-engineer the implied model from that. "PROC GLM provided mean predictions of random effects by the method of moment" is especially confusing: my understanding is that PROC GLM applies to fixed effects rather than random effects, so what is meant by this statement is at best unclear. This whole section remains muddled, suggests over-reliance/over-confidence in software, and still underplays the problems caused by the unbalanced design. Likelihood-based methods are not automatically reliable in small or highly unbalanced settings, and similarly REML "unbiasedness" is not guaranteed in that same setting. It should not be for the reader or reviewer to have to unpick this.

Response: The manuscript was revised to clarify the methodological approaches. Specifically, to estimate variance components, hybrid, site, replicate within site, and the hybrid-by-site interaction were all treated as random effects, with the residual term representing error variance. Random effects were assumed to be independently and identically distributed within each classification, each associated with its own variance component. Variance was partitioned into these additive components using SAS PROC MIXED with REML (Restricted Maximum Likelihood) estimation, a well-established approach for variance component estimation in unbalanced trial designs.

The significance of variance components was then evaluated using PROC GLIMMIX with the COVTEST option via likelihood ratio tests. Because the null hypothesis places a variance component on the boundary of the parameter space at zero, the resulting test statistic follows a mixture chi-square distribution.

Significance was evaluated at the 5%, 1%, and 0.1% levels without multiplicity correction.
For variance components that were found to be significant, additional analyses were performed to examine mean changes for individual hybrids across sites, thereby illustrating the extent to which hybrid effects were connected across sites and characterizing site-specific hybrid patterns.
These analyses were conducted within a linear mixed model framework using PROC MIXED. hybrid, site, and the hybrid-by-site interaction were treated as fixed effects, whereas Replicate nested within Site was modeled as a random effect. Treating hybrid and site as fixed effects produced local LS-means for characterizing hybrid-by-site patterns, while modeling nested replications as random prevented estimates from being biased by local block-level anomalies.
In addition, we removed references to SAS PROC GLM, which had been used only for intermediate data exploration, so that the manuscript now reflects only the analyses underlying the reported results.
Consequently, the approach provides valid and reliable inference for unbalanced data, though statistical power may be reduced relative to a fully balanced design.

On multiple testing, I also found the authors' response confusing ("The multiple testing was not considered because the relative comparison (e.g. hybrid versus site)…"), and it would help to be more explicit. In Table 5, are you not reporting significance tests for variance components (at least hybrid, site, and hybrid × site, I think) separately for 10 GA–tissue combinations, i.e. at least 30 formal tests? Then later there are five separate GA1–GA4 regression/correlation tests, one for each tissue. At present these nominal p-values appear to be interpreted directly, and merely noting significance at multiple levels (5%, 1%, and 0.1%) does not really address the multiplicity issue. I am not suggesting that a formal multiple-testing correction is needed in what is partly an exploratory study, but the manuscript should at least acknowledge the number of related tests being carried out and temper the interpretation of marginal findings accordingly.

Response: The revisions were made in the manuscript to address this concern. The primary objective of this analysis was to describe how variation in GA levels was partitioned among model components, rather than to perform confirmatory hypothesis testing. For each GA-by-tissue combination, variance components associated with hybrid, site, replicate nested within site, and the hybrid-by-site interaction were evaluated using individual likelihood ratio tests, resulting in 40 variance-component tests in total. No multiplicity adjustment was applied because these tests were used for an exploratory assessment of variance structure. Accordingly, the resulting p-values should be interpreted descriptively, together with the estimated variance components and their relative contributions to total variation. Although conducting 40 tests increases the chance of false positives, effects supported by both substantial estimated variance contributions and very small p-values provide stronger evidence of meaningful structure in the data than marginally significant effects.

On confidence intervals, I do not understand why the authors regard the log scale as an obstacle (since variance components are naturally on that scale anyway). Confidence intervals are precisely what convey uncertainty in weakly identified settings, such as stemming from unbalanced design.

Response: Variance components were estimated by fitting an all-random model that included hybrid, site, replicate nested within site, the hybrid-by-site interaction, and residual error. This model was specified to decompose total response variation and quantify the relative contributions of environmental, design-related, and interaction sources across sites. Because the primary objective was to identify the main drivers of variability, results are presented as variance-component point estimates and their relative percentage contributions. These measures provide the most direct and practical basis for evaluating the importance of each source of variation.

Reviewer #3: This manuscript reports a validated high-throughput LC-MS/MS workflow and uses it to quantify the bioactive gibberellins GA1 and GA4 in five maize tissues from ten commercial hybrids grown at six U.S. field sites, partitioning the variation into genetic (hybrid), environmental (site), and interaction components. The analytical method appears sound and the geographic breadth is a genuine strength, so the descriptive dataset has value as a baseline resource.

I have read the previous review round. The authors have made constructive changes — the Results and Discussion are now separated, the experimental design and sampling are described in more detail (Table S1), the GA1–GA4 correlation discussion has been appropriately tempered to acknowledge its correlational nature, and the closing claims have been softened to "preliminary insights." I appreciate these revisions.

That said, my main remaining concern is that the conclusions still reach beyond what the measured data can support, and this stems in large part from what was measured. Only the two bioactive end-products (GA1, GA4) were quantified. A steady-state pool reflects the balance of biosynthesis, 2β-hydroxylation-mediated inactivation, and conjugation, so the present data cannot attribute any observed change to a specific step. This is particularly problematic for the central claim that site (i.e., environment) is the dominant factor: the manuscript cannot say whether a site-associated change in GA1/GA4 arises from altered biosynthetic flux or from altered inactivation.

Maize is an early-13-hydroxylation (GA1-type) species, in which GA1 is inactivated to GA8 and GA4 to GA34 by GA2ox. At minimum, the authors must (i) state this limitation explicitly and (ii) remove or rephrase "biosynthesis" and "plasticity of the gibberellin pathway" language in favour of "endogenous level/abundance" wherever flux was not measured. To support any mechanistic interpretation of the site effect, quantification of the principal inactivated GAs (GA8, GA34) and key precursors (e.g., GA20, GA9), together with active-to-inactive ratios (GA1/GA8, GA4/GA34), would help indicate whether a given change reflects enhanced synthesis or reduced catabolism —

although, as these are themselves steady-state pools, such ratios remain indirect proxies for the underlying flux.

Response: Thanks for the comments. We revised the Abstract, Introduction, Discussion, and Conclusion by deleting the language such as "biosynthesis," "pathway plasticity," and "regulation". Biosynthetic pathway was not discussed in the paper.

Methods (L167–181). Present the LC gradient as a table including the initial hold, and confirm the steep short segments (e.g., B 26%→55% over 0.1 min) are not transcription errors. Critically, retention times for GA1, GA4, and the internal standards are not reported; please add them (and a representative MRM chromatogram near the LLOQ), as they are needed to judge whether this unusual gradient performs as intended.

Response: Thank you for this helpful suggestion. We have revised the Methods section to present the LC gradient as Table 2, including the initial hold. We confirm that the steep, short gradient segments, such as the change from 26% to 55% B over 0.1 min, are intentional and are not transcription errors.
We have also added the retention times for GA1, GA4, and the corresponding internal standards in Table 3. In addition, we provided a representative MRM chromatogram at the LLOQ (standard 1), as Supporting Information S1 Fig. This added information should allow readers to better assess the chromatographic performance of the method and confirm that the gradient performs as intended.
S1 Fig. The representative chromatogram of LLOQ (standard 1)
Top left: TIC, Top right: XIC of GA1, Bottom left: XIC of GA1-D4 and GA4-D2, Bottom right: XIC of GA4.

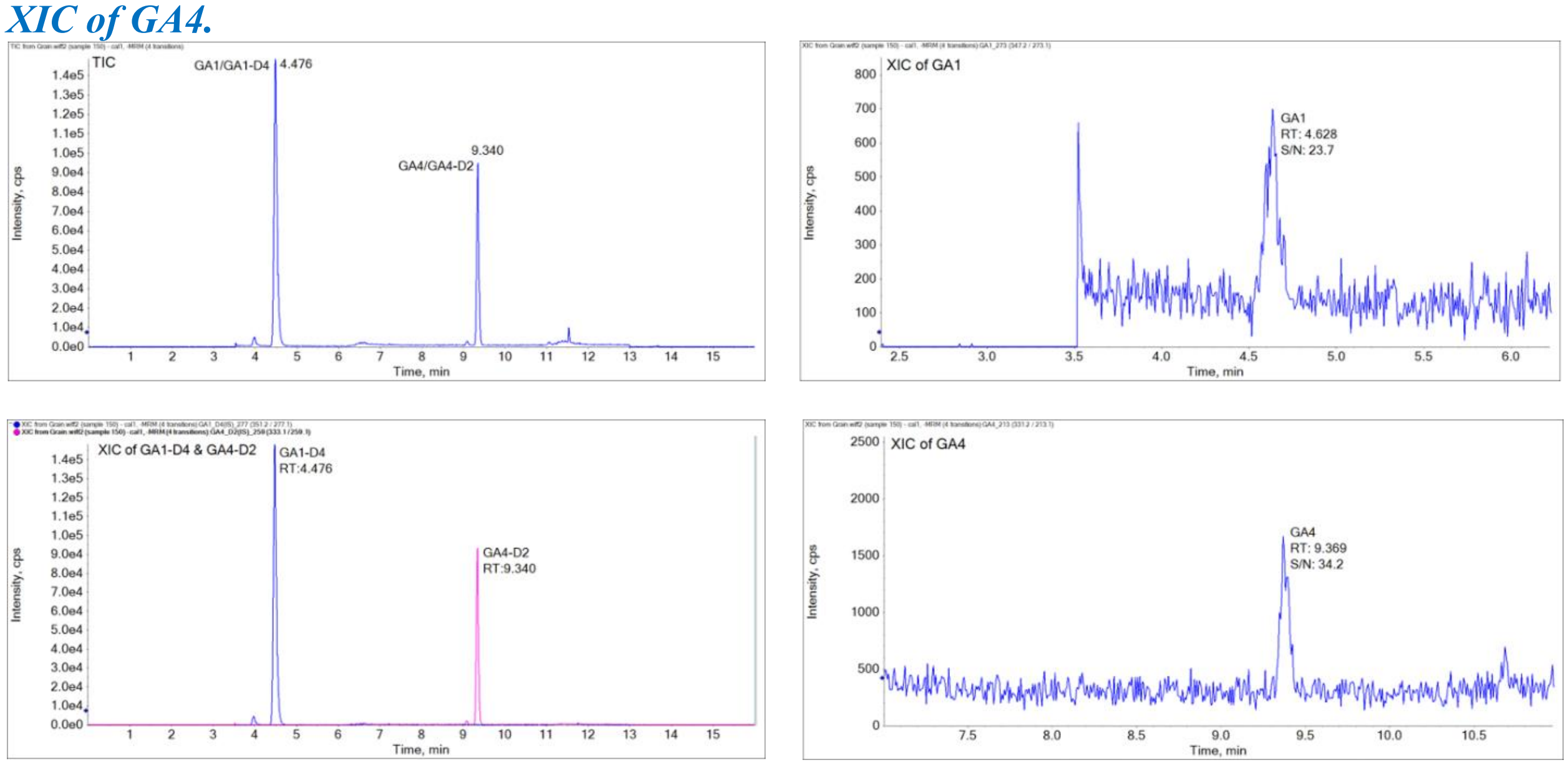

Table 3 (pollen). The text states that pollen data were not reported because of its short stability window (L262–263), and pollen is correctly absent from Tables 4–5 and Figs 2–4. However, Table 3 still lists method-validation data for pollen.

Response: Thank you for raising this point. The pollen results included in Table 3 refer only to the method-validation experiment. We validated the method for pollen along with the other maize tissues, including assessments of accuracy, precision, extraction efficiency, recovery, and benchtop stability; therefore, the validated pollen data are appropriately included in Table 3.
However, pollen had a much shorter benchtop stability window than the other tissues. Although this was manageable during method validation because only a small number of samples were processed, it was not feasible for the large field-sample set. The field study included more samples, and pollen sample preparation could not be completed within the validated stability window. For this reason, we did not consider the field pollen measurements sufficiently reliable for downstream biological interpretation and therefore excluded pollen from Tables 4–5 and Figures 2–4 for field data.

Minor comments
Introduction (L49). "Gibberella fujikuroi" → the accepted name is Fusarium fujikuroi (teleomorph Gibberella fujikuroi).
Response: "Gibberella fujikuroi" was changed to "Fusarium fujikuroi".

Methods (L157). "until about 400 mL of aqueous phase was left" — should be 400 µL.
Response: 400 "mL" was corrected to 400 "µL".

Methods (L152). "Nutating Mixter" → Mixer; and a missing space after "88-861-043)" before "After incubation".
Response: "Nutating Mixter" was changed to "Mixer", and a space was added before "After incubation".

Methods (L168). "AB Sxiex OS" → "AB Sciex OS"
Response: "AB Sxiex OS" was corrected to "AB Sciex OS".

7. PLOS authors have the option to publish the peer review history of their article (what does this mean?). If published, this will include your full peer review and any attached files.

If you choose "no", your identity will remain anonymous but your review may still be made public.

Do you want your identity to be public for this peer review? For information about this choice, including consent withdrawal, please see our Privacy Policy.
Reviewer #1: No
Reviewer #3: No

[NOTE: If reviewer comments were submitted as an attachment file, they will be attached to this email and accessible via the submission site. Please log into your account, locate the manuscript record, and check for the action link "View Attachments". If this link does not appear, there are no attachment files.]

To ensure your figures meet our technical requirements, please review our figure guidelines: https://journals.plos.org/plosone/s/figures
You may also use PLOS's free figure tool, NAAS, to help you prepare publication quality figures: https://journals.plos.org/plosone/s/figures#loc-tools-for-figure-preparation.
NAAS will assess whether your figures meet our technical requirements by comparing each figure against our figure specifications.
