# Supplementary material for: Natural variation of bioactive gibberellin levels in maize tissues across North America
Source: PLoS One. 2026 Oct 7;21(10):e0340156. doi: 10.1371/journal.pone.0340156 (PMC13645078; doi:10.1371/journal.pone.0340156)
Supplement: Supplementary file 3 [file pone.0340156.s001.docx]

**S1 Table. Summary of sample type and collection sampling information.**

| Sample Type | Pooled Sample Amount per Plot | Number of Plants Sampled per Plot | Sampling Details | Collection Growth Stage | Growth Stage Description |
| --- | --- | --- | --- | --- | --- |
| Leaf | 60 leaves | 60 | Approximately 15 cm of most recently collared leaf | V2-V4 | A plant with 2 to 4 leaf collars |
| Root | 8 roots | 8 | All below-ground root tissue, thoroughly washed | V2-V4 | A plant with 2 to 4 leaf collars |
| Stalk | 4 stalks | 4 | All above-ground stalk tissue with leaf blade, leaf sheath, ear, and tassel removed | V10-V12 | A plant with 10 to 12 leaf collars |
| Pollen | 10 mL | Highly variable based on environmental conditions, though typically >10 | Fresh pollen with all anthers removed | Pollen shed | A plant that is actively shedding pollen |
| Forage | 1 plant | 1 | All above-ground plant tissue | R5, early dent | Most kernels are dented and contain approximately 55% moisture |
| Grain | 1 kg | Minimum of 4 | Shelled grain dried to ≤15.5% moisture prior to shipping and storage | R6 | Physiological maturity; black layer visible at base of kernels |
