# Supplementary material for: Natural variation of bioactive gibberellin levels in maize tissues across North America
Source: PLoS One. 2026 Oct 7;21(10):e0340156. doi: 10.1371/journal.pone.0340156 (PMC13645078; doi:10.1371/journal.pone.0340156)
Supplement: Supplementary file 4 [file pone.0340156.s002.docx]

**S2 Table. Value assigned to non-detection (ND) data points for statistical analysis**

| GA | Tissue | Detected sample number | Minimum value (pmol/g) | Replaced value for ND data points |
| --- | --- | --- | --- | --- |
| GA_1_ | Forage | 50 | 0.705 | 0.35 |
| GA_1_ | Grain | 65 | 0.106 | 0.05 |
| GA_1_ | Leaf | 50 | 0.831 | 0.42 |
| GA_1_ | Root | 45 | 0.223 | 0.11 |
| GA_1_ | Stalk | 68 | 0.927 | - |
| GA_4_ | Forage | 62 | 0.124 | 0.06 |
| GA_4_ | Grain | 68 | 0.312 | - |
| GA_4_ | Leaf | 46 | 0.104 | 0.05 |
| GA_4_ | Root | 66 | 0.200 | 0.10 |
| GA_4_ | Stalk | 68 | 0.168 | - |

 Note: “-” denotes there were no ND data points
