# Supplementary material for: Natural variation of bioactive gibberellin levels in maize tissues across North America
Source: PLoS One. 2026 Oct 7;21(10):e0340156. doi: 10.1371/journal.pone.0340156 (PMC13645078; doi:10.1371/journal.pone.0340156)
Supplement: Supplementary file 5 [file pone.0340156.s007.docx]

Dear Academic editor,

We are grateful for the opportunity to refine our work and resubmit it for your consideration. Please find our responses in blue below. Please note, we included all three response to reviewers in this document. Please let us know if there is any additional information you need or if further clarification is required.

Thank you once again for your guidance and support throughout the revision process.

Warm regards,

Yanfei Wang

**Response to reviewer 3**

PONE-D-25-66749R1

Natural variation of gibberellin levels in maize tissues across North America Dr. Yanfei Wang

Dear Dr. Wang,

We've checked your submission and before we can proceed, we need you to address the following issues:

1. Thank you for updating Figure 1. The revised illustrations are noted. Could you please confirm that these new images were created by the authors without the use of AI-generation tools? This is simply to ensure our records are accurate given the earlier versions.

Response: Figure 1 in the revised manuscript is an **original author-created illustration** of our workflow that can be licensed under CC BY 4.0. The submitted figure was prepared by the authors using standard drawing tools and was **not copied or traced** from any AI-generated image. No AI-generation tools were used in creating this figure.

We look forward to hearing from you.

We've returned your manuscript to your account. Please resolve these issues and resubmit your manuscript within 21 days. If you need more time, please email the journal office at [plosone@plos.org](mailto:plosone@plos.org). We are happy to grant extensions of up to one month past this due date. If we do not hear from you within 21 days, we will withdraw your manuscript.

Please log on to PLOS Editorial Manager at <https://eur03.safelinks.protection.outlook.com/?url=https%3A%2F%2Fwww.editorialmanager.com%2Fpone%2F&data=05%7C02%7C%7C5da17ce380344a69416208debc7966d0%7Cfcb2b37b5da0466b9b830014b67a7c78%7C0%7C0%7C639155426756799707%7CUnknown%7CTWFpbGZsb3d8eyJFbXB0eU1hcGkiOnRydWUsIlYiOiIwLjAuMDAwMCIsIlAiOiJXaW4zMiIsIkFOIjoiTWFpbCIsIldUIjoyfQ%3D%3D%7C0%7C%7C%7C&sdata=S81mgknf8OqG7qd2yCZZSLUw13iRoSajfQTTG1Ds6js%3D&reserved=0> to access your manuscript. You will find your manuscript in the 'Submissions Sent Back to Author' link under the New Submissions menu. Be sure to remove your previous manuscript file if you are uploading a new file in response to these requests. After you've made the changes requested above, please be sure to view and approve the revised PDF after rebuilding the PDF to complete the resubmission process.

We are requesting these changes to comply with the PLOS ONE submission guidelines (<https://eur03.safelinks.protection.outlook.com/?url=https%3A%2F%2Fjournals.plos.org%2Fplosone%2Fs%2Fsubmission-guidelines&data=05%7C02%7C%7C5da17ce380344a69416208debc7966d0%7Cfcb2b37b5da0466b9b830014b67a7c78%7C0%7C0%7C639155426756872182%7CUnknown%7CTWFpbGZsb3d8eyJFbXB0eU1hcGkiOnRydWUsIlYiOiIwLjAuMDAwMCIsIlAiOiJXaW4zMiIsIkFOIjoiTWFpbCIsIldUIjoyfQ%3D%3D%7C0%7C%7C%7C&sdata=GMhbQcjkuCCgcxzxa0vt1ktIfpE0mIrhunlZrJxPYPw%3D&reserved=0>). Please note that we won't send your manuscript for review until you have resolved the above requests.

Thank you for submitting your work to PLOS ONE and supporting our mission of Open Science.

Kind regards,

Gema Queenie Salem

PLOS One

**Response to reviewer 2**

ONE-D-25-66749R1

Natural variation of gibberellin levels in maize tissues across North America Dr. Yanfei Wang

Dear Dr. Wang,

We've checked your submission and before we can proceed, we need you to address the following issues:

1. We note that your author list was updated during the revision process. In order to add or remove authors or update the order of the author byline after initial submission, we ask that authors complete an Authorship Change Request form. You may review our full authorship change policy and download the Authorship Change Request form here: <https://eur03.safelinks.protection.outlook.com/?url=https%3A%2F%2Fjournals.plos.org%2Fplosone%2Fs%2Fauthorship%23loc-authorship-changes&data=05%7C02%7C%7C4eafb8e3435147c5cdee08dea4c00395%7Cfcb2b37b5da0466b9b830014b67a7c78%7C0%7C0%7C639129341761324165%7CUnknown%7CTWFpbGZsb3d8eyJFbXB0eU1hcGkiOnRydWUsIlYiOiIwLjAuMDAwMCIsIlAiOiJXaW4zMiIsIkFOIjoiTWFpbCIsIldUIjoyfQ%3D%3D%7C0%7C%7C%7C&sdata=fGKs8plcZP7khUMVwLM8LSBxBz5j4MoNO7L7XFq5oYA%3D&reserved=0>.

Please fill out all required sections of the form. If you are adding or removing more than 2 authors, you can complete multiple forms and submit as many forms as needed to reflect the updates. Please return the form(s) as an attachment by emailing [plosone@plos.org](mailto:plosone@plos.org) or by uploading it as a submission file labeled with the file type ‘Other’. The form will be reviewed by PLOS staff for approval. Should the changes be approved, we will require written confirmation from all authors confirming that they approve of the changes. Please note that if your manuscript is accepted, we will not be able to complete the publication process without the completed form.

Response: The completed **Authorship Change Request form** has been uploaded as a submission file labeled **“Other.”** In addition, the **signed Authorship Change Request form**, including e-signatures from all authors, is attached under the file type **“Other”** as well.

2. Thank you for including your ethics statement on the online submission form: " Release Location (County, State) USDA EPA/EUP Permit Permit Jefferson County AUTH - 0000233243 524-EUP-117 IA Shelby County AUTH - 0000233243 524-EUP-117 IL

Boone County AUTH - 0000233243 524-EUP-117 IN Adair County AUTH - 0000233243 524-EUP-117 MO York County AUTH - 0000233243 524-EUP-117 NE Miami County AUTH - 0000233243 524-EUP-117 OH ". To help ensure that the wording of your manuscript is suitable for publication, would you please also add this statement at the beginning of the Methods section of your manuscript file.

Response: We have added the ethics statement to the beginning of the **Methods** section of the manuscript, consistent with the information provided in the submission form. Specifically, we added the following statement at line 120: **“Plant materials consisted of conventional crop varieties grown in experimental plots that were collocated with USDA-permitted materials under Authorization No. AUTH-0000233243.”**

## **Maize field trials**

Maize samples from ten commercially available conventional maize hybrids were collected from field trials during the 2023 growing season. Six field trial locations were spread across states representing major maize-growing regions of the United States (Iowa, Illinois, Indiana, Missouri, Nebraska, and Ohio) (Table 1). Plant materials consisted of conventional crop varieties grown in experimental plots that were collocated with USDA permitted materials under Authorization No. AUTH-0000233243.

**Table 1. Field trial locations and maize hybrid names**

| **Site Code** | **County, State** | **Hybrid Name** |
| --- | --- | --- |
| Site 1 | Jefferson County, Iowa | Dekalb DKC58-32 |
|  |  | Lewis 12CV779 |
|  |  | Channel 210-79 |
| Site 2 | Shelby County, Illinois | Stone 6290 |
|  |  | Hubner H2390 |
|  |  | Dekalb DKC65-92 |
| Site 3 | Boone County, Indiana | Dekalb DKC63-58 |
|  |  | Hubner H2390 |
| Site 4 | Adair County, Missouri | Dekalb DKC58-32 |
|  |  | Lewis 12CV779 |
|  |  | Stone 6070 |
| Site 5 | York County, Nebraska | Stone 6290 |
|  |  | Hubner H2390 |
|  |  | Dekalb DKC64-32 |
| Site 6 | Miami County, Ohio | Dekalb DKC58-32 |
|  |  | Stewart 12CV209 |
|  |  | Channel 210-79 |

3. Thank you for your explanation regarding Figure 1 and for confirming that the original version was generated using your internal ChatGPT system and has now been manually redrawn to avoid licensing issues.

PLOS has specific guidance for figures that involve generative AI tools. To ensure that Figure 1 is fully compatible with the CC BY 4.0 license, could you please clarify the following:

- Tool and terms of use

Please specify which generative AI service your “internal ChatGPT” is based on (for example, an enterprise instance of OpenAI’s ChatGPT, or another provider), and provide the direct URL to the Terms of Use governing that service.

- Relationship between the AI output and the current figure

Please confirm whether the newly redrawn Figure 1 was created without directly copying or tracing any part of the AI‑generated image, and that the current version can be considered an original author‑created illustration that you are entitled to license under CC BY 4.0.

- Figure legend disclosure (if applicable)

If any elements of the current figure were materially based on an AI‑generated output (e.g., used as a design/template that has been refined), PLOS requires that this be clearly disclosed in the figure legend (for example: “Figure concept was initially drafted using a generative AI tool (internal ChatGPT); this version was manually redrawn by the authors.”).

Once we have this clarification, we’ll be able to confirm that Figure 1 complies with PLOS copyright and AI‑use requirements.

Response: Figure 1 in the revised manuscript is an **original author-created illustration** of our workflow that can be licensed under CC BY 4.0. The submitted figure was prepared by the authors using standard drawing tools and was **not copied or traced** from any AI-generated image.

We've returned your manuscript to your account. Please resolve these issues and resubmit your manuscript within 21 days. If you need more time, please email the journal office at [plosone@plos.org](mailto:plosone@plos.org). We are happy to grant extensions of up to one month past this due date. If we do not hear from you within 21 days, we will withdraw your manuscript.

Please log on to PLOS Editorial Manager at <https://eur03.safelinks.protection.outlook.com/?url=https%3A%2F%2Fwww.editorialmanager.com%2Fpone%2F&data=05%7C02%7C%7C4eafb8e3435147c5cdee08dea4c00395%7Cfcb2b37b5da0466b9b830014b67a7c78%7C0%7C0%7C639129341761353939%7CUnknown%7CTWFpbGZsb3d8eyJFbXB0eU1hcGkiOnRydWUsIlYiOiIwLjAuMDAwMCIsIlAiOiJXaW4zMiIsIkFOIjoiTWFpbCIsIldUIjoyfQ%3D%3D%7C0%7C%7C%7C&sdata=ewSYp6I4078AA%2B4zwEjBVhQRuRfaJd7VHyFLGjl5r%2Fg%3D&reserved=0> to access your manuscript. You will find your manuscript in the 'Submissions Sent Back to Author' link under the New Submissions menu. Be sure to remove your previous manuscript file if you are uploading a new file in response to these requests. After you've made the changes requested above, please be sure to view and approve the revised PDF after rebuilding the PDF to complete the resubmission process.

We are requesting these changes to comply with the PLOS ONE submission guidelines (<https://eur03.safelinks.protection.outlook.com/?url=https%3A%2F%2Fjournals.plos.org%2Fplosone%2Fs%2Fsubmission-guidelines&data=05%7C02%7C%7C4eafb8e3435147c5cdee08dea4c00395%7Cfcb2b37b5da0466b9b830014b67a7c78%7C0%7C0%7C639129341761373970%7CUnknown%7CTWFpbGZsb3d8eyJFbXB0eU1hcGkiOnRydWUsIlYiOiIwLjAuMDAwMCIsIlAiOiJXaW4zMiIsIkFOIjoiTWFpbCIsIldUIjoyfQ%3D%3D%7C0%7C%7C%7C&sdata=JIuAUGPyJjTDlIOT%2BVDU4P3CypeNInl2az924nZi9cE%3D&reserved=0>). Please note that we won't send your manuscript for review until you have resolved the above requests.

Thank you for submitting your work to PLOS ONE and supporting our mission of Open Science.

Kind regards,

Gema Queenie Salem

PLOS One

**Response to reviewer 1**

PONE-D-25-66749

Natural variation of gibberellin levels in maize tissues across North America

PLOS One

Dear Dr. Wang,

Thank you for submitting your manuscript to PLOS ONE. After careful consideration, we feel that it has merit but does not fully meet PLOS ONE’s publication criteria as it currently stands. Therefore, we invite you to submit a revised version of the manuscript that addresses the points raised during the review process.

Please submit your revised manuscript by Apr 19 2026 11:59PM. If you will need more time than this to complete your revisions, please reply to this message or contact the journal office at [plosone@plos.org](mailto:plosone@plos.org). When you're ready to submit your revision, log on to <https://www.editorialmanager.com/pone/> and select the 'Submissions Needing Revision' folder to locate your manuscript file.

Please include the following items when submitting your revised manuscript:

- A letter that responds to each point raised by the academic editor and reviewer(s). You should upload this letter as a separate file labeled 'Response to Reviewers'.
- A marked-up copy of your manuscript that highlights changes made to the original version. You should upload this as a separate file labeled 'Revised Manuscript with Track Changes'.
- An unmarked version of your revised paper without tracked changes. You should upload this as a separate file labeled 'Manuscript'.

If you would like to make changes to your financial disclosure, please include your updated statement in your cover letter. Guidelines for resubmitting your figure files are available below the reviewer comments at the end of this letter.

If applicable, we recommend that you deposit your laboratory protocols in protocols.io to enhance the reproducibility of your results. Protocols.io assigns your protocol its own identifier (DOI) so that it can be cited independently in the future. For instructions see: [https://journals.plos.org/plosone/s/submission-guidelines#loc-laboratory-protocols](https://eur03.safelinks.protection.outlook.com/?url=https%3A%2F%2Ftrack.editorialmanager.com%2FCL0%2Fhttps%3A%252F%252Fjournals.plos.org%252Fplosone%252Fs%252Fsubmission-guidelines%2523loc-laboratory-protocols%2F1%2F010f019cc125ad6c-294c59e9-74b9-4907-865c-a741ee48af1d-000000%2F7ujBe9NjBegGwp0bmNEajZ1RpV53VBe1DCqMAk-B8iE%3D253&data=05%7C02%7C%7Cd3f9efc377ce4b00c8ab08de7b2ec83f%7Cfcb2b37b5da0466b9b830014b67a7c78%7C0%7C0%7C639083638484801603%7CUnknown%7CTWFpbGZsb3d8eyJFbXB0eU1hcGkiOnRydWUsIlYiOiIwLjAuMDAwMCIsIlAiOiJXaW4zMiIsIkFOIjoiTWFpbCIsIldUIjoyfQ%3D%3D%7C0%7C%7C%7C&sdata=pGomy7eC30n8jQH80QoiUocr0B90GWDMib2QxshIF1I%3D&reserved=0). Additionally, PLOS ONE offers an option for publishing peer-reviewed Lab Protocol articles, which describe protocols hosted on protocols.io. Read more information on sharing protocols at [https://plos.org/protocols?utm_medium=editorial-email&utm_source=authorletters&utm_campaign=protocols](https://eur03.safelinks.protection.outlook.com/?url=https%3A%2F%2Ftrack.editorialmanager.com%2FCL0%2Fhttps%3A%252F%252Fplos.org%252Fprotocols%253Futm_medium%3Deditorial-email%2526utm_source%3Dauthorletters%2526utm_campaign%3Dprotocols%2F1%2F010f019cc125ad6c-294c59e9-74b9-4907-865c-a741ee48af1d-000000%2FYKdttVXN7H-TYPlZXKfpdL_edTujuDu0QjTrg69ijPs%3D253&data=05%7C02%7C%7Cd3f9efc377ce4b00c8ab08de7b2ec83f%7Cfcb2b37b5da0466b9b830014b67a7c78%7C0%7C0%7C639083638484833220%7CUnknown%7CTWFpbGZsb3d8eyJFbXB0eU1hcGkiOnRydWUsIlYiOiIwLjAuMDAwMCIsIlAiOiJXaW4zMiIsIkFOIjoiTWFpbCIsIldUIjoyfQ%3D%3D%7C0%7C%7C%7C&sdata=21sgSyLi%2FlpU3Q7iuT4vHDZ6dUuXMCSmcK4jI1eA28E%3D&reserved=0).

We look forward to receiving your revised manuscript.

Kind regards,

Tobias Isaac Baskin
Academic Editor
PLOS One

**Response:** Thanks for the opportunity to refine our work and resubmit it for your consideration, the following items have been addressed when submitting the revised manuscript:

- A letter that responds to each point raised by the academic editor and reviewer(s) as a separate file labeled 'Response to Reviewers' has been uploaded
- A 'Revised Manuscript with Track Changes'. has been uploaded
- An unmarked version of the revised paper without tracked changes labeled 'Manuscript' has been uploaded
- An updated Funding Statement and Competing Interests Statement have been included in cover letter.
- Figure file has been updated
- The completed **Authorship Change Request form** has been uploaded as a submission file labeled **“Other.”** In addition, the **signed Authorship Change Request form**, including e-signatures from all authors, is attached under the file type **“Other”** As well.

Journal Requirements:

When submitting your revision, we need you to address these additional requirements.

1.Please ensure that your manuscript meets PLOS ONE's style requirements, including those for file naming. The PLOS ONE style templates can be found at

<https://journals.plos.org/plosone/s/file?id=wjVg/PLOSOne_formatting_sample_main_body.pdf> and

[https://journals.plos.org/plosone/s/file?id=ba62/PLOSOne_formatting_sample_title_authors_affiliations.pdf](https://eur03.safelinks.protection.outlook.com/?url=https%3A%2F%2Ftrack.editorialmanager.com%2FCL0%2Fhttps%3A%252F%252Fjournals.plos.org%252Fplosone%252Fs%252Ffile%253Fid%3Dba62%252FPLOSOne_formatting_sample_title_authors_affiliations.pdf%2F1%2F010f019cc125ad6c-294c59e9-74b9-4907-865c-a741ee48af1d-000000%2FoXR7r-PowX4f48Whx52poEHUYqXtNTycLETAY7WSIwA%3D253&data=05%7C02%7C%7Cd3f9efc377ce4b00c8ab08de7b2ec83f%7Cfcb2b37b5da0466b9b830014b67a7c78%7C0%7C0%7C639083638484852999%7CUnknown%7CTWFpbGZsb3d8eyJFbXB0eU1hcGkiOnRydWUsIlYiOiIwLjAuMDAwMCIsIlAiOiJXaW4zMiIsIkFOIjoiTWFpbCIsIldUIjoyfQ%3D%3D%7C0%7C%7C%7C&sdata=sQeBnwOUNUGKuYitmw9hJdLt9GrVPNfXP8jWf64ckLA%3D&reserved=0)

**Response:** We have reviewed the PLOS ONE style templates provided in the links and made the necessary adjustments to our manuscript, including body formatting and file naming conventions.

2. Please note that funding information should not appear in any section or other areas of your manuscript. We will only publish funding information present in the Funding Statement section of the online submission form. Please remove any funding-related text from the manuscript.

**Response:** The funding statement has been removed from the manuscript. Updated Financial disclosure statement has been submitted online.

Funding for this research was provided by Bayer CropScience LLC.

The funder provided support in the form of salaries for authors XG, NS, CJ, JG, EA, CD, CP, SS, YW, but did not have any additional role in the study design, data collection and analysis, decision to publish, or preparation of the manuscript. The specific roles of these authors are articulated in the ‘author contributions’ section.

3. Thank you for stating the following in the Competing Interests section:

“The authors have declared that no competing interests exist.”

We note that one or more of the authors are employed by a commercial company: Bayer CropScience LLC

1.        Please provide an amended Funding Statement declaring this commercial affiliation, as well as a statement regarding the Role of Funders in your study. If the funding organization did not play a role in the study design, data collection and analysis, decision to publish, or preparation of the manuscript and only provided financial support in the form of authors' salaries and/or research materials, please review your statements relating to the author contributions, and ensure you have specifically and accurately indicated the role(s) that these authors had in your study. You can update author roles in the Author Contributions section of the online submission form.

Please also include the following statement within your amended Funding Statement.

“The funder provided support in the form of salaries for authors [insert relevant initials], but did not have any additional role in the study design, data collection and analysis, decision to publish, or preparation of the manuscript. The specific roles of these authors are articulated in the ‘author contributions’ section.”

If your commercial affiliation did play a role in your study, please state and explain this role within your updated Funding Statement.

**Response:** The funding statement has been removed from the manuscript. Updated Financial disclosure statement has been submitted online.

Funding for this research was provided by Bayer CropScience LLC.

The funder provided support in the form of salaries for authors XG, NS, CJ, JG, EA, CD, CP, SS, YW, but did not have any additional role in the study design, data collection and analysis, decision to publish, or preparation of the manuscript. The specific roles of these authors are articulated in the ‘author contributions’ section.

2. Please also provide an updated Competing Interests Statement declaring this commercial affiliation along with any other relevant declarations relating to employment, consultancy, patents, products in development, or marketed products, etc.

Within your Competing Interests Statement, please confirm that this commercial affiliation does not alter your adherence to all PLOS ONE policies on sharing data and materials by including the following statement: "This does not alter our adherence to  PLOS ONE policies on sharing data and materials.” (as detailed online in our guide for authors <http://journals.plos.org/plosone/s/competing-interests>) . If this adherence statement is not accurate and  there are restrictions on sharing of data and/or materials, please state these. Please note that we cannot proceed with consideration of your article until this information has been declared.

Please include both an updated Funding Statement and Competing Interests Statement in your cover letter. We will change the online submission form on your behalf.

**Response:** An updated Funding Statement and Competing Interests Statement have been included in cover letter. updated Competing Interests Statement are as below.

**Updated Competing Interests Statement**

A patent application has been filed for this work. This does not alter our adherence to  PLOS ONE policies on sharing data and materials.

4. Please include your full ethics statement in the ‘Methods’ section of your manuscript file. In your statement, please include the full name of the IRB or ethics committee who approved or waived your study, as well as whether or not you obtained informed written or verbal consent. If consent was waived for your study, please include this information in your statement as well.

**Response:** The research applied in this study involved only plant species and did not include human subjects or animals, ethics approval was not applicable. We have added the ethics statement to the beginning of the **Methods** section of the manuscript, consistent with the information provided in the submission form. Specifically, we added the following statement at line 120: **“Plant materials consisted of conventional crop varieties grown in experimental plots that were collocated with USDA-permitted materials under Authorization No. AUTH-0000233243.”**

5. We note that Figure 1 in your submission contain copyrighted images. All PLOS content is published under the Creative Commons Attribution License (CC BY 4.0), which means that the manuscript, images, and Supporting Information files will be freely available online, and any third party is permitted to access, download, copy, distribute, and use these materials in any way, even commercially, with proper attribution. For more information, see our copyright guidelines: <http://journals.plos.org/plosone/s/licenses-and-copyright>.

 We require you to either (1) present written permission from the copyright holder to publish these figures specifically under the CC BY 4.0 license, or (2) remove the figures from your submission:

 1. You may seek permission from the original copyright holder of Figure 1 to publish the content specifically under the CC BY 4.0 license.

 We recommend that you contact the original copyright holder with the Content Permission Form (<http://journals.plos.org/plosone/s/file?id=7c09/content-permission-form.pdf>) and the following text:

“I request permission for the open-access journal PLOS ONE to publish XXX under the Creative Commons Attribution License (CCAL) CC BY 4.0 (<http://creativecommons.org/licenses/by/4.0/>). Please be aware that this license allows unrestricted use and distribution, even commercially, by third parties. Please reply and provide explicit written permission to publish XXX under a CC BY license and complete the attached form.”

 Please upload the completed Content Permission Form or other proof of granted permissions as an "Other" file with your submission.

 In the figure caption of the copyrighted figure, please include the following text: “Reprinted from [ref] under a CC BY license, with permission from [name of publisher], original copyright [original copyright year].”

 2. If you are unable to obtain permission from the original copyright holder to publish these figures under the CC BY 4.0 license or if the copyright holder’s requirements are incompatible with the CC BY 4.0 license, please either i) remove the figure or ii) supply a replacement figure that complies with the CC BY 4.0 license. Please check copyright information on all replacement figures and update the figure caption with source information. If applicable, please specify in the figure caption text when a figure is similar but not identical to the original image and is therefore for illustrative purposes only.

**Response:** Figure 1 in the revised manuscript is an **original author-created illustration** of our workflow that can be licensed under CC BY 4.0. The submitted figure was prepared by the authors using standard drawing tools and was **not copied or traced** from any AI-generated image.

6. We notice that your supplementary tables are included in the manuscript file. Please remove them and upload them with the file type 'Supporting Information'. Please ensure that each Supporting Information file has a legend listed in the manuscript after the references list.

**Response:** Supplementary tables are removed and uploaded with the file type 'Supporting Information'. The supporting information files have legend listed in the manuscript after the references list.

7. If the reviewer comments include a recommendation to cite specific previously published works, please review and evaluate these publications to determine whether they are relevant and should be cited. There is no requirement to cite these works unless the editor has indicated otherwise.

**Response:** We have carefully evaluated the recommended publications that address the spatial distributions of gibberellins in growing organs and the impact of environmental factors on gibberellins levels. Reference 7 has been previously cited for the spatial distribution of gibberellins in our introduction. We agreed with the reviewer that relevant references provide important context and insights that complement our study. Therefore, we incorporated additional 4 relevant citations into the introduction to enhance the literature review and address the reviewer's concerns.

Reference 7

Rizza A, Jones AM. The makings of a gradient: spatiotemporal distribution of gibberellins in plant development. Current Opinion in Plant Biology, 2019, 47:9-15.

Reference 4

Ubeda-Tomás S, Federici F, Casimiro I, Beemster GTS, Bhalerao R, Swarup R, et al. Gibberellin Signaling in the Endodermis Controls Root Meristem Size. Current Biology. 2009;19(14):1194-9.

Reference 8

Nelissen H, Rymen B, Jikumaru Y, Demuynck K, Van Lijsebettens M, Kamiya Y, et al. A local maximum in gibberellin levels regulates maize leaf growth by spatial control of cell division. Current Biology. 2012;22(13):1183-7.

Reference 14

Rizza A, [Walia](https://www.nature.com/articles/s41477-017-0021-9#auth-Ankit-Walia-Aff1) A, [Lanquar](https://www.nature.com/articles/s41477-017-0021-9#auth-Viviane-Lanquar-Aff2) V, [Frommer](https://www.nature.com/articles/s41477-017-0021-9#auth-Wolf_B_-Frommer-Aff2-Aff3-Aff4) WB, [Alexander M.Jones.](https://www.nature.com/articles/s41477-017-0021-9#auth-Alexander_M_-Jones-Aff1-Aff2) In vivo gibberellin gradients visualized in rapidly elongating tissues, [Nature Plants](https://www.nature.com/nplants) 2017, 3: 803–813

Reference 15

Binenbaum J, Weinstain R, Shani E. Gibberellin Localization and Transport in Plants, Trends in Plant Science, 2018, 23: 410-421

Additional Editor Comments:

Thank you for submitting your work to our journal. Your work has been read by two reviewers who both found substantial flaws. Reviewer 1 is a statistician. This reviewers points out places with missing or confusing information with respect to experimental design and interpretation. These points need addressing before a reader can have confidence in your approach. Reviewer 2 is a biologist; they point out issues with respect to plant development. Gibberellin varies within an organ and over time as the organ develops. In the best case, this axis would be sampled but at the very least it needs to be addressed.

I have a three additional points. First, please spell out gibberellin when used to refer to the hormone in general. Gibberellin is a lovely word, echoing as it does the word gibberish, inspired by the foolish seedling disease that led to the hormone's discovery. Scientists have come to think that they are supposed to abbreviate words of their discourse but readers find this practice unhelpful. We prefer to read words over symbols. Familiarity is irrelevant.

The other day, I saw this with respect to current culture war on-going in the U.S.A. “If you oppose DEI you should have to use the full phase instead of the letters. Be brave and say, I oppose diversity, equity, and inclusion. Making it an acronym transforms it into a thought-terminating cliche.” While thought “terminating” is perhaps an exaggeration, thought “suppressing” is correct. As scientists we are urged to communicate better; the first and easiest thing we can do toward that end is to write with words.

In the case of gibberellin, a compromise can be to use GA1 (etc.) for the specific molecule (although I encourage you to bite this bullet and use gibberellin-1).

**Response:** Thank you for your detailed feedback and for the insights provided by the reviewers. We appreciate the opportunity to address the substantial concerns raised regarding our manuscript.

**Addressing Reviewer 1's Comments**: We acknowledge the points raised by Reviewer 1 regarding the experimental design and result interpretation. We have reviewed the sections highlighted and provided additional clarity and detail to ensure that our methodology is transparent and comprehensible. This information was included in Supplementary 1 (S1 table) and a more thorough explanation of our statistical analyses and their relevance to our findings in results and discussion.

**Addressing Reviewer 2's Comments**: We recognize the importance of addressing the variability of gibberellin levels within an organ and over time during development. We have incorporated additional relevant citations (4,8,14,15) into the introduction to enhance the literature review and address the reviewer's concerns, highlighting how it impacts our results and interpretations.

Reference 4

Ubeda-Tomás S, Federici F, Casimiro I, Beemster GTS, Bhalerao R, Swarup R, et al. Gibberellin Signaling in the Endodermis Controls Root Meristem Size. Current Biology. 2009;19(14):1194-9.

Reference 8

Nelissen H, Rymen B, Jikumaru Y, Demuynck K, Van Lijsebettens M, Kamiya Y, et al. A local maximum in gibberellin levels regulates maize leaf growth by spatial control of cell division. Current Biology. 2012;22(13):1183-7.

Reference 14

[Rizza](https://www.nature.com/articles/s41477-017-0021-9#auth-Annalisa-Rizza-Aff1) A, [Walia](https://www.nature.com/articles/s41477-017-0021-9#auth-Ankit-Walia-Aff1) A, [Lanquar](https://www.nature.com/articles/s41477-017-0021-9#auth-Viviane-Lanquar-Aff2) V, [Frommer](https://www.nature.com/articles/s41477-017-0021-9#auth-Wolf_B_-Frommer-Aff2-Aff3-Aff4) WB, [Alexander M.Jones.](https://www.nature.com/articles/s41477-017-0021-9#auth-Alexander_M_-Jones-Aff1-Aff2) In vivo gibberellin gradients visualized in rapidly elongating tissues, [Nature Plants](https://www.nature.com/nplants) 2017, 3: 803–813

Reference 15

Binenbaum J, Weinstain R, Shani E. Gibberellin Localization and Transport in Plants, Trends in Plant Science, 2018, 23: 410-421

**Spelling Out Gibberellin**: We appreciate your perspective on the use of terminology in scientific writing. In response to your comments, we will spell out "gibberellin" in full when referring to the hormone in general throughout the manuscript. We will use the specific abbreviations (e.g., GA_1_) only when referring to particular molecules, as suggested. We understand the importance of clear communication and will strive to enhance the readability of our work.

Second, PLoS One publishes papers that are valid with no reflection on whether they are important or even interesting. Because most journals want the work to be more than valid, writers tend to pitch the importance of their story. But this gets in the way here. What is needed is simply to introduce the reader to the problem, say what you did to solve it, and what you think it means. There is no need to stretch beyond the straightforward.

**Response:** we have revised the manuscript to ensure that it presents the research in a straightforward manner. Specifically, we have:

1. **Introduce the Problem Clearly**: We succinctly outlined the problem we are addressing without overstating its significance.
2. **Describe Our Approach**: We articulated the methods and approaches we employed to address the problem, ensuring that the description is concise and focused.
3. **Discuss the Implications**: We presented our findings and their implications in a straightforward manner, avoiding any embellishments or overstated claims.

By adopting this more direct approach, we enhanced the clarity and accessibility of our manuscript, ensuring it meets the expectations of your journal.

Third, please use separate results and discussion sections. The combination is useful for a short paper, for example with one figure. But your paper is full-size. Present the results; then discuss. This format has persisted for years because it is helpful.

[Note: HTML markup is below. Please do not edit.]

**Response:** Thanks for the great suggestion. Results and Discussion were separated and organized into two sections in paper.

Reviewers' comments:

Reviewer's Responses to Questions

**Comments to the Author**

1. Is the manuscript technically sound, and do the data support the conclusions?

The manuscript must describe a technically sound piece of scientific research with data that supports the conclusions. Experiments must have been conducted rigorously, with appropriate controls, replication, and sample sizes. The conclusions must be drawn appropriately based on the data presented.

Reviewer #1: Partly

Reviewer #2: Partly

2. Has the statistical analysis been performed appropriately and rigorously?

Reviewer #1: No

Reviewer #2: Yes

3. Have the authors made all data underlying the findings in their manuscript fully available?

The [PLOS Data policy](https://eur03.safelinks.protection.outlook.com/?url=http%3A%2F%2Ftrack.editorialmanager.com%2FCL0%2Fhttp%3A%252F%252Fwww.plosone.org%252Fstatic%252Fpolicies.action%2523sharing%2F1%2F010f019cc125ad6c-294c59e9-74b9-4907-865c-a741ee48af1d-000000%2FXWqpC21HlZFFXc8KyBO0bIvK0Y2NYzlXdEenpZ_CXP4%3D253&data=05%7C02%7C%7Cd3f9efc377ce4b00c8ab08de7b2ec83f%7Cfcb2b37b5da0466b9b830014b67a7c78%7C0%7C0%7C639083638484871560%7CUnknown%7CTWFpbGZsb3d8eyJFbXB0eU1hcGkiOnRydWUsIlYiOiIwLjAuMDAwMCIsIlAiOiJXaW4zMiIsIkFOIjoiTWFpbCIsIldUIjoyfQ%3D%3D%7C0%7C%7C%7C&sdata=pnHFhAjxCHrC4i2Bx2GfeCZJ%2BssYXnxgBdxQqIfbNSA%3D&reserved=0) requires authors to make all data underlying the findings described in their manuscript fully available without restriction, with rare exception (please refer to the Data Availability Statement in the manuscript PDF file). The data should be provided as part of the manuscript or its supporting information, or deposited to a public repository. For example, in addition to summary statistics, the data points behind means, medians and variance measures should be available. If there are restrictions on publicly sharing data—e.g. participant privacy or use of data from a third party—those must be specified.

Reviewer #1: Yes

Reviewer #2: Yes

4. Is the manuscript presented in an intelligible fashion and written in standard English?

PLOS ONE does not copyedit accepted manuscripts, so the language in submitted articles must be clear, correct, and unambiguous. Any typographical or grammatical errors should be corrected at revision, so please note any specific errors here.

Reviewer #1: Yes

Reviewer #2: Yes

5. Review Comments to the Author

Please use the space provided to explain your answers to the questions above. You may also include additional comments for the author, including concerns about dual publication, research ethics, or publication ethics. (Please upload your review as an attachment if it exceeds 20,000 characters)

Reviewer #1: I focus my comments mainly on the statistical aspects of the manuscript. The study benefits from a good spread of geographical locations, and it is sensible in aiming to use a classical experimental design and linear statistical model. However, important details on the design are missing, and too much is wrapped up in the sentence “Each site was planted in a randomized complete block design with four replicated blocks. Normal agronomic practices were followed for each site.” It is unclear what the “unit” of planting was. What, physically, were the four blocks, and how were they established? For the hybrids listed at each particular site, does each hybrid (at that site) appear in each block? What was the plot size and layout, was there any obvious spatial heterogeneity? After growing, how was sampling carried out? How many plants per plot were sampled, and were samples from single plants or pooled? These points matter for interpreting both the structure of the model and the meaning of the variance components.

**Response:** We have added details to the method section in paper and more details “Maize field trials” section in Table S1. Each site was planted in a randomized complete block design with four replicated blocks. Hybrids listed in Table 1 were included in each of the four replicated blocks where they were planted, in addition to other hybrids not associated with this study. Within a block, each hybrid was planted in an approximately 6 m × 6 m plot. Each site received a unique randomization scheme. Blocks at each site were arranged to limit variability in environmental conditions such as soil type, slope, and previous cropping history. Normal agronomic practices were followed for each site. Trial maintenance (fertilization, pesticide use, tillage practices, irrigation, etc.) varied across sites, but all maintenance activities were applied uniformly within a site. Maize samples were collected throughout the growing season and included the following tissues: leaf, root, stalk, pollen, forage, and grain. Tissues collected from each plot were pooled into a single sample. Sampling details and growth stage timing can be found in Table S1.

The authors note (Table 1) the unbalanced design with respect to hybrids (some appear once, some at several sites, none of them appears at all six). There is no obvious or systematic overlap structure, and I assume this was driven by logistical constraints rather than design. Nevertheless, it warrants careful discussion because it strongly affects what can be inferred: in particular, the genotype (hybrid) × environment (site) interaction will be poorly identified. With such an incomplete hybrid–site crossing, the data contain (possibly very) limited information to identify hybrid main effects, site effects, and their interaction.
In the linear model, a log transform is used for the response variable. Some explanation of the motivation would be helpful. There is clearly enormous variation (Table 4), spanning four orders of magnitude for some tissue types, which perhaps drives that choice. But is that range biologically plausible, or does it raise concerns about quantification? The fact that some observations fall below detection limits and need to be replaced by a small non-zero value so that the log transformation is defined further raises questions about sensitivity to the choice of imputed value. A simple sensitivity analysis (replacing the imputed value with something smaller and something larger and checking that the conclusions are robust) would be valuable here.
**Response:** Thanks reviewer for the great question. The goal of this investigation is to estimate the natural variation of gibberellins for variety of hybrids in multiple sites. This imbalance would certainly limit the quantitative estimation of each component and the significance of testing but is not expected to affect a qualitative assessment of e.g. comparing contributions of hybrid and site, or evidence of interactions. Despite the unbalanced hybrid-site design resulting in fewer degrees of freedom for the interaction, the statistical test of the interaction is a type III test and not supposed to be affected by the main effects. In terms of log-transformation, both distributions of the observed response and the residual plots support the choice of the transformation and no evidence of heterogeneity.For the observed response < LOQ (limit of quantitation), replacing with half of LOQ is standard procedure in regulatory. Those replacements are not imputations because the observed responses are in fact between 0 and LOQ not missing, thus in the lower end of the ranges in Figure 4 and Figure 5, and would have little effect on the mean (of four replicates often with only one replacement in one mean) and the correlation in the figure. Some clarification was added to the revision.

For the model itself, eqn. (1) seems broadly reasonable, but because the experimental design is not clearly described, it is difficult to understand exactly what the indices run over and how many parameters the model entails. For example, an interaction term cannot be fitted for a site–hybrid combination for which no data exist (e.g. Jefferson County × Stone 6290). The authors state that they use an “allrandomeffect” model, which is perhaps meant to address this, but then immediately switch to a fixedeffects model (“further investigation was made for the mean changes of individual hybrid across sites… a fixedeffect model was assumed and least-square means were estimated”). It is unclear what fixedeffects model is meant here, i.e., which terms are treated as fixed and which as random. The following paragraph (around line 202) switches back to discussion of random effects. This is confusing, because the distinction between fixed and random effects is not simply a matter of computational convenience but relates to the assumptions and interpretation of the model. The hybrids in question are specific, named commercial hybrids, not a random sample from a hypothetical population of hybrids, so treating them as random requires justification. Even if the SAS procedures return variancecomponent estimates, the standard likelihood/chi-squaredbased testing assumptions (which I believe the SAS procedures will use) are almost certainly unreliable in designs as unbalanced as the present one - and in view of that, the resulting pvalues and variance partitions should be interpreted with caution/caveats. Further, I did not understand the description of the statistical procedures well enough to know whether the authors were alert to, and accounted for, multiple testing.

**Response:** Estimation of the hybrid-site interaction is possible only when more than one reference presents at more than one site. Therefore, Figure 4 was included to visualize the interaction connecting the hybrid means over available sites. In the revised manuscript, three SAS procedures were explicitly explained under the same statistical model assuming no fixed effect, each procedure has some advantages over others which is especially important for the unbalanced data. For example, PROC MIXED is better for an unbiased estimate of the variance component due to the restricted maximum likelihood applied, and PROC GLIMMX can provide more reliable significance test of each component because it is a true likelihood based method and applied the likelihood ratio using probability from the mixed chi-squared distributions (because of the zero boundary of the variance component). The hybrid-site means, however, were obtained from PROC GLM which can provide mean prediction for each level of a random factor. The multiple testing was not considered because the relative comparison (e.g. hybrid versus site) is more important than the individual component estimate. However, in the revised manuscript, the separation was made between the significance at the 5% level, 1% level and 0.1% level..

The discussion how positive correlations between GA₁ and GA₄ concentrations could be attributed to regulatory pathways doesn't seem implausible, but isn't a simpler explanation just that both GA₁ and GA₄ are higher when plants are growing more vigorously, i.e. that overall physiological “health” could be a confounder? And indeed isn't "health" itself substantially environmentally driven by "quality of growing conditions"? (This is consistent with the authors finding that the site effect was very significant; this being field data, the authors could presumably not control for weather variation between sites.) The manuscript moves quickly from correlation to, for example, speculation about shared transcriptional networks which whilst possible, seems quite a leap beyond what the data alone suggest.

**Response:** We thank the reviewer for this insightful comment. We agree that the observed positive GA₁–GA₄ correlations could reflect overall plant vigor/physiological status and environmentally driven differences in growing conditions among sites (consistent with the significant site effects we observed). Although some growth and environmental information was recorded during the field trials, these measures were not sufficiently detailed to support a robust covariate adjustment, so we cannot rule out confounding by vigor or weather. In addition, the GA₁–GA₄ association was also observed when examining residual variation after accounting for hybrid, site, and hybrid × site effects, suggesting that the correlation is less likely to be the result of those experimental factors and strongly in favor of biological connection. Nevertheless, mechanistic interpretations (e.g., shared regulatory pathways or transcriptional networks) remain hypotheses requiring targeted follow-up. We revised the Discussion accordingly to emphasize the correlational nature of these results and to temper mechanistic language.

Some suggested actions:
- clarify the experimental design (describing clearly the "plot"s, block structure, what appears in what blocks, etc)

**Response:** We have added details to the “Maize field trials” section in Table S1.

Each site was planted in a randomized complete block design with four replicated blocks. Hybrids listed in Table 1 were included in each of the four replicated blocks where they were planted, in addition to other hybrids not associated with this study. Within a block, each hybrid was planted in an approximately 6 m × 6 m plot. Each site received a unique randomization scheme. Blocks at each site were arranged to limit variability in environmental conditions such as soil type, slope, and previous cropping history. Normal agronomic practices were followed for each site. Trial maintenance (fertilization, pesticide use, tillage practices, irrigation, etc.) varied across sites, but all maintenance activities were applied uniformly within a site. Maize samples were collected throughout the growing season and included the following tissues: leaf, root, stalk, pollen, forage, and grain. Tissues collected from each plot were pooled into a single sample. Sampling details and growth stage timing can be found in Table S1.

- describe fully the sampling (how many plants, which were selected for measurement, whether there was pooling, etc)

**Response:** We have added details to the “Maize field trials” section in Table S1.

Each site was planted in a randomized complete block design with four replicated blocks. Hybrids listed in Table 1 were included in each of the four replicated blocks where they were planted, in addition to other hybrids not associated with this study. Within a block, each hybrid was planted in an approximately 6 m × 6 m plot. Each site received a unique randomization scheme. Blocks at each site were arranged to limit variability in environmental conditions such as soil type, slope, and previous cropping history. Normal agronomic practices were followed for each site. Trial maintenance (fertilization, pesticide use, tillage practices, irrigation, etc.) varied across sites, but all maintenance activities were applied uniformly within a site. Maize samples were collected throughout the growing season and included the following tissues: leaf, root, stalk, pollen, forage, and grain. Tissues collected from each plot were pooled into a single sample. Sampling details and growth stage timing can be found in Table S1.

- explain the consequences of the design imbalance (related in particular to the hybrid x site interaction)

**Response:** The goal of this investigation is to estimate the natural variation and to compare contributions of hybrid (i.e. genetic) and site (or environment) and their interactions. Unbalanced design is due to nature of this purpose, e.g. not all references adaptable in all sites (e.g. limitation of maturity) and the limited capacity of each site. This imbalance would certainly limit the quantitative estimation of each component and the significance of the testing but is not expected to affect a qualitative assessment of e.g. comparing contributions of hybrid and site, or evidence of interactions. Despite the unbalanced hybrid-site design resulting in fewer degrees of freedom for the interaction, the statistical test of the interaction is a type III test and not supposed to be affected by the main effects. The distributions of the observed response and the residual plots support the choice of the log-normal transformation and there is no evidence of heterogeneity. We revised the paper to reflect it.

- [to help with the preceding bullet:] report CIs, not just significance test results, for the variance components

**Response:** The estimate of the variance component is in the log scale and the comparison can only be made on a relative base rather than the scale of the observed response, thus CIs would have no direct practical use.

- clarify the statistical modelling: at different stages, what is treated as random and what is treated as fixed? (with explanation for these choices)

**Response:** The original version of the manuscript called the fixed effects in visualizing the interaction, which caused some confusion for the change of model assumption. In the revised manuscript, the random effects were assumed in all analyses, but a different SAS procedure PROC GLM was applied to obtain the mean prediction for visualizing the interaction. This was explained in the revision.

- clarify whether there were distinct multiple hypothesis tests, and if so whether multiple testing was accounted for in reporting the significance of the results

**Response:** Multiple comparisons were not considered, but significance was indicated at the 5% level,1% level, and 0.1%.

-discuss/justify the log transformation more, including a discussion of whether the extreme range of experimental measurements is biologically reasonable.

**Response:** The log transformation was based on both distributions of the observed response and the residual after fitting the statistical model. For example, the following are the scattered plots of the observed response GA4 in Grain (there are 4 samples for each hybrid at each site) before and after the log transformation, which clearly indicate that some extreme values before the transformation are due to the scale (log scale or the scale of the observation) and in fact comparable with other responses after the transformation.

- if there is value imputation (for effective zeros in the experimental data) perform a sensitivity analysis that the choice of imputed value isn't too consequential to the conclusions

**Response:** The response <LLOQ consists of a small fraction of the available data and almost none of four field replicates of the same hybrid at the same site are all <LLOQ (as shown in the above plot). It is therefore expected that their effects on hybrid and site are limited due to the mean of four replicates. Furthermore, In the mean plots of Fig. 3 and the correlation in Fig. 4, those low responses are normally on the lower end which are not expected to determine the general trend (Fig. 4) and the comparisons of the variance components (Fig. 2 and Fig. 3).

- temper the interpretation/claims in places, e.g. related to the GA1 and GA4 concentrations. The last sentence of the abstract also feels a bit overreaching in relation to the results mentioned before it in the abstract

**Response:** Here’s a revised version of the sentence that tempers the interpretation while still conveying the essential findings: "Our findings provide preliminary insights into the interplay between genetics, environment, and hormonal regulation in maize, highlighting potential targets for breeding programs that could enhance agronomic traits, including yield." This revision softens the claims regarding the implications of GA1 and GA4 concentrations and avoids overreaching conclusions in relation to the results discussed earlier in the abstract.

Reviewer #2: The paper deals with large analysis of the two most bioactive GAs GA1 and GA4 in 6 different tissues of maize plants of different hybrid varieties and grown on a number of contrasting field sites.
The analysis is performed mainly by performing a correlation analysis, of which the findings appear to be that GA1 and 4 broadly covary, that there are large differences in GA levels in different tissues and that the environment has a stronger effect than Genotype, although both are significant.

I’m not enough of an expert to evaluate to what extent the analytical method is state of the art and what the quality of the measurements is.
Other than that the manuscript fails to convince.
In the introduction literature is reviewed about previous analyses of GA, but non of the work on spatial distributions of GA in growing organs or the impact of environmental factors on GA levels.

**Response:** We thank the reviewer for this helpful comment. We agree that prior studies on within-organ spatial distributions (gradients) of gibberellins and on specific environmental drivers are important for interpreting GA biology. Our study does not attempt to resolve sub-tissue spatial gradients or to isolate individual environmental variables; rather, we quantify the two most bioactive gibberellins (GA1 and GA4) at the tissue level across maize tissues, hybrids, and contrasting field sites (with “site” serving as an overall proxy for environmental effects). To provide appropriate context on the importance of GA gradients in development, we cite Ref. 7 (Rizza and Jones, 2019) and additional 4 references in the Introduction. We also note higher-resolution spatial mapping and targeted environment-factor experiments as important directions for future consideration.

Reference 4

Ubeda-Tomás S, Federici F, Casimiro I, Beemster GTS, Bhalerao R, Swarup R, et al. Gibberellin Signaling in the Endodermis Controls Root Meristem Size. Current Biology. 2009;19(14):1194-9.

Reference 8

Nelissen H, Rymen B, Jikumaru Y, Demuynck K, Van Lijsebettens M, Kamiya Y, et al. A local maximum in gibberellin levels regulates maize leaf growth by spatial control of cell division. Current Biology. 2012;22(13):1183-7.

Reference 14

[Rizza](https://www.nature.com/articles/s41477-017-0021-9) A, [Walia](https://www.nature.com/articles/s41477-017-0021-9) A, [Lanquar](https://www.nature.com/articles/s41477-017-0021-9) V, [Frommer](https://www.nature.com/articles/s41477-017-0021-9) WB, [Alexander M.Jones.](https://www.nature.com/articles/s41477-017-0021-9) In vivo gibberellin gradients visualized in rapidly elongating tissues, [Nature Plants](https://www.nature.com/nplants) 2017, 3: 803–813

Reference 15

Binenbaum J, Weinstain R, Shani E. Gibberellin Localization and Transport in Plants, Trends in Plant Science, 2018, 23: 410-421

A lot of space is taken to explain the technical details of the method, even the whole first section of the results is devoted to that. Nevertheless it is not clear what exactly was sampled. Which (parts of) leaves, roots, silks etc did they harvest, what was the developmental stage of the plant, harvest time of day, etc. All of these are expected have a significant effect on the measurements.

**Response:** We have added details to the “Maize field trials” section in Table S1.

Each site was planted in a randomized complete block design with four replicated blocks. Hybrids listed in Table 1 were included in each of the four replicated blocks where they were planted, in addition to other hybrids not associated with this study. Within a block, each hybrid was planted in an approximately 6 m × 6 m plot. Each site received a unique randomization scheme. Blocks at each site were arranged to limit variability in environmental conditions such as soil type, slope, and previous cropping history. Normal agronomic practices were followed for each site. Trial maintenance (fertilization, pesticide use, tillage practices, irrigation, etc.) varied across sites, but all maintenance activities were applied uniformly within a site. Maize samples were collected throughout the growing season and included the following tissues: leaf, root, stalk, pollen, forage, and grain. Tissues collected from each plot were pooled into a single sample. Sampling details and growth stage timing can be found in Table S1.

The results basically just deal with the observed variations, but we learn nothing about the biological meaning of these observations, so in the end there seems very little new insight.
In the discussion the authors suggest that we have learned a lot on the regulation of GA regulation, but I fail to see how they assessed regulatory mechanisms or effects.
Curiously they state to be surprised to see that there is a significant covariation between GA1 and GA4, given the (according to them) distinct synthetic pathways. This ignores the topology of the biosynthetic pathway: many of the enzymes are involved with the synthesis of both bioactive GAs. The upstream pathway up to GA12 is shared and only GA13ox transforms GA12 a precursor of GA4 to GA97 a precursor of GA1. All enzymes working GA12 and GA97 are identical creating two paralel branches regulated by the same enzymes.
In this light it is quite logical there is a covariation.

**Response:** We thank the reviewer for their insightful comment regarding the covariation of GA1 and GA4. The reviewer is correct that the biosynthetic pathways for GA1 and GA4 share a common upstream pathway and that the subsequent parallel branches utilize the same families of enzymes (e.g., GA20ox, GA3ox). From this perspective, a degree of co-regulation is indeed a logical expectation.

Our intention was not to express surprise at the fundamental biochemistry, but rather to highlight the novelty of observing this relationship so robustly in maize across diverse genetic and environmental backgrounds in the field. The maize genome contains multiple paralogs for these key enzymes, and it is not currently known whether these different enzyme isoforms exhibit preferences for substrates in the C13-hydroxylated (leading to GA1) versus the non-C13-hydroxylated (leading to GA4) pathways, or if their expression is differentially regulated by tissue type or environmental stimuli.

Given this potential for divergent regulation, observing a consistent positive correlation is a non-trivial finding. It provides the first empirical, field-level evidence that the biosynthesis of these two major bioactive GAs is, in practice, highly coordinated in maize.

To address the reviewer's concern and improve clarity, we have revised the manuscript to reframe this discussion. We have modified the text to explicitly acknowledge the shared enzymatic steps as the likely basis for the correlation, while also discussing the complexity introduced by multiple enzyme isoforms and how our data helps to resolve the question of their integrated output in an agricultural context.

So altogether this analysis and the manuscript as a whole to my mind is not yet suitable for publication.

**Response:** Below, we outlined our responses to each of the points raised:

1. **Analysis and Findings**: We have enhanced the discussion in the manuscript to clarify the biological significance of these findings and how they contribute to our understanding of gibberellin regulation in maize.
2. **Literature Review**: We have added discussion on spatial distributions of GA in growing organs and the impact of environmental factors. We have revised the introduction to incorporate relevant literature that addresses these aspects, thereby providing a more comprehensive context for our study.
3. **Technical Details and Sampling**: We have included detailed information about which parts of the maize plants were harvested (e.g., specific leaf sections, roots, silks), the developmental stages of the plants at the time of sampling, and the time of day when samples were collected in table S1. In supporting information, table 1 listed sample type and collection information (sampling details, collection growth stage, and growth stage) which shows what exactly was sampled.
4. **Biological Meaning of Observations**: We have revised the results section to not only present the observed variations but also to discuss their biological implications. This helped to provide insights into the underlying mechanisms of gibberellin regulation and its relevance to maize development.
5. **Regulatory Mechanisms**: We have revised the discussion to better articulate how our findings relate to the regulation of GA levels and to clarify any misconceptions regarding the distinct synthetic pathways of GA1 and GA4. We have incorporated the reviewer’s insights about the shared biosynthetic pathways and the implications for covariation.

We have addressed the reviewer’s concerns thoroughly to strengthen our manuscript. With these revisions, we have enhanced the clarity and impact of our findings, making the manuscript more suitable for publication.

6. PLOS authors have the option to publish the peer review history of their article ([what does this mean?](https://eur03.safelinks.protection.outlook.com/?url=https%3A%2F%2Ftrack.editorialmanager.com%2FCL0%2Fhttps%3A%252F%252Fjournals.plos.org%252Fplosone%252Fs%252Feditorial-and-peer-review-process%2523loc-peer-review-history%2F1%2F010f019cc125ad6c-294c59e9-74b9-4907-865c-a741ee48af1d-000000%2FW8sZ8-zv7awh9bLpXNsdE2gLU-bFqhE9piAnYZv_KbQ%3D253&data=05%7C02%7C%7Cd3f9efc377ce4b00c8ab08de7b2ec83f%7Cfcb2b37b5da0466b9b830014b67a7c78%7C0%7C0%7C639083638484888506%7CUnknown%7CTWFpbGZsb3d8eyJFbXB0eU1hcGkiOnRydWUsIlYiOiIwLjAuMDAwMCIsIlAiOiJXaW4zMiIsIkFOIjoiTWFpbCIsIldUIjoyfQ%3D%3D%7C0%7C%7C%7C&sdata=UqLKKz8K5zO8a7EbjdFcpM2snk8lwr5WxWYRCI40Mtk%3D&reserved=0)). If published, this will include your full peer review and any attached files.
If you choose “no”, your identity will remain anonymous but your review may still be made public.
**Do you want your identity to be public for this peer review?** For information about this choice, including consent withdrawal, please see our [Privacy Policy](https://eur03.safelinks.protection.outlook.com/?url=https%3A%2F%2Ftrack.editorialmanager.com%2FCL0%2Fhttps%3A%252F%252Fwww.plos.org%252Fprivacy-policy%2F1%2F010f019cc125ad6c-294c59e9-74b9-4907-865c-a741ee48af1d-000000%2F0-HlygqcPwvb6ZOY-xrM1b_rNDfbLlJWBynToO6uFBk%3D253&data=05%7C02%7C%7Cd3f9efc377ce4b00c8ab08de7b2ec83f%7Cfcb2b37b5da0466b9b830014b67a7c78%7C0%7C0%7C639083638484905288%7CUnknown%7CTWFpbGZsb3d8eyJFbXB0eU1hcGkiOnRydWUsIlYiOiIwLjAuMDAwMCIsIlAiOiJXaW4zMiIsIkFOIjoiTWFpbCIsIldUIjoyfQ%3D%3D%7C0%7C%7C%7C&sdata=PKWGDYmZ%2Bx66sxnulQvLnPRY0CjM0zrNRBq51LXtORk%3D&reserved=0).

Reviewer #1: No

Reviewer #2: **Yes:** Gerrit Beemster

[NOTE: If reviewer comments were submitted as an attachment file, they will be attached to this email and accessible via the submission site. Please log into your account, locate the manuscript record, and check for the action link "View Attachments". If this link does not appear, there are no attachment files.]

To ensure your figures meet our technical requirements, please review our figure guidelines: <https://journals.plos.org/plosone/s/figures>

You may also use PLOS’s free figure tool, NAAS, to help you prepare publication quality figures: <https://journals.plos.org/plosone/s/figures#loc-tools-for-figure-preparation>.

NAAS will assess whether your figures meet our technical requirements by comparing each figure against our figure specifications.

*In compliance with data protection regulations, you may request that we remove your personal registration details at any time.* [*(Remove my information/details)*](https://eur03.safelinks.protection.outlook.com/?url=https%3A%2F%2Ftrack.editorialmanager.com%2FCL0%2Fhttps%3A%252F%252Fwww.editorialmanager.com%252Fpone%252Flogin.asp%253Fa%3Dr%2F1%2F010f019cc125ad6c-294c59e9-74b9-4907-865c-a741ee48af1d-000000%2FI4Wg49dN3N3tvWGHK3SB18I5Zs2BAvr_Jey1kNYOpAc%3D253&data=05%7C02%7C%7Cd3f9efc377ce4b00c8ab08de7b2ec83f%7Cfcb2b37b5da0466b9b830014b67a7c78%7C0%7C0%7C639083638484922295%7CUnknown%7CTWFpbGZsb3d8eyJFbXB0eU1hcGkiOnRydWUsIlYiOiIwLjAuMDAwMCIsIlAiOiJXaW4zMiIsIkFOIjoiTWFpbCIsIldUIjoyfQ%3D%3D%7C0%7C%7C%7C&sdata=rvqUTO9Wp939pWkCJX00JPccEfic8e4H%2Bh9WLDlzXOY%3D&reserved=0)*. Please contact the publication office if you have any questions.*
