# Supplementary material for: Natural variation of bioactive gibberellin levels in maize tissues across North America
Source: PLoS One. 2026 Oct 7;21(10):e0340156. doi: 10.1371/journal.pone.0340156 (PMC13645078; doi:10.1371/journal.pone.0340156)
Supplement: Supplementary file 6 [file pone.0340156.s009.docx]

Response to Reviewers

PONE-D-25-66749R2

Natural variation of bioactive gibberellin levels in maize tissues across North America

PLOS One

Dear Dr. Wang,

Thank you for submitting your manuscript to PLOS ONE. After careful consideration, we feel that it has merit but does not fully meet PLOS ONE’s publication criteria as it currently stands. Therefore, we invite you to submit a revised version of the manuscript that addresses the points raised during the review process.

***Response***

***Dear Editor,***

***Thank you very much for your letter and for the careful evaluation of our manuscript. We sincerely appreciate your comments and the reviewers’ constructive suggestions, which have been very helpful in improving the quality and clarity of our work.***

***We have carefully addressed the comments from both the editor and the reviewers on a point-by-point basis. In response to these comments, we have revised the manuscript accordingly and made the necessary changes throughout the text.***

***In our revised submission, we have included the following files as requested:***

***1. Response to Reviewers – a separate letter responding to each point raised by the academic editor and reviewers.***

***2. Revised Manuscript with Track Changes – a marked-up version highlighting all changes made to the manuscript PONE-D-25-66749R1.***

***3. Manuscript – a clean, unmarked version of the revised paper without tracked changes.***

***Thank you again for your time and consideration. We are grateful for the opportunity to revise our manuscript for further consideration by PLOS ONE.***

You have improved the paper notably. Nevertheless problems remain. Reviewer 1, a statistician, points out fairly serious inadequacies remain in your presentation. Insofar as statistical analysis constitutes a big chunk of your paper, I believe that you need to get this right. Hopefully, the problems identified by the reviewer are matters of language (what you write about the methods). But in any case, I hope you can respond satisfactorily.

***Response: Thank you for highlighting this important concern. We appreciate the editor’s emphasis on the need for a clear and rigorous presentation of the statistical analysis, particularly given its central role in the manuscript. In response, we carefully re-examined the statistical methods and substantially revised the “Variance component analysis to compare genetic and environmental variations” section to improve clarity, precision, transparency, and consistency.***

***We thoroughly revised the methodology section to clarify our statistical approaches, with emphasis on model assumptions, structure, and limitations. We explicitly defined our variance-partitioning framework, detailing how hybrid, site, replicate within site, and the hybrid-by-site interaction were treated across different stages of the analysis, specifically for estimating variance components, testing their significance, and examining changes in hybrid means across sites. To address the reviewer's concerns, we clarified that our use of REML (Restricted Maximum Likelihood) estimation is a well-established approach for variance component estimation in unbalanced trial designs. We also explained our rationale for not applying multiple-testing adjustments during significance testing of these components, and why confidence intervals for variance components were not provided. In addition, we removed references to SAS PROC GLM, which had been used only for intermediate data exploration, so that the manuscript now reflects only the analyses underlying the reported results. Overall, these revisions present the statistical methodology with greater transparency, stronger theoretical justification, and more appropriate caution regarding the scope and unbalanced nature of the available data.***

Reviewer three (the original reviewer 2 was not available) points out several small but useful things. Their main point tho is about interpretation. Writing for PLoS One differs from many other journals because "importance" does not matter. For most journals, we write to convince the editor, the reviewers, and indeed readers, that our work is important. But importance is subjective. PLoS One removes that criteron, explicitly. Instead, what matters is validity. Writing on that head relieves us of the burden of hype. We present what we found, a straightforward account. We can of course state why we think the work matters but in doing so we should be brief and circumspect. Reviewer 3 points out where you need to town down your claims. I would add that you can probably shorten the discussion; compared to the results, it seems quite long.

***Response: Thank you for the great comments. The revised Discussion now focuses more directly on the validity of the findings, the limitations of the study design and measurements, and a more circumspect interpretation of the implications.***

***We revised the Discussion to align more closely with the data presented in the Results. Specifically, we removed or rephrased language that could be interpreted as overly broad or mechanistic, including statements related to “biosynthesis” and “plasticity of the gibberellin pathway,”. We also shortened the Discussion by removing repetitive statements and reducing broader contextual language.***

Small points: When you cite references in the text, please put a space between the word and the bracket. Like this [14]. Not like this[14].  Also on line 74, the word 'corn' should not be capitalized.

***Response: Thank you for pointing these out. We have made these corrections throughout the manuscript, including adding a space before citation brackets where needed and changing “Corn” to “corn” on previous line 74.***

Please submit your revised manuscript by Aug 05 2026 11:59PM. If you will need more time than this to complete your revisions, please reply to this message or contact the journal office at [plosone@plos.org](mailto:plosone@plos.org). When you're ready to submit your revision, log on to <https://www.editorialmanager.com/pone/> and select the 'Submissions Needing Revision' folder to locate your manuscript file.

Please include the following items when submitting your revised manuscript:

- A letter that responds to each point raised by the academic editor and reviewer(s). You should upload this letter as a separate file labeled 'Response to Reviewers'.
- A marked-up copy of your manuscript that highlights changes made to the original version. You should upload this as a separate file labeled 'Revised Manuscript with Track Changes'.
- An unmarked version of your revised paper without tracked changes. You should upload this as a separate file labeled 'Manuscript'.

If you would like to make changes to your financial disclosure, please include your updated statement in your cover letter. Guidelines for resubmitting your figure files are available below the reviewer comments at the end of this letter.

If applicable, we recommend that you deposit your laboratory protocols in protocols.io to enhance the reproducibility of your results. Protocols.io assigns your protocol its own identifier (DOI) so that it can be cited independently in the future. For instructions see: [https://journals.plos.org/plosone/s/submission-guidelines#loc-laboratory-protocols](https://eur03.safelinks.protection.outlook.com/?url=https%3A%2F%2Ftrack.editorialmanager.com%2FCL0%2Fhttps%3A%252F%252Fjournals.plos.org%252Fplosone%252Fs%252Fsubmission-guidelines%2523loc-laboratory-protocols%2F1%2F010f019eed212bcb-2e923432-4d7a-4dfe-b499-b927df7c47c4-000000%2Fabc4g1wACBx0u-wBgM085qLC0jonyJJpiCvFepbNaTc%3D258&data=05%7C02%7C%7Ce9ccd69d9bf244dd6bf208ded004d848%7Cfcb2b37b5da0466b9b830014b67a7c78%7C0%7C0%7C639176916391789051%7CUnknown%7CTWFpbGZsb3d8eyJFbXB0eU1hcGkiOnRydWUsIlYiOiIwLjAuMDAwMCIsIlAiOiJXaW4zMiIsIkFOIjoiTWFpbCIsIldUIjoyfQ%3D%3D%7C0%7C%7C%7C&sdata=CQpqlwWoXxKH610RGSsMpZtRzKZWq74b%2FAecShjT1LU%3D&reserved=0). Additionally, PLOS ONE offers an option for publishing peer-reviewed Lab Protocol articles, which describe protocols hosted on protocols.io. Read more information on sharing protocols at [https://plos.org/protocols?utm_medium=editorial-email&utm_source=authorletters&utm_campaign=protocols](https://eur03.safelinks.protection.outlook.com/?url=https%3A%2F%2Ftrack.editorialmanager.com%2FCL0%2Fhttps%3A%252F%252Fplos.org%252Fprotocols%253Futm_medium%3Deditorial-email%2526utm_source%3Dauthorletters%2526utm_campaign%3Dprotocols%2F1%2F010f019eed212bcb-2e923432-4d7a-4dfe-b499-b927df7c47c4-000000%2Ffele3_dOcQHdgQq0jaKZxlu2rIw74v9-9llnnVUMzdw%3D258&data=05%7C02%7C%7Ce9ccd69d9bf244dd6bf208ded004d848%7Cfcb2b37b5da0466b9b830014b67a7c78%7C0%7C0%7C639176916391822508%7CUnknown%7CTWFpbGZsb3d8eyJFbXB0eU1hcGkiOnRydWUsIlYiOiIwLjAuMDAwMCIsIlAiOiJXaW4zMiIsIkFOIjoiTWFpbCIsIldUIjoyfQ%3D%3D%7C0%7C%7C%7C&sdata=fj8n6ZMSkMhYTL7f1YJbV0jv193pg%2F%2BwaO3ZMreKVKg%3D&reserved=0).

As the corresponding author, your ORCID iD is verified in the submission system and will appear in the published article. PLOS supports the use of ORCID, and we encourage all coauthors to register for an ORCID iD and use it as well. Please encourage your coauthors to verify their ORCID iD within the submission system before final acceptance, as unverified ORCID iDs will not appear in the published article. *Only* the individual author can complete the verification step; PLOS staff *cannot* verify ORCID iDs on behalf of authors.

We look forward to receiving your revised manuscript.

Kind regards,

Tobias Isaac Baskin
Academic Editor
PLOS One

Journal Requirements:

If the reviewer comments include a recommendation to cite specific previously published works, please review and evaluate these publications to determine whether they are relevant and should be cited. There is no requirement to cite these works unless the editor has indicated otherwise. 

[Note: HTML markup is below. Please do not edit.]

Reviewers' comments:
Reviewer's Responses to Questions

**Comments to the Author**
1. If the authors have adequately addressed your comments raised in a previous round of review and you feel that this manuscript is now acceptable for publication, you may indicate that here to bypass the “Comments to the Author” section, enter your conflict of interest statement in the “Confidential to Editor” section, and submit your "Accept" recommendation.

Reviewer #1: (No Response)

Reviewer #3: (No Response)

2. Is the manuscript technically sound, and do the data support the conclusions?

The manuscript must describe a technically sound piece of scientific research with data that supports the conclusions. Experiments must have been conducted rigorously, with appropriate controls, replication, and sample sizes. The conclusions must be drawn appropriately based on the data presented.

Reviewer #1: Partly

Reviewer #3: Partly

3. Has the statistical analysis been performed appropriately and rigorously?

Reviewer #1: No

Reviewer #3: Yes

4. Have the authors made all data underlying the findings in their manuscript fully available?

The [PLOS Data policy](https://eur03.safelinks.protection.outlook.com/?url=http%3A%2F%2Ftrack.editorialmanager.com%2FCL0%2Fhttp%3A%252F%252Fwww.plosone.org%252Fstatic%252Fpolicies.action%2523sharing%2F1%2F010f019eed212bcb-2e923432-4d7a-4dfe-b499-b927df7c47c4-000000%2FMKeghzzDYBUz4O8zqdrk9z-3BU_aprvoa_pLbKXaSr8%3D258&data=05%7C02%7C%7Ce9ccd69d9bf244dd6bf208ded004d848%7Cfcb2b37b5da0466b9b830014b67a7c78%7C0%7C0%7C639176916391842144%7CUnknown%7CTWFpbGZsb3d8eyJFbXB0eU1hcGkiOnRydWUsIlYiOiIwLjAuMDAwMCIsIlAiOiJXaW4zMiIsIkFOIjoiTWFpbCIsIldUIjoyfQ%3D%3D%7C0%7C%7C%7C&sdata=uNGwOurDU4BRVWKHO3C3G4EabrcIsjIYIs5%2FnDTE1yY%3D&reserved=0) requires authors to make all data underlying the findings described in their manuscript fully available without restriction, with rare exception (please refer to the Data Availability Statement in the manuscript PDF file). The data should be provided as part of the manuscript or its supporting information, or deposited to a public repository. For example, in addition to summary statistics, the data points behind means, medians and variance measures should be available. If there are restrictions on publicly sharing data—e.g. participant privacy or use of data from a third party—those must be specified.

Reviewer #1: Yes

Reviewer #3: Yes

5. Is the manuscript presented in an intelligible fashion and written in standard English?

PLOS ONE does not copyedit accepted manuscripts, so the language in submitted articles must be clear, correct, and unambiguous. Any typographical or grammatical errors should be corrected at revision, so please note any specific errors here.

Reviewer #1: Yes

Reviewer #3: Yes

6. Review Comments to the Author

Please use the space provided to explain your answers to the questions above. You may also include additional comments for the author, including concerns about dual publication, research ethics, or publication ethics. (Please upload your review as an attachment if it exceeds 20,000 characters)

**Reviewer #1**: Experiments (blocking structure, randomisation, etc) are now much more clearly explained in the Methods. Though l130-132 "Maize samples were collected throughout the growing season and included the following tissues: leaf, root, stalk, pollen, forage, and grain. The same tissues collected from each plot were pooled into a single sample." remains somewhat ambiguous. How much of each tissue (and was this by weight, or volume, or something else)? From how many plants, and how were they selected (random, non-edge, convenience)? How pooled? Was pooling consistent across all sites?

***Response: Thank you for the comments. We have revised the Methods section to clarify sufficient details regarding the amount of tissue collected, the number of plants sampled, the selection criteria, and the pooling procedure. For each plot, sampled plants were selected non-systematically but with consistent field-sampling criteria: plot ends were avoided, and plants were chosen to be competitive and representative of the plot as a whole. The selected tissue samples from each plot were combined to generate one pooled sample per plot, which served as the observational unit for downstream analysis. This pooling approach was applied consistently across all sites.***

***We have added detailed sampling information in S1 Table, including the pooled sample amount per plot, the number of plants sampled per plot, tissue-specific sampling details, and the collection growth stage. Specifically, the revised table now specifies the tissue-specific sampling amount used to generate one pooled sample per lot: 60 leaf sections, 8 roots, 4 stalks, 10 mL pollen, 1 forage plant, or 1 kg grain, depending on tissue type, along with the corresponding number of sampled plants and growth stages.***

***S1 Table. Summary of sample type and collection information***

| ***Sample Type*** | ***Pooled Sample Amount per Plot*** | ***Number of Plants Sampled per Plot*** | ***Sampling Details*** | ***Collection Growth Stage*** | ***Growth Stage Description*** |
| --- | --- | --- | --- | --- | --- |
| ***Leaf*** | ***60 leaves*** | ***60*** | ***Approximately 15 cm of most recently collared leaf*** | ***V2-V4*** | ***A plant with 2 to 4 leaf collars*** |
| ***Root*** | ***8 roots*** | ***8*** | ***All below-ground root tissue, thoroughly washed*** | ***V2-V4*** | ***A plant with 2 to 4 leaf collars*** |
| ***Stalk*** | ***4 stalks*** | ***4*** | ***All above-ground stalk tissue with leaf blade, leaf sheath, ear, and tassel removed*** | ***V10-V12*** | ***A plant with 10 to 12 leaf collars*** |
| ***Pollen*** | ***10 mL*** | ***Highly variable based on environmental conditions, though typically >10*** | ***Fresh pollen with all anthers removed*** | ***Pollen shed*** | ***A plant that is actively shedding pollen*** |
| ***Forage*** | ***1 plant*** | ***1*** | ***All above-ground plant tissue*** | ***R5, early dent*** | ***Most kernels are dented and contain approximately 55% moisture*** |
| ***Grain*** | ***1 kg*** | ***Minimum of 4*** | ***Shelled grain dried to ≤15.5% moisture prior to shipping and storage*** | ***R6*** | ***Physiological maturity; black layer visible at base of kernels*** |

Whilst the Methods section explains the experiment better, there's been no attempt made to connect it to the subsequent model, eqn (1). Does "block" (in intro) = "replicate" (in the model)? Does "plot" (in the intro) = the observational unit (in the model)? Does "pooling" (in intro) mean there is one observation per plot? The disconnect is a bit jarring, but could easily be fixed.

***Response: Thank you for the suggestion. In the revised manuscript, we clarified that “block” in the experimental design corresponds to “replicate” in the statistical model, and that “plot” is the observational unit used in the model. We also clarified that the tissue samples collected within each plot were pooled to generate one composite sample per plot; therefore, there was one analytical observation per plot for each tissue and GA measurement. These clarifications have been added to the revised Methods section to better connect the field design, sample pooling, and statistical model.***

The issue of unbalancedness is still not well acknowledged or addressed. I appreciate that this is (now) historical data and it is out of scope to remedy the imbalance by collecting more data. Nevertheless, it is important still that the statistical inference is accurate and doesn't overstate what the data imply. l202-230 unfortunately still feel muddled with respect to the assumed statistical model. l204-206 refer to "fitting the model", but does so before fully specifying the model: which coefficients are random, and with what variance structure? The sentence "Differences may present under an unbalanced design as in this investigation, and PROC GLIMMIX is the best for significant testing due to the likelihood-based method (which is the ultimate standard for all statistical analyses), PROC MIXED supposes to be unbiased for the estimate of variance component because of the restricted maximum likelihood and the application of mixed model equations for random effects, and PROC GLM provided mean predictions of random effects by the method of moment (i.e. the expected mean square or EMS)" is very unclear to me. It does not describe the statistical assumptions or methods directly, but rather refers to SAS procedures and seems to expect the reader to reverse-engineer the implied model from that. “PROC GLM provided mean predictions of random effects by the method of moment” is especially confusing: my understanding is that PROC GLM applies to fixed effects rather than random effects, so what is meant by this statement is at best unclear. This whole section remains muddled, suggests over-reliance/over-confidence in software, and still underplays the problems caused by the unbalanced design. Likelihood-based methods are not automatically reliable in small or highly unbalanced settings, and similarly REML “unbiasedness” is not guaranteed in that same setting. It should not be for the reader or reviewer to have to unpick this.

***Response: The manuscript was revised to clarify the methodological approaches. Specifically, to estimate variance components, hybrid, site, replicate within site, and the hybrid-by-site interaction were all treated as random effects, with the residual term representing error variance. Random effects were assumed to be independently and identically distributed within each classification, each associated with its own variance component. Variance was partitioned into these additive components using SAS PROC MIXED with REML (Restricted Maximum Likelihood) estimation, a well-established approach for variance component estimation in unbalanced trial designs.***

***The significance of variance components was then evaluated using PROC GLIMMIX with the COVTEST option via likelihood ratio tests. Because the null hypothesis places a variance component on the boundary of the parameter space at zero, the resulting test statistic follows a mixture chi-square distribution. Significance was evaluated at the 5%, 1%, and 0.1% levels without multiplicity correction.***

***For variance components that were found to be significant, additional analyses were performed to examine mean changes for individual hybrids across sites, thereby illustrating the extent to which hybrid effects were connected across sites and characterizing site-specific hybrid patterns.***

***These analyses were conducted within a linear mixed model framework using PROC MIXED. hybrid, site, and the hybrid-by-site interaction were treated as fixed effects, whereas Replicate nested within Site was modeled as a random effect. Treating hybrid and site as fixed effects produced local LS-means for characterizing hybrid-by-site patterns, while modeling nested replications as random prevented estimates from being biased by local block-level anomalies.***

***In addition, we removed references to SAS PROC GLM, which had been used only for intermediate data exploration, so that the manuscript now reflects only the analyses underlying the reported results.***

***Consequently, the approach provides valid and reliable inference for unbalanced data, though statistical power may be reduced relative to a fully balanced design.***

On multiple testing, I also found the authors’ response confusing (“The multiple testing was not considered because the relative comparison (e.g. hybrid versus site)…”), and it would help to be more explicit. In Table 5, are you not reporting significance tests for variance components (at least hybrid, site, and hybrid × site, I think) separately for 10 GA–tissue combinations, i.e. at least 30 formal tests? Then later there are five separate GA1–GA4 regression/correlation tests, one for each tissue. At present these nominal p-values appear to be interpreted directly, and merely noting significance at multiple levels (5%, 1%, and 0.1%) does not really address the multiplicity issue. I am not suggesting that a formal multiple-testing correction is needed in what is partly an exploratory study, but the manuscript should at least acknowledge the number of related tests being carried out and temper the interpretation of marginal findings accordingly.

***Response: The revisions were made in the manuscript to address this concern. The primary objective of this analysis was to describe how variation in GA levels was partitioned among model components, rather than to perform confirmatory hypothesis testing. For each GA-by-tissue combination, variance components associated with hybrid, site, replicate nested within site, and the hybrid-by-site interaction were evaluated using individual likelihood ratio tests, resulting in 40 variance-component tests in total. No multiplicity adjustment was applied because these tests were used for an exploratory assessment of variance structure. Accordingly, the resulting p-values should be interpreted descriptively, together with the estimated variance components and their relative contributions to total variation. Although conducting 40 tests increases the chance of false positives, effects supported by both substantial estimated variance contributions and very small p-values provide stronger evidence of meaningful structure in the data than marginally significant effects.***

On confidence intervals, I do not understand why the authors regard the log scale as an obstacle (since variance components are naturally on that scale anyway). Confidence intervals are precisely what convey uncertainty in weakly identified settings, such as stemming from unbalanced design.

***Response: Variance components were estimated by fitting an all-random model that included hybrid, site, replicate nested within site, the hybrid-by-site interaction, and residual error. This model was specified to decompose total response variation and quantify the relative contributions of environmental, design-related, and interaction sources across sites. Because the primary objective was to identify the main drivers of variability, results are presented as variance-component point estimates and their relative percentage contributions. These measures provide the most direct and practical basis for evaluating the importance of each source of variation.***

**Reviewer #3**: This manuscript reports a validated high-throughput LC-MS/MS workflow and uses it to quantify the bioactive gibberellins GA1 and GA4 in five maize tissues from ten commercial hybrids grown at six U.S. field sites, partitioning the variation into genetic (hybrid), environmental (site), and interaction components. The analytical method appears sound and the geographic breadth is a genuine strength, so the descriptive dataset has value as a baseline resource.

I have read the previous review round. The authors have made constructive changes — the Results and Discussion are now separated, the experimental design and sampling are described in more detail (Table S1), the GA1–GA4 correlation discussion has been appropriately tempered to acknowledge its correlational nature, and the closing claims have been softened to "preliminary insights." I appreciate these revisions.

That said, my main remaining concern is that the conclusions still reach beyond what the measured data can support, and this stems in large part from what was measured. Only the two bioactive end-products (GA1, GA4) were quantified. A steady-state pool reflects the balance of biosynthesis, 2β-hydroxylation-mediated inactivation, and conjugation, so the present data cannot attribute any observed change to a specific step. This is particularly problematic for the central claim that site (i.e., environment) is the dominant factor: the manuscript cannot say whether a site-associated change in GA1/GA4 arises from altered biosynthetic flux or from altered inactivation.

Maize is an early-13-hydroxylation (GA1-type) species, in which GA1 is inactivated to GA8 and GA4 to GA34 by GA2ox. At minimum, the authors must (i) state this limitation explicitly and (ii) remove or rephrase "biosynthesis" and "plasticity of the gibberellin pathway" language in favour of "endogenous level/abundance" wherever flux was not measured. To support any mechanistic interpretation of the site effect, quantification of the principal inactivated GAs (GA8, GA34) and key precursors (e.g., GA20, GA9), together with active-to-inactive ratios (GA1/GA8, GA4/GA34), would help indicate whether a given change reflects enhanced synthesis or reduced catabolism — although, as these are themselves steady-state pools, such ratios remain indirect proxies for the underlying flux.

***Response: Thanks for the comments. We revised the Abstract, Introduction, Discussion, and Conclusion by deleting the language such as “biosynthesis,” “pathway plasticity,” and “regulation”. Biosynthetic pathway was not discussed in the paper.***

Methods (L167–181). Present the LC gradient as a table including the initial hold, and confirm the steep short segments (e.g., B 26%→55% over 0.1 min) are not transcription errors. Critically, retention times for GA1, GA4, and the internal standards are not reported; please add them (and a representative MRM chromatogram near the LLOQ), as they are needed to judge whether this unusual gradient performs as intended.

***Response: Thank you for this helpful suggestion. We have revised the Methods section to present the LC gradient as Table 2, including the initial hold. We confirm that the steep, short gradient segments, such as the change from 26% to 55% B over 0.1 min, are intentional and are not transcription errors.***

***We have also added the retention times for GA1, GA4, and the corresponding internal standards in Table 3. In addition, we provided a representative MRM chromatogram at the LLOQ (standard 1), as Supporting Information S1 Fig. This added information should allow readers to better assess the chromatographic performance of the method and confirm that the gradient performs as intended.***

***S1 Fig. The representative chromatogram of LLOQ (standard 1)***

***Top left: TIC, Top right: XIC of GA1, Bottom left: XIC of GA1-D4 and GA4-D2, Bottom right: XIC of GA4.***

Table 3 (pollen). The text states that pollen data were not reported because of its short stability window (L262–263), and pollen is correctly absent from Tables 4–5 and Figs 2–4. However, Table 3 still lists method-validation data for pollen.

***Response: Thank you for raising this point. The pollen results included in Table 3 refer only to the method-validation experiment. We validated the method for pollen along with the other maize tissues, including assessments of accuracy, precision, extraction efficiency, recovery, and benchtop stability; therefore, the validated pollen data are appropriately included in Table 3.***

***However, pollen had a much shorter benchtop stability window than the other tissues. Although this was manageable during method validation because only a small number of samples were processed, it was not feasible for the large field-sample set. The field study included more samples, and pollen sample preparation could not be completed within the validated stability window. For this reason, we did not consider the field pollen measurements sufficiently reliable for downstream biological interpretation and therefore excluded pollen from Tables 4–5 and Figures 2–4 for field data.***

Minor comments

Introduction (L49). "Gibberella fujikuroi" → the accepted name is Fusarium fujikuroi (teleomorph Gibberella fujikuroi).

***Response: “Gibberella fujikuroi" was changed to “Fusarium fujikuroi”.***

Methods (L157). "until about 400 mL of aqueous phase was left" — should be 400 µL.

***Response: 400 “mL” was corrected to 400 “µL”.***

Methods (L152). "Nutating Mixter" → Mixer; and a missing space after "88-861-043)" before "After incubation".

***Response: "Nutating Mixter" was changed to “Mixer”, and a space was added before "After incubation".***

Methods (L168). "AB Sxiex OS" → "AB Sciex OS"

***Response: "AB Sxiex OS" was corrected to "AB Sciex OS".***

7. PLOS authors have the option to publish the peer review history of their article ([what does this mean?](https://eur03.safelinks.protection.outlook.com/?url=https%3A%2F%2Ftrack.editorialmanager.com%2FCL0%2Fhttps%3A%252F%252Fjournals.plos.org%252Fplosone%252Fs%252Feditorial-and-peer-review-process%2523loc-peer-review-history%2F1%2F010f019eed212bcb-2e923432-4d7a-4dfe-b499-b927df7c47c4-000000%2FQojGTO6dVByeM2UcTzsbU-GYRotikTY9X5b8mF1-nDc%3D258&data=05%7C02%7C%7Ce9ccd69d9bf244dd6bf208ded004d848%7Cfcb2b37b5da0466b9b830014b67a7c78%7C0%7C0%7C639176916391859933%7CUnknown%7CTWFpbGZsb3d8eyJFbXB0eU1hcGkiOnRydWUsIlYiOiIwLjAuMDAwMCIsIlAiOiJXaW4zMiIsIkFOIjoiTWFpbCIsIldUIjoyfQ%3D%3D%7C0%7C%7C%7C&sdata=RC7hYom2vOf%2BKRXpFv1ozFFg8wsw8saEzfgdlUPYzbk%3D&reserved=0)). If published, this will include your full peer review and any attached files.

If you choose “no”, your identity will remain anonymous but your review may still be made public.

**Do you want your identity to be public for this peer review?** For information about this choice, including consent withdrawal, please see our [Privacy Policy](https://eur03.safelinks.protection.outlook.com/?url=https%3A%2F%2Ftrack.editorialmanager.com%2FCL0%2Fhttps%3A%252F%252Fwww.plos.org%252Fprivacy-policy%2F1%2F010f019eed212bcb-2e923432-4d7a-4dfe-b499-b927df7c47c4-000000%2Fl_Er0sLTtBKMVED9sMoDIKGfzjj7iRUPA5pk924gHu0%3D258&data=05%7C02%7C%7Ce9ccd69d9bf244dd6bf208ded004d848%7Cfcb2b37b5da0466b9b830014b67a7c78%7C0%7C0%7C639176916391880187%7CUnknown%7CTWFpbGZsb3d8eyJFbXB0eU1hcGkiOnRydWUsIlYiOiIwLjAuMDAwMCIsIlAiOiJXaW4zMiIsIkFOIjoiTWFpbCIsIldUIjoyfQ%3D%3D%7C0%7C%7C%7C&sdata=YgB506pYVhEnne%2BIOB2iHc%2BdlXLBSoY7FV7OHlN2p1I%3D&reserved=0).

Reviewer #1: No

Reviewer #3: No

[NOTE: If reviewer comments were submitted as an attachment file, they will be attached to this email and accessible via the submission site. Please log into your account, locate the manuscript record, and check for the action link "View Attachments". If this link does not appear, there are no attachment files.]

To ensure your figures meet our technical requirements, please review our figure guidelines: <https://journals.plos.org/plosone/s/figures>

You may also use PLOS’s free figure tool, NAAS, to help you prepare publication quality figures: <https://journals.plos.org/plosone/s/figures#loc-tools-for-figure-preparation>.

NAAS will assess whether your figures meet our technical requirements by comparing each figure against our figure specifications.
