# Supplementary material for: Natural variation of bioactive gibberellin levels in maize tissues across North America
Source: PLoS One. 2026 Oct 7;21(10):e0340156. doi: 10.1371/journal.pone.0340156 (PMC13645078; doi:10.1371/journal.pone.0340156)
Supplement: Supplementary file 7 [file pone.0340156.s010.docx]

PONE-D-25-66749R2

Natural variation of bioactive gibberellin levels in maize tissues across North America

PLOS One

Dear Dr. Wang,
Thank you for submitting your manuscript to PLOS One. After careful consideration, the Journal and I feel that your manuscript is much improved but does not yet entirely meet PLOS One’s publication criteria. A few minor issues remain. Therefore, we invite you to submit a revised version of the manuscript that addresses the points raised during the review process.
First, I apologize for the delay in getting this back to you. The original reviewer 1, who is a statistician, was unavailable. I needed to find another statistitian and this took me some time, especially because of the summer holidays. Happily, this new reviewer (number 4) is mostly satisified and brings up just a few points needing clarification. Reviewer 3 is completely happy.

 I read the paper too and feel that it is in good shape. I have a few matters of language to call to your attention.

Line 25 (and elsewhere). I suggest you delete the word 'endogenous'. How could a hormone be 'exogenous'? I think in most places you can delete the word 'endogenous'.

**Response:** “endogenous” in the paper was deleted.

Line 25. The word 'phytohomorme' was coined more than 100 years ago when scientists had little idea what was going on. Now we understand that plants have hormones. Of course they differ from those of animals, but there is no reason they would be the same. Other than the fact that they occur in plants, there is nothing that unites 'phytohormones' into a group. What is more, no one ever uses the word "zoohormones" to indicate animal hormones. There is no need. LIkewise, I can see no reason to use the term phytohormones. Writing 'hormones' is clear. No one would think you are writing about animal hormones.

**Response:** We thank the editor for this helpful suggestion. We agree that “plant hormones” is more appropriate in this context. Accordingly, we have revised “phytohormone(s)” to “plant hormone(s)” throughout the manuscript where applicable.

Line 30, and elsewhere. The word tissue is defined as a group of cells of a similar type, such as epidermis or mesophyll. A root is not a tissue. Unfortunately, people often write loosely about throwing plant tissue in the blender, using the word 'tissue' as a synonym for 'material'. In that case, the casual wording probably doesn't matter. But here, the source of the sample is a big part of the story. It strikes me as misleading for you to call your samples 'tissues' when they are not. I realize that because your samples are not all organs, there is not an obvious alternative. However, I have gone thru your paper and suggested revisions. Please see the uploaded marked manuscript. I don't know that these are the best choices, you might well find alternatives. But at least my edits will give you a place to start. (You might like them).

 By the way, the markup on the uploaded ms covers a few more things than the issue of tissue. These are editing suggestions. You do not have to address the markup in your rebuttal letter that will accompany your revision.

**Response:** We thank the editor for the careful review and helpful suggested revisions. We understand the concern that the standalone use of “tissue” may be imprecise when referring to sample sources such as root, stalk, grain, or combined plant material. To improve clarity, we have reviewed the manuscript and revised the wording where appropriate. Specifically, we now use more precise terms such as “maize tissue samples,” “maize samples,” or “plant material” when referring to the specimens collected, processed, or analyzed. Where the text discusses biological differences among sample types such as leaf, root, stalk, pollen, and grain, we retain “maize tissues” because this terminology is commonly used in the plant science literature to describe different maize sample types in comparative analyses.

For example, similar usage can be found in PLOS ONE publications, including Lin et al. (2014), where maize root, stem, and leaf were described as different plant tissues, and Luís et al. (2016), where “maize-leaf tissue” was used to describe maize leaves. We have also considered the editor’s marked manuscript suggestions and incorporated revisions where they improved clarity and accuracy.

Reference 1

LinY, ZhangC, LanH, GaoS, LiuH,etal. (2014) Validation of Potential Reference Genes for qPCR in Maize across Abiotic Stresses, Hormone Treatments, and Tissue Types. PLoSONE9(5):e95445.doi:10.1371/journal.pone.0095445

Reference 2

Luís IM, Alexandre BM, Oliveira MM, Abreu IA (2016) Selection of an Appropriate Protein Extraction Method to Study the Phosphoproteome of Maize Photosynthetic Tissue. PLoS ONE 11(10): e0164387. https://doi.org/10.1371/journal.pone.0164387

Line 52. "regulate and control". Both? How do these concepts differ? Pick one. But even better, use neither. I find it odd to say that 'molecule x regulates process y.' Biologists write like this all the time but in doing so they cheapen the concept of regulation almost to the point of meaninglessness. Regulation implies feedback; and for feedback you need a system. A cell or a plant or perhaps a pathway can be said to regulate a process. Here, you can write "They are involved in ..."

**Response:** Thank you for your suggestion. In the manuscript, we changed “regulate and control” to “are involved in” and adjust the “Regulate” to “modulate” or “contribute to” when it fits.

 Line 85. Did the research in all of those papers measure active gibberellins only? That surprises me; usually both active and inactive ones are measured. Please check. By the way, why write 'bioactive'? How does that differ from 'active'? Where else would a gibberellin be active if not in an organism? I suggest removing the superfluous 'bio'.

**Response:** We thank the editor for this helpful comment. We have checked the cited papers and revised the text to ensure that the description accurately reflects the gibberellins measured in those studies. In the present study, we measured GA1 and GA4, which are commonly recognized as bioactive gibberellins in plants.

Regarding the term “bioactive,” we respectfully prefer to retain it where appropriate because it has a specific meaning in the gibberellin literature. Although more than 130 gibberellins have been identified in plants, only a small subset, including GA1, GA3, GA4, and GA7, can bind the GID1 receptor and trigger GA signaling. Other gibberellins function as biosynthetic precursors or inactive catabolites. Therefore, “bioactive gibberellins” distinguishes the signaling-competent forms from inactive or precursor forms.

We agree that “active” and “bioactive” can appear similar in general usage; however, “bioactive gibberellins” is the established terminology used in the plant hormone field to refer specifically to receptor-active, signal-transducing gibberellins. This usage is supported by prior literature, including Tang et al. (2025), Rahman et al. (2026), and Hedden and Thomas (2012). Therefore, we have retained “bioactive gibberellins” where it is scientifically specific, while reviewing the manuscript to avoid unnecessary or unclear wording elsewhere.

Reference 1

Tang, J., Zhao, G., Tian, X., Bu, Q. and Li, W. (2025), Precise modulation of bioactive gibberellin homeostasis: A promising strategy for enhancing abiotic stress tolerance. J. Integr. Plant Biol., 67: 2247-2249. <https://doi.org/10.1111/jipb.13942>

Reference 2

Shafeeq Rahman, Shabanamol Subaida Beevi, Fathimath Nishana, Khaled Masmoudi, Rajesh Muliyar Krishna, Shyam Sreedhara Kurup, Gibberellin homeostasis and signaling in plant growth, stress adaptation, and crop improvement, Current Plant Biology, Volume 49,

2026, 100654, <https://doi.org/10.1016/j.cpb.2026.100654>.

Reference 3

Peter Hedden, Stephen G. Thomas; Gibberellin biosynthesis and its regulation. Biochem J 15 May 2012; 444 (1): 11–25. doi: <https://doi.org/10.1042/BJ20120245>

Line 128. "forage". What does that mean? Webster's dictionary defines forage (used a noun): "Food for animals especially when taken by browsing or grazing." That is not specific. This kind of sample needs to be defined more fully.

**Response:** We thank the editor for this helpful comment. We agree that the term “forage” should be defined more clearly in the manuscript. In this study, the “forage” sample refers to the aboveground portion of the maize plant collected by cutting the plant just above the soil surface. All aboveground plant parts, including leaves, ears, and stalks, were retained, cut into approximately 5-inch segments, and pooled for each plot. We have clarified this definition in the manuscript on lines 131–133.

Line 145. What is "OlChemlm"? Is this the name of a company? Provide more information (maybe a url?).

**Response:** Yes. “OlChemlm” is the company where the plant hormone standards were purchased. We have revised the manuscript to provide the full company name and website information for clarity: OlChemIm s.r.o. (OlChemIm s.r.o.., Czech Republic; https://olchemim.cz).

Line 248. What is "SLE"? Please spell it out here. You may use the acronym after that.

## **Response:** “SLE” refers to **supported liquid extraction**. The acronym was first defined in the “Sample preparation” section as follows: “extracts were loaded into a Biotage 96-well supported liquid extraction (SLE) plate (Biotage, 8200400P01).” We have also reviewed the manuscript to ensure that the term is spelled out at first use and that the acronym is used consistently thereafter.

Line 260. And elsewhere. Do not capitalize every word in the figure legend title. In English publications, that style is reservd for books.

**Response:** We have revised the figure legend titles throughout the manuscript to use sentence case rather than title case.

Please submit your revised manuscript by Oct 08 2026 11:59PM. If you will need more time than this to complete your revisions, please reply to this message or contact the journal office at [plosone@plos.org](mailto:plosone@plos.org). When you're ready to submit your revision, log on to <https://www.editorialmanager.com/pone/> and select the 'Submissions Needing Revision' folder to locate your manuscript file.

Please include the following items when submitting your revised manuscript:

- A letter that responds to each point raised by the academic editor and reviewer(s). You should upload this letter as a separate file labeled 'Response to Reviewers'.
- A marked-up copy of your manuscript that highlights changes made to the original version. You should upload this as a separate file labeled 'Revised Manuscript with Track Changes'.
- An unmarked version of your revised paper without tracked changes. You should upload this as a separate file labeled 'Manuscript'.

If you would like to make changes to your financial disclosure, please include your updated statement in your cover letter. Guidelines for resubmitting your figure files are available below the reviewer comments at the end of this letter.

If applicable, we recommend that you deposit your laboratory protocols in protocols.io to enhance the reproducibility of your results. Protocols.io assigns your protocol its own identifier (DOI) so that it can be cited independently in the future. For instructions see: [https://journals.plos.org/plosone/s/submission-guidelines#loc-laboratory-protocols](https://eur03.safelinks.protection.outlook.com/?url=https%3A%2F%2Ftrack.editorialmanager.com%2FCL0%2Fhttps%3A%252F%252Fjournals.plos.org%252Fplosone%252Fs%252Fsubmission-guidelines%2523loc-laboratory-protocols%2F1%2F010f01a03689738b-cf63b73e-cb9d-4efc-9b04-fff01991dd55-000000%2FFDaF_A8LiV01oDA42ixOASxGnx7fDDWHyPn6t7qPeQ4%3D258&data=05%7C02%7C%7Cd82e8206fe6145751e9b08df024851f9%7Cfcb2b37b5da0466b9b830014b67a7c78%7C0%7C0%7C639232182349478284%7CUnknown%7CTWFpbGZsb3d8eyJFbXB0eU1hcGkiOnRydWUsIlYiOiIwLjAuMDAwMCIsIlAiOiJXaW4zMiIsIkFOIjoiTWFpbCIsIldUIjoyfQ%3D%3D%7C0%7C%7C%7C&sdata=riNQMtcAGZrlDibgV6p6qxuSDMRnjtcbMkyp21M4yus%3D&reserved=0). Additionally, PLOS One offers an option for publishing peer-reviewed Lab Protocol articles, which describe protocols hosted on protocols.io. Read more information on sharing protocols at [https://plos.org/protocols?utm_medium=editorial-email&utm_source=authorletters&utm_campaign=protocols](https://eur03.safelinks.protection.outlook.com/?url=https%3A%2F%2Ftrack.editorialmanager.com%2FCL0%2Fhttps%3A%252F%252Fplos.org%252Fprotocols%253Futm_medium%3Deditorial-email%2526utm_source%3Dauthorletters%2526utm_campaign%3Dprotocols%2F1%2F010f01a03689738b-cf63b73e-cb9d-4efc-9b04-fff01991dd55-000000%2F2TVH6_WpZ0KcU77RtuqOGvCHACiUv3SA7B4U0kHY4KU%3D258&data=05%7C02%7C%7Cd82e8206fe6145751e9b08df024851f9%7Cfcb2b37b5da0466b9b830014b67a7c78%7C0%7C0%7C639232182349513899%7CUnknown%7CTWFpbGZsb3d8eyJFbXB0eU1hcGkiOnRydWUsIlYiOiIwLjAuMDAwMCIsIlAiOiJXaW4zMiIsIkFOIjoiTWFpbCIsIldUIjoyfQ%3D%3D%7C0%7C%7C%7C&sdata=%2BYn5Zz9a6aDF4cAJbAeTNWp9GcrT2NZpcwMezxu0ZNM%3D&reserved=0).

As the corresponding author, your ORCID iD is verified in the submission system and will appear in the published article. PLOS supports the use of ORCID, and we encourage all coauthors to register for an ORCID iD and use it as well. Please encourage your coauthors to verify their ORCID iD within the submission system before final acceptance, as unverified ORCID iDs will not appear in the published article. *Only* the individual author can complete the verification step; PLOS staff *cannot* verify ORCID iDs on behalf of authors.
We look forward to receiving your revised manuscript.

Kind regards,

Tobias Isaac Baskin
Academic Editor
PLOS One

**Journal Requirements:**

1. If the reviewer comments include a recommendation to cite specific previously published works, please review and evaluate these publications to determine whether they are relevant and should be cited. There is no requirement to cite these works unless the editor has indicated otherwise.
2. Please review your reference list to ensure that it is complete and correct. If you have cited papers that have been retracted, please include the rationale for doing so in the manuscript text, or remove these references and replace them with relevant current references. Any changes to the reference list should be mentioned in the rebuttal letter that accompanies your revised manuscript. If you need to cite a retracted article, indicate the article’s retracted status in the References list and also include a citation and full reference for the retraction notice.

[Note: HTML markup is below. Please do not edit.]

Reviewers' comments:

Reviewer's Responses to Questions

**Comments to the Author**

1. If the authors have adequately addressed your comments raised in a previous round of review and you feel that this manuscript is now acceptable for publication, you may indicate that here to bypass the “Comments to the Author” section, enter your conflict of interest statement in the “Confidential to Editor” section, and submit your "Accept" recommendation.

Reviewer #3: All comments have been addressed

Reviewer #4: (No Response)

2. Is the manuscript technically sound, and do the data support the conclusions?

The manuscript must describe a technically sound piece of scientific research with data that supports the conclusions. Experiments must have been conducted rigorously, with appropriate controls, replication, and sample sizes. The conclusions must be drawn appropriately based on the data presented.

Reviewer #3: Partly

Reviewer #4: Yes

3. Has the statistical analysis been performed appropriately and rigorously?

Reviewer #3: N/A

Reviewer #4: Yes

4. Have the authors made all data underlying the findings in their manuscript fully available?

The [PLOS Data policy](https://eur03.safelinks.protection.outlook.com/?url=http%3A%2F%2Ftrack.editorialmanager.com%2FCL0%2Fhttp%3A%252F%252Fwww.plosone.org%252Fstatic%252Fpolicies.action%2523sharing%2F1%2F010f01a03689738b-cf63b73e-cb9d-4efc-9b04-fff01991dd55-000000%2FRlGYVMSegZIE7q5B0Wj1B3E46OFYFC3zPUTkNVhODnU%3D258&data=05%7C02%7C%7Cd82e8206fe6145751e9b08df024851f9%7Cfcb2b37b5da0466b9b830014b67a7c78%7C0%7C0%7C639232182349533208%7CUnknown%7CTWFpbGZsb3d8eyJFbXB0eU1hcGkiOnRydWUsIlYiOiIwLjAuMDAwMCIsIlAiOiJXaW4zMiIsIkFOIjoiTWFpbCIsIldUIjoyfQ%3D%3D%7C0%7C%7C%7C&sdata=Z%2FdNsSYe2EUz8nMS8HPYduA35PoLFVuDBPMXkn4rgxI%3D&reserved=0) requires authors to make all data underlying the findings described in their manuscript fully available without restriction, with rare exception (please refer to the Data Availability Statement in the manuscript PDF file). The data should be provided as part of the manuscript or its supporting information, or deposited to a public repository. For example, in addition to summary statistics, the data points behind means, medians and variance measures should be available. If there are restrictions on publicly sharing data—e.g. participant privacy or use of data from a third party—those must be specified.

Reviewer #3: Yes

Reviewer #4: Yes

5. Is the manuscript presented in an intelligible fashion and written in standard English?

PLOS ONE does not copyedit accepted manuscripts, so the language in submitted articles must be clear, correct, and unambiguous. Any typographical or grammatical errors should be corrected at revision, so please note any specific errors here.

Reviewer #3: Yes

Reviewer #4: Yes

6. Review Comments to the Author

Please use the space provided to explain your answers to the questions above. You may also include additional comments for the author, including concerns about dual publication, research ethics, or publication ethics. (Please upload your review as an attachment if it exceeds 20,000 characters)

Reviewer #3: (No Response)

Reviewer #4: This paper investigates the genetic and environmental contributions to variations in GA1 and GA4 levels across maize tissues using linear mixed-effects models. The authors conclude that environmental conditions contribute more than genetic background in some tissues. The statistical methodology is sound, and below are some minor questions and comments:

- The authors indicate that an additional analysis was conducted treating Hybrid, Site, and the Hybrid-by-Site interaction as fixed effects (lines 222-232). It might be helpful to refer to this when you show results of this analysis, for example, I assume this model is used to calculate quantities in Fig.4, is that correct?

**Response:** We thank the reviewer for this helpful suggestion. Yes. This model was used to calculate quantities shown in Fig 4. To make this clearer, we have revised the Results section of the manuscript, Lines 324-327, to state: “The analyses were conducted within a linear mixed model framework using PROC MIXED. Hybrid, Site, and the Hybrid-by-Site interaction were treated as fixed effects, whereas Replicate nested within Site was modeled as a random effect.”

- The author imputes missing data with half of the smallest observed value of a certain tissue. Some sensitivity analysis appropriate for the missing data mechanism might be helpful to clarify whether this affects the estimated variance components, e.g., if the measurement tends to be truncated then you can use a model that assumes the response is truncated at a certain value.

**Response:** We thank the reviewer for this helpful suggestion. To evaluate whether the handling of ND values affected the statistical conclusions, we conducted a sensitivity analysis using two imputation approaches: (1) substituting all ND values with a single value, defined as ½ of the LLOQ, and (2) substituting ND values with tissue-specific values, defined as ½ of the smallest observed value for each tissue, with the tissue-specific values provided in Supplemental Table S2. The results were consistent between the two approaches, including the estimated variance components and statistical significance. Therefore, the conclusions were not sensitive to the imputation method used for ND values.

Because the current approach is conventional for values below the detection or quantification threshold and produced consistent results across the sensitivity analysis, we retained this method in the revised manuscript.

- The authors mention that trial maintenance varied across sites. Could this be a potential confounder? It would be helpful if the author can discuss that a bit when they describe what the environmental effect entails.

**Response**: We agree that differences in trial maintenance among sites could potentially contribute to confounding between Hybrid and Site (environment), particularly given the imbalanced design of this experiment. In the manuscript, we describe the nature of this imbalance and discuss its implications for interpreting hybrid-by-environment interactions. The mixed effects model was selected to account for site-related variation and the imbalance in the data when estimating treatment effects. Therefore, the interaction estimates presented in Table 6 are model adjusted estimates that account for these factors.

It might be helpful to clarify how the standard error in Fig. 3 calculated, for example whether this the standard errors of replicates within each site/hybrid combination or were calculated using an alternative method.

**Response:** Thanks for the comments. The error bars in Fig. 3 represent the standard errors of the predicted mean for each site/hybrid combination from fitting the mixed-effect model. We have clarified this in the manuscript (lines 304-305) by adding the sentence “The standard errors are for the predicted mean of each site/hybrid combination from fitting the mixed-effect model”.

7. PLOS authors have the option to publish the peer review history of their article ([what does this mean?](https://eur03.safelinks.protection.outlook.com/?url=https%3A%2F%2Ftrack.editorialmanager.com%2FCL0%2Fhttps%3A%252F%252Fjournals.plos.org%252Fplosone%252Fs%252Feditorial-and-peer-review-process%2523loc-peer-review-history%2F1%2F010f01a03689738b-cf63b73e-cb9d-4efc-9b04-fff01991dd55-000000%2FiNCm0RjjT8jYsC5iIaYBLOdOuLWsdj4MLy8N2tuBFKQ%3D258&data=05%7C02%7C%7Cd82e8206fe6145751e9b08df024851f9%7Cfcb2b37b5da0466b9b830014b67a7c78%7C0%7C0%7C639232182349551458%7CUnknown%7CTWFpbGZsb3d8eyJFbXB0eU1hcGkiOnRydWUsIlYiOiIwLjAuMDAwMCIsIlAiOiJXaW4zMiIsIkFOIjoiTWFpbCIsIldUIjoyfQ%3D%3D%7C0%7C%7C%7C&sdata=gWPT%2Fb3%2FeEWu6SShnX%2FQ6ZdHy98i%2BYPQ%2FrSxlp2ffJg%3D&reserved=0)). If published, this will include your full peer review and any attached files.

If you choose “no”, your identity will remain anonymous but your review may still be made public.

**Do you want your identity to be public for this peer review?** For information about this choice, including consent withdrawal, please see our [Privacy Policy](https://eur03.safelinks.protection.outlook.com/?url=https%3A%2F%2Ftrack.editorialmanager.com%2FCL0%2Fhttps%3A%252F%252Fwww.plos.org%252Fprivacy-policy%2F1%2F010f01a03689738b-cf63b73e-cb9d-4efc-9b04-fff01991dd55-000000%2FRv5EX7oxSaVlape2byQ_yB28KywDgfypqb6qZZzC2kg%3D258&data=05%7C02%7C%7Cd82e8206fe6145751e9b08df024851f9%7Cfcb2b37b5da0466b9b830014b67a7c78%7C0%7C0%7C639232182349572087%7CUnknown%7CTWFpbGZsb3d8eyJFbXB0eU1hcGkiOnRydWUsIlYiOiIwLjAuMDAwMCIsIlAiOiJXaW4zMiIsIkFOIjoiTWFpbCIsIldUIjoyfQ%3D%3D%7C0%7C%7C%7C&sdata=pte6za0M6xKbuaZyxTOFxK0Rq5mgrbsXPGJzywkpWM4%3D&reserved=0).

Reviewer #3: No

Reviewer #4: No

[NOTE: If reviewer comments were submitted as an attachment file, they will be attached to this email and accessible via the submission site. Please log into your account, locate the manuscript record, and check for the action link "View Attachments". If this link does not appear, there are no attachment files.]

To ensure your figures meet our technical requirements, please review our figure guidelines: <https://journals.plos.org/plosone/s/figures>

You may also use PLOS’s free figure tool, NAAS, to help you prepare publication quality figures: <https://journals.plos.org/plosone/s/figures#loc-tools-for-figure-preparation>.

NAAS will assess whether your figures meet our technical requirements by comparing each figure against our figure specifications.
