# Supplementary material for: Natural variation of bioactive gibberellin levels in maize tissues across North America
Source: PLoS One. 2026 Oct 7;21(10):e0340156. doi: 10.1371/journal.pone.0340156 (PMC13645078; doi:10.1371/journal.pone.0340156)
Supplement: Peer review history

# Peer review: Decision Letter 0

**DOI:** 10.1371/journal.pone.0340156.r001

**Authors:** Tobias Baskin

5 Mar 2026

Dear Dr. Wang,

Thank you for submitting your manuscript to PLOS ONE. After careful consideration, we feel that it has merit but does not fully meet PLOS ONE’s publication criteria as it currently stands. Therefore, we invite you to submit a revised version of the manuscript that addresses the points raised during the review process.

Please submit your revised manuscript by Apr 19 2026 11:59PM. If you will need more time than this to complete your revisions, please reply to this message or contact the journal office at plosone@plos.org. When you're ready to submit your revision, log on to https://www.editorialmanager.com/pone/ and select the 'Submissions Needing Revision' folder to locate your manuscript file.

- A letter that responds to each point raised by the academic editor and reviewer(s). You should upload this letter as a separate file labeled 'Response to Reviewers'.
- A marked-up copy of your manuscript that highlights changes made to the original version. You should upload this as a separate file labeled 'Revised Manuscript with Track Changes'.
- An unmarked version of your revised paper without tracked changes. You should upload this as a separate file labeled 'Manuscript'.

If you would like to make changes to your financial disclosure, please include your updated statement in your cover letter. Guidelines for resubmitting your figure files are available below the reviewer comments at the end of this letter.

If applicable, we recommend that you deposit your laboratory protocols in protocols.io to enhance the reproducibility of your results. Protocols.io assigns your protocol its own identifier (DOI) so that it can be cited independently in the future. For instructions see: https://journals.plos.org/plosone/s/submission-guidelines#loc-laboratory-protocols. Additionally, PLOS ONE offers an option for publishing peer-reviewed Lab Protocol articles, which describe protocols hosted on protocols.io. Read more information on sharing protocols at https://plos.org/protocols?utm_medium=editorial-email&utm_source=authorletters&utm_campaign=protocols.

We look forward to receiving your revised manuscript.

Kind regards,

Tobias Isaac Baskin

Academic Editor

PLOS One

Journal Requirements:

When submitting your revision, we need you to address these additional requirements.

1.Please ensure that your manuscript meets PLOS ONE's style requirements, including those for file naming. The PLOS ONE style templates can be found at

https://journals.plos.org/plosone/s/file?id=wjVg/PLOSOne_formatting_sample_main_body.pdf and

https://journals.plos.org/plosone/s/file?id=ba62/PLOSOne_formatting_sample_title_authors_affiliations.pdf

2. Please note that funding information should not appear in any section or other areas of your manuscript. We will only publish funding information present in the Funding Statement section of the online submission form. Please remove any funding-related text from the manuscript.

3. Thank you for stating the following in the Competing Interests section:

“The authors have declared that no competing interests exist.”

We note that one or more of the authors are employed by a commercial company: Bayer CropScience LLC

1.        Please provide an amended Funding Statement declaring this commercial affiliation, as well as a statement regarding the Role of Funders in your study. If the funding organization did not play a role in the study design, data collection and analysis, decision to publish, or preparation of the manuscript and only provided financial support in the form of authors' salaries and/or research materials, please review your statements relating to the author contributions, and ensure you have specifically and accurately indicated the role(s) that these authors had in your study. You can update author roles in the Author Contributions section of the online submission form.

Please also include the following statement within your amended Funding Statement.

“The funder provided support in the form of salaries for authors [insert relevant initials], but did not have any additional role in the study design, data collection and analysis, decision to publish, or preparation of the manuscript. The specific roles of these authors are articulated in the ‘author contributions’ section.”

If your commercial affiliation did play a role in your study, please state and explain this role within your updated Funding Statement.

2. Please also provide an updated Competing Interests Statement declaring this commercial affiliation along with any other relevant declarations relating to employment, consultancy, patents, products in development, or marketed products, etc.

Within your Competing Interests Statement, please confirm that this commercial affiliation does not alter your adherence to all PLOS ONE policies on sharing data and materials by including the following statement: "This does not alter our adherence to  PLOS ONE policies on sharing data and materials.” (as detailed online in our guide for authors http://journals.plos.org/plosone/s/competing-interests) . If this adherence statement is not accurate and  there are restrictions on sharing of data and/or materials, please state these. Please note that we cannot proceed with consideration of your article until this information has been declared.

Please include both an updated Funding Statement and Competing Interests Statement in your cover letter. We will change the online submission form on your behalf.

4. Please include your full ethics statement in the ‘Methods’ section of your manuscript file. In your statement, please include the full name of the IRB or ethics committee who approved or waived your study, as well as whether or not you obtained informed written or verbal consent. If consent was waived for your study, please include this information in your statement as well.

5. We note that Figure 1 in your submission contain copyrighted images. All PLOS content is published under the Creative Commons Attribution License (CC BY 4.0), which means that the manuscript, images, and Supporting Information files will be freely available online, and any third party is permitted to access, download, copy, distribute, and use these materials in any way, even commercially, with proper attribution. For more information, see our copyright guidelines: http://journals.plos.org/plosone/s/licenses-and-copyright.

We require you to either (1) present written permission from the copyright holder to publish these figures specifically under the CC BY 4.0 license, or (2) remove the figures from your submission:

1. You may seek permission from the original copyright holder of Figure 1 to publish the content specifically under the CC BY 4.0 license.

We recommend that you contact the original copyright holder with the Content Permission Form (http://journals.plos.org/plosone/s/file?id=7c09/content-permission-form.pdf) and the following text:

“I request permission for the open-access journal PLOS ONE to publish XXX under the Creative Commons Attribution License (CCAL) CC BY 4.0 (http://creativecommons.org/licenses/by/4.0/). Please be aware that this license allows unrestricted use and distribution, even commercially, by third parties. Please reply and provide explicit written permission to publish XXX under a CC BY license and complete the attached form.”

Please upload the completed Content Permission Form or other proof of granted permissions as an "Other" file with your submission.

In the figure caption of the copyrighted figure, please include the following text: “Reprinted from [ref] under a CC BY license, with permission from [name of publisher], original copyright [original copyright year].”

2. If you are unable to obtain permission from the original copyright holder to publish these figures under the CC BY 4.0 license or if the copyright holder’s requirements are incompatible with the CC BY 4.0 license, please either i) remove the figure or ii) supply a replacement figure that complies with the CC BY 4.0 license. Please check copyright information on all replacement figures and update the figure caption with source information. If applicable, please specify in the figure caption text when a figure is similar but not identical to the original image and is therefore for illustrative purposes only.

6. We notice that your supplementary tables are included in the manuscript file. Please remove them and upload them with the file type 'Supporting Information'. Please ensure that each Supporting Information file has a legend listed in the manuscript after the references list.

7. If the reviewer comments include a recommendation to cite specific previously published works, please review and evaluate these publications to determine whether they are relevant and should be cited. There is no requirement to cite these works unless the editor has indicated otherwise.

Additional Editor Comments:

Thank you for submitting your work to our journal. Your work has been read by two reviewers who both found substantial flaws. Reviewer 1 is a statistician. This reviewers points out places with missing or confusing information with respect to experimental design and interpretation. These points need addressing before a reader can have confidence in your approach. Reviewer 2 is a biologist; they point out issues with respect to plant development. Gibberellin varies within an organ and over time as the organ develops. In the best case, this axis would be sampled but at the very least it needs to be addressed.

I have a three additional points. First, please spell out gibberellin when used to refer to the hormone in general. Gibberellin is a lovely word, echoing as it does the word gibberish, inspired by the foolish seedling disease that led to the hormone's discovery. Scientists have come to think that they are supposed to abbreviate words of their discourse but readers find this practice unhelpful. We prefer to read words over symbols. Familiarity is irrelevant.

The other day, I saw this with respect to current culture war on-going in the U.S.A. “If you oppose DEI you should have to use the full phase instead of the letters. Be brave and say, I oppose diversity, equity, and inclusion. Making it an acronym transforms it into a thought-terminating cliche.” While thought “terminating” is perhaps an exaggeration, thought “suppressing” is correct. As scientists we are urged to communicate better; the first and easiest thing we can do toward that end is to write with words.

In the case of gibberellin, a compromise can be to use GA1 (etc.) for the specific molecule (although I encourage you to bite this bullet and use gibberellin-1).

Second, PLoS One publishes papers that are valid with no reflection on whether they are important or even interesting. Because most journals want the work to be more than valid, writers tend to pitch the importance of their story. But this gets in the way here. What is needed is simply to introduce the reader to the problem, say what you did to solve it, and what you think it means. There is no need to stretch beyond the straightforward.

Third, please use separate results and discussion sections. The combination is useful for a short paper, for example with one figure. But your paper is full-size. Present the results; then discuss. This format has persisted for years because it is helpful.

[Note: HTML markup is below. Please do not edit.]

Reviewers' comments:

Reviewer's Responses to Questions

**Comments to the Author**

1. Is the manuscript technically sound, and do the data support the conclusions?

Reviewer #1: Partly

Reviewer #2: Partly

**********

2. Has the statistical analysis been performed appropriately and rigorously?

Reviewer #1: No

Reviewer #2: Yes

**********

3. Have the authors made all data underlying the findings in their manuscript fully available?

The PLOS Data policy

Reviewer #1: Yes

Reviewer #2: Yes

**********

4. Is the manuscript presented in an intelligible fashion and written in standard English?

Reviewer #1: Yes

Reviewer #2: Yes

**********

Reviewer #1: I focus my comments mainly on the statistical aspects of the manuscript. The study benefits from a good spread of geographical locations, and it is sensible in aiming to use a classical experimental design and linear statistical model. However, important details on the design are missing, and too much is wrapped up in the sentence “Each site was planted in a randomized complete block design with four replicated blocks. Normal agronomic practices were followed for each site.” It is unclear what the “unit” of planting was. What, physically, were the four blocks, and how were they established? For the hybrids listed at each particular site, does each hybrid (at that site) appear in each block? What was the plot size and layout, was there any obvious spatial heterogeneity? After growing, how was sampling carried out? How many plants per plot were sampled, and were samples from single plants or pooled? These points matter for interpreting both the structure of the model and the meaning of the variance components.

The authors note (Table 1) the unbalanced design with respect to hybrids (some appear once, some at several sites, none of them appears at all six). There is no obvious or systematic overlap structure, and I assume this was driven by logistical constraints rather than design. Nevertheless, it warrants careful discussion because it strongly affects what can be inferred: in particular, the genotype (hybrid) × environment (site) interaction will be poorly identified. With such an incomplete hybrid–site crossing, the data contain (possibly very) limited information to identify hybrid main effects, site effects, and their interaction.

In the linear model, a log transform is used for the response variable. Some explanation of the motivation would be helpful. There is clearly enormous variation (Table 4), spanning four orders of magnitude for some tissue types, which perhaps drives that choice. But is that range biologically plausible, or does it raise concerns about quantification? The fact that some observations fall below detection limits and need to be replaced by a small non-zero value so that the log transformation is defined further raises questions about sensitivity to the choice of imputed value. A simple sensitivity analysis (replacing the imputed value with something smaller and something larger and checking that the conclusions are robust) would be valuable here.

For the model itself, eqn. (1) seems broadly reasonable, but because the experimental design is not clearly described, it is difficult to understand exactly what the indices run over and how many parameters the model entails. For example, an interaction term cannot be fitted for a site–hybrid combination for which no data exist (e.g. Jefferson County × Stone 6290). The authors state that they use an “all‑random‑effect” model, which is perhaps meant to address this, but then immediately switch to a fixed‑effects model (“further investigation was made for the mean changes of individual hybrid across sites… a fixed‑effect model was assumed and least-square means were estimated”). It is unclear what fixed‑effects model is meant here, i.e., which terms are treated as fixed and which as random. The following paragraph (around line 202) switches back to discussion of random effects. This is confusing, because the distinction between fixed and random effects is not simply a matter of computational convenience but relates to the assumptions and interpretation of the model. The hybrids in question are specific, named commercial hybrids, not a random sample from a hypothetical population of hybrids, so treating them as random requires justification. Even if the SAS procedures return variance‑component estimates, the standard likelihood/chi-squared‑based testing assumptions (which I believe the SAS procedures will use) are almost certainly unreliable in designs as unbalanced as the present one - and in view of that, the resulting p‑values and variance partitions should be interpreted with caution/caveats. Further, I did not understand the description of the statistical procedures well enough to know whether the authors were alert to, and accounted for, multiple testing.

The discussion how positive correlations between GA₁ and GA₄ concentrations could be attributed to regulatory pathways doesn't seem implausible, but isn't a simpler explanation just that both GA₁ and GA₄ are higher when plants are growing more vigorously, i.e. that overall physiological “health” could be a confounder? And indeed isn't "health" itself substantially environmentally driven by "quality of growing conditions"? (This is consistent with the authors finding that the site effect was very significant; this being field data, the authors could presumably not control for weather variation between sites.) The manuscript moves quickly from correlation to, for example, speculation about shared transcriptional networks which whilst possible, seems quite a leap beyond what the data alone suggest.

Some suggested actions:

- clarify the experimental design (describing clearly the "plot"s, block structure, what appears in what blocks, etc)

- describe fully the sampling (how many plants, which were selected for measurement, whether there was pooling, etc)

- explain the consequences of the design imbalance (related in particular to the hybrid x site interaction)

- [to help with the preceding bullet:] report CIs, not just significance test results, for the variance components

- clarify the statistical modelling: at different stages, what is treated as random and what is treated as fixed? (with explanation for these choices)

- clarify whether there were distinct multiple hypothesis tests, and if so whether multiple testing was accounted for in reporting the significance of the results

- discuss/justify the log transformation more, including a discussion of whether the extreme range of experimental measurements is biologically reasonable

- if there is value imputation (for effective zeros in the experimental data) perform a sensitivity analysis that the choice of imputed value isn't too consequential to the conclusions

- temper the interpretation/claims in places, e.g. related to the GA1 and GA4 concentrations. The last sentence of the abstract also feels a bit overreaching in relation to the results mentioned before it in the abstract

Reviewer #2: The paper deals with large analysis of the two most bioactive GAs GA1 and GA4 in 6 different tissues of maize plants of different hybrid varieties and grown on a number of contrasting field sites.

The analysis is performed mainly by performing a correlation analysis, of which the findings appear to be that GA1 and 4 broadly covary, that there are large differences in GA levels in different tissues and that the environment has a stronger effect than Genotype, although both are significant.

I’m not enough of an expert to evaluate to what extent the analytical method is state of the art and what the quality of the measurements is.

Other than that the manuscript fails to convince.

In the introduction literature is reviewed about previous analyses of GA, but non of the work on spatial distributions of GA in growing organs or the impact of environmental factors on GA levels.

A lot of space is taken to explain the technical details of the method, even the whole first section of the results is devoted to that. Nevertheless it is not clear what exactly was sampled. Which (parts of) leaves, roots, silks etc did they harvest, what was the developmental stage of the plant, harvest time of day, etc. All of these are expected have a significant effect on the measurements.

The results basically just deal with the observed variations, but we learn nothing about the biological meaning of these observations, so in the end there seems very little new insight.

In the discussion the authors suggest that we have learned a lot on the regulation of GA regulation, but I fail to see how they assessed regulatory mechanisms or effects.

Curiously they state to be surprised to see that there is a significant covariation between GA1 and GA4, given the (according to them) distinct synthetic pathways. This ignores the topology of the biosynthetic pathway: many of the enzymes are involved with the synthesis of both bioactive GAs. The upstream pathway up to GA12 is shared and only GA13ox transforms GA12 a precursor of GA4 to GA97 a precursor of GA1. All enzymes working GA12 and GA97 are identical creating two paralel branches regulated by the same enzymes.

In this light it is quite logical there is a covariation.

So altogether this analysis and the manuscript as a whole to my mind is not yet suitable for publication.

**********

what does this mean?). If published, this will include your full peer review and any attached files.

If you choose “no”, your identity will remain anonymous but your review may still be made public.

**Do you want your identity to be public for this peer review?** For information about this choice, including consent withdrawal, please see our Privacy Policy

Reviewer #1: No

Reviewer #2: **Yes:**  Gerrit Beemster

**********

[NOTE: If reviewer comments were submitted as an attachment file, they will be attached to this email and accessible via the submission site. Please log into your account, locate the manuscript record, and check for the action link "View Attachments". If this link does not appear, there are no attachment files.]

To ensure your figures meet our technical requirements, please review our figure guidelines: https://journals.plos.org/plosone/s/figures

You may also use PLOS’s free figure tool, NAAS, to help you prepare publication quality figures: https://journals.plos.org/plosone/s/figures#loc-tools-for-figure-preparation.

NAAS will assess whether your figures meet our technical requirements by comparing each figure against our figure specifications.

# Author comment: Author response to Decision Letter 1

**DOI:** 10.1371/journal.pone.0340156.r002

29 May 2026

Dear Academic editor,

We are grateful for the opportunity to refine our work and resubmit it for your consideration. Please find our responses in blue below. Please note, we included all three response to reviewers in this document. Please let us know if there is any additional information you need or if further clarification is required.

Thank you once again for your guidance and support throughout the revision process.

Warm regards,

Yanfei Wang

Response to reviewer 3

PONE-D-25-66749R1

Natural variation of gibberellin levels in maize tissues across North America Dr. Yanfei Wang

Dear Dr. Wang,

We've checked your submission and before we can proceed, we need you to address the following issues:

1. Thank you for updating Figure 1. The revised illustrations are noted. Could you please confirm that these new images were created by the authors without the use of AI-generation tools? This is simply to ensure our records are accurate given the earlier versions.

Response: Figure 1 in the revised manuscript is an original author-created illustration of our workflow that can be licensed under CC BY 4.0. The submitted figure was prepared by the authors using standard drawing tools and was not copied or traced from any AI-generated image. No AI-generation tools were used in creating this figure.

We look forward to hearing from you.

We've returned your manuscript to your account. Please resolve these issues and resubmit your manuscript within 21 days. If you need more time, please email the journal office at plosone@plos.org. We are happy to grant extensions of up to one month past this due date. If we do not hear from you within 21 days, we will withdraw your manuscript.

Please log on to PLOS Editorial Manager at https://eur03.safelinks.protection.outlook.com/?url=https%3A%2F%2Fwww.editorialmanager.com%2Fpone%2F&data=05%7C02%7C%7C5da17ce380344a69416208debc7966d0%7Cfcb2b37b5da0466b9b830014b67a7c78%7C0%7C0%7C639155426756799707%7CUnknown%7CTWFpbGZsb3d8eyJFbXB0eU1hcGkiOnRydWUsIlYiOiIwLjAuMDAwMCIsIlAiOiJXaW4zMiIsIkFOIjoiTWFpbCIsIldUIjoyfQ%3D%3D%7C0%7C%7C%7C&sdata=S81mgknf8OqG7qd2yCZZSLUw13iRoSajfQTTG1Ds6js%3D&reserved=0 to access your manuscript. You will find your manuscript in the 'Submissions Sent Back to Author' link under the New Submissions menu. Be sure to remove your previous manuscript file if you are uploading a new file in response to these requests. After you've made the changes requested above, please be sure to view and approve the revised PDF after rebuilding the PDF to complete the resubmission process.

We are requesting these changes to comply with the PLOS ONE submission guidelines (https://eur03.safelinks.protection.outlook.com/?url=https%3A%2F%2Fjournals.plos.org%2Fplosone%2Fs%2Fsubmission-guidelines&data=05%7C02%7C%7C5da17ce380344a69416208debc7966d0%7Cfcb2b37b5da0466b9b830014b67a7c78%7C0%7C0%7C639155426756872182%7CUnknown%7CTWFpbGZsb3d8eyJFbXB0eU1hcGkiOnRydWUsIlYiOiIwLjAuMDAwMCIsIlAiOiJXaW4zMiIsIkFOIjoiTWFpbCIsIldUIjoyfQ%3D%3D%7C0%7C%7C%7C&sdata=GMhbQcjkuCCgcxzxa0vt1ktIfpE0mIrhunlZrJxPYPw%3D&reserved=0). Please note that we won't send your manuscript for review until you have resolved the above requests.

Thank you for submitting your work to PLOS ONE and supporting our mission of Open Science.

Kind regards,

Gema Queenie Salem

PLOS One

Response to reviewer 2

ONE-D-25-66749R1

Natural variation of gibberellin levels in maize tissues across North America Dr. Yanfei Wang

Dear Dr. Wang,

We've checked your submission and before we can proceed, we need you to address the following issues:

1. We note that your author list was updated during the revision process. In order to add or remove authors or update the order of the author byline after initial submission, we ask that authors complete an Authorship Change Request form. You may review our full authorship change policy and download the Authorship Change Request form here: https://eur03.safelinks.protection.outlook.com/?url=https%3A%2F%2Fjournals.plos.org%2Fplosone%2Fs%2Fauthorship%23loc-authorship-changes&data=05%7C02%7C%7C4eafb8e3435147c5cdee08dea4c00395%7Cfcb2b37b5da0466b9b830014b67a7c78%7C0%7C0%7C639129341761324165%7CUnknown%7CTWFpbGZsb3d8eyJFbXB0eU1hcGkiOnRydWUsIlYiOiIwLjAuMDAwMCIsIlAiOiJXaW4zMiIsIkFOIjoiTWFpbCIsIldUIjoyfQ%3D%3D%7C0%7C%7C%7C&sdata=fGKs8plcZP7khUMVwLM8LSBxBz5j4MoNO7L7XFq5oYA%3D&reserved=0.

Please fill out all required sections of the form. If you are adding or removing more than 2 authors, you can complete multiple forms and submit as many forms as needed to reflect the updates. Please return the form(s) as an attachment by emailing plosone@plos.org or by uploading it as a submission file labeled with the file type ‘Other’. The form will be reviewed by PLOS staff for approval. Should the changes be approved, we will require written confirmation from all authors confirming that they approve of the changes. Please note that if your manuscript is accepted, we will not be able to complete the publication process without the completed form.

Response: The completed Authorship Change Request form has been uploaded as a submission file labeled “Other.” In addition, the signed Authorship Change Request form, including e-signatures from all authors, is attached under the file type “Other” as well.

2. Thank you for including your ethics statement on the online submission form: " Release Location (County, State) USDA EPA/EUP Permit Permit Jefferson County AUTH - 0000233243 524-EUP-117 IA Shelby County AUTH - 0000233243 524-EUP-117 IL

Boone County AUTH - 0000233243 524-EUP-117 IN Adair County AUTH - 0000233243 524-EUP-117 MO York County AUTH - 0000233243 524-EUP-117 NE Miami County AUTH - 0000233243 524-EUP-117 OH ". To help ensure that the wording of your manuscript is suitable for publication, would you please also add this statement at the beginning of the Methods section of your manuscript file.

Response: We have added the ethics statement to the beginning of the Methods section of the manuscript, consistent with the information provided in the submission form. Specifically, we added the following statement at line 120: “Plant materials consisted of conventional crop varieties grown in experimental plots that were collocated with USDA-permitted materials under Authorization No. AUTH-0000233243.”

Maize field trials

Maize samples from ten commercially available conventional maize hybrids were collected from field trials during the 2023 growing season. Six field trial locations were spread across states representing major maize-growing regions of the United States (Iowa, Illinois, Indiana, Missouri, Nebraska, and Ohio) (Table 1). Plant materials consisted of conventional crop varieties grown in experimental plots that were collocated with USDA permitted materials under Authorization No. AUTH-0000233243.

Table 1. Field trial locations and maize hybrid names

Site Code County, State Hybrid Name

Site 1 Jefferson County, Iowa Dekalb DKC58-32

Lewis 12CV779

Channel 210-79

Site 2 Shelby County, Illinois Stone 6290

Hubner H2390

Dekalb DKC65-92

Site 3 Boone County, Indiana Dekalb DKC63-58

Hubner H2390

Site 4 Adair County, Missouri Dekalb DKC58-32

Lewis 12CV779

Stone 6070

Site 5 York County, Nebraska Stone 6290

Hubner H2390

Dekalb DKC64-32

Site 6 Miami County, Ohio Dekalb DKC58-32

Stewart 12CV209

Channel 210-79

3. Thank you for your explanation regarding Figure 1 and for confirming that the original version was generated using your internal ChatGPT system and has now been manually redrawn to avoid licensing issues.

PLOS has specific guidance for figures that involve generative AI tools. To ensure that Figure 1 is fully compatible with the CC BY 4.0 license, could you please clarify the following:

- Tool and terms of use

Please specify which generative AI service your “internal ChatGPT” is based on (for example, an enterprise instance of OpenAI’s ChatGPT, or another provider), and provide the direct URL to the Terms of Use governing that service.

- Relationship between the AI output and the current figure

Please confirm whether the newly redrawn Figure 1 was created without directly copying or tracing any part of the AI generated image, and that the current version can be considered an original author created illustration that you are entitled to license under CC BY 4.0.

- Figure legend disclosure (if applicable)

If any elements of the current figure were materially based on an AI generated output (e.g., used as a design/template that has been refined), PLOS requires that this be clearly disclosed in the figure legend (for example: “Figure concept was initially drafted using a generative AI tool (internal ChatGPT); this version was manually redrawn by the authors.”).

Once we have this clarification, we’ll be able to confirm that Figure 1 complies with PLOS copyright and AI use requirements.

Response: Figure 1 in the revised manuscript is an original author-created illustration of our workflow that can be licensed under CC BY 4.0. The submitted figure was prepared by the authors using standard drawing tools and was not copied or traced from any AI-generated image.

We've returned your manuscript to your account. Please resolve these issues and resubmit your manuscript within 21 days. If you need more time, please email the journal office at plosone@plos.org. We are happy to grant extensions of up to one month past this due date. If we do not hear from you within 21 days, we will withdraw your manuscript.

Please log on to PLOS Editorial Manager at https://eur03.safelinks.protection.outlook.com/?url=https%3A%2F%2Fwww.editorialmanager.com%2Fpone%2F&data=05%7C02%7C%7C4eafb8e3435147c5cdee08dea4c00395%7Cfcb2b37b5da0466b9b830014b67a7c78%7C0%7C0%7C639129341761353939%7CUnknown%7CTWFpbGZsb3d8eyJFbXB0eU1hcGkiOnRydWUsIlYiOiIwLjAuMDAwMCIsIlAiOiJXaW4zMiIsIkFOIjoiTWFpbCIsIldUIjoyfQ%3D%3D%7C0%7C%7C%7C&sdata=ewSYp6I4078AA%2B4zwEjBVhQRuRfaJd7VHyFLGjl5r%2Fg%3D&reserved=0 to access your manuscript. You will find your manuscript in the 'Submissions Sent Back to Author' link under the New Submissions menu. Be sure to remove your previous manuscript file if you are uploading a new file in response to these requests. After you've made the changes requested above, please be sure to view and approve the revised PDF after rebuilding the PDF to complete the resubmission process.

We are requesting these changes to comply with the PLOS ONE submission guidelines (https://eur03.safelinks.protection.outlook.com/?url=https%3A%2F%2Fjournals.plos.org%2Fplosone%2Fs%2Fsubmission-guidelines&data=05%7C02%7C%7C4eafb8e3435147c5cdee08dea4c00395%7Cfcb2b37b5da0466b9b830014b67a7c78%7C0%7C0%7C639129341761373970%7CUnknown%7CTWFpbGZsb3d8eyJFbXB0eU1hcGkiOnRydWUsIlYiOiIwLjAuMDAwMCIsIlAiOiJXaW4zMiIsIkFOIjoiTWFpbCIsIldUIjoyfQ%3D%3D%7C0%7C%7C%7C&sdata=JIuAUGPyJjTDlIOT%2BVDU4P3CypeNInl2az924nZi9cE%3D&reserved=0). Please note that we won't send your manuscript for review until you have resolved the above requests.

Thank you for submitting your work to PLOS ONE and supporting our mission of Open Science.

Kind regards,

Gema Queenie Salem

PLOS One

Response to reviewer 1

PONE-D-25-66749

Natural variation of gibberellin levels in maize tissues across North America

PLOS One

Dear Dr. Wang,

Thank you for submitting your manuscript to PLOS ONE. After careful consideration, we feel that it has merit but does not fully meet PLOS ONE’s publication criteria as it currently stands. Therefore, we invite you to submit a revised version of the manuscript that addresses the points raised during the review process.

Please submit your revised manuscript by Apr 19 2026 11:59PM. If you will need more time than this to complete your revisions, please reply to this message or contact the journal office at plosone@plos.org. When you're ready to submit your revision, log on to https://www.editorialmanager.com/pone/ and select the 'Submissions Needing Revision' folder to locate your manuscript file.

Please include the following items when submitting your revised manuscript:

• A letter that responds to each point raised by the academic editor and reviewer(s). You should upload this letter as a separate file labeled 'Response to Reviewers'.

• A marked-up copy of your manuscript that highlights changes made to the original version. You should upload this as a separate file labeled 'Revised Manuscript with Track Changes'.

• An unmarked version of your revised paper without tracked changes. You should upload this as a separate file labeled 'Manuscript'.

If you would like to make changes to your financial disclosure, please include your updated statement in your cover letter. Guidelines for resubmitting your figure files are available below the reviewer comments at the end of this letter.

If applicable, we recommend that you deposit your laboratory protocols in protocols.io to enhance the reproducibility of your results. Protocols.io assigns your protocol its own identifier (DOI) so that it can be cited independently in the future. For instructions see: https://journals.plos.org/plosone/s/submission-guidelines#loc-laboratory-protocols. Additionally, PLOS ONE offers an option for publishing peer-reviewed Lab Protocol articles, which describe protocols hosted on protocols.io. Read more information on sharing protocols at https://plos.org/protocols?utm_medium=editorial-email&utm_source=authorletters&utm_campaign=protocols.

We look forward to receiving your revised manuscript.

Kind regards,

Tobias Isaac Baskin

Academic Editor

PLOS One

Response: Thanks for the opportunity to refine our work and resubmit it for your consideration, the following items have been addressed when submitting the revised manuscript:

• A letter that responds to each point raised by the academic editor and reviewer(s) as a separate file labeled 'Response to Reviewers' has been uploaded

• A 'Revised Manuscript with Track Changes'. has been uploaded

• An unmarked version of the revised paper without tracked changes labeled 'Manuscript' has been uploaded

• An updated Funding Statement and Competing Interests Statement have been included in cover letter.

• Figure file has been updated

• The completed Authorship Change Request form has been uploaded as a submission file labeled “Other.” In addition, the signed Authorship Change Request form, including e-signatures from all authors, is attached under the file type “Other” As well.

Journal Requirements:

When submitting your revision, we need you to address these additional requirements.

1.Please ensure that your manuscript meets PLOS ONE's style requirements, including those for file naming. The PLOS ONE style templates can be found at

https://journals.plos.org/plosone/s/file?id=wjVg/PLOSOne_formatting_sample_main_body.pdf and

https://journals.plos.org/plosone/s/file?id=ba62/PLOSOne_formatting_sample_title_authors_affiliations.pdf

Response: We have reviewed the PLOS ONE style templates provided in the links and made the necessary adjustments to our manuscript, including body formatting and file naming conventions.

2. Please note that funding information should not appear in any section or other areas of your manuscript. We will only publish funding information present in the Funding Statement section of the online submission form. Please remove any funding-related text from the manuscript.

Response: The funding statement has been removed from the manuscript. Updated Financial disclosure statement has been submitted online.

Funding for this research was provided by Bayer CropScience LLC.

The funder provided support in the form of salaries for authors XG, NS, CJ, JG, EA, CD, CP, SS, YW, but did not have any additional role in the study design, data collection and analysis, decision to publish, or preparation of the manuscript. The specific roles of these authors are articulated in the ‘author contributions’ section.

3. Thank you for stating the following in the Competing Interests section:

“The authors have declared that no competing interests exist.”

We note that one or more of the authors are employed by a commercial company: Bayer CropScience LLC

1. Please provide an amended Funding Statement declaring this commercial affiliation, as well as a statement regarding the Role of Funders in your study. If the funding organization did not play a role in the study design, data collection and analysis, decision to publish, or preparation of the manuscript and only provided financial support in the form of authors' salaries and/or research materials, please review your statements relating to the author contributions, and ensure you have specifically and accurately indicated the role(s) that these authors had in your study. You can update author roles in the Author Contributions section of the online submission form.

Please also include the following statement within your amended Funding Statement.

“The funder provided support in the form of salaries for authors [insert relevant initials], but did not have any additional role in the study design, data collection and analysis, decision to publish, or preparation of the manuscript. The specific roles of these authors are articulated in the ‘author contributions’ section.”

If your commercial affiliation did play a role in your study, please state and explain this role within your updated Funding Statement.

Response: The funding statement has been removed from the manuscript. Updated Financial disclosure statement has been submitted online.

Funding for this research was provided by Bayer CropScience LLC.

The funder provided support in the form of salaries for authors XG, NS, CJ, JG, EA, CD, CP, SS, YW, but did not have any additional role in the study design, data collection and analysis, decision to publish, or preparation of the manuscript. The specific roles of these authors are articulated in the ‘author contributions’ section.

2. Please also provide an updated Competing Interests Statement declaring this commercial affiliation along with any other relevant declarations relating to employment, consultancy, patents, products in development, or marketed products, etc.

Within your Competing Interests Statement, please confirm that this commercial affiliation does not alter your adherence to all PLOS ONE policies on sharing data and materials by including the following statement: "This does not alter our adherence to PLOS ONE policies on sharing data and materials.” (as detailed online in our guide for authors http://journals.plos.org/plosone/s/competing-interests) . If this adherence statement is not accurate and there are restrictions on sharing of data and/or materials, please state these. Please note that we cannot proceed with consideration of your article until this information has been declared.

Please include both an updated Funding Statement and Competing Interests Statement in your cover letter. We will change the online submission form on your behalf.

Response: An updated Funding Statement and Competing Interests Statement have been included in cover letter. updated Competing Interests Statement are as below.

Updated Competing Interests Statement

A patent application has been filed for this work. This does not alter our adherence to PLOS ONE policies on sharing data and materials.

4. Please include your full ethics statement in the ‘Methods’ section of your manuscript file. In your statement, please include the full name of the IRB or ethics committee who approved or waived your study, as well as whether or not you obtained informed written or verbal consent. If consent was waived for your study, please include this information in your statement as well.

Response: The research applied in this study involved only plant species and did not include human subjects or animals, ethics approval was not applicable. We have added the ethics statement to the beginning of the Methods section of the manuscript, consistent with the information provided in the submission form. Specifically, we added the following statement at line 120: “Plant materials consisted of conventional crop varieties grown in experimental plots that were collocated with USDA-permitted materials under Authorization No. AUTH-0000233243.”

5. We note that Figure 1 in your submission contain copyrighted images. All PLOS content is published under the Creative Commons Attribution License (CC BY 4.0), which means that the manuscript, images, and Supporting Information files will be freely available online, and any third party is permitted to access, download, copy, distribute, and use these materials in any way, even commercially, with proper attribution. For more information, see our copyright guidelines: http://journals.plos.org/plosone/s/licenses-and-copyright.

We require you to either (1) present written permission from the copyright holder to publish these figures specifically under the CC BY 4.0 license, or (2) remove the figures from your submission:

1. You may seek permission from the original copyright holder of Figure 1 to publish the content specifically under the CC BY 4.0 license.

We recommend that you contact the original copyright holder with the Content Permission Form (http://journals.plos.org/plosone/s/file?id=7c09/content-permission-form.pdf) and the following text:

“I request permission for the open-access journal PLOS ONE to publish XXX under the Creative Commons Attribution License (CCAL) CC BY 4.0 (http://creativecommons.org/licenses/by/4.0/). Please be aware that this license allows unrestricted use and distribution, even commercially, by third parties. Please reply and provide explicit written permission to publish XXX under a CC BY license and complete the attached form.”

Please upload the completed Content Permission Form or other proof of granted permissions as an "Other" file with your submission.

In the figure caption of the copyrighted figure, please include the following text: “Reprinted from [ref] under a CC BY license, with permission from [name of publisher], original copyright [original copyright year].”

2. If you are unable to obtain permission from the original copyright holder to publish these figures under the CC BY 4.0 license or if the copyright holder’s requirements are incompatible with the CC BY 4.0 license, please either i) remove the figure or ii) supply a replacement figure that complies with the CC BY 4.0 license. Please check copyright information on all replacement figures and update the figure caption with source information. If applicable, please specify in the figure caption text when a figure is similar but not identical to the original image and is therefore for illustrative purposes only.

Response: Figure 1 in the revised manuscript is an original author-created illustration of our workflow that can be licensed under CC BY 4.0. The submitted figure was prepared by the authors using standard drawing tools and was not copied or traced from any AI-generated image.

6. We notice that your supplementary tables are included in the manuscript file. Please remove them and upload them with the file type 'Supporting Information'. Please ensure that each Supporting Information file has a legend listed in the manuscript after the references list.

Response: Supplementary tables are removed and uploaded with the file type 'Supporting Information'. The supporting information files have legend listed in the manuscript after the references list.

7. If the reviewer comments include a recommendation to cite specific previously published works, please review and evaluate these publications to determine whether they are relevant and should be cited. There is no requirement to cite these works unless the editor has indicated otherwise.

Response: We have carefully evaluated the recommended publications that address the spatial distributions of gibberellins in growing organs and the impact of environmental factors on gibberellins levels. Reference 7 has been previously cited for the spatial distribution of gibberellins in our introduction. We agreed with the reviewer that relevant references provide important context and insights that complement our study. Therefore, we incorporated additional 4 relevant citations into the introduction to enhance the literature review and address the reviewer's concerns.

Reference 7

Rizza A, Jones AM. The makings of a gradient: spatiotemporal distribution of gibberellins in plant development. Current Opinion in Plant Biology, 2019, 47:9-15.

Reference 4

Ubeda-Tomás S, Federici F, Casimiro I, Beemster GTS, Bhalerao R, Swarup R, et al. Gibberellin Signaling in the Endodermis Controls Root Meristem Size. Current Biology. 2009;19(14):1194-9.

Reference 8

Nelissen H, Rymen B, Jikumaru Y, Demuynck K, Van Lijsebettens M, Kamiya Y, et al. A local maximum in gibberellin levels regulates maize leaf growth by spatial control of cell division. Current Biology. 2012;22(13):1183-7.

Reference 14

Rizza A, Walia A, Lanquar V, Frommer WB, Alexander M.Jones. In vivo gibberellin gradients visualized in rapidly elongating tissues, Nature Plants 2017, 3: 803–813

Reference 15

Binenbaum J, Weinstain R, Shani E. Gibberellin Localization and Transport in Plants, Trends in Plant Science, 2018, 23: 410-421

Additional Editor Comments:

Thank you for submitting your work to our journal. Your work has been read by two reviewers who both found substantial flaws. Reviewer 1 is a statistician. This reviewers points out places with missing or confusing information with respect to experimental design and interpretation. These points need addressing before a reader can have confidence in your approach. Reviewer 2 is a biologist; they point out issues with respect to plant development. Gibberellin varies within an organ and over time as the organ develops. In the best case, this axis would be sampled but at the very least it needs to be addressed.

I have a three additional points. First, please spell out gibberellin when used to refer to the hormone in general. Gibberellin is a lovely word, echoing as it does the word gibberish, inspired by the foolish seedling disease that led to the hormone's discovery. Scientists have come to think that they are supposed to abbreviate words of their discourse but readers find this practice unhelpful. We prefer to read words over symbols. Familiarity is irrelevant.

The other day, I saw this with respect to current culture war on-going in the U.S.A. “If you oppose DEI you should have to use the full phase instead of the letters. Be brave and say, I oppose diversity, equity, and inclusion. Making it an acronym transforms it into a thought-terminating cliche.” While thought “terminating” is perhaps an exaggeration, thought “suppressing” is correct. As scientists we are urged to communicate better; the first and easiest thing we can do toward that end is to write with words.

In the case of gibberellin, a compromise can be to use GA1 (etc.) for the specific molecule (although I encourage you to bite this bullet and use gibberellin-1).

Response: Thank you for your detailed feedback and for the insights provided by the reviewers. We appreciate the opportunity to address the substantial concerns raised regarding our manuscript.

Addressing Reviewer 1's Comments: We acknowledge the points raised by Reviewer 1 regarding the experimental design and result interpretation. We have reviewed the sections highlighted and provided additional clarity and detail to ensure that our methodology is transparent and comprehensible. This information was included in Supplementary 1 (S1 table) and a more thorough explanation of our statistical analyses and their relevance to our findings in results and discussion.

Addressing Reviewer 2's Comments: We recognize the importance of addressing the variability of gibberellin levels within an organ and over time during development. We have incorporated additional relevant citations (4,8,14,15) into the introduction to enhance the literature review and address the reviewer's concerns, highlighting how it impacts our results and interpretations.

Reference 4

Ubeda-Tomás S, Federici F, Casimiro I, Beemster GTS, Bhalerao R, Swarup R, et al. Gibberellin Signaling in the Endodermis Controls Root Meristem Size. Current Biology. 2009;19(14):1194-9.

Reference 8

Nelissen H, Rymen B, Jikumaru Y, Demuynck K, Van Lijsebettens M, Kamiya Y, et al. A local maximum in gibberellin levels regulates maize leaf growth by spatial control of cell division. Current Biology. 2012;22(13):1183-7.

Reference 14

Rizza A, Walia A, Lanquar V, Frommer WB, Alexander M.Jones. In vivo gibberellin gradients visualized in rapidly elongating tissues, Nature Plants 2017, 3: 803–813

Reference 15

Binenbaum J, Weinstain R, Shani E. Gibberellin Localization and Transport in Plants, Trends in Plant Science, 2018, 23: 410-421

Spelling Out Gibberellin: We appreciate your perspective on the use of terminology in scientific writing. In response to your comments, we will spell out "gibberellin" in full when referring to the hormone in general throughout the manuscript. We will use the specific abbreviations (e.g., GA1) only when referring to particular molecules, as suggested. We understand the importance of clear communication and will strive to enhance the readability of our work.

Second, PLoS One publishes papers that are valid with no reflection on whether they are important or even interesting. Because most journals want the work to be more than valid, writers tend to pitch the importance of their story. But this gets in the way here. What is needed is simply to introduce the reader to the problem, say what you did to solve it, and what you think it means. There is no need to stretch beyond the straightforward.

Response: we have revised the manuscript to ensure that it presents the research in a straightforward manner. Specifically, we have:

1. Introduce the Problem Clearly: We succinctly outlined the problem we are addressing without overstating its significance.

2. Describe Our Approach: We articulated the methods and approaches we employed to address the problem, ensuring that the description is concise and focused.

3. Discuss the Implications: We presented our findings and their implications in a straightforward manner, avoiding any embellishments or overstated claims.

By adopting this more direct approach, we enhanced the clarity and accessibility of our manuscript, ensuring it meets the expectations of your journal.

Third, please use separate results and discussion sections. The combination is useful for a short paper, for example with one figure. But your paper is full-size. Present the results; then discuss. This format has persisted for years because it is helpful.

[Note: HTML markup is below. Please do not edit.]

Response: Thanks for the great suggestion. Results and Discussion were separated and organized into two sections in paper.

Reviewers' comments:

Reviewer's Responses to Questions

Comments to the Author

1. Is the manuscript technically sound, and do the data support the conclusions?

The manuscript must describe a technically sound piece of scientific research with data that supports the conclusions. Experiments must have been conducted rigorously, with appropriate controls, replication, and sample sizes. The conclusions must be drawn appropriately based on the data presented.

Reviewer #1: Partly

Reviewer #2: Partly

________________________________________

2. Has the statistical analysis been performed appropriately and rigorously?

Reviewer #1: No

Reviewer #2: Yes

________________________________________

3. Have the authors made all data underlying the findings in their manuscript fully available?

The PLOS Data policy requires authors to make all data underlying the findings described in their manuscript fully available without restriction, with rare exception (please refer to the Data Availability Statement in the manuscript PDF file). The data should be provided as part of the manuscript or its supporting information, or deposited to a public repository. For example, in addition to summary statistics, the data points behind means, medians and variance measures should be available. If there are restrictions on publicly sharing data—e.g. participant privacy or use of data from a third party—those must be specified.

Reviewer #1: Yes

Reviewer #2: Yes

________________________________________

4. Is the manuscript presented in an intelligible fashion and written in standard English?

PLOS ONE does not copyedit accepted manuscripts, so the language in submitted articles must be clear, correct, and unambiguous. Any typographical or grammatical errors should be corrected at revision, so please note any specific errors here.

Reviewer #1: Yes

Reviewer #2: Yes

________________________________________

5. Review Comments to the Author

Please use the space provided to explain your answers to the questions above. You may also include additional comments for the author, including concerns about dual publication, research ethics, or publication ethics. (Please upload your review as an attachment if it exceeds 20,000 characters)

Reviewer #1: I focus my comments mainly on the statistical aspects of the manuscript. The study benefits from a good spread of geographical locations, and it is sensible in aiming to use a classical experimental design and linear statistical model. However, important details on the design are missing, and too much is wrapped up in the sentence “Each site was planted in a randomized complete block design with four replicated blocks. Normal agronomic practices were followed for each site.” It is unclear what the “unit” of planting was. What, physically, were the four blocks, and how were they established? For the hybrids listed at each particular site, does each hybrid (at that site) appear in each block? What was the plot size and layout, was there any obvious spatial heterogeneity? After growing, how was sampling carried out? How many plants per plot were sampled, and were samples from single plants or pooled? These points matter for interpreting both the structure of the model and the meaning of the variance components.

Response: We have added details to the method section in paper and more details “Maize field trials” section in Table S1. Each site was planted in a randomized complete block design with four replicated blocks. Hybrids listed in Table 1 were included in each of the four replicated blocks where they were planted, in addition to other hybrids not associated with this study. Within a block, each hybrid was planted in an approximately 6 m × 6 m plot. Each site received a unique randomization scheme. Blocks at each site were arranged to limit variability in environmental conditions such as soil type, slope, and previous cropping history. Normal agronomic practices were followed for each site. Trial maintenance (fertilization, pesticide use, tillage practices, irrigation, etc.) varied across sites, but all maintenance activities were applied uniformly within a site. Maize samples were collected throughout the growing season and included the following tissues: leaf, root, stalk, pollen, forage, and grain. Tissues collected from each plot were pooled into a single sample. Sampling details and growth stage timing can be found in Table S1.

The authors note (Table 1) the unbalanced design with respect to hybrids (some appear once, some at several sites, none of them appears at all six). There is no obvious or systematic overlap structure, and I assume this was driven by logistical constraints rather than design. Nevertheless, it warrants careful discussion because it strongly affects what can be inferred: in particular, the genotype (hybrid) × environment (site) interaction will be poorly identified. With such an incomplete hybrid–site crossing, the data contain (possibly very) limited information to identify hybrid main effects, site effects, and their interaction.

In the linear model, a log transform is used for the response variable. Some explanation of the motivation would be helpful. There is clearly enormous variation (Table 4), spanning four orders of magnitude for some tissue types, which perhaps drives that choice. But is that range biologically plausible, or does it raise concerns about quantification? The fact that some observations fall below detection limits and need to be replaced by a small non-zero value so that the log transformation is defined further raises questions about sensitivity to the choice of imputed value. A simple sensitivity analysis (replacing the imputed value with something smaller and something larger and checking that the conclusions are robust) would be valuable here.

Response: Thanks reviewer for the great question. The goal of this investigation is to estimate the natural variation of gibberellins for variety of hybrids in multiple sites. This imbalance would certainly limit the quantitative estimation of each component and the significance of testing but is not expected to affect a qualitative assessment of e.g. comparing contributions of hybrid and site, or evidence of interactions. Despite the unbalanced hybrid-site design resulting in fewer degrees of freedom for the interaction, the statistical test of the interaction is a type III test and not supposed to be affected by the main effects. In terms of log-transformation, both distributions of the observed response and the residual plots support the choice of the transformation and no evidence of heterogeneity.For the observed response < LOQ (limit of quantitation), replacing with half of LOQ is standard procedure in regulatory. Those replacements are not imputations because the observed responses are in fact between 0 and LOQ not missing, thus in the lower end of the ranges in Figure 4 and Figure 5, and would have little effect on the mean (of four replicates often with only one replacement in one mean) and the correlation in the figure. Some clarification was added to the revision.

For the model itself, eqn. (1) seems broadly reasonable, but because the experimental design is not clearly described, it is difficult to understand exactly what the indices run over and how many parameters the model entails. For example, an interaction term cannot be fitted for a site–hybrid combination for which no data exist (e.g. Jefferson County × Stone 6290). The authors state that they use an “allrandomeffect” model, which is perhaps meant to address this, but then immediately switch to a fixedeffects model (“further investigation was made for the mean changes of individual hybrid across sites… a fixedeffect model was assumed and least-square means were estimated”). It is unclear what fixedeffects model is meant here, i.e., which terms are treated as fixed and which as random. The following paragraph (around line 202) switches back to discussion of random effects. This is confusing, because the distinction between fixed and random effects is not simply a matter of computational convenience but relates to the assumptions and interpretation of the model. The hybrids in question are specific, named commercial hybrids, not a random sample from a hypothetical population of hybrids, so treating them as random requires justification. Even if the SAS procedures return variancecomponent estimates, the standard likelihood/chi-squaredbased testing assumptions (which I believe the SAS procedures will use) are almost certainly unreliable in designs as unbalanced as the present one - and in view of that, the resulting pvalues and variance partitions should be interpreted with caution/caveats. Further, I did not understand the description of the statistical procedures well enough to know whether the authors were alert to, and accounted for, multiple testing.

Response: Estimation of the hybrid-site interaction is possible only when more than one reference presents at more than one site. Therefore, Figure 4 was included to visualize the interaction connecting the hybrid means over available sites. In the revised manuscript, three SAS procedures were explicitly explained under the same statistical model assuming no fixed effect, each procedure has some advantages over others which is especially important for the unbalanced data. For example, PROC MIXED is better for an unbiased estimate of the variance component due to the restricted maximum likelihood applied, and PROC GLIMMX can provide more reliable significance test of each component because it is a true likelihood based method and applied the likelihood ratio using probability from the mixed chi-squared distributions (because of the zero boundary of the variance component). The hybrid-site means, however, were obtained from PROC GLM which can provide mean prediction for each level of a random factor. The multiple testing was not considered because the relative comparison (e.g. hybrid versus site) is more important than the individual component estimate. However, in the revised manuscript, the separation was made between the significance at the 5% level, 1% level and 0.1% level..

The discussion how positive correlations between GA₁ and GA₄ concentrations could be attributed to regulatory pathways doesn't seem implausible, but isn't a simpler explanation just that both GA₁ and GA₄ are higher when plants are growing more vigorously, i.e. that overall physiological “health” could be a confounder? And indeed isn't "health" itself substantially environmentally driven by "quality of growing conditions"? (This is consistent with the authors finding that the site effect was very significant; this being field data, the authors could presumably not control for weather variation between sites.) The manuscript moves quickly from correlation to, for example, speculation about shared transcriptional networks which whilst possible, seems quite a leap beyond what the data alone suggest.

Response: We thank the reviewer for this insightful comment. We agree that the observed positive GA₁–GA₄ correlations could reflect overall plant vigor/physiological status and environmentally driven differences in growing conditions among sites (consistent with the significant site effects we observed). Although some growth and environmental information was recorded during the field trials, these measures were not sufficiently detailed to support a robust covariate adjustment, so we cannot rule out confounding by vigor or weather. In addition, the GA₁–GA₄ association was also observed when examining residual variation after accounting for hybrid, site, and hybrid × site effects, suggesting that the correlation is less likely to be the result of those experimental factors and strongly in favor of biological connection. Nevertheless, mechanistic interpretations (e.g., shared regulatory pathways or transcriptional networks) remain hypotheses requiring targeted follow-up. We revised the Discussion accordingly to emphasize the correlational nature of these results and to temper mechanistic language.

Some suggested actions:

- clarify the experimental design (describing clearly the "plot"s, block structure, what appears in what blocks, etc)

Response: We have added details to the “Maize field trials” section in Table S1.

Each site was planted in a randomized complete block design with four replicated blocks. Hybrids listed in Table 1 were included in each of the four replicated blocks where they were planted, in addition to other hybrids not associated with this study. Within a block, each hybrid was planted in an approximately 6 m × 6 m plot. Each site received a unique randomization scheme. Blocks at each site were arranged to limit variability in environmental conditions such as soil type, slope, and previous cropping history. Normal agronomic practices were followed for each site. Trial maintenance (fertilization, pesticide use, tillage practices, irrigation, etc.) varied across sites, but all maintenance activities were applied uniformly within a site. Maize samples were collected throughout the growing season and included the following tissues: leaf, root, stalk, pollen, forage, and grain. Tissues collected from each plot were pooled into a single sample. Sampling details and growth stage timing can be found in Table S1.

- describe fully the sampling (how many plants, which were selected for measurement, whether there was pooling, etc)

Response: We have added details to the “Maize field trials” section in Table S1.

Each site was planted in a randomized complete block design with four replicated blocks. Hybrids listed in Table 1 were included in each of the four replicated blocks where they were planted, in addition to other hybrids not associated with this study. Within a block, each hybrid was planted in an approximately 6 m × 6 m plot. Each site received a unique randomization scheme. Blocks at each site were arranged to limit variability in environmental conditions such as soil type, slope, and previous cropping history. Normal agronomic practices were followed for each site. Trial maintenance (fertilization, pesticide use, tillage practices, irrigation, etc.) varied across sites, but all maintenance activities were applied uniformly within a site. Maize samples were collected throughout the growing season and included the following tissues: leaf, root, stalk, pollen, forage, and grain. Tissues collected from each plot were pooled into a single sample. Sampling details and growth stage timing can be found in Table S1.

- explain the consequences of the design imbalance (related in particular to the hybrid x site interaction)

Response: The goal of this investigation is to estimate the natural variation and to compare contributions of hybrid (i.e. genetic) and site (or environment) and their interactions. Unbalanced design is due to nature of this purpose, e.g. not all references adaptable in all sites (e.g. limitation of maturity) and the limited capacity of each site. This imbalance would certainly limit the quantitative estimation of each component and the significance of the testing but is not expected to affect a qualitative assessment of e.g. comparing contributions of hybrid and site, or evidence of interactions. Despite the unbalanced hybrid-site design resulting in fewer degrees of freedom for the interaction, the statistical test of the interaction is a type III test and not supposed to be affected by the main effects. The distributions of the observed response and the residual plots support the choice of the log-normal transformation and there is no evidence of heterogeneity. We revised the paper to reflect it.

- [to help with the preceding bullet:] report CIs, not just significance test results, for the variance components

Response: The estimate of the variance component is in the log scale and the comparison can only be made on a relative base rather than the scale of the observed response, thus CIs would have no direct practical use.

- clarify the statistical modelling: at different stages, what is treated as random and what is treated as fixed? (with explanation for these choices)

Response: The original version of the manuscript called the fixed effects in visualizing the interaction, which caused some confusion for the change of model assumption. In the revised manuscript, the random effects were assumed in all analyses, but a different SAS procedure PROC GLM was applied to obtain the mean prediction for visualizing the interaction. This was explained in the revision.

- clarify whether there were distinct multiple hypothesis tests, and if so whether multiple testing was accounted for in reporting the significance of the results

Response: Multiple comparisons were not considered, but significance was indicated at the 5% level,1% level, and 0.1%.

-discuss/justify the log transformation more, including a discussion of whether the extreme range of experimental measurements is biologically reasonable.

Response: The log transformation was based on both distributions of the observed response and the residual after fitting the statistical model. For example, the following are the scattered plots of the observed response GA4 in Grain (there are 4 samples for each hybrid at each site) before and after the log transformation, which clearly indicate that some extreme values before the transformation are due to the scale (log scale or the scale of the observation) and in fact comparable with other responses after the transformation.

- if there is value imputation (for effective zeros in the experimental data) perform a sensitivity analysis that the choice of imputed value isn't too consequential to the conclusions

Response: The response <LLOQ consists of a small fraction of the available data and almost none of four field replicates of the same hybrid at the same site are all <LLOQ (as shown in the above plot). It is therefore expected that their effects on hybrid and site are limited due to the mean of four replicates. Furthermore, In the mean plots of Fig. 3 and the correlation in Fig. 4, those low responses are normally on the lower end which are not expected to determine the general trend (Fig. 4) and the comparisons of the variance components (Fig. 2 and Fig. 3).

- temper the interpretation/claims in places, e.g. related to the GA1 and GA4 concentrations. The last sentence of the abstract also feels a bit overreaching in relation to the results mentioned before it in the abstract

Response: Here’s a revised version of the sentence that tempers the interpretation while still conveying the essential findings: "Our findings provide preliminary insights into the interplay between genetics, environment, and hormonal regulation in maize, highlighting potential targets for breeding programs that could enhance agronomic traits, including yield." This revision softens the claims regarding the implications of GA1 and GA4 concentrations and avoids overreaching conclusions in relation to the results discussed earlier in the abstract.

Reviewer #2: The paper deals with large analysis of the two most bioactive GAs GA1 and GA4 in 6 different tissues of maize plants of different hybrid varieties and grown on a number of contrasting field sites.

The analysis is performed mainly by performing a correlation analysis, of which the findings appear to be that GA1 and 4 broadly covary, that there are large differences in GA levels in different tissues and that the environment has a stronger effect than Genotype, although both are significant.

I’m not enough of an expert to evaluate to what extent the analytical method is state of the art and what the quality of the measurements is.

Other than that the manuscript fails to convince.

In the introduction literature is reviewed about previous analyses of GA, but non of the work on spatial distributions of GA in growing organs or the impact of environmental factors on GA levels.

Response: We thank the reviewer for this helpful comment. We agree that prior studies on within-organ spatial distributions (gradients) of gibberellins and on specific environmental drivers are important for interpreting GA biology. Our study does not attempt to resolve sub-tissue spatial gradients or to isolate individual environmental variables; rather, we quantify the two most bioactive gibberellins (GA1 and GA4) at the tissue level across maize tissues, hybrids, and contrasting field sites (with “site” serving as an overall proxy for environmental effects). To provide appropriate context on the importance of GA gradients in development, we cite Ref. 7 (Rizza and Jones, 2019) and additional 4 references in the Introduction. We also note higher-resolution spatial mapping and targeted environment-factor experiments as important directions for future consideration.

Reference 4

Ubeda-Tomás S, Federici F, Casimiro I, Beemster GTS, Bhalerao R, Swarup R, et al. Gibberellin Signaling in the Endodermis Controls Root Meristem Size. Current Biology. 2009;19(14):1194-9.

Reference 8

Nelissen H, Rymen B, Jikumaru Y, Demuynck K, Van Lijsebettens M, Kamiya Y, et al. A local maximum in gibberellin levels regulates maize leaf growth by spatial control of cell division. Current Biology. 2012;22(13):1183-7.

Reference 14

Rizza A, Walia A, Lanquar V, Frommer WB, Alexander M.Jones. In vivo gibberellin gradients visualized in rapidly elongating tissues, Nature Plants 2017, 3: 803–813

Reference 15

Binenbaum J, Weinstain R, Shani E. Gibberellin Localization and Transport in Plants, Trends in Plant Science, 2018, 23: 410-421

A lot of space is taken to explain the technical details of the method, even the whole first section of the results is devoted to that. Nevertheless it is not clear what exactly was sampled. Which (parts of) leaves, roots, silks etc did they harvest, what was the developmental stage of the plant, harvest time of day, etc. All of these are expected have a significant effect on the measurements.

Response: We have added details to the “Maize field trials” section in Table S1.

Each site was planted in a randomized complete block design with four replicated blocks. Hybrids listed in Table 1 were included in each of the four replicated blocks where they were planted, in addition to other hybrids not associated with this study. Within a block, each hybrid was planted in an approximately 6 m × 6 m plot. Each site received a unique randomization scheme. Blocks at each site were arranged to limit variability in environmental conditions such as soil type, slope, and previous cropping history. Normal agronomic practices were followed for each site. Trial maintenance (fertilization, pesticide use, tillage practices, irrigation, etc.) varied across sites, but all maintenance activities were applied uniformly within a site. Maize samples were collected throughout the growing season and included the following tissues: leaf, root, stalk, pollen, forage, and grain. Tissues collected from each plot were pooled into a single sample. Sampling details and growth stage timing can be found in Table S1.

The results basically just deal with the observed variations, but we learn nothing about the biological meaning of these observations, so in the end there seems very little new insight.

In the discussion the authors suggest that we have learned a lot on the regulation of GA regulation, but I fail to see how they assessed regulatory mechanisms or effects.

Curiously they state to be surprised to see that there is a significant covariation between GA1 and GA4, given the (according to them) distinct synthetic pathways. This ignores the topology of the biosynthetic pathway: many of the enzymes are involved with the synthesis of both bioactive GAs. The upstream pathway up to GA12 is shared and only GA13ox transforms GA12 a precursor of GA4 to GA97 a precursor of GA1. All enzymes working GA12 and GA97 are identical creating two paralel branches regulated by the same enzymes.

In this light it is quite logical there is a covariation.

Response: We thank the reviewer for their insightful comment regarding the covariation of GA1 and GA4. The reviewer is correct that the biosynthetic pathways for GA1 and GA4 share a common upstream pathway and that the subsequent parallel branches utilize the same families of enzymes (e.g., GA20ox, GA3ox). From this perspective, a degree of co-regulation is indeed a logical expectation.

Our intention was not to express surprise at the fundamental biochemistry, but rather to highlight the novelty of observing this relationship so robustly in maize across diverse genetic and environmental backgrounds in the field. The maize genome contains multiple paralogs for these key enzymes, and it is not currently known whether these different enzyme isoforms exhibit preferences for substrates in the C13-hydroxylated (leading to GA1) versus the non-C13-hydroxylated (leading to GA4) pathways, or if their expression is differentially regulated by tissue type or environmental stimuli.

Given this potential for divergent regulation, observing a consistent positive correlation is a non-trivial finding. It provides the first empirical, field-level evidence that the biosynthesis of these two major bioactive GAs is, in practice, highly coordinated in maize.

To address the reviewer's concern and improve clarity, we have revised the manuscript to reframe this discussion. We have modified the text to explicitly acknowledge the shared enzymatic steps as the likely basis for the correlation, while also discussing the complexity introduced by multiple enzyme isoforms and how our data helps to resolve the question of their integrated output in an agricultural context.

So altogether this analysis and the manuscript as a whole to my mind is not yet suitable for publication.

Response: Below, we outlined our responses to each of the points raised:

1. Analysis and Findings: We have enhanced the discussion in the manuscript to clarify the biological significance of these findings and how they contribute to our understanding of gibberellin regulation in maize.

2. Literature Review: We have added discussion on spatial distributions of GA in growing organs and the impact of environmental factors. We have revised the introduction to incorporate relevant literature that addresses these aspects, thereby providing a more comprehensive context for our study.

3. Technical Details and Sampling: We have included detailed information about which parts of the maize plants were harvested (e.g., specific leaf sections, roots, silks), the developmental stages of the plants at the time of sampling, and the time of day when samples were collected in table S1. In supporting information, table 1 listed sample type and collection information (sampling details, collection growth stage, and growth stage) which shows what exactly was sampled.

4. Biological Meaning of Observations: We have revised the results section to not only present the observed variations but also to discuss their biological implications. This helped to provide insights into the underlying mechanisms of gibberellin regulation and its relevance to maize development.

5. Regulatory Mechanisms: We have revised the discussion to better articulate how our findings relate to the regulation of GA levels and to clarify any misconceptions regarding the distinct synthetic pathways of GA1 and GA4. We have incorporated the reviewer’s insights about the shared biosynthetic pathways and the implications for covariation.

We have addressed the reviewer’s concerns thoroughly to strengthen our manuscript. With these revisions, we have enhanced the clarity and impact of our findings, making the manuscript more suitable for publication.

________________________________________

6. PLOS authors have the option to publish the peer review history of their article (what does this mean?). If published, this will include your full peer review and any attached files.

If you choose “no”, your identity will remain anonymous but your review may still be made public.

Do you want your identity to be public for this peer review? For information about this choice, including consent withdrawal, please see our Privacy Policy.

Reviewer #1: No

Reviewer #2: Yes: Gerrit Beemster

________________________________________

[NOTE: If reviewer comments were submitted as an attachment file, they will be attached to this email and accessible via the submission site. Please log into your account, locate the manuscript record, and check for the action link "View Attachments". If this link does not appear, there are no attachment files.]

To ensure your figures meet our technical requirements, please review our figure guidelines: https://journals.plos.org/plosone/s/figures

You may also use PLOS’s free figure tool, NAAS, to help you prepare publication quality figures: https://journals.plos.org/plosone/s/figures#loc-tools-for-figure-preparation.

NAAS will assess whether your figures meet our technical requirements by comparing each figure against our figure specifications.

________________________________________

In compliance with data protection regulations, you may request that we remove your personal registration details at any time. (Remove my information/details). Please contact the publication office if you have any questions.

Attachment Submitted filename: Response to Reviewers.docx

# Peer review: Decision Letter 1

**DOI:** 10.1371/journal.pone.0340156.r003

**Authors:** Tobias Baskin

21 Jun 2026

Dear Dr. Wang,

Thank you for submitting your manuscript to PLOS ONE. After careful consideration, we feel that it has merit but does not fully meet PLOS ONE’s publication criteria as it currently stands. Therefore, we invite you to submit a revised version of the manuscript that addresses the points raised during the review process.

Please submit your revised manuscript by Aug 05 2026 11:59PM. If you will need more time than this to complete your revisions, please reply to this message or contact the journal office at plosone@plos.org. When you're ready to submit your revision, log on to https://www.editorialmanager.com/pone/ and select the 'Submissions Needing Revision' folder to locate your manuscript file.

- A letter that responds to each point raised by the academic editor and reviewer(s). You should upload this letter as a separate file labeled 'Response to Reviewers'.
- A marked-up copy of your manuscript that highlights changes made to the original version. You should upload this as a separate file labeled 'Revised Manuscript with Track Changes'.
- An unmarked version of your revised paper without tracked changes. You should upload this as a separate file labeled 'Manuscript'.

If you would like to make changes to your financial disclosure, please include your updated statement in your cover letter. Guidelines for resubmitting your figure files are available below the reviewer comments at the end of this letter.

If applicable, we recommend that you deposit your laboratory protocols in protocols.io to enhance the reproducibility of your results. Protocols.io assigns your protocol its own identifier (DOI) so that it can be cited independently in the future. For instructions see: https://journals.plos.org/plosone/s/submission-guidelines#loc-laboratory-protocols. Additionally, PLOS ONE offers an option for publishing peer-reviewed Lab Protocol articles, which describe protocols hosted on protocols.io. Read more information on sharing protocols at https://plos.org/protocols?utm_medium=editorial-email&utm_source=authorletters&utm_campaign=protocols.

As the corresponding author, your ORCID iD is verified in the submission system and will appear in the published article. PLOS supports the use of ORCID, and we encourage all coauthors to register for an ORCID iD and use it as well. Please encourage your coauthors to verify their ORCID iD within the submission system before final acceptance, as unverified ORCID iDs will not appear in the published article. *Only* the individual author can complete the verification step; PLOS staff *cannot* verify ORCID iDs on behalf of authors.

We look forward to receiving your revised manuscript.

Kind regards,

Tobias Isaac Baskin

Academic Editor

PLOS One

Journal Requirements:

If the reviewer comments include a recommendation to cite specific previously published works, please review and evaluate these publications to determine whether they are relevant and should be cited. There is no requirement to cite these works unless the editor has indicated otherwise.

[Note: HTML markup is below. Please do not edit.]

Reviewers' comments:

Reviewer's Responses to Questions

**Comments to the Author**

Reviewer #1: (No Response)

Reviewer #3: (No Response)

**********

2. Is the manuscript technically sound, and do the data support the conclusions?

Reviewer #1: Partly

Reviewer #3: Partly

**********

3. Has the statistical analysis been performed appropriately and rigorously?

Reviewer #1: No

Reviewer #3: Yes

**********

4. Have the authors made all data underlying the findings in their manuscript fully available?

The PLOS Data policy

Reviewer #1: Yes

Reviewer #3: Yes

**********

5. Is the manuscript presented in an intelligible fashion and written in standard English?

Reviewer #1: Yes

Reviewer #3: Yes

**********

Reviewer #1: Experiments (blocking structure, randomisation, etc) are now much more clearly explained in the Methods. Though l130-132 "Maize samples were collected throughout the growing season and included the following tissues: leaf, root, stalk, pollen, forage, and grain. The same tissues collected from each plot were pooled into a single sample." remains somewhat ambiguous. How much of each tissue (and was this by weight, or volume, or something else)? From how many plants, and how were they selected (random, non-edge, convenience)? How pooled? Was pooling consistent across all sites?

Whilst the Methods section explains the experiment better, there's been no attempt made to connect it to the subsequent model, eqn (1). Does "block" (in intro) = "replicate" (in the model)? Does "plot" (in the intro) = the observational unit (in the model)? Does "pooling" (in intro) mean there is one observation per plot? The disconnect is a bit jarring, but could easily be fixed.

The issue of unbalancedness is still not well acknowledged or addressed. I appreciate that this is (now) historical data and it is out of scope to remedy the imbalance by collecting more data. Nevertheless, it is important still that the statistical inference is accurate and doesn't overstate what the data imply. l202-230 unfortunately still feel muddled with respect to the assumed statistical model. l204-206 refer to "fitting the model", but does so before fully specifying the model: which coefficients are random, and with what variance structure? The sentence "Differences may present under an unbalanced design as in this investigation, and PROC GLIMMIX is the best for significant testing due to the likelihood-based method (which is the ultimate standard for all statistical analyses), PROC MIXED supposes to be unbiased for the estimate of variance component because of the restricted maximum likelihood and the application of mixed model equations for random effects, and PROC GLM provided mean predictions of random effects by the method of moment (i.e. the expected mean square or EMS)" is very unclear to me. It does not describe the statistical assumptions or methods directly, but rather refers to SAS procedures and seems to expect the reader to reverse-engineer the implied model from that. “PROC GLM provided mean predictions of random effects by the method of moment” is especially confusing: my understanding is that PROC GLM applies to fixed effects rather than random effects, so what is meant by this statement is at best unclear. This whole section remains muddled, suggests over-reliance/over-confidence in software, and still underplays the problems caused by the unbalanced design. Likelihood-based methods are not automatically reliable in small or highly unbalanced settings, and similarly REML “unbiasedness” is not guaranteed in that same setting. It should not be for the reader or reviewer to have to unpick this.

On multiple testing, I also found the authors’ response confusing (“The multiple testing was not considered because the relative comparison (e.g. hybrid versus site)…”), and it would help to be more explicit. In Table 5, are you not reporting significance tests for variance components (at least hybrid, site, and hybrid × site, I think) separately for 10 GA–tissue combinations, i.e. at least 30 formal tests? Then later there are five separate GA1–GA4 regression/correlation tests, one for each tissue. At present these nominal p-values appear to be interpreted directly, and merely noting significance at multiple levels (5%, 1%, and 0.1%) does not really address the multiplicity issue. I am not suggesting that a formal multiple-testing correction is needed in what is partly an exploratory study, but the manuscript should at least acknowledge the number of related tests being carried out and temper the interpretation of marginal findings accordingly.

On confidence intervals, I do not understand why the authors regard the log scale as an obstacle (since variance components are naturally on that scale anyway). Confidence intervals are precisely what convey uncertainty in weakly identified settings, such as stemming from unbalanced design.

Reviewer #3: This manuscript reports a validated high-throughput LC-MS/MS workflow and uses it to quantify the bioactive gibberellins GA1 and GA4 in five maize tissues from ten commercial hybrids grown at six U.S. field sites, partitioning the variation into genetic (hybrid), environmental (site), and interaction components. The analytical method appears sound and the geographic breadth is a genuine strength, so the descriptive dataset has value as a baseline resource.

I have read the previous review round. The authors have made constructive changes — the Results and Discussion are now separated, the experimental design and sampling are described in more detail (Table S1), the GA1–GA4 correlation discussion has been appropriately tempered to acknowledge its correlational nature, and the closing claims have been softened to "preliminary insights." I appreciate these revisions.

That said, my main remaining concern is that the conclusions still reach beyond what the measured data can support, and this stems in large part from what was measured. Only the two bioactive end-products (GA1, GA4) were quantified. A steady-state pool reflects the balance of biosynthesis, 2β-hydroxylation-mediated inactivation, and conjugation, so the present data cannot attribute any observed change to a specific step. This is particularly problematic for the central claim that site (i.e., environment) is the dominant factor: the manuscript cannot say whether a site-associated change in GA1/GA4 arises from altered biosynthetic flux or from altered inactivation.

Maize is an early-13-hydroxylation (GA1-type) species, in which GA1 is inactivated to GA8 and GA4 to GA34 by GA2ox. At minimum, the authors must (i) state this limitation explicitly and (ii) remove or rephrase "biosynthesis" and "plasticity of the gibberellin pathway" language in favour of "endogenous level/abundance" wherever flux was not measured. To support any mechanistic interpretation of the site effect, quantification of the principal inactivated GAs (GA8, GA34) and key precursors (e.g., GA20, GA9), together with active-to-inactive ratios (GA1/GA8, GA4/GA34), would help indicate whether a given change reflects enhanced synthesis or reduced catabolism — although, as these are themselves steady-state pools, such ratios remain indirect proxies for the underlying flux.

Methods (L167–181). Present the LC gradient as a table including the initial hold, and confirm the steep short segments (e.g., B 26%→55% over 0.1 min) are not transcription errors. Critically, retention times for GA1, GA4, and the internal standards are not reported; please add them (and a representative MRM chromatogram near the LLOQ), as they are needed to judge whether this unusual gradient performs as intended.

Table 3 (pollen). The text states that pollen data were not reported because of its short stability window (L262–263), and pollen is correctly absent from Tables 4–5 and Figs 2–4. However, Table 3 still lists method-validation data for pollen.

Minor comments

Introduction (L49). "Gibberella fujikuroi" → the accepted name is Fusarium fujikuroi (teleomorph Gibberella fujikuroi).

Methods (L157). "until about 400 mL of aqueous phase was left" — should be 400 µL.

Methods (L152). "Nutating Mixter" → Mixer; and a missing space after "88-861-043)" before "After incubation".

Methods (L168). "AB Sxiex OS" → "AB Sciex OS"

**********

what does this mean?). If published, this will include your full peer review and any attached files.

If you choose “no”, your identity will remain anonymous but your review may still be made public.

**Do you want your identity to be public for this peer review?** For information about this choice, including consent withdrawal, please see our Privacy Policy

Reviewer #1: No

Reviewer #3: No

**********

[NOTE: If reviewer comments were submitted as an attachment file, they will be attached to this email and accessible via the submission site. Please log into your account, locate the manuscript record, and check for the action link "View Attachments". If this link does not appear, there are no attachment files.]

To ensure your figures meet our technical requirements, please review our figure guidelines: https://journals.plos.org/plosone/s/figures

You may also use PLOS’s free figure tool, NAAS, to help you prepare publication quality figures: https://journals.plos.org/plosone/s/figures#loc-tools-for-figure-preparation.

NAAS will assess whether your figures meet our technical requirements by comparing each figure against our figure specifications.

# Author comment: Author response to Decision Letter 2

**DOI:** 10.1371/journal.pone.0340156.r004

2 Aug 2026

Response to Reviewers

PONE-D-25-66749R2

Natural variation of bioactive gibberellin levels in maize tissues across North America

PLOS One

Dear Dr. Wang,

Thank you for submitting your manuscript to PLOS ONE. After careful consideration, we feel that it has merit but does not fully meet PLOS ONE’s publication criteria as it currently stands. Therefore, we invite you to submit a revised version of the manuscript that addresses the points raised during the review process.

Response

Dear Editor,

Thank you very much for your letter and for the careful evaluation of our manuscript. We sincerely appreciate your comments and the reviewers’ constructive suggestions, which have been very helpful in improving the quality and clarity of our work.

We have carefully addressed the comments from both the editor and the reviewers on a point-by-point basis. In response to these comments, we have revised the manuscript accordingly and made the necessary changes throughout the text.

In our revised submission, we have included the following files as requested:

1. Response to Reviewers – a separate letter responding to each point raised by the academic editor and reviewers.

2. Revised Manuscript with Track Changes – a marked-up version highlighting all changes made to the manuscript PONE-D-25-66749R1.

3. Manuscript – a clean, unmarked version of the revised paper without tracked changes.

Thank you again for your time and consideration. We are grateful for the opportunity to revise our manuscript for further consideration by PLOS ONE.

You have improved the paper notably. Nevertheless problems remain. Reviewer 1, a statistician, points out fairly serious inadequacies remain in your presentation. Insofar as statistical analysis constitutes a big chunk of your paper, I believe that you need to get this right. Hopefully, the problems identified by the reviewer are matters of language (what you write about the methods). But in any case, I hope you can respond satisfactorily.

Response:

Thank you for highlighting this important concern. We appreciate the editor’s emphasis on the need for a clear and rigorous presentation of the statistical analysis, particularly given its central role in the manuscript. In response, we carefully re-examined the statistical methods and substantially revised the “Variance component analysis to compare genetic and environmental variations” section to improve clarity, precision, transparency, and consistency.

We thoroughly revised the methodology section to clarify our statistical approaches, with emphasis on model assumptions, structure, and limitations. We explicitly defined our variance-partitioning framework, detailing how hybrid, site, replicate within site, and the hybrid-by-site interaction were treated across different stages of the analysis, specifically for estimating variance components, testing their significance, and examining changes in hybrid means across sites. To address the reviewer's concerns, we clarified that our use of REML (Restricted Maximum Likelihood) estimation is a well-established approach for variance component estimation in unbalanced trial designs. We also explained our rationale for not applying multiple-testing adjustments during significance testing of these components, and why confidence intervals for variance components were not provided. In addition, we removed references to SAS PROC GLM, which had been used only for intermediate data exploration, so that the manuscript now reflects only the analyses underlying the reported results. Overall, these revisions present the statistical methodology with greater transparency, stronger theoretical justification, and more appropriate caution regarding the scope and unbalanced nature of the available data.

Reviewer three (the original reviewer 2 was not available) points out several small but useful things. Their main point tho is about interpretation. Writing for PLoS One differs from many other journals because "importance" does not matter. For most journals, we write to convince the editor, the reviewers, and indeed readers, that our work is important. But importance is subjective. PLoS One removes that criteron, explicitly. Instead, what matters is validity. Writing on that head relieves us of the burden of hype. We present what we found, a straightforward account. We can of course state why we think the work matters but in doing so we should be brief and circumspect. Reviewer 3 points out where you need to town down your claims. I would add that you can probably shorten the discussion; compared to the results, it seems quite long.

Response:

Thank you for the great comments. The revised Discussion now focuses more directly on the validity of the findings, the limitations of the study design and measurements, and a more circumspect interpretation of the implications.

We revised the Discussion to align more closely with the data presented in the Results. Specifically, we removed or rephrased language that could be interpreted as overly broad or mechanistic, including statements related to “biosynthesis” and “plasticity of the gibberellin pathway,”. We also shortened the Discussion by removing repetitive statements and reducing broader contextual language.

Small points: When you cite references in the text, please put a space between the word and the bracket. Like this [14]. Not like this[14]. Also on line 74, the word 'corn' should not be capitalized.

Response:

Thank you for pointing these out. We have made these corrections throughout the manuscript, including adding a space before citation brackets where needed and changing “Corn” to “corn” on previous line 74.

Please submit your revised manuscript by Aug 05 2026 11:59PM. If you will need more time than this to complete your revisions, please reply to this message or contact the journal office at plosone@plos.org. When you're ready to submit your revision, log on to https://www.editorialmanager.com/pone/ and select the 'Submissions Needing Revision' folder to locate your manuscript file.

Please include the following items when submitting your revised manuscript:

• A letter that responds to each point raised by the academic editor and reviewer(s). You should upload this letter as a separate file labeled 'Response to Reviewers'.

• A marked-up copy of your manuscript that highlights changes made to the original version. You should upload this as a separate file labeled 'Revised Manuscript with Track Changes'.

• An unmarked version of your revised paper without tracked changes. You should upload this as a separate file labeled 'Manuscript'.

If you would like to make changes to your financial disclosure, please include your updated statement in your cover letter. Guidelines for resubmitting your figure files are available below the reviewer comments at the end of this letter.

If applicable, we recommend that you deposit your laboratory protocols in protocols.io to enhance the reproducibility of your results. Protocols.io assigns your protocol its own identifier (DOI) so that it can be cited independently in the future. For instructions see: https://journals.plos.org/plosone/s/submission-guidelines#loc-laboratory-protocols. Additionally, PLOS ONE offers an option for publishing peer-reviewed Lab Protocol articles, which describe protocols hosted on protocols.io. Read more information on sharing protocols at https://plos.org/protocols?utm_medium=editorial-email&utm_source=authorletters&utm_campaign=protocols.

As the corresponding author, your ORCID iD is verified in the submission system and will appear in the published article. PLOS supports the use of ORCID, and we encourage all coauthors to register for an ORCID iD and use it as well. Please encourage your coauthors to verify their ORCID iD within the submission system before final acceptance, as unverified ORCID iDs will not appear in the published article. Only the individual author can complete the verification step; PLOS staff cannot verify ORCID iDs on behalf of authors.

We look forward to receiving your revised manuscript.

Kind regards,

Tobias Isaac Baskin

Academic Editor

PLOS One

Journal Requirements:

If the reviewer comments include a recommendation to cite specific previously published works, please review and evaluate these publications to determine whether they are relevant and should be cited. There is no requirement to cite these works unless the editor has indicated otherwise.

[Note: HTML markup is below. Please do not edit.]

Reviewers' comments:

Reviewer's Responses to Questions

Comments to the Author

1. If the authors have adequately addressed your comments raised in a previous round of review and you feel that this manuscript is now acceptable for publication, you may indicate that here to bypass the “Comments to the Author” section, enter your conflict of interest statement in the “Confidential to Editor” section, and submit your "Accept" recommendation.

Reviewer #1: (No Response)

Reviewer #3: (No Response)

________________________________________

2. Is the manuscript technically sound, and do the data support the conclusions?

The manuscript must describe a technically sound piece of scientific research with data that supports the conclusions. Experiments must have been conducted rigorously, with appropriate controls, replication, and sample sizes. The conclusions must be drawn appropriately based on the data presented.

Reviewer #1: Partly

Reviewer #3: Partly

________________________________________

3. Has the statistical analysis been performed appropriately and rigorously?

Reviewer #1: No

Reviewer #3: Yes

________________________________________

4. Have the authors made all data underlying the findings in their manuscript fully available?

The PLOS Data policy requires authors to make all data underlying the findings described in their manuscript fully available without restriction, with rare exception (please refer to the Data Availability Statement in the manuscript PDF file). The data should be provided as part of the manuscript or its supporting information, or deposited to a public repository. For example, in addition to summary statistics, the data points behind means, medians and variance measures should be available. If there are restrictions on publicly sharing data—e.g. participant privacy or use of data from a third party—those must be specified.

Reviewer #1: Yes

Reviewer #3: Yes

________________________________________

5. Is the manuscript presented in an intelligible fashion and written in standard English?

PLOS ONE does not copyedit accepted manuscripts, so the language in submitted articles must be clear, correct, and unambiguous. Any typographical or grammatical errors should be corrected at revision, so please note any specific errors here.

Reviewer #1: Yes

Reviewer #3: Yes

________________________________________

6. Review Comments to the Author

Please use the space provided to explain your answers to the questions above. You may also include additional comments for the author, including concerns about dual publication, research ethics, or publication ethics. (Please upload your review as an attachment if it exceeds 20,000 characters)

Reviewer #1: Experiments (blocking structure, randomisation, etc) are now much more clearly explained in the Methods. Though l130-132 "Maize samples were collected throughout the growing season and included the following tissues: leaf, root, stalk, pollen, forage, and grain. The same tissues collected from each plot were pooled into a single sample." remains somewhat ambiguous. How much of each tissue (and was this by weight, or volume, or something else)? From how many plants, and how were they selected (random, non-edge, convenience)? How pooled? Was pooling consistent across all sites?

Response:

Thank you for the comments. We have revised the Methods section to clarify sufficient details regarding the amount of tissue collected, the number of plants sampled, the selection criteria, and the pooling procedure. For each plot, sampled plants were selected non-systematically but with consistent field-sampling criteria: plot ends were avoided, and plants were chosen to be competitive and representative of the plot as a whole. The selected tissue samples from each plot were combined to generate one pooled sample per plot, which served as the observational unit for downstream analysis. This pooling approach was applied consistently across all sites.

We have added detailed sampling information in S1 Table, including the pooled sample amount per plot, the number of plants sampled per plot, tissue-specific sampling details, and the collection growth stage. Specifically, the revised table now specifies the tissue-specific sampling amount used to generate one pooled sample per lot: 60 leaf sections, 8 roots, 4 stalks, 10 mL pollen, 1 forage plant, or 1 kg grain, depending on tissue type, along with the corresponding number of sampled plants and growth stages.

S1 Table. Summary of sample type and collection information

Sample Type Pooled Sample Amount per Plot Number of Plants Sampled per Plot Sampling Details Collection Growth Stage Growth Stage Description

Leaf 60 leaves 60 Approximately 15 cm of most recently collared leaf V2-V4 A plant with 2 to 4 leaf collars

Root 8 roots 8 All below-ground root tissue, thoroughly washed V2-V4 A plant with 2 to 4 leaf collars

Stalk 4 stalks 4 All above-ground stalk tissue with leaf blade, leaf sheath, ear, and tassel removed V10-V12 A plant with 10 to 12 leaf collars

Pollen 10 mL Highly variable based on environmental conditions, though typically >10 Fresh pollen with all anthers removed Pollen shed A plant that is actively shedding pollen

Forage 1 plant 1 All above-ground plant tissue R5, early dent Most kernels are dented and contain approximately 55% moisture

Grain 1 kg Minimum of 4 Shelled grain dried to ≤15.5% moisture prior to shipping and storage R6 Physiological maturity; black layer visible at base of kernels

Whilst the Methods section explains the experiment better, there's been no attempt made to connect it to the subsequent model, eqn (1). Does "block" (in intro) = "replicate" (in the model)? Does "plot" (in the intro) = the observational unit (in the model)? Does "pooling" (in intro) mean there is one observation per plot? The disconnect is a bit jarring, but could easily be fixed.

Response: Thank you for the suggestion. In the revised manuscript, we clarified that “block” in the experimental design corresponds to “replicate” in the statistical model, and that “plot” is the observational unit used in the model. We also clarified that the tissue samples collected within each plot were pooled to generate one composite sample per plot; therefore, there was one analytical observation per plot for each tissue and GA measurement. These clarifications have been added to the revised Methods section to better connect the field design, sample pooling, and statistical model.

The issue of unbalancedness is still not well acknowledged or addressed. I appreciate that this is (now) historical data and it is out of scope to remedy the imbalance by collecting more data. Nevertheless, it is important still that the statistical inference is accurate and doesn't overstate what the data imply. l202-230 unfortunately still feel muddled with respect to the assumed statistical model. l204-206 refer to "fitting the model", but does so before fully specifying the model: which coefficients are random, and with what variance structure? The sentence "Differences may present under an unbalanced design as in this investigation, and PROC GLIMMIX is the best for significant testing due to the likelihood-based method (which is the ultimate standard for all statistical analyses), PROC MIXED supposes to be unbiased for the estimate of variance component because of the restricted maximum likelihood and the application of mixed model equations for random effects, and PROC GLM provided mean predictions of random effects by the method of moment (i.e. the expected mean square or EMS)" is very unclear to me. It does not describe the statistical assumptions or methods directly, but rather refers to SAS procedures and seems to expect the reader to reverse-engineer the implied model from that. “PROC GLM provided mean predictions of random effects by the method of moment” is especially confusing: my understanding is that PROC GLM applies to fixed effects rather than random effects, so what is meant by this statement is at best unclear. This whole section remains muddled, suggests over-reliance/over-confidence in software, and still underplays the problems caused by the unbalanced design. Likelihood-based methods are not automatically reliable in small or highly unbalanced settings, and similarly REML “unbiasedness” is not guaranteed in that same setting. It should not be for the reader or reviewer to have to unpick this.

Response:

The manuscript was revised to clarify the methodological approaches. Specifically, to estimate variance components, hybrid, site, replicate within site, and the hybrid-by-site interaction were all treated as random effects, with the residual term representing error variance. Random effects were assumed to be independently and identically distributed within each classification, each associated with its own variance component. Variance was partitioned into these additive components using SAS PROC MIXED with REML (Restricted Maximum Likelihood) estimation, a well-established approach for variance component estimation in unbalanced trial designs.

The significance of variance components was then evaluated using PROC GLIMMIX with the COVTEST option via likelihood ratio tests. Because the null hypothesis places a variance component on the boundary of the parameter space at zero, the resulting test statistic follows a mixture chi-square distribution. Significance was evaluated at the 5%, 1%, and 0.1% levels without multiplicity correction.

For variance components that were found to be significant, additional analyses were performed to examine mean changes for individual hybrids across sites, thereby illustrating the extent to which hybrid effects were connected across sites and characterizing site-specific hybrid patterns.

These analyses were conducted within a linear mixed model framework using PROC MIXED. hybrid, site, and the hybrid-by-site interaction were treated as fixed effects, whereas Replicate nested within Site was modeled as a random effect. Treating hybrid and site as fixed effects produced local LS-means for characterizing hybrid-by-site patterns, while modeling nested replications as random prevented estimates from being biased by local block-level anomalies.

In addition, we removed references to SAS PROC GLM, which had been used only for intermediate data exploration, so that the manuscript now reflects only the analyses underlying the reported results.

Consequently, the approach provides valid and reliable inference for unbalanced data, though statistical power may be reduced relative to a fully balanced design.

On multiple testing, I also found the authors’ response confusing (“The multiple testing was not considered because the relative comparison (e.g. hybrid versus site)…”), and it would help to be more explicit. In Table 5, are you not reporting significance tests for variance components (at least hybrid, site, and hybrid × site, I think) separately for 10 GA–tissue combinations, i.e. at least 30 formal tests? Then later there are five separate GA1–GA4 regression/correlation tests, one for each tissue. At present these nominal p-values appear to be interpreted directly, and merely noting significance at multiple levels (5%, 1%, and 0.1%) does not really address the multiplicity issue. I am not suggesting that a formal multiple-testing correction is needed in what is partly an exploratory study, but the manuscript should at least acknowledge the number of related tests being carried out and temper the interpretation of marginal findings accordingly.

Response:

The revisions were made in the manuscript to address this concern. The primary objective of this analysis was to describe how variation in GA levels was partitioned among model components, rather than to perform confirmatory hypothesis testing. For each GA-by-tissue combination, variance components associated with hybrid, site, replicate nested within site, and the hybrid-by-site interaction were evaluated using individual likelihood ratio tests, resulting in 40 variance-component tests in total. No multiplicity adjustment was applied because these tests were used for an exploratory assessment of variance structure. Accordingly, the resulting p-values should be interpreted descriptively, together with the estimated variance components and their relative contributions to total variation. Although conducting 40 tests increases the chance of false positives, effects supported by both substantial estimated variance contributions and very small p-values provide stronger evidence of meaningful structure in the data than marginally significant effects.

On confidence intervals, I do not understand why the authors regard the log scale as an obstacle (since variance components are naturally on that scale anyway). Confidence intervals are precisely what convey uncertainty in weakly identified settings, such as stemming from unbalanced design.

Response:

Variance components were estimated by fitting an all-random model that included hybrid, site, replicate nested within site, the hybrid-by-site interaction, and residual error. This model was specified to decompose total response variation and quantify the relative contributions of environmental, design-related, and interaction sources across sites. Because the primary objective was to identify the main drivers of variability, results are presented as variance-component point estimates and their relative percentage contributions. These measures provide the most direct and practical basis for evaluating the importance of each source of variation.

Reviewer #3: This manuscript reports a validated high-throughput LC-MS/MS workflow and uses it to quantify the bioactive gibberellins GA1 and GA4 in five maize tissues from ten commercial hybrids grown at six U.S. field sites, partitioning the variation into genetic (hybrid), environmental (site), and interaction components. The analytical method appears sound and the geographic breadth is a genuine strength, so the descriptive dataset has value as a baseline resource.

I have read the previous review round. The authors have made constructive changes — the Results and Discussion are now separated, the experimental design and sampling are described in more detail (Table S1), the GA1–GA4 correlation discussion has been appropriately tempered to acknowledge its correlational nature, and the closing claims have been softened to "preliminary insights." I appreciate these revisions.

That said, my main remaining concern is that the conclusions still reach beyond what the measured data can support, and this stems in large part from what was measured. Only the two bioactive end-products (GA1, GA4) were quantified. A steady-state pool reflects the balance of biosynthesis, 2β-hydroxylation-mediated inactivation, and conjugation, so the present data cannot attribute any observed change to a specific step. This is particularly problematic for the central claim that site (i.e., environment) is the dominant factor: the manuscript cannot say whether a site-associated change in GA1/GA4 arises from altered biosynthetic flux or from altered inactivation.

Maize is an early-13-hydroxylation (GA1-type) species, in which GA1 is inactivated to GA8 and GA4 to GA34 by GA2ox. At minimum, the authors must (i) state this limitation explicitly and (ii) remove or rephrase "biosynthesis" and "plasticity of the gibberellin pathway" language in favour of "endogenous level/abundance" wherever flux was not measured. To support any mechanistic interpretation of the site effect, quantification of the principal inactivated GAs (GA8, GA34) and key precursors (e.g., GA20, GA9), together with active-to-inactive ratios (GA1/GA8, GA4/GA34), would help indicate whether a given change reflects enhanced synthesis or reduced catabolism — although, as these are themselves steady-state pools, such ratios remain indirect proxies for the underlying flux.

Response:

Thanks for the comments. We revised the Abstract, Introduction, Discussion, and Conclusion by deleting the language such as “biosynthesis,” “pathway plasticity,” and “regulation”. Biosynthetic pathway was not discussed in the paper.

Methods (L167–181). Present the LC gradient as a table including the initial hold, and confirm the steep short segments (e.g., B 26%→55% over 0.1 min) are not transcription errors. Critically, retention times for GA1, GA4, and the internal standards are not reported; please add them (and a representative MRM chromatogram near the LLOQ), as they are needed to judge whether this unusual gradient performs as intended.

Response:

Thank you for this helpful suggestion. We have revised the Methods section to present the LC gradient as Table 2, including the initial hold. We confirm that the steep, short gradient segments, such as the change from 26% to 55% B over 0.1 min, are intentional and are not transcription errors.

We have also added the retention times for GA1, GA4, and the corresponding internal standards in Table 3. In addition, we provided a representative MRM chromatogram at the LLOQ (standard 1), as Supporting Information S1 Fig. This added information should allow readers to better assess the chromatographic performance of the method and confirm that the gradient performs as intended.

S1 Fig. The representative chromatogram of LLOQ (standard 1)

Top left: TIC, Top right: XIC of GA1, Bottom left: XIC of GA1-D4 and GA4-D2, Bottom right: XIC of GA4.

Table 3 (pollen). The text states that pollen data were not reported because of its short stability window (L262–263), and pollen is correctly absent from Tables 4–5 and Figs 2–4. However, Table 3 still lists method-validation data for pollen.

Response:

Thank you for raising this point. The pollen results included in Table 3 refer only to the method-validation experiment. We validated the method for pollen along with the other maize tissues, including assessments of accuracy, precision, extraction efficiency, recovery, and benchtop stability; therefore, the validated pollen data are appropriately included in Table 3.

However, pollen had a much shorter benchtop stability window than the other tissues. Although this was manageable during method validation because only a small number of samples were processed, it was not feasible for the large field-sample set. The field study included more samples, and pollen sample preparation could not be completed within the validated stability window. For this reason, we did not consider the field pollen measurements sufficiently reliable for downstream biological interpretation and therefore excluded pollen from Tables 4–5 and Figures 2–4 for field data.

Minor comments

Introduction (L49). "Gibberella fujikuroi" → the accepted name is Fusarium fujikuroi (teleomorph Gibberella fujikuroi).

Response: “Gibberella fujikuroi" was changed to “Fusarium fujikuroi”.

Methods (L157). "until about 400 mL of aqueous phase was left" — should be 400 µL.

Response: 400 “mL” was corrected to 400 “µL”.

Methods (L152). "Nutating Mixter" → Mixer; and a missing space after "88-861-043)" before "After incubation".

Response: "Nutating Mixter" was changed to “Mixer”, and a space was added before "After incubation".

Methods (L168). "AB Sxiex OS" → "AB Sciex OS"

Response: "AB Sxiex OS" was corrected to "AB Sciex OS".

7. PLOS authors have the option to publish the peer review history of their article (what does this mean?). If published, this will include your full peer review and any attached files.

If you choose “no”, your identity will remain anonymous but your review may still be made public.

Do you want your identity to be public for this peer review? For information about this choice, including consent withdrawal, please see our Privacy Policy.

Reviewer #1: No

Reviewer #3: No

________________________________________

[NOTE: If reviewer comments were submitted as an attachment file, they will be attached to this email and accessible via the submission site. Please log into your account, locate the manuscript record, and check for the action link "View Attachments". If this link does not appear, there are no attachment files.]

To ensure your figures meet our technical requirements, please review our figure guidelines: https://journals.plos.org/plosone/s/figures

You may also use PLOS’s free figure tool, NAAS, to help you prepare publication quality figures: https://journals.plos.org/plosone/s/figures#loc-tools-for-figure-preparation.

NAAS will assess whether your figures meet our technical requirements by comparing each figure against our figure specifications.

Attachment Submitted filename: Response to Reviewers .docx

# Peer review: Decision Letter 2

**DOI:** 10.1371/journal.pone.0340156.r005

**Authors:** Tobias Baskin

24 Aug 2026

Dear Dr. Wang,

Thank you for submitting your manuscript to PLOS One. After careful consideration, the Journal and I feel that your manuscript is much improved but does not yet entirely meet PLOS One’s publication criteria. A few minor issues remain. Therefore, we invite you to submit a revised version of the manuscript that addresses the points raised during the review process.

I read the paper too and feel that it is in good shape. I have a few matters of language to call to your attention.

Line 25 (and elsewhere). I suggest you delete the word 'endogenous'. How could a hormone be 'exogenous'? I think in most places you can delete the word 'endogenous'.

Line 25. The word 'phytohomorme' was coined more than 100 years ago when scientists had little idea what was going on. Now we understand that plants have hormones. Of course they differ from those of animals, but there is no reason they would be the same. Other than the fact that they occur in plants, there is nothing that unites 'phytohormones' into a group. What is more, no one ever uses the word "zoohormones" to indicate animal hormones. There is no need. LIkewise, I can see no reason to use the term phytohormones. Writing 'hormones' is clear. No one would think you are writing about animal hormones.

Line 30, and elsewhere. The word tissue is defined as a group of cells of a similar type, such as epidermis or mesophyll. A root is not a tissue. Unfortunately, people often write loosely about throwing plant tissue in the blender, using the word 'tissue' as a synonym for 'material'. In that case, the casual wording probably doesn't matter. But here, the source of the sample is a big part of the story. It strikes me as misleading for you to call your samples 'tissues' when they are not. I realize that because your samples are not all organs, there is not an obvious alternative. However, I have gone thru your paper and suggested revisions. Please see the uploaded marked manuscript. I don't know that these are the best choices, you might well find alternatives. But at least my edits will give you a place to start. (You might like them).

By the way, the markup on the uploaded ms covers a few more things than the issue of tissue. These are editing suggestions. You do not have to address the markup in your rebuttal letter that will accompany your revision.

Line 52. "regulate and control". Both? How do these concepts differ? Pick one. But even better, use neither. I find it odd to say that 'molecule x regulates process y.' Biologists write like this all the time but in doing so they cheapen the concept of regulation almost to the point of meaninglessness. Regulation implies feedback; and for feedback you need a system. A cell or a plant or perhaps a pathway can be said to regulate a process. Here, you can write "They are involved in ..."

Line 85. Did the research in all of those papers measure active gibberellins only? That surprises me; usually both active and inactive ones are measured. Please check. By the way, why write 'bioactive'? How does that differ from 'active'? Where else would a gibberellin be active if not in an organism? I suggest removing the superfluous 'bio'.

Line 128. "forage". What does that mean? Webster's dictionary defines forage (used a noun): "Food for animals especially when taken by browsing or grazing." That is not specific. This kind of sample needs to be defined more fully.

Line 145. What is "OlChemlm"? Is this the name of a company? Provide more information (maybe a url?).

Line 248. What is "SLE"? Please spell it out here. You may use the acronym after that.

Line 260. And elsewhere. Do not capitalize every word in the figure legend title. In English publications, that style is reservd for books.

Please submit your revised manuscript by Oct 08 2026 11:59PM. If you will need more time than this to complete your revisions, please reply to this message or contact the journal office at ?>plosone@plos.org. When you're ready to submit your revision, log on to https://www.editorialmanager.com/pone/ and select the 'Submissions Needing Revision' folder to locate your manuscript file.

- A letter that responds to each point raised by the academic editor and reviewer(s). You should upload this letter as a separate file labeled 'Response to Reviewers'.
- A marked-up copy of your manuscript that highlights changes made to the original version. You should upload this as a separate file labeled 'Revised Manuscript with Track Changes'.
- An unmarked version of your revised paper without tracked changes. You should upload this as a separate file labeled 'Manuscript'.

If you would like to make changes to your financial disclosure, please include your updated statement in your cover letter. Guidelines for resubmitting your figure files are available below the reviewer comments at the end of this letter.

If applicable, we recommend that you deposit your laboratory protocols in protocols.io to enhance the reproducibility of your results. Protocols.io assigns your protocol its own identifier (DOI) so that it can be cited independently in the future. For instructions see: https://journals.plos.org/plosone/s/submission-guidelines#loc-laboratory-protocols. Additionally, PLOS One offers an option for publishing peer-reviewed Lab Protocol articles, which describe protocols hosted on protocols.io. Read more information on sharing protocols at https://plos.org/protocols?utm_medium=editorial-email&utm_source=authorletters&utm_campaign=protocols.

As the corresponding author, your ORCID iD is verified in the submission system and will appear in the published article. PLOS supports the use of ORCID, and we encourage all coauthors to register for an ORCID iD and use it as well. Please encourage your coauthors to verify their ORCID iD within the submission system before final acceptance, as unverified ORCID iDs will not appear in the published article. *Only* the individual author can complete the verification step; PLOS staff *cannot* verify ORCID iDs on behalf of authors.

We look forward to receiving your revised manuscript.

Kind regards,

Tobias Isaac Baskin

Academic Editor

PLOS One

**Journal Requirements:**

1. If the reviewer comments include a recommendation to cite specific previously published works, please review and evaluate these publications to determine whether they are relevant and should be cited. There is no requirement to cite these works unless the editor has indicated otherwise.

2. Please review your reference list to ensure that it is complete and correct. If you have cited papers that have been retracted, please include the rationale for doing so in the manuscript text, or remove these references and replace them with relevant current references. Any changes to the reference list should be mentioned in the rebuttal letter that accompanies your revised manuscript. If you need to cite a retracted article, indicate the article’s retracted status in the References list and also include a citation and full reference for the retraction notice.

[Note: HTML markup is below. Please do not edit.]

Reviewers' comments:

Reviewer's Responses to Questions

**Comments to the Author**

Reviewer #3: All comments have been addressed

Reviewer #4: (No Response)

**********

2. Is the manuscript technically sound, and do the data support the conclusions?

Reviewer #3: Partly

Reviewer #4: Yes

**********

3. Has the statistical analysis been performed appropriately and rigorously?

Reviewer #3: N/A

Reviewer #4: Yes

**********

4. Have the authors made all data underlying the findings in their manuscript fully available?

The PLOS Data policy

Reviewer #3: Yes

Reviewer #4: Yes

**********

5. Is the manuscript presented in an intelligible fashion and written in standard English?

Reviewer #3: Yes

Reviewer #4: Yes

**********

Reviewer #3: (No Response)

Reviewer #4: This paper investigates the genetic and environmental contributions to variations in GA1 and GA4 levels across maize tissues using linear mixed-effects models. The authors conclude that environmental conditions contribute more than genetic background in some tissues. The statistical methodology is sound, and below are some minor questions and comments:

- The authors indicate that an additional analysis was conducted treating Hybrid, Site, and the Hybrid-by-Site interaction as fixed effects (lines 222-232). It might be helpful to refer to this when you show results of this analysis, for example, I assume this model is used to calculate quantities in Fig.4, is that correct?

- The author imputes missing data with half of the smallest observed value of a certain tissue. Some sensitivity analysis appropriate for the missing data mechanism might be helpful to clarify whether this affects the estimated variance components, e.g., if the measurement tends to be truncated then you can use a model that assumes the response is truncated at a certain value.

- The authors mention that trial maintenance varied across sites. Could this be a potential confounder? It would be helpful if the author can discuss that a bit when they describe what the environmental effect entails.

- It might be helpful to clarify how the standard error in Fig. 3 calculated, for example whether this the standard errors of replicates within each site/hybrid combination or were calculated using an alternative method.

**********

what does this mean?). If published, this will include your full peer review and any attached files.

If you choose “no”, your identity will remain anonymous but your review may still be made public.

**Do you want your identity to be public for this peer review?** For information about this choice, including consent withdrawal, please see our Privacy Policy

Reviewer #3: No

Reviewer #4: No

**********

[NOTE: If reviewer comments were submitted as an attachment file, they will be attached to this email and accessible via the submission site. Please log into your account, locate the manuscript record, and check for the action link "View Attachments". If this link does not appear, there are no attachment files.]

To ensure your figures meet our technical requirements, please review our figure guidelines: https://journals.plos.org/plosone/s/figures

You may also use PLOS’s free figure tool, NAAS, to help you prepare publication quality figures: https://journals.plos.org/plosone/s/figures#loc-tools-for-figure-preparation.

NAAS will assess whether your figures meet our technical requirements by comparing each figure against our figure specifications.

Attachment Submitted filename: PONE-D-25-66749_R2TB.pdf

# Author comment: Author response to Decision Letter 3

**DOI:** 10.1371/journal.pone.0340156.r006

16 Sep 2026

PONE-D-25-66749R2

Natural variation of bioactive gibberellin levels in maize tissues across North America

PLOS One

Dear Dr. Wang,

Thank you for submitting your manuscript to PLOS One. After careful consideration, the Journal and I feel that your manuscript is much improved but does not yet entirely meet PLOS One’s publication criteria. A few minor issues remain. Therefore, we invite you to submit a revised version of the manuscript that addresses the points raised during the review process.

First, I apologize for the delay in getting this back to you. The original reviewer 1, who is a statistician, was unavailable. I needed to find another statistitian and this took me some time, especially because of the summer holidays. Happily, this new reviewer (number 4) is mostly satisified and brings up just a few points needing clarification. Reviewer 3 is completely happy.

I read the paper too and feel that it is in good shape. I have a few matters of language to call to your attention.

Response: Thanks for the great comments. please see our response below to each point:

Line 25 (and elsewhere). I suggest you delete the word 'endogenous'. How could a hormone be 'exogenous'? I think in most places you can delete the word 'endogenous'.

Response: “endogenous” in the paper was deleted.

Line 25. The word 'phytohomorme' was coined more than 100 years ago when scientists had little idea what was going on. Now we understand that plants have hormones. Of course they differ from those of animals, but there is no reason they would be the same. Other than the fact that they occur in plants, there is nothing that unites 'phytohormones' into a group. What is more, no one ever uses the word "zoohormones" to indicate animal hormones. There is no need. LIkewise, I can see no reason to use the term phytohormones. Writing 'hormones' is clear. No one would think you are writing about animal hormones.

Response: We thank the editor for this helpful suggestion. We agree that “plant hormones” is more appropriate in this context. Accordingly, we have revised “phytohormone(s)” to “plant hormone(s)” throughout the manuscript where applicable.

Line 30, and elsewhere. The word tissue is defined as a group of cells of a similar type, such as epidermis or mesophyll. A root is not a tissue. Unfortunately, people often write loosely about throwing plant tissue in the blender, using the word 'tissue' as a synonym for 'material'. In that case, the casual wording probably doesn't matter. But here, the source of the sample is a big part of the story. It strikes me as misleading for you to call your samples 'tissues' when they are not. I realize that because your samples are not all organs, there is not an obvious alternative. However, I have gone thru your paper and suggested revisions. Please see the uploaded marked manuscript. I don't know that these are the best choices, you might well find alternatives. But at least my edits will give you a place to start. (You might like them).

By the way, the markup on the uploaded ms covers a few more things than the issue of tissue. These are editing suggestions. You do not have to address the markup in your rebuttal letter that will accompany your revision.

Response: We thank the editor for the careful review and helpful suggested revisions. We understand the concern that the standalone use of “tissue” may be imprecise when referring to sample sources such as root, stalk, grain, or combined plant material. To improve clarity, we have reviewed the manuscript and revised the wording where appropriate. Specifically, we now use more precise terms such as “maize tissue samples,” “maize samples,” or “plant material” when referring to the specimens collected, processed, or analyzed. Where the text discusses biological differences among sample types such as leaf, root, stalk, pollen, and grain, we retain “maize tissues” because this terminology is commonly used in the plant science literature to describe different maize sample types in comparative analyses.

For example, similar usage can be found in PLOS ONE publications, including Lin et al. (2014), where maize root, stem, and leaf were described as different plant tissues, and Luís et al. (2016), where “maize-leaf tissue” was used to describe maize leaves. We have also considered the editor’s marked manuscript suggestions and incorporated revisions where they improved clarity and accuracy.

Reference 1

LinY, ZhangC, LanH, GaoS, LiuH,etal. (2014) Validation of Potential Reference Genes for qPCR in Maize across Abiotic Stresses, Hormone Treatments, and Tissue Types. PLoSONE9(5):e95445.doi:10.1371/journal.pone.0095445

Reference 2

Luís IM, Alexandre BM, Oliveira MM, Abreu IA (2016) Selection of an Appropriate Protein Extraction Method to Study the Phosphoproteome of Maize Photosynthetic Tissue. PLoS ONE 11(10): e0164387. https://doi.org/10.1371/journal.pone.0164387

Line 52. "regulate and control". Both? How do these concepts differ? Pick one. But even better, use neither. I find it odd to say that 'molecule x regulates process y.' Biologists write like this all the time but in doing so they cheapen the concept of regulation almost to the point of meaninglessness. Regulation implies feedback; and for feedback you need a system. A cell or a plant or perhaps a pathway can be said to regulate a process. Here, you can write "They are involved in ..."

Response: Thank you for your suggestion. In the manuscript, we changed “regulate and control” to “are involved in” and adjust the “Regulate” to “modulate” or “contribute to” when it fits.

Line 85. Did the research in all of those papers measure active gibberellins only? That surprises me; usually both active and inactive ones are measured. Please check. By the way, why write 'bioactive'? How does that differ from 'active'? Where else would a gibberellin be active if not in an organism? I suggest removing the superfluous 'bio'.

Response: We thank the editor for this helpful comment. We have checked the cited papers and revised the text to ensure that the description accurately reflects the gibberellins measured in those studies. In the present study, we measured GA1 and GA4, which are commonly recognized as bioactive gibberellins in plants.

Regarding the term “bioactive,” we respectfully prefer to retain it where appropriate because it has a specific meaning in the gibberellin literature. Although more than 130 gibberellins have been identified in plants, only a small subset, including GA1, GA3, GA4, and GA7, can bind the GID1 receptor and trigger GA signaling. Other gibberellins function as biosynthetic precursors or inactive catabolites. Therefore, “bioactive gibberellins” distinguishes the signaling-competent forms from inactive or precursor forms.

We agree that “active” and “bioactive” can appear similar in general usage; however, “bioactive gibberellins” is the established terminology used in the plant hormone field to refer specifically to receptor-active, signal-transducing gibberellins. This usage is supported by prior literature, including Tang et al. (2025), Rahman et al. (2026), and Hedden and Thomas (2012). Therefore, we have retained “bioactive gibberellins” where it is scientifically specific, while reviewing the manuscript to avoid unnecessary or unclear wording elsewhere.

Reference 1

Tang, J., Zhao, G., Tian, X., Bu, Q. and Li, W. (2025), Precise modulation of bioactive gibberellin homeostasis: A promising strategy for enhancing abiotic stress tolerance. J. Integr. Plant Biol., 67: 2247-2249. https://doi.org/10.1111/jipb.13942

Reference 2

Shafeeq Rahman, Shabanamol Subaida Beevi, Fathimath Nishana, Khaled Masmoudi, Rajesh Muliyar Krishna, Shyam Sreedhara Kurup, Gibberellin homeostasis and signaling in plant growth, stress adaptation, and crop improvement, Current Plant Biology, Volume 49,

2026, 100654, https://doi.org/10.1016/j.cpb.2026.100654.

Reference 3

Peter Hedden, Stephen G. Thomas; Gibberellin biosynthesis and its regulation. Biochem J 15 May 2012; 444 (1): 11–25. doi: https://doi.org/10.1042/BJ20120245

Line 128. "forage". What does that mean? Webster's dictionary defines forage (used a noun): "Food for animals especially when taken by browsing or grazing." That is not specific. This kind of sample needs to be defined more fully.

Response: We thank the editor for this helpful comment. We agree that the term “forage” should be defined more clearly in the manuscript. In this study, the “forage” sample refers to the aboveground portion of the maize plant collected by cutting the plant just above the soil surface. All aboveground plant parts, including leaves, ears, and stalks, were retained, cut into approximately 5-inch segments, and pooled for each plot. We have clarified this definition in the manuscript on lines 131–133.

Line 145. What is "OlChemlm"? Is this the name of a company? Provide more information (maybe a url?).

Response: Yes. “OlChemlm” is the company where the plant hormone standards were purchased. We have revised the manuscript to provide the full company name and website information for clarity: OlChemIm s.r.o. (OlChemIm s.r.o.., Czech Republic; https://olchemim.cz).

Line 248. What is "SLE"? Please spell it out here. You may use the acronym after that.

Response: “SLE” refers to supported liquid extraction. The acronym was first defined in the “Sample preparation” section as follows: “extracts were loaded into a Biotage 96-well supported liquid extraction (SLE) plate (Biotage, 8200400P01).” We have also reviewed the manuscript to ensure that the term is spelled out at first use and that the acronym is used consistently thereafter.

Line 260. And elsewhere. Do not capitalize every word in the figure legend title. In English publications, that style is reservd for books.

Response: We have revised the figure legend titles throughout the manuscript to use sentence case rather than title case.

Please submit your revised manuscript by Oct 08 2026 11:59PM. If you will need more time than this to complete your revisions, please reply to this message or contact the journal office at plosone@plos.org. When you're ready to submit your revision, log on to https://www.editorialmanager.com/pone/ and select the 'Submissions Needing Revision' folder to locate your manuscript file.

Please include the following items when submitting your revised manuscript:

• A letter that responds to each point raised by the academic editor and reviewer(s). You should upload this letter as a separate file labeled 'Response to Reviewers'.

• A marked-up copy of your manuscript that highlights changes made to the original version. You should upload this as a separate file labeled 'Revised Manuscript with Track Changes'.

• An unmarked version of your revised paper without tracked changes. You should upload this as a separate file labeled 'Manuscript'.

If you would like to make changes to your financial disclosure, please include your updated statement in your cover letter. Guidelines for resubmitting your figure files are available below the reviewer comments at the end of this letter.

If applicable, we recommend that you deposit your laboratory protocols in protocols.io to enhance the reproducibility of your results. Protocols.io assigns your protocol its own identifier (DOI) so that it can be cited independently in the future. For instructions see: https://journals.plos.org/plosone/s/submission-guidelines#loc-laboratory-protocols. Additionally, PLOS One offers an option for publishing peer-reviewed Lab Protocol articles, which describe protocols hosted on protocols.io. Read more information on sharing protocols at https://plos.org/protocols?utm_medium=editorial-email&utm_source=authorletters&utm_campaign=protocols.

As the corresponding author, your ORCID iD is verified in the submission system and will appear in the published article. PLOS supports the use of ORCID, and we encourage all coauthors to register for an ORCID iD and use it as well. Please encourage your coauthors to verify their ORCID iD within the submission system before final acceptance, as unverified ORCID iDs will not appear in the published article. Only the individual author can complete the verification step; PLOS staff cannot verify ORCID iDs on behalf of authors.

We look forward to receiving your revised manuscript.

Kind regards,

Tobias Isaac Baskin

Academic Editor

PLOS One

Journal Requirements:

1. If the reviewer comments include a recommendation to cite specific previously published works, please review and evaluate these publications to determine whether they are relevant and should be cited. There is no requirement to cite these works unless the editor has indicated otherwise.

2. Please review your reference list to ensure that it is complete and correct. If you have cited papers that have been retracted, please include the rationale for doing so in the manuscript text, or remove these references and replace them with relevant current references. Any changes to the reference list should be mentioned in the rebuttal letter that accompanies your revised manuscript. If you need to cite a retracted article, indicate the article’s retracted status in the References list and also include a citation and full reference for the retraction notice.

[Note: HTML markup is below. Please do not edit.]

Reviewers' comments:

Reviewer's Responses to Questions

Comments to the Author

1. If the authors have adequately addressed your comments raised in a previous round of review and you feel that this manuscript is now acceptable for publication, you may indicate that here to bypass the “Comments to the Author” section, enter your conflict of interest statement in the “Confidential to Editor” section, and submit your "Accept" recommendation.

Reviewer #3: All comments have been addressed

Reviewer #4: (No Response)

________________________________________

2. Is the manuscript technically sound, and do the data support the conclusions?

The manuscript must describe a technically sound piece of scientific research with data that supports the conclusions. Experiments must have been conducted rigorously, with appropriate controls, replication, and sample sizes. The conclusions must be drawn appropriately based on the data presented.

Reviewer #3: Partly

Reviewer #4: Yes

________________________________________

3. Has the statistical analysis been performed appropriately and rigorously?

Reviewer #3: N/A

Reviewer #4: Yes

________________________________________

4. Have the authors made all data underlying the findings in their manuscript fully available?

The PLOS Data policy requires authors to make all data underlying the findings described in their manuscript fully available without restriction, with rare exception (please refer to the Data Availability Statement in the manuscript PDF file). The data should be provided as part of the manuscript or its supporting information, or deposited to a public repository. For example, in addition to summary statistics, the data points behind means, medians and variance measures should be available. If there are restrictions on publicly sharing data—e.g. participant privacy or use of data from a third party—those must be specified.

Reviewer #3: Yes

Reviewer #4: Yes

________________________________________

5. Is the manuscript presented in an intelligible fashion and written in standard English?

PLOS ONE does not copyedit accepted manuscripts, so the language in submitted articles must be clear, correct, and unambiguous. Any typographical or grammatical errors should be corrected at revision, so please note any specific errors here.

Reviewer #3: Yes

Reviewer #4: Yes

________________________________________

6. Review Comments to the Author

Please use the space provided to explain your answers to the questions above. You may also include additional comments for the author, including concerns about dual publication, research ethics, or publication ethics. (Please upload your review as an attachment if it exceeds 20,000 characters)

Reviewer #3: (No Response)

Reviewer #4: This paper investigates the genetic and environmental contributions to variations in GA1 and GA4 levels across maize tissues using linear mixed-effects models. The authors conclude that environmental conditions contribute more than genetic background in some tissues. The statistical methodology is sound, and below are some minor questions and comments:

- The authors indicate that an additional analysis was conducted treating Hybrid, Site, and the Hybrid-by-Site interaction as fixed effects (lines 222-232). It might be helpful to refer to this when you show results of this analysis, for example, I assume this model is used to calculate quantities in Fig.4, is that correct?

Response: We thank the reviewer for this helpful suggestion. Yes. This model was used to calculate quantities shown in Fig 4. To make this clearer, we have revised the Results section of the manuscript, Lines 324-327, to state: “The analyses were conducted within a linear mixed model framework using PROC MIXED. Hybrid, Site, and the Hybrid-by-Site interaction were treated as fixed effects, whereas Replicate nested within Site was modeled as a random effect.”

- The author imputes missing data with half of the smallest observed value of a certain tissue. Some sensitivity analysis appropriate for the missing data mechanism might be helpful to clarify whether this affects the estimated variance components, e.g., if the measurement tends to be truncated then you can use a model that assumes the response is truncated at a certain value.

Response: We thank the reviewer for this helpful suggestion. To evaluate whether the handling of ND values affected the statistical conclusions, we conducted a sensitivity analysis using two imputation approaches: (1) substituting all ND values with a single value, defined as ½ of the LLOQ, and (2) substituting ND values with tissue-specific values, defined as ½ of the smallest observed value for each tissue, with the tissue-specific values provided in Supplemental Table S2. The results were consistent between the two approaches, including the estimated variance components and statistical significance. Therefore, the conclusions were not sensitive to the imputation method used for ND values.

Because the current approach is conventional for values below the detection or quantification threshold and produced consistent results across the sensitivity analysis, we retained this method in the revised manuscript.

- The authors mention that trial maintenance varied across sites. Could this be a potential confounder? It would be helpful if the author can discuss that a bit when they describe what the environmental effect entails.

Response: We agree that differences in trial maintenance among sites could potentially contribute to confounding between Hybrid and Site (environment), particularly given the imbalanced design of this experiment. In the manuscript, we describe the nature of this imbalance and discuss its implications for interpreting hybrid-by-environment interactions. The mixed effects model was selected to account for site-related variation and the imbalance in the data when estimating treatment effects. Therefore, the interaction estimates presented in Table 6 are model adjusted estimates that account for these factors.

It might be helpful to clarify how the standard error in Fig. 3 calculated, for example whether this the standard errors of replicates within each site/hybrid combination or were calculated using an alternative method.

Response: Thanks for the comments. The error bars in Fig. 3 represent the standard errors of the predicted mean for each site/hybrid combination from fitting the mixed-effect model. We have clarified this in the manuscript (lines 304-305) by adding the sentence “The standard errors are for the predicted mean of each site/hybrid combination from fitting the mixed-effect model”.

________________________________________

7. PLOS authors have the option to publish the peer review history of their article (what does this mean?). If published, this will include your full peer review and any attached files.

If you choose “no”, your identity will remain anonymous but your review may still be made public.

Do you want your identity to be public for this peer review? For information about this choice, including consent withdrawal, please see our Privacy Policy.

Reviewer #3: No

Reviewer #4: No

________________________________________

[NOTE: If reviewer comments were submitted as an attachment file, they will be attached to this email and accessible via the submission site. Please log into your account, locate the manuscript record, and check for the action link "View Attachments". If this link does not appear, there are no attachment files.]

To ensure your figures meet our technical requirements, please review our figure guidelines: https://journals.plos.org/plosone/s/figures

You may also use PLOS’s free figure tool, NAAS, to help you prepare publication quality figures: https://journals.plos.org/plosone/s/figures#loc-tools-for-figure-preparation.

NAAS will assess whether your figures meet our technical requirements by comparing each figure against our figure specifications.

Attachment Submitted filename: Response to Reviwers.docx

# Editor report: Decision Letter 3

**DOI:** 10.1371/journal.pone.0340156.r007

**Authors:** Tobias Baskin

17 Sep 2026

Natural variation of bioactive gibberellin levels in maize tissues across North America

PONE-D-25-66749R3

Dear Dr. Wang,

We’re pleased to inform you that your manuscript has been judged scientifically suitable for publication and will be formally accepted for publication once it meets all outstanding technical requirements.

Thank you for your careful revision and clear response letter. While I have your attention, on the matter of 'active' versus 'bioactive, I think that improving language is reasonable. I suppose people used active vs bioactive to distinguish those types of gibberellin. Most scientists do not think carefully about language and so those terms got adopted by default. But now that we stop and think we realise that adding 'bio' is stupid. Yes, active vs inactive matters. Why not bioactive vs bioinactive? Also stupid. Some terms are difficult to change; for example, the gene name "sonic hedgehog" comes from a video game but now is recognized as being important in certain human diseases. Patients who are told that their disease is caused by a mutation in sonic hedgehog report feeling upset. Nevertheless changing that name would be difficult because the gene name is so widespread. However, in the context of gibberellin, bioactive is read as an adjective (rather than a name). I think no one would be confused by reading 'active' gibberellin. I think it is worth adopting more sensible language wherever possible. I think it is possible in this case. Anyway thank you for considering.

Within one week, you’ll receive an e-mail detailing the required amendments. When these have been addressed, you’ll receive a formal acceptance letter and your manuscript will be scheduled for publication.

An invoice will be generated when your article is formally accepted. Please note, if your institution has a publishing partnership with PLOS and your article meets the relevant criteria, all or part of your publication costs will be covered. Please make sure your user information is up-to-date by logging into Editorial Manager at Editorial Manager® and clicking the ‘Update My Information' link at the top of the page. For questions related to billing, please contact billing support.

If your institution or institutions have a press office, please notify them about your upcoming paper to help maximize its impact. If they’ll be preparing press materials, please inform our press team as soon as possible -- no later than 48 hours after receiving the formal acceptance. Your manuscript will remain under strict press embargo until 2 pm Eastern Time on the date of publication. For more information, please contact onepress@plos.org.

Kind regards,

Tobias Isaac Baskin

Academic Editor

PLOS One

Additional Editor Comments (optional):

Reviewers' comments:

# Editor report: Acceptance letter

**DOI:** 10.1371/journal.pone.0340156.r008

**Authors:** Tobias Baskin

PONE-D-25-66749R3

PLOS One

Dear Dr. Wang,

I'm pleased to inform you that your manuscript has been deemed suitable for publication in PLOS One. Congratulations! Your manuscript is now being handed over to our production team.

At this stage, our production department will prepare your paper for publication. This includes ensuring the following:

* All references, tables, and figures are properly cited

* All relevant supporting information is included in the manuscript submission,

* There are no issues that prevent the paper from being properly typeset

You will receive further instructions from the production team, including instructions on how to review your proof when it is ready. Please keep in mind that we are working through a large volume of accepted articles, so please give us a few days to review your paper and let you know the next and final steps.

Lastly, if your institution or institutions have a press office, please let them know about your upcoming paper now to help maximize its impact. If they'll be preparing press materials, please inform our press team within the next 48 hours. Your manuscript will remain under strict press embargo until 2 pm Eastern Time on the date of publication. For more information, please contact onepress@plos.org.

You will receive an invoice from PLOS for your publication fee after your manuscript has reached the completed accept phase. If you receive an email requesting payment before acceptance or for any other service, this may be a phishing scheme. Learn how to identify phishing emails and protect your accounts at https://explore.plos.org/phishing.

If we can help with anything else, please email us at customercare@plos.org.

Thank you for submitting your work to PLOS One and supporting open access.

Kind regards,

PLOS One Editorial Office Staff

on behalf of

Dr. Tobias Isaac Baskin

Academic Editor

PLOS One
